# Developing a nomogram for preoperative prediction of cervical cancer lymph node metastasis by multiplex immunofluorescence

**DOI:** 10.1186/s12885-023-10932-0

**Published:** 2023-05-30

**Authors:** Jiangchun Wu, Qinhao Guo, Jun Zhu, Yong Wu, Simin Wang, Siyuan Liang, Xingzhu Ju, Xiaohua Wu

**Affiliations:** 1 Department of Oncology, Shanghai Medical College, Fudan University, 200032 Shanghai, China; 2 Department of Gynecologic Oncology, Fudan University Shanghai Cancer Center, Fudan University, 200032 Shanghai, China; 3 Department of Gastric Surgery, Fudan University Shanghai Cancer Center, Shanghai, China; 4 Department of Gynecologic Oncology, Fudan University Shanghai Cancer Center, Shanghai, 200000 PR China

**Keywords:** Cervical cancer, Preoperative individualized prediction, Multiplex immunofluorescence, Nomogram, Internally validation

## Abstract

**Background:**

Most traditional procedures can destroy tissue natural structure, and the information on spatial distribution and temporal distribution of immune milieu in situ would be lost. We aimed to explore the potential mechanism of pelvic lymph node (pLN) metastasis of cervical cancer (CC) by multiplex immunofluorescence (mIF) and construct a nomogram for preoperative prediction of pLN metastasis in patients with CC.

**Methods:**

Patients (180 IB1-IIA2 CC patients of 2009 FIGO (International Federation of Gynecology and Obstetrics)) were divided into two groups based on pLN status. Tissue microarray (TMA) was prepared and tumor-infiltrating immune markers were assessed by mIF. Multivariable logistic regression analysis and nomogram were used to develop the predicting model.

**Results:**

Multivariable logistic regression analysis constructs a predictive model and the area under the curve (AUC) can reach 0.843. By internal validation with the remaining 40% of cases, a new ROC curve has emerged and the AUC reached 0.888.

**Conclusions:**

This study presents an immune nomogram, which can be conveniently used to facilitate the preoperative individualized prediction of LN metastasis in patients with CC.

**Supplementary Information:**

The online version contains supplementary material available at 10.1186/s12885-023-10932-0.

## Background

Cervical cancer (CC) is one of the leading malignant tumors in the United States [1, 2]. Lymph node (LN) metastasis has been considered a vital factor in the development of CC [3–6]. Patients with this stage IB1-IIA2 CC (2009 International Federation of Gynecology and Obstetrics (FIGO) stage) will undergo pelvic LN (pLN) resection [7] and cause a variety of complications, including bleeding, nerve injury, lower pelvic lymphocele, and extremity lymphedema [8–11]. Thus, it will be particularly important to further study the mechanism of CC pLN metastasis. Furthermore, preoperative assessment of pLN status in CC patients is therefore critical for clinical decision-making.

Increasing evidence has demonstrated that pLN metastasis is a complex process involving tumor immune milieu [12–15]. However, molecular mechanisms behind metastasis processes remain obscure. Traditional procedures have been used to elucidate the mechanism by regularly exploring molecular pathways [14, 16, 17]. However, due to the tissue-destructive nature of most of these methods, the spatial distribution and temporal distribution of the immune milieu in situ will not be preserved [18]. Although morphological examination including conventional immunohistochemistry (IHC) or immunofluorescence (IF) can reveal some information, its effect is extremely limited because of high inter-observer variability and the capacity to label only one marker per tissue Sect. [19].

Multiplex immunohistochemistry/immunofluorescence (m-IHC/IF) has emerged and provides high-throughput multiplex staining and further standardized quantitative analysis for highly efficient, reproducible, and cost-effective tissue studies [19–21]. It can show up to seven targets simultaneously on a single slide. Afterward, HALO (Indica Labs, Albuquerque, USA), an image analysis system not only can be used for quantitative tissue analysis but also can reveal the spatial location of each target [22–26].

In the current study, we sought to comprehensively compare the quantification of immune markers and their spatial orientation interrelation between CC in situ tissue with positive pLN and negative pLN. Based on differential expression of immune-related indicators, a clinical prediction model was constructed for individual preoperative prediction of pLN metastasis and an internal validation was assessed. In the future, we can predict pLN metastasis by cervical biopsy, thus avoiding its dissection and improving patients’ quality of life.

## Materials and methods

### Microarray dataset collection and data process

To analyze the differentially expressed genes between LN metastasis and non-LN metastasis in cervical squamous cell carcinoma (CESC). We are using R language package (http://bioconductor.org/bioclite.R), DESeq2 do variance analysis. The DESeqDataSet objects required by DEseq2 were constructed by using the DESeq2 package, reading the representation matrix and creating the grouping matrix. DEseq was used for gene differential expression analysis, summary function was used to view the up-down-regulated distribution of gene expression, and finally, differential expression genes were extracted and stored in csv files. To get the difference of gene analysis by setting |log2(FC)|>1, *P* < 0.05, FDR < 0.05, corresponding differentially expressed genes were obtained.

Then, we downloaded the mRNA expression data through the cancer genome atlas TCGA database, extracted the data, used “perl” to put all the extracted files into the same folder, and used the script “mRNA_merge” to merge the extracted files and obtain the mRNA matrix. The “metadata” file is also used to obtain information about the number of node-negative and node-positive samples. After running, the organized expression matrix file and sample number information of the two groups can be obtained. Subsequently, ID in the expression matrix was transformed to extract mRNA expression information. The expression information is further standardized with “limma package”. After finishing, CIBERSORT method was used to calculate the immune cell composition information in each sample. Among them, we integrate the tool source code into R, and “run.R” can be done through reference file “ref” and source code. At the end of the run, we can get a “CIBERSORT-Results” file. The results are then filtered to remove the ones that don’t make sense. Run “perl” and “filter” directly. At the end of the run, all that’s left is the sample information and the immune cell information.

### Patient cohort

All procedures were ethically approved by the institutional Ethics Review Committee of Fudan University Shanghai Cancer Center (FUSCC). Appropriate written informed consent was obtained from all patients before sample collection. All experimental methods comply with relevant regulations and ethical principles.

A retrospective cohort study was conducted in the Department of Gynecology Oncology, FUSCC, which included 180 patients with the 2009 FIGO stage IB1-IIA2 who underwent radical abdominal hysterectomy with or without bilateral salpingo-oophorectomy and pelvic ± para-aortic lymphadenectomy from 2009 to 2012. All the enrolled patients had undergone standard pelvic lymphadenectomy by an experienced gynecological oncologist. All the microscopic slides were reviewed by the same professional gynecologic pathologist and were reconfirmed by another experienced gynecologic pathologist. A total of patients (90 with positive pLN and 90 with negative pLN) were assayed and their tissue specimens and clinical records were retrospectively studied.

### Making tissue microarray (TMA)

The 180 CC tissues (in situ) with pLN positive metastasis or negative metastasis were prepared into TMA as previously described [27–29]. We used paraffin-embedded tissue chips from our center’s tissue sample bank. Firstly, tissue microtome fine needle drilling was used to collect dozens to hundreds of small cylindrical tissues from all tissue wax blocks (donor wax blocks), and they were neatly arranged in another empty white wax block (recipient wax blocks) to make tissue chip wax blocks. The wax block is then sliced and the slice is transferred to a slide to make the TMA.

The detailed steps are as follows: First make 45 mm * 20 mm acceptor wax block. Drill holes in the wax blocks approximately 0.1 mm apart and 0.6 mm in diameter, and accurately locate the coordinates of each hole. Secondly, representative points on the wax block of the sample were labeled according to HE staining, including the LN positive CC tissue and the LN negative CC tissue. Select the corresponding points on the tissue of the wax block and use a stainless steel needle (inner diameter 0.6 mm; Depth: 2-3 mm), fixed in the hole of the receiving wax block. Finally, the acceptor wax blocks were precooled at 4℃ for about 4 h, and the whole tissue was corrected using a microtome. 30–50 sections with a thickness of 5 μm were quickly generated and applied to slides.

### M-IF staining protocol

Opal 7-colour kit (NEL811001KT, PerkinElmer) was used for mIF. TMAs were dewaxed and rehydrated. In the first step, the antigen was retrieved at 125 ℃ for 3 min and then cooled to room temperature (RT). Washed with TBST three times for 5 min, incubated in H_2_O_2_ for 10 min. Repeated washed and blocked with blocking buffer. The primary antibody, PDL-1 (ab237726, Abcam, 1:500, dye 480) was incubated at RT for 30 min. Slides were washed and an HRP-conjugated secondary antibody was incubated at RT for 10 min. TSA dye (1:100) was applied for 10 min after washes. The procedures were repeated six times using the following antibodies, CD3 (ab16669, Abcam, 1:200, dye 690; used as T lymphocyte cell marker [30]), CD8 (ab93278, Abcam, 1:100, dye 570; used as cytotoxic T cell marker [31]), CD56 (ab75813, Abcam, 1:500, dye 620; used as NK cell marker [32]), CD68 (ab213363, 1:1000, Abcam, dye 780; used as pan-macrophage marker [33]), programmed death-1 (PD-1) (ab237728, Abcam, 1:300, dye 520), programmed death ligand-1 (PD-L1) (ab237726, 1:500, dye 480) [34]. Secondary antibodies anti-mouse (NEF822001EA, PerkinElmer) or anti-rabbit (NEF812001EA, PerkinElmer) were used at a 1:1000 dilution.

With further analysis by the HALO system, we quantified the number of six immune targets and the spatial position relationship between them [35].

### Constructing a diagnostic prediction model

By comparing the differences in the quantitative and spatial distribution of immune markers, we further constructed the diagnosis and prediction model of CC LN metastasis by R [36, 37].

### Validation of the prediction nomogram

We used R language to randomly select 40% of 180 cases of cervical cancer for internal validation of the diagnostic prediction model.

### Statistical analysis

Statistical analysis was performed with Graphpad Prism 8 and R language. Unsupervised hierarchical clustering was conducted to define the immune subtypes by different markers. Comparisons between two conditions were based on a two-sided Student’s test. *P<0.05 were judged to be statistically significant. See the supplementary material for the code in R.

## Results

### TCGA of cervical squamous cell carcinoma (CESC)

**Fig. 1:**
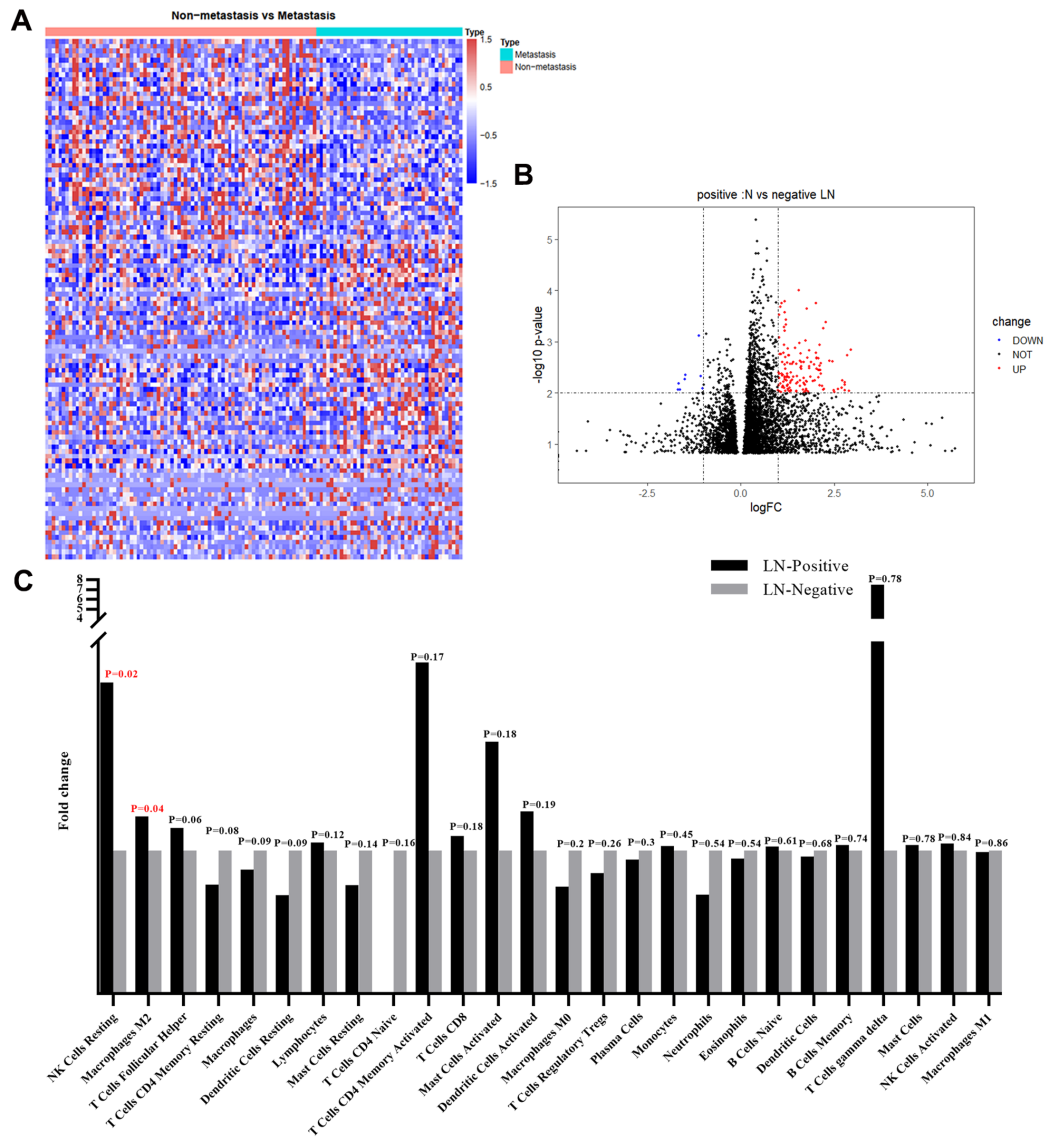
TCGA of cervical squamous cell carcinoma (CESC). **A** By TCGA analysis, differentially expressed genes in Cervical cancer with or without LN metastasis have been showed in heatmap and (**B**) volcano plot. **C** Differentially expressed immune related cells were presented in histogram

Our TCGA data have systematically analyzed the differential gene makeup in the two groups. The expression profiles of fragments per kilobase of transcript per million fragments mapped (FPKM) based on TCGA Cervical Squamous Cell Carcinoma (CESC) were used to analyze the expression differences of non-metastasis and metastasis in CC patients. The differential screening criteria were set at |log2(FC)|>1, *P<0.05, FDR<0.05 and the results showed that forty-two genes were upregulated and sixty-seven genes were downregulated (Fig. 1A and Fig. B).

Then, we analyzed the relationship between CC LN metastasis and immune cell infiltration through the TCGA database. The results showed that the infiltration difference of NK cells Resting and macrophage M2 in different groups was statistically significant (Fig. 1C).

### Patient characteristics

The study cohort contained 180 cases of CC with high-quality TMA. The median age of the patients was 46 years (range, 23–71 years). The histological diagnosis was all squamous cell carcinoma and based on the FIGO 2009 guidelines, nineteen (21.1%) subjects were stage IB1, twenty (22.2%) subjects were stage IB2, twenty-nine (32.3%) subjects were stage IIA1, and twenty-two (24.4%) subjects were stage IIA2. The median follow-up time was 61.05 months (range, 7.93–78.50 months) while thirteen (14.4%) subjects relapsed and seven (7.8%) subjects died (Table 1).

**Table 1:** Patients’ Characteristics

Characteristics	N (%/Range)
**Number**	188 (100%)
**Median age (years)**	46 (23–71)
**FIGO Stage**	
IB1	38 (21.1%)
IIIB	40 (22.2%)
IIIC	58 (32.3%)
IV	44 (24.4%)
**Histologic type**	
Squamous cell carcinoma	180 (100%)
**Follow-up time**	61.05 (7.93–78.50)
**Outcome**	
Relapsed	26 (14.4%)
Died	14 (7.8%)
Other	140 (77.78%)

### Immune infiltrates in CC

**Fig. 2:**
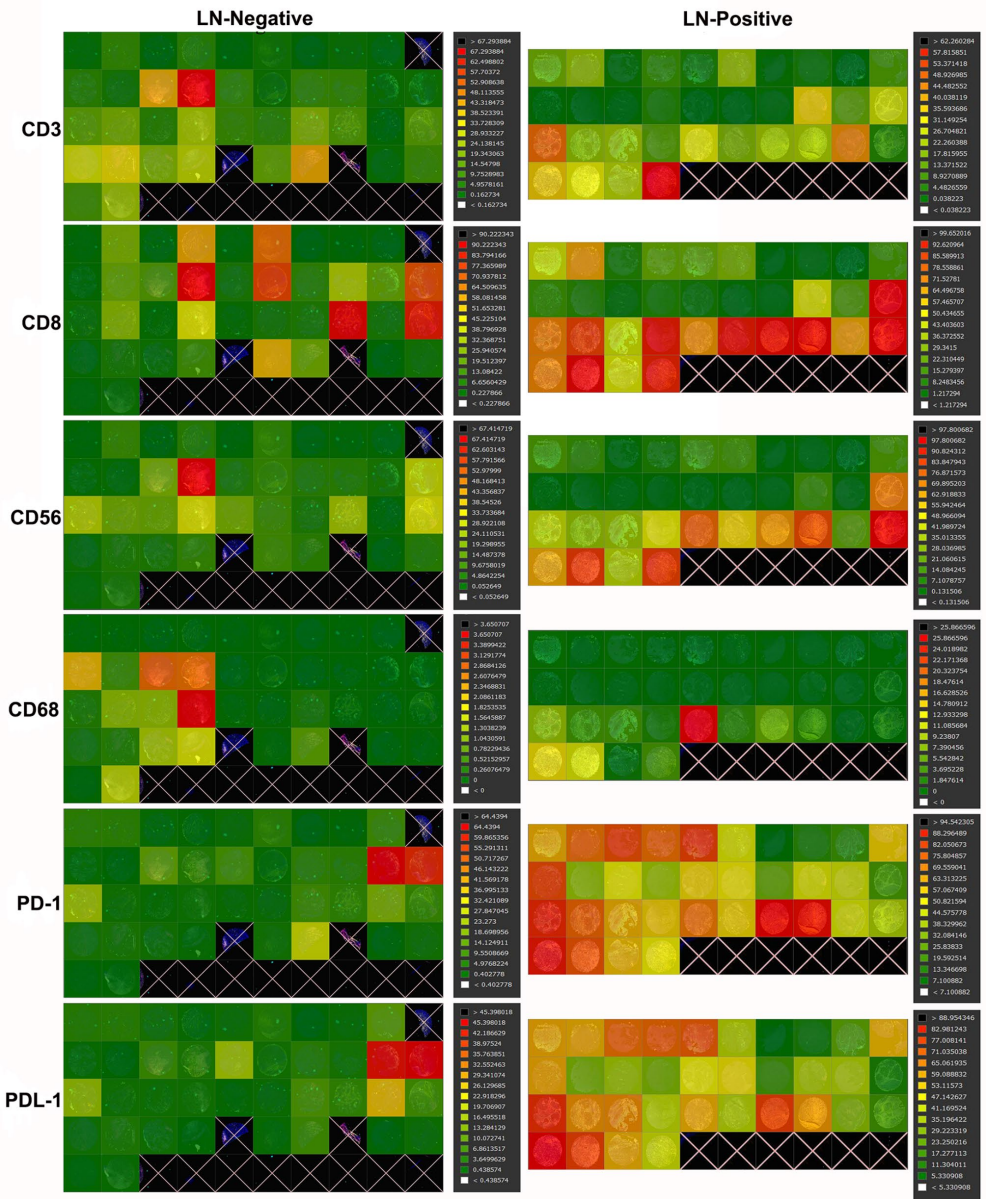
Heatmaps of immune infiltrates in cervical cancer (CC). Representative heatmaps of two groups with antibodies against CD3, CD8, CD56, CD68, PD-1, PD-L1. The right-hand column shows the proportions represented by different shades of heatmap color

By mIF and HALO system was further applied to scan the relative ratio of each target on each tissue on the TMA [38, 39]. The results can be graphically presented in heatmaps and their color can be digitized (Fig. 2).

**Fig. 3:**
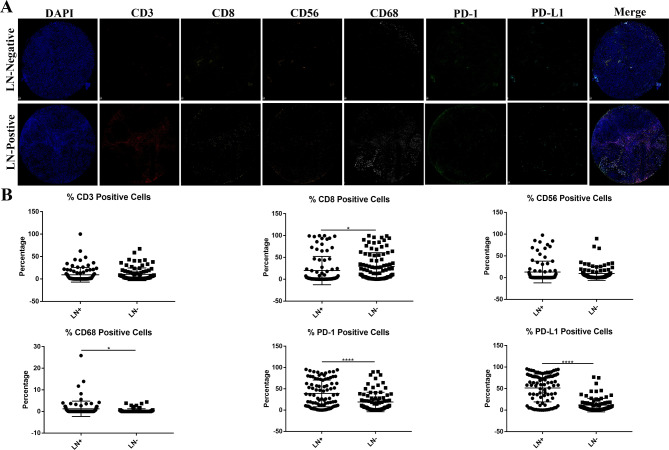
Multiplex immunofluorescence (mIF) of Immune infiltrates in cervical cancer (CC). **A** Representative image of two groups by multiplex immunofluorescence (mIF) with antibodies against CD3, CD8, CD56, CD68, PD-1, PD-L1. **B** Comparison of different immune markers proportion. The percentage of CD3 positive cells, CD56 positive cells have no difference between two groups, while the proportion of CD8 decreased and the proportion of CD68, PD-1, PD-L1 increased with lymph node (LN) metastasis

To better understand the complex immune characteristics in CC, we quantified the immune stains by the HALO system. These six immunologic markers revealed distinct positive staining ratios in 180 samples. We observed CD8 marker (19.87% ± 3.47% vs. 29.65% ± 3.31%; *P*<0.05), CD68 + macrophages (1.23% ± 0.38% vs. 0.35% ± 0.94%; *P*<0.05), PD-1 (39.23% ± 3.37% vs. 19.06% ± 2.43%; *P*<0.0001), PD-L1 (51.41% ± 3.47% vs. 11.62% ± 1.67%; *P*<0.0001) in CC with positive pLN and negative pLN respectively (Fig. 3).

In a word, our results demonstrated that the positive proportion of CD68, PD-1 and PD-L1 immune cell infiltration were significantly up-regulated with pLN metastasis, while CD8 was significantly decreased, CD3 and CD56 did not change.

### Distinct spatial distribution of immune infiltration

The HALO can not only quantify the number of immune cells on a panel but also locate their position, and measure their spatial distance [20, 40, 41]. In this way, we can further find the relative number and location of immune cells in situ tissue of CC after pLN metastasis more visually and thereby draw the relationship of immune cells which would promote LN metastasis.

**Fig. 4:**
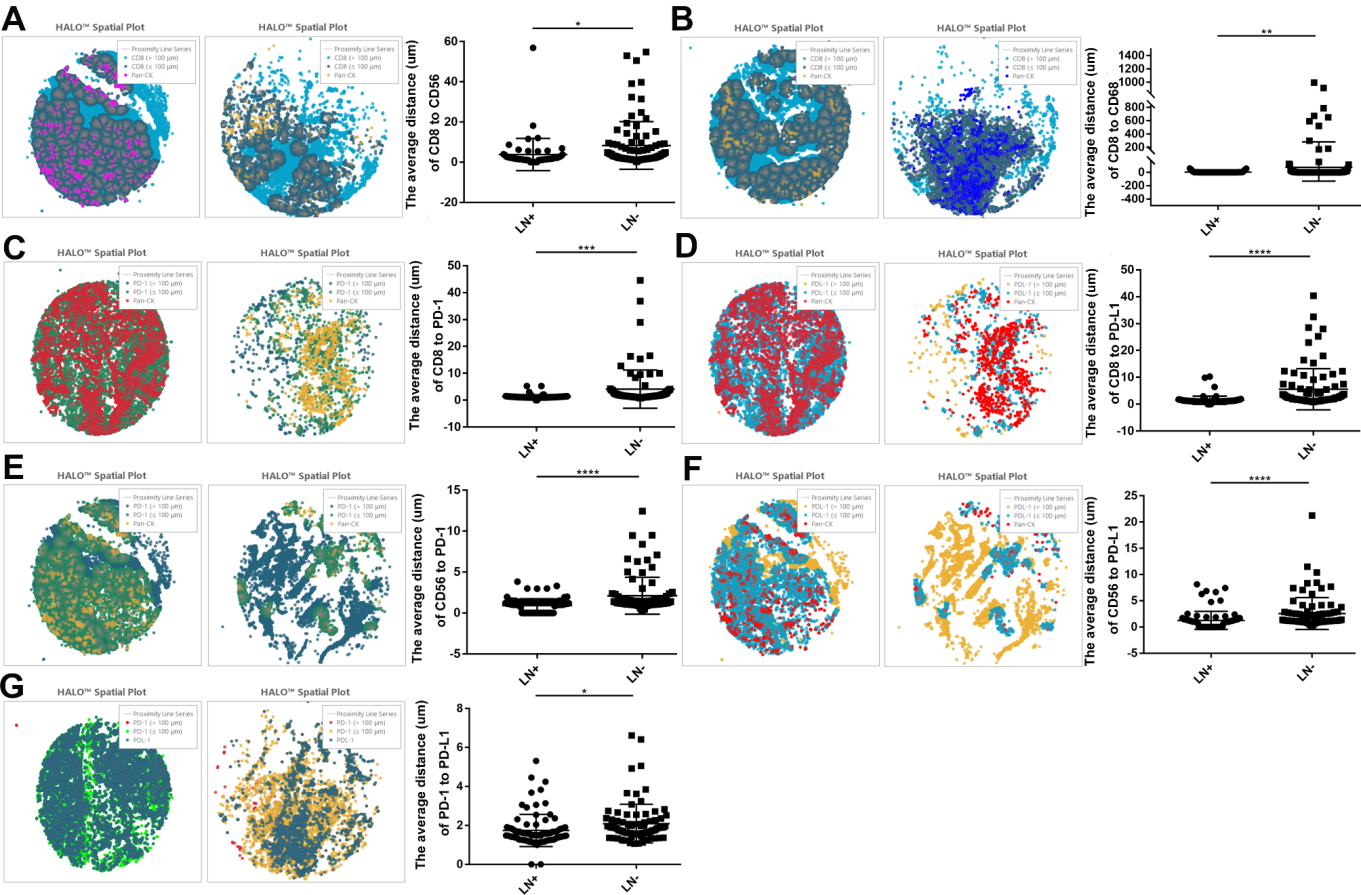
Distinct spatial distribution of immune infiltration. **A** The average distance (um) of CD8 to CD56 of the two groups. **B** The average distance (um) of CD8 to CD68 of the two groups. **C** The average distance (um) of CD8 to PD-1 of the two groups. **D** The average distance (um) of CD8 to PD-L1. **E** The average distance (um) of CD56 to PD-1. **F** The average distance (um) of CD56 to PD-L1. **G** The average distance (um) of PD-1 to PD-L1.

The average distance (um) of CD8 to CD56 was 8.33 ± 1.26% in the pLN-negative group, 3.86 ± 1.08% in the pLN-positive group (pLN-negative versus pLN-positive, *P* < 0.05) (Fig. 4A). The average distance (um) of CD8 to CD68 was 75.08 ± 21.91% in the pLN-negative group, 5.56 ± 1.62% in the pLN-positive group (pLN-negative versus pLN-positive, *P* < 0.01) (Fig. 4B). The average distance (um) of CD8 to PD-1 was 4.12 ± 0.76% in the pLN-negative group, 1.30 ± 0.08% in the pLN-positive group (pLN-negative versus pLN-positive, *P* < 0.001) (Fig. 4C). The average distance (um) of CD8 to PD-L1 was 5.58 ± 0.81% in the pLN-negative group, 1.50 ± 0.16% in the pLN-positive group (pLN-negative versus pLN-positive, *P* < 0.0001) (Fig. 4D). In these data, CD8 + T cells were significantly close to NK cells, macrophages and tumor cells with pLN metastasis.

The average distance (um) of CD56 to PD-1 was 2.11 ± 0.24% in the pLN-negative group, 0.91 ± 0.09% in the pLN-positive group (pLN-negative versus pLN-positive, *P* < 0.0001) (Fig. 4E). The average distance (um) of CD56 to PD-L1 was 2.57 ± 0.32% in the pLN-negative group, 1.26 ± 0.18% in the pLN-positive group (pLN-negative versus pLN-positive, *P* < 0.0001) (Fig. 4F). Thus, NK cells were significantly close to tumor cells with pLN metastasis.

The average distance (um) of PD-1 to PD-L1 was 2.09 ± 0.10% in the pLN-negative group, 1.74 ± 0.09% in the pLN-positive group (pLN-negative versus pLN-positive, *P* < 0.05) (Fig. 4G).

### Development of an individualized prediction model

**Fig. 5:**
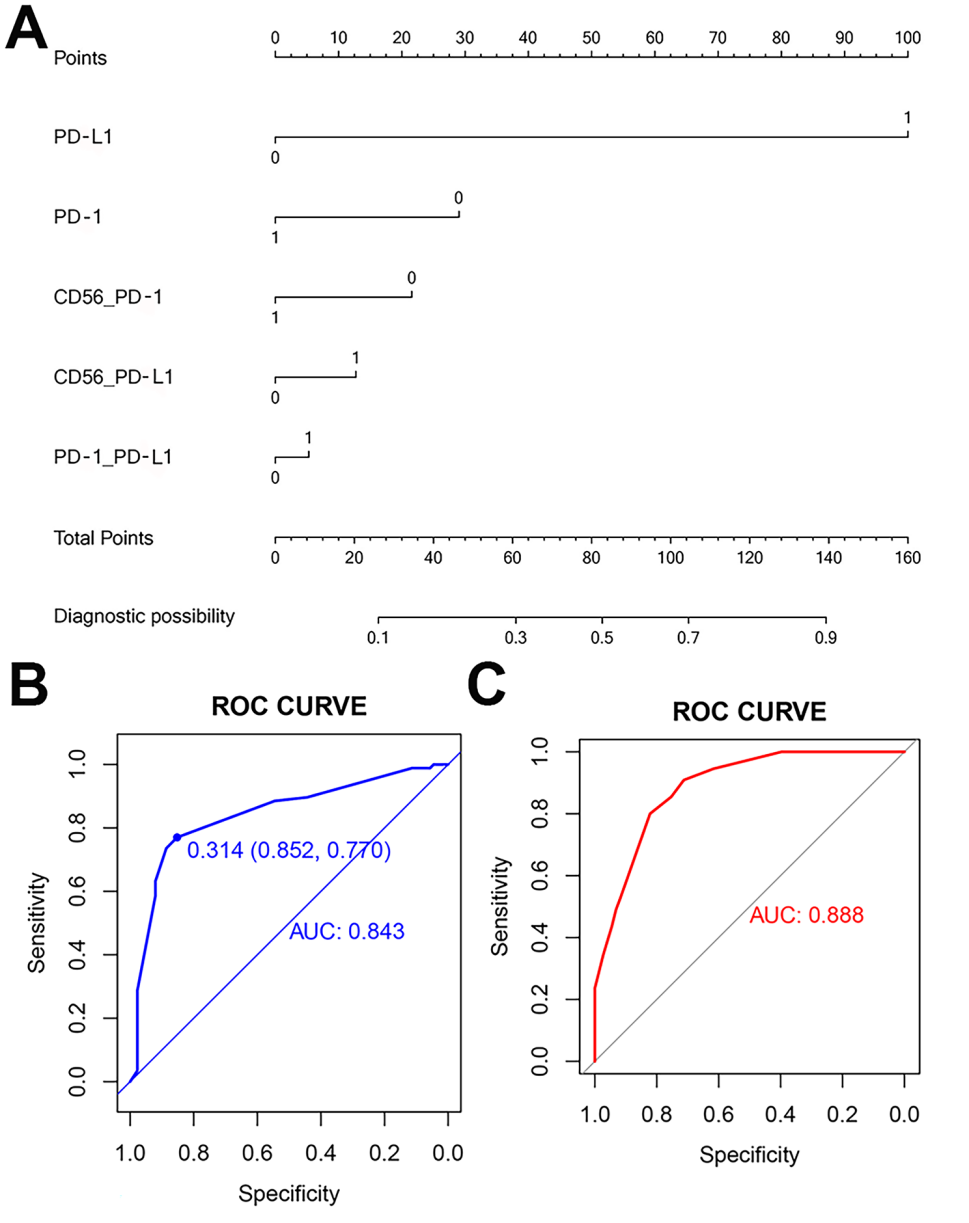
Developed immune nomogram and Internally Validation. **A-B** The immune nomogram was developed in the cohort, with the immune signature, PD-1, PDL-1, the distance between CD56 and PD-1, CD56 and PDL-1, PD-1 and PDL-1 incorporated. **C** Internally validation. ROC = receiver operator characteristic. AUC = area under curve

Multivariable logistic regression analysis began with the following immune variables: CD3, CD8, CD56, CD68, PD-1, PD-L1, the distance between CD8 and CD56, CD8 and CD68, CD8 and PD-1, CD8 and PD-L1, CD56 and PD-1, CD56 and PD-L1, PD-1 and PDL-1. The immune signature was applied to develop a diagnostic model for pLN metastasis by using the two groups. A ROC curve is made for each meaningful marker and every cutoff value has emerged. Thereafter, these cutoff values are divided into two groups. At this point, we can make a diagnostic ROC curve and the AUC reaches 0.843 (Fig. 5A). The model that incorporated these above independent predictors was developed and presented as the nomogram (Fig. 5B).

### Internally validation of the prediction model

With the nomogram, we then tested the remaining 40% of cases and obtained a ROC curve with an AUC reaching 0.888. (Fig. 5C).

## Discussion

Patients with the 2009 FIGO stage of IB1-IIA2 CC will undergo pLN resection and impair the patient’s quality of life [8, 42]. Current studies have shown that LN metastasis of CC is related to a variety of complex factors, especially the close relationship with immunity, but its specific mechanism remains unclear[19, 43–45]. Therefore, it is very important to find the mechanism of CC metastasis. If LN metastasis can be predicted before surgery, it can avoid dissection during surgery, thus providing patients with quality of life.

Firstly, bioinformatics was used to analyze the differentially expressed genes in the LN-negative and LN-positive groups in the TCGA data, and the results showed that 42 genes were up-regulated, while 67 genes were down-regulated, including immune-related genes. Further analysis of the relationship between LN metastasis and immune cell invasion in TCGA showed that the infiltration of NK cells and M2-type macrophages increased significantly with LN metastasis. These results suggest that NK cells and M2 macrophages may play an important role in LN metastasis of CC.

We have previously demonstrated that SPOP gene can promote the metastasis of cervical cancer by regulating the spatial distance between PD-1 and PD-L1 genes[29]. It is concluded that the infiltration of immune cells and the interaction between immune molecules reflected by spatial distance may play an important role in lymph node metastasis of cervical cancer. Therefore, by multicolor immunofluorescence and HAO analysis, it was found that with lymph node metastasis of cervical cancer, the number of invasion of CD68 increased significantly, and the spatial distance between CD68 and CD8 decreased significantly. CD68 is the main marker of macrophage expression, and CD8 is the main surface marker of cytotoxic T cells. This indicated a significant increase in macrophage infiltration, which was consistent with the results of our bioinformatics analysis. The surface distance between macrophages and CD8 toxic T cells was significantly reduced, indicating that their possible interactions were significantly increased. However, our results suggest that the number of CD8 cells is significantly reduced, indicating that macrophages interact with CD8 cytotoxic T cells to inhibit CD8 positive T cell infiltration by increasing their own immune infiltration.

CD3 was a marker on the surface of universal T cells, and CD68 had no effect on the number and spatial distance of CD3-positive T cells. However, CD8 positive T cell expression was significantly decreased in the lymph node metastasis group, indicating that CD4 positive T cell infiltration was significantly increased. However, there was no difference in the spatial distance between CD68 and the total T cells (CD3) of the two groups, indicating that the distance between CD68 and CD4 positive T cells increased, indicating that CD68 had no direct effect on CD4. The above results showed that with lymph node metastasis, macrophage infiltration increased significantly, directly inhibiting CD8 positive T cell infiltration and indirectly promoting CD4 positive T cell infiltration.

Our HALO analysis showed no change in CD56 expression with lymph node metastasis. CD56 was mainly a surface marker of NK cells, indicating no change in NK cell infiltration. However, by analyzing the spatial distance of NK cells, it was found that with lymph node metastasis, the spatial distance between CD56 and CD8, PD-1 and PD-L1 was significantly close. The expressions of PD-1 and PD-L1 were significantly increased in the lymph node metastasis group. PD-1 is mainly expressed on the surface of activated T cells and B cells. This indicated increased infiltration of activated T cells and B cells in the lymph node metastasis group. In addition to being expressed on the tumor surface and participating in immune escape, PD-L1 is also expressed on the surface of antigen presenting cells (DC cells, macrophages, etc.) and vascular endothelial cells under the stimulation of IFN-γ, which indicates that the infiltration of tumor cells, DC cells or macrophages increases in the lymph node metastasis group. The spatial distance between CD56 and CD68 did not change. The above indicated that NK cell infiltration did not increase with lymph node metastasis, but it could inhibit its infiltration by interacting with CD8 positive T cells, promote its infiltration by interacting with activated T cells or B cells, and promote its infiltration by interacting with tumor cells or DC cells.

In addition, the spatial distance between CD8 and PD-1 or PD-L1 decreased, indicating that CD8 positive T cells could interact with activated T cells or B cells to promote their infiltration, and interact with tumor cells or DC or macrophages to promote their infiltration, while their own infiltration was inhibited.

Through univariate analysis of the above differentially expressed immune cells and the spatial distance between each other, the meaningful factors were included in the multivariate analysis, and R was used to make a nomogram of the diagnosis and prediction of lymph node metastasis. The nomogram incorporates five items of PD-1, PD-L1, the average distance of CD56 to PD-1, the nomogram incorporates five items of PD-1, PD-L1, the average distance of CD56 to PD-1, the average distance of CD56 to PD-L1, and the average distance of PD-1 to PD-L1. Through internal verification, a new ROC curve was generated and the AUC reached 0.888. Our nomogram can serve as an effective preoperative predictive tool to assess LN status in CC patients.

The cervical biopsy is an essential step before the diagnosis of CC. In the process, we can take part of cancer tissue through the cervical biopsy for mIF detection. HALO system is further applied in quantitative and spatial analysis. By the diagnostic prediction model, we can predict pLN metastasis preoperatively, thus avoiding unnecessary routine pLN dissection. In this way, we can avoid additional surgical trauma and possible complications for patients.

The limitations of our study include external validation for the model. External validation is needed to acquire high-level evidence for clinical application.

## Conclusion

In conclusion, we construct an immune nomogram that incorporates five items of PD-1, PD-L1, the average distance of CD56 to PD-1, the average distance of CD56 to PD-L1, and the average distance of PD-1 to PD-L1, which can be conveniently used to facilitate the preoperative individualized prediction of pLN metastasis in patients with CC.

## Electronic supplementary material

Below is the link to the electronic supplementary material.

Supplementary Material 1

Supplementary Material 2

Supplementary Material 3

## Data Availability

The data that support the results of this study are available from the corresponding author upon reasonable request.
