# Supplementary material for: Developing a nomogram for preoperative prediction of cervical cancer lymph node metastasis by multiplex immunofluorescence
Source: BMC Cancer. 2023 May 30;23:485. doi: 10.1186/s12885-023-10932-0 (PMC10228122; doi:10.1186/s12885-023-10932-0)
Supplement: Supplementary file 1 — Supplementary Material 1 [file 12885_2023_10932_MOESM1_ESM.docx]

1. **R of Heatmap**

#install.packages("pheatmap")

library(pheatmap)

inputFile="input.txt"

groupFile="group.txt"

outFile="heatmap.pdf"

setwd("D:\\biowolf\\bioR\\17.heatmap")

rt=read.table(inputFile,header=T,sep="\t",row.names=1,check.names=F)

ann=read.table(groupFile,header=T,sep="\t",row.names=1,check.names=F)

pdf(file=outFile,width=6,height=5.5)

pheatmap(rt,

annotation=ann,

cluster_cols = T,

color = colorRampPalette(c("blue", "white", "red"))(50),

show_colnames = T,

scale="row",

#border_color ="NA",

fontsize = 8,

fontsize_row=6,

fontsize_col=6)

dev.off()

1. **R of Volcanic map**

#install.packages("ggplot2")

library(ggplot2)

inputFile="input.txt"

outFile="vol.pdf"

logFCfilter=1

fdrFilter=0.05

setwd("D:\\biowolf\\Desktop\\bioR\\19.vol")

rt=read.table(inputFile,sep="\t",header=T,check.names=F)

Significant=ifelse((rt$fdr<fdrFilter & abs(rt$logFC)>logFCfilter), ifelse(rt$logFC>logFCfilter,"Up","Down"), "Not")

p = ggplot(rt, aes(logFC, -log10(fdr)))+

geom_point(aes(col=Significant))+

scale_color_manual(values=c("green", "black", "red"))+

labs(title = " ")+

theme(plot.title = element_text(size = 16, hjust = 0.5, face = "bold"))

p=p+theme_bw()

pdf(outFile,width=5.5,height=4.5)

print(p)

dev.off()

1. **R of nomogram**

#install.packages("rms")

library(rms)

inputFile="input.txt"

setwd("D:\\biowolf\\bioR\\39.Nomo")

rt=read.table(inputFile,header=T,sep="\t",check.names=F,row.names=1)

dd <- datadist(rt)

options(datadist="dd")

f <- cph(Surv(futime, fustat) ~ Age+Gender+Grade+T+M+N+VCAN, x=T, y=T, surv=T, data=rt, time.inc=1)

surv <- Survival(f)

nomogram

nom <- nomogram(f, fun=list(function(x) surv(1, x), function(x) surv(2, x), function(x) surv(3, x)),

lp=F, funlabel=c("1-year survival", "2-year survival", "3-year survival"),

maxscale=100,

fun.at=c(0.99, 0.9, 0.8, 0.7, 0.5, 0.3,0.1,0.01))

#nomogram

pdf(file="Nomogram.pdf",height=7,width=8.5)

plot(nom)

dev.off()

#calibration curve

time=3

f <- cph(Surv(futime, fustat) ~ Age+Gender+Grade+T+M+N+VCAN, x=T, y=T, surv=T, data=rt, time.inc=time)

cal <- calibrate(f, cmethod="KM", method="boot", u=time, m=100, B=1000)

pdf(file="calibration.pdf",height=6,width=7)

plot(cal,xlab="Nomogram-Predicted Probability of 3-Year OS",ylab="Actual 3-Year OS(proportion)",col="red",sub=F)

dev.off()

#install.packages("pROC")

1. **R of ROC**

library(pROC)

inputFile="input.txt"

outFile="ROC.pdf"

x="TTYH3"

setwd("D:\\biowolf\\bioR\\40.ROC")

rt=read.table(inputFile,header=T,sep="\t",check.names=F,row.names=1)

y=colnames(rt)[1]

rocobj1=roc(rt[,y], as.vector(rt[,x]))

pdf(file=outFile,width=5,height=5)

plot(rocobj1, print.auc=TRUE, col="red")

dev.off()
