# Supplementary material for: Developing a nomogram for preoperative prediction of cervical cancer lymph node metastasis by multiplex immunofluorescence
Source: BMC Cancer. 2023 May 30;23:485. doi: 10.1186/s12885-023-10932-0 (PMC10228122; doi:10.1186/s12885-023-10932-0)
Supplement: Supplementary file 2 — Supplementary Material 2 [file 12885_2023_10932_MOESM2_ESM.docx]

**Supplementary Table S1. The differential immune cells of cervical cancer (CC) with positive lymph node (LN) and CC with negative LN were analyzed by TCGA database.**

| Immune Cells   \| NK Cells Resting \|  \| \| --- \| --- \| \| Macrophages M2 \|  \| \| T Cells Follicular Helper \|  \| \| T Cells CD4 Memory Resting \|  \| \| Macrophages \|  \| \| Dendritic Cells Resting \|  \| \| Lymphocytes \|  \| \| Mast Cells Resting \|  \| \| T Cells CD4 Naive \|  \| \| T Cells CD4 Memory Activated \|  \| \| T Cells CD8 \|  \| \| Mast Cells Activated \|  \| \| Dendritic Cells Activated \|  \| \| Macrophages M0 \|  \| \| T Cells Regulatory Tregs \|  \| \| Plasma Cells \|  \| \| Monocytes \|  \| \| Neutrophils \|  \| \| Eosinophils \|  \| \| B Cells Naive \|  \| \| Dendritic Cells \|  \| \| B Cells Memory \|  \| \| T Cells gamma delta \|  \| \| Mast Cells \|  \| \| NK Cells Activated \|  \| \| Macrophages M1 \|  \| | *p*   \| 0.018541905 \| \| --- \| \| 0.047824583 \| \| 0.068322478 \| \| 0.080677069 \| \| 0.090194369 \| \| 0.09994947 \| \| 0.128978153 \| \| 0.143653743 \| \| 0.161284212 \| \| 0.177972695 \| \|  \| \| 0.186258009 \| \| 0.189166657 \| \| 0.190740752 \| \| 0.209207292 \| \| 0.263179585 \| \| 0.308225953 \| \| 0.459766988 \| \| 0.545253788 \| \| 0.545491581 \| \| 0.610608146 \| \| 0.682244064 \| \| 0.749376379 \| \| 0.784304749 \| \| 0.787269281 \| \| 0.849883239 \| \| 0.861498785 \| | FC   \| 2.192855302 \| \| --- \| \| 0.842309742 \| \| 1.162871985 \| \| 0.757890993 \| \| 0.864395489 \| \| 0.685821565 \| \| 1.057408527 \| \| 0.754401153 \| \| inf \| \| 2.335029839 \| \| 1.10527558 \| \| 1.773782423 \| \| 1.277881961 \| \| 0.744863256 \| \| 0.840718369 \| \| 0.937842547 \| \| 1.034213574 \| \| 0.687859301 \| \| 0.945682743 \| \| 1.027946402 \| \| 0.957811741 \| \| 1.041088781 \| \| 7.27846557 \| \| 1.041220345 \| \| 1.05215515 \| \| 0.991220603 \| |
| --- | --- | --- | --- | --- | --- | --- | --- | --- | --- | --- | --- | --- | --- | --- | --- | --- | --- | --- | --- | --- | --- | --- | --- | --- | --- | --- | --- | --- | --- | --- | --- | --- | --- | --- | --- | --- | --- | --- | --- | --- | --- | --- | --- | --- | --- | --- | --- | --- | --- | --- | --- | --- | --- | --- | --- | --- | --- | --- | --- | --- | --- | --- | --- | --- | --- | --- | --- | --- | --- | --- | --- | --- | --- | --- | --- | --- | --- | --- | --- | --- | --- | --- | --- | --- | --- | --- | --- | --- | --- | --- | --- | --- | --- | --- | --- | --- | --- | --- | --- | --- | --- | --- | --- | --- | --- | --- | --- |
