# Supplementary material for: Developing a nomogram for preoperative prediction of cervical cancer lymph node metastasis by multiplex immunofluorescence
Source: BMC Cancer. 2023 May 30;23:485. doi: 10.1186/s12885-023-10932-0 (PMC10228122; doi:10.1186/s12885-023-10932-0)
Supplement: Supplementary file 3 — Supplementary Material 3 [file 12885_2023_10932_MOESM3_ESM.docx]

**Supplementary Table S2. The differential immune genes of cervical cancer (CC) with positive lymph node (LN) and CC with negative LN were analyzed by TCGA database.**

| gene_name | p | fc |
| --- | --- | --- |
| RP11-1267H10.4 | 0.038905389 | 198.100354 |
| SP3P | 0.082192181 | 79.03178019 |
| RP11-308B16.2 | 0.083426137 | 68.8459413 |
| AC023347.1 | 0.044214578 | 65.55043933 |
| AC106875.1 | 0.109890045 | 57.85977052 |
| RP11-474D1.3 | 0.011095997 | 55.28797398 |
| ARF4 | 4.16E-06 | 1.322489709 |
| CASP6 | 1.10E-05 | 1.351951899 |
| LRRC49 | 1.54E-05 | 1.627310559 |
| TMF1 | 1.90E-05 | 1.38098221 |
| RP11-474D1.4 | 0.076182586 | 40.42276496 |
| RP11-317N12.1 | 0.07962073 | 38.19955659 |
| CCDC66 | 1.91E-05 | 1.330546187 |
| CADPS2 | 2.65E-05 | 1.618778201 |
| RP11-369E15.3 | 0.147584159 | 33.4875875 |
| LINC00523 | 0.124479662 | 31.59282753 |
| CTSO | 3.91E-05 | 1.453348933 |
| RP11-618I10.2 | 0.143727435 | 31.10667964 |
| AC113607.3 | 0.144737931 | 31.08358975 |
| RP11-464C19.3 | 0.037072749 | 28.92366213 |
| RP11-328J2.1 | 0.12978893 | 27.45828405 |
| AC000111.4 | 0.002259215 | 27.11752137 |
| LINC01571 | 0.041652114 | 27.06085641 |
| AC008271.1 | 0.098768283 | 26.61948768 |
| RP11-319E12.1 | 0.037734167 | 25.31704813 |
| DNAJC1 | 3.95E-05 | 1.282110242 |
| CDADC1 | 4.86E-05 | 1.266683038 |
| RP11-468N14.7 | 0.126317241 | 21.89026707 |
| RP11-513O17.3 | 0.051174754 | 21.0249796 |
| KCTD6 | 5.45E-05 | 1.50027576 |
| CH17-125A10.2 | 0.032546224 | 19.13435069 |
| TMED7 | 5.79E-05 | 1.249870304 |
| LINC01231 | 0.125227011 | 18.62391442 |
| AC016821.2 | 0.052008902 | 18.39897434 |
| SGCB | 6.19E-05 | 1.513002642 |
| RP11-369E15.4 | 0.048284739 | 18.07703605 |
| RP11-713P17.5 | 0.023330889 | 17.9961983 |
| LINC00473 | 0.020126584 | 17.80207252 |
| LINC00602 | 0.003054322 | 17.31132568 |
| RN7SL250P | 0.014808148 | 17.12235713 |
| RP11-473L15.3 | 0.145800739 | 17.00844419 |
| AC012506.2 | 0.113179153 | 16.96522414 |
| HMGB3 | 6.52E-05 | 1.487478147 |
| RP11-3G21.1 | 0.126331343 | 16.69726355 |
| RP11-127O4.3 | 0.086601413 | 16.67428929 |
| DDX11L5 | 0.071387338 | 16.60931841 |
| AC018685.2 | 0.085038879 | 16.53760961 |
| FAAH2 | 7.87E-05 | 1.540366368 |
| LZTFL1 | 8.60E-05 | 1.430585171 |
| SEMA3B | 9.93E-05 | 2.935862977 |
| CTC-295J13.3 | 0.081149934 | 16.14287052 |
| CMC1 | 0.000108091 | 1.244950964 |
| RP11-364B14.3 | 0.060076779 | 15.30788516 |
| RP11-468N14.6 | 0.036888427 | 15.27275574 |
| RPS24P14 | 0.000340815 | 15.15855351 |
| CMTM6 | 0.000108245 | 1.394822149 |
| JAKMIP2-AS1 | 0.052869102 | 15.091932 |
| AP000477.2 | 0.049512405 | 14.92443291 |
| RP11-279O17.1 | 0.128635411 | 14.84256386 |
| RP11-642E20.2 | 0.020164253 | 14.3365629 |
| LINC00434 | 0.057810841 | 14.17509079 |
| RP5-1185K9.1 | 0.092922594 | 13.83526943 |
| ACSL4 | 0.000122818 | 1.613212211 |
| TGIF1 | 0.000127902 | 1.289114195 |
| PLEKHH1 | 0.000130113 | 1.764858427 |
| CTD-2022H16.2 | 0.003068181 | 13.39782832 |
| AC025016.1 | 0.126667789 | 13.29334166 |
| MSRA | 0.000139601 | 1.359219214 |
| MIR4478 | 0.048846618 | 13.26625754 |
| RP11-488I20.9 | 0.124776549 | 13.24544292 |
| FAM107B | 0.000139639 | 1.671311092 |
| RP11-655M14.4 | 0.026946373 | 13.07636233 |
| BDH2 | 0.000157122 | 1.413214254 |
| ALG10B | 0.000157777 | 1.317214704 |
| PTP4A1 | 0.000160837 | 1.658729404 |
| RHOBTB3 | 0.000164002 | 2.275393911 |
| AF003625.3 | 0.05182755 | 12.75262585 |
| RP11-140I24.2 | 0.004534012 | 12.74490728 |
| RP11-200K12.2 | 0.001172889 | 12.62898272 |
| GLT8D1 | 0.000169372 | 1.247599145 |
| TMBIM4 | 0.000172125 | 1.240414167 |
| RP4-745K6.1 | 0.018771942 | 12.36765677 |
| RP11-184E9.2 | 0.096467616 | 12.34941813 |
| CARM1P1 | 0.077864863 | 12.32702041 |
| RP11-85G21.2 | 0.109311639 | 12.27615512 |
| PPP4R2 | 0.000173683 | 1.257327461 |
| HSD3BP2 | 0.020148474 | 12.01445596 |
| ZNF844 | 0.000174515 | 1.939635672 |
| KCNH3 | 0.000175967 | 4.04335851 |
| CPO | 0.00017605 | 2.131946099 |
| RP11-352D3.2 | 0.037698215 | 11.66647866 |
| LINC00261 | 0.016165211 | 11.63180385 |
| RP11-550P17.5 | 0.055461375 | 11.53711646 |
| SAT1 | 0.000177402 | 1.455947737 |
| STX18 | 0.000178225 | 1.375085433 |
| KIAA1456 | 0.000210558 | 2.089843206 |
| BMPR1B-AS1 | 0.018572038 | 11.31872577 |
| RFK | 0.000219816 | 1.517190493 |
| DYDC2 | 0.000226498 | 3.401439767 |
| RP11-401P9.1 | 0.036637351 | 11.17460837 |
| NAV2-AS5 | 0.034653087 | 11.1713336 |
| PHC2 | 0.000246641 | 1.287789735 |
| U8 | 0.067685259 | 11.0482941 |
| RHOA | 0.000257 | 1.1968965 |
| RP11-293E1.1 | 0.01255165 | 10.98236638 |
| RNA5SP67 | 0.002561421 | 10.96013413 |
| CRYGEP | 0.125120514 | 10.87109008 |
| LINC00566 | 0.002295268 | 10.85091761 |
| AC007277.3 | 0.135267056 | 10.7876054 |
| DENND6A | 0.000259922 | 1.251057628 |
| RP11-12M5.3 | 0.058741613 | 10.6627735 |
| RP11-442N1.1 | 0.070057597 | 10.59003167 |
| RNU6-178P | 0.003233738 | 10.56081952 |
| RP11-143E21.1 | 0.064139997 | 10.53620653 |
| RP11-69C17.3 | 0.10007827 | 10.44955003 |
| AC004702.2 | 0.002216757 | 10.33924735 |
| AC010329.1 | 0.02322788 | 10.31560471 |
| POM121L3P | 0.020319862 | 10.29798174 |
| RP1-41P2.7 | 0.010376477 | 10.28392748 |
| RP11-218F4.2 | 0.000860276 | 10.25444762 |
| RP1-90L14.1 | 0.034439127 | 10.24878244 |
| RP11-763K15.1 | 0.101534772 | 10.23788638 |
| ENPP5 | 0.000268481 | 2.254366932 |
| CTD-2008P7.8 | 0.079164224 | 10.22423842 |
| RP11-687M24.8 | 0.042479003 | 10.22357663 |
| RP11-403I13.5 | 0.00155785 | 10.17634741 |
| RP11-523J2.1 | 0.00344027 | 10.10274394 |
| RP11-655G22.1 | 0.143066683 | 10.01889976 |
| CTB-174D11.1 | 0.137302311 | 9.920865438 |
| RP3-331H24.4 | 0.004493611 | 9.910433285 |
| TRIM8 | 0.000270442 | 1.252077483 |
| AC011525.2 | 0.150530984 | 9.789731315 |
| CTB-10G5.1 | 0.006740903 | 9.736485241 |
| AL109763.2 | 0.063376868 | 9.696796855 |
| UGGT2 | 0.000281617 | 1.316534602 |
| B4GALT1 | 0.000286533 | 1.455199351 |
| AC103996.1 | 0.078391735 | 9.626365642 |
| DUSP6 | 0.000297781 | 2.042211 |
| CCDC149 | 0.000347463 | 1.449235388 |
| SPEF2 | 0.000374543 | 2.313097492 |
| AC098872.3 | 0.013874324 | 9.455925521 |
| RP13-895J2.8 | 0.094088757 | 9.387641645 |
| POC1B | 0.00037559 | 1.342469873 |
| OR8G3P | 0.145061175 | 9.333780189 |
| MTHFD2L | 0.000381259 | 1.402315902 |
| AL109763.1 | 0.149928751 | 9.278454373 |
| CTD-2311B13.9 | 0.136884018 | 9.246110631 |
| IRAK3 | 0.000388909 | 1.787133636 |
| MAPK8 | 0.000414357 | 1.212720249 |
| SLC7A2 | 0.000418672 | 4.826310041 |
| COX18 | 0.000425419 | 1.282695786 |
| GAPDHP19 | 0.00256248 | 9.154618172 |
| UQCRBP3 | 0.000895381 | 9.152653088 |
| RP11-482E14.2 | 0.019357326 | 9.122720773 |
| LINC01411 | 0.09388385 | 9.069173988 |
| RNU1-65P | 0.100453606 | 8.981125646 |
| AC079586.1 | 0.14874995 | 8.972086741 |
| AC012075.2 | 0.026322077 | 8.934676409 |
| ROR2 | 0.000427801 | 1.813462932 |
| RP11-351O1.3 | 0.001721047 | 8.90431547 |
| RP11-39M21.2 | 0.001073321 | 8.841553988 |
| TMEM212-AS1 | 0.016518359 | 8.806640918 |
| RP11-164O23.5 | 0.008470346 | 8.785568585 |
| ERVH48-1 | 0.082914704 | 8.782177527 |
| AE000658.30 | 0.120056896 | 8.733596413 |
| RP1-283K11.3 | 0.053880913 | 8.733304353 |
| LINC01241 | 0.0912207 | 8.718603434 |
| RP11-762N20.1 | 0.07154197 | 8.702864261 |
| RP11-386M24.8 | 0.028704486 | 8.690549889 |
| LINC00355 | 0.07558741 | 8.605006925 |
| RNA5SP79 | 0.034553807 | 8.600407259 |
| RNU6-1208P | 0.006183641 | 8.59491291 |
| RP11-46I8.4 | 0.001944138 | 8.589089694 |
| GLB1 | 0.000444954 | 1.359752068 |
| ECEL1P1 | 0.136258669 | 8.545028611 |
| RP11-214N1.1 | 0.103476794 | 8.544607037 |
| UFL1 | 0.000465402 | 1.255959502 |
| RP11-661D19.3 | 0.040070357 | 8.476766408 |
| MOB1B | 0.000470805 | 1.247146088 |
| AC091736.1 | 0.116098028 | 8.432096605 |
| AC104809.4 | 0.011790985 | 8.386843254 |
| RP11-495K9.6 | 0.033644266 | 8.364295555 |
| CFAP57 | 0.000471669 | 2.328002708 |
| CBX5 | 0.000474769 | 1.293214835 |
| PCAT4 | 0.039259594 | 8.335055128 |
| C15orf65 | 0.00048147 | 1.522618252 |
| RP11-465O11.2 | 0.043474384 | 8.306924627 |
| RP11-396O20.2 | 0.045417604 | 8.195649461 |
| FSD1L | 0.0004859 | 1.550117629 |
| AC012065.4 | 0.019888953 | 8.102831863 |
| MED28 | 0.000487444 | 1.212341272 |
| C15orf32 | 0.076119652 | 8.022542846 |
| KLHL29 | 0.000493324 | 1.84566442 |
| FIGN | 0.000515402 | 2.230814139 |
| AC011298.2 | 0.0268901 | 7.958416497 |
| RP11-79C6.2 | 0.00608713 | 7.955028398 |
| LINC00376 | 0.150344706 | 7.954756715 |
| RP11-369C8.1 | 0.093417847 | 7.945431896 |
| RP11-301G19.1 | 0.08938563 | 7.943160058 |
| CTC-430J12.2 | 0.021304342 | 7.905053307 |
| RP11-315A17.1 | 0.137041949 | 7.898097704 |
| SDAD1P4 | 0.019604155 | 7.866722651 |
| RP11-195B3.1 | 0.138188379 | 7.853203282 |
| ECEL1P2 | 0.01362047 | 7.850799362 |
| RN7SL586P | 0.015988505 | 7.845162363 |
| COL11A2P1 | 0.117086232 | 7.839622354 |
| RP1-240B8.3 | 0.089567834 | 7.827514175 |
| RP11-152C17.1 | 0.042649905 | 7.824215992 |
| NDP-AS1 | 0.011044869 | 7.766039683 |
| RP11-76I14.1 | 0.114460103 | 7.751971383 |
| RPP14 | 0.000529722 | 1.198050954 |
| snoU13 | 0.00412083 | 7.736404721 |
| RP1-241P17.1 | 0.064095009 | 7.731348653 |
| TMEM263 | 0.000538597 | 1.413259573 |
| RP11-645N11.3 | 0.001404395 | 7.700084433 |
| RNA5SP168 | 0.03623268 | 7.69444834 |
| PRSS41 | 0.115862922 | 7.693878767 |
| RP11-1166P10.6 | 0.106894074 | 7.692515216 |
| MED7 | 0.000546992 | 1.1783765 |
| RNU6ATAC29P | 0.024097714 | 7.67985288 |
| SLC4A4 | 0.000549847 | 4.611422478 |
| RP11-124G5.3 | 0.112405398 | 7.663642086 |
| RP11-476I15.6 | 0.017045047 | 7.662570782 |
| RP11-542F9.1 | 0.113118247 | 7.651056781 |
| RP11-638F5.2 | 0.003935289 | 7.650925043 |
| CTD-2258A20.3 | 0.01133801 | 7.648223761 |
| TESC-AS1 | 0.014223036 | 7.616423818 |
| RP11-91I8.2 | 0.061842488 | 7.589536151 |
| NICN1 | 0.000575215 | 1.327172764 |
| MAP1LC3P | 0.05068653 | 7.559245761 |
| SNORA63 | 0.057113584 | 7.556718043 |
| RP3-364H10.1 | 0.111954973 | 7.538301907 |
| RP11-666A1.4 | 0.023399314 | 7.537959825 |
| Y_RNA | 0.016643881 | 7.501595986 |
| OVAAL | 0.016478577 | 7.474807558 |
| ST13P16 | 0.007089859 | 7.448515897 |
| DECR1 | 0.000580904 | 1.253764577 |
| PDHB | 0.000601227 | 1.223846509 |
| RPL31P59 | 0.031447259 | 7.397492261 |
| CTA-796E4.4 | 0.121335032 | 7.39026922 |
| RP11-542M13.1 | 0.06196928 | 7.364392131 |
| RP11-443K8.2 | 0.102914802 | 7.328977335 |
| TSNAX-DISC1 | 0.000610126 | 2.276085656 |
| RP11-692C24.1 | 0.068034407 | 7.295164005 |
| RP11-115J16.2 | 0.095916215 | 7.291832134 |
| RP11-349F21.5 | 0.00036537 | 7.284541625 |
| AC105402.3 | 0.024757471 | 7.278640983 |
| MRGPRG-AS1 | 0.078903819 | 7.265458588 |
| pRNA | 0.027377933 | 7.243864856 |
| AC011523.2 | 0.028851497 | 7.243451524 |
| FAM92A1 | 0.000617681 | 1.370424537 |
| KSR1P1 | 0.002327356 | 7.241861953 |
| TEAD1 | 0.000657906 | 1.325364411 |
| RP5-1021I20.2 | 0.046128885 | 7.199629179 |
| EIF1B | 0.000683286 | 1.231872892 |
| HIST1H1PS2 | 0.016080103 | 7.182348297 |
| AP000997.2 | 0.04713121 | 7.160297008 |
| RP11-108H9.1 | 0.049985232 | 7.135648438 |
| SSXP3 | 0.061766498 | 7.131352253 |
| CTD-2210P24.6 | 0.050499022 | 7.104925952 |
| RP11-114H24.7 | 0.024860417 | 7.089785376 |
| RP11-424M24.5 | 0.023702366 | 7.086749822 |
| CCDC174 | 0.000699427 | 1.183255589 |
| IGKV2-14 | 0.139043981 | 7.058261544 |
| OR51K1P | 0.024127051 | 7.048600141 |
| RP11-108N13.1 | 0.036142208 | 7.040133 |
| KCNS1 | 0.000705467 | 0.526249317 |
| ARSK | 0.000714327 | 1.264618158 |
| RP11-315E17.1 | 0.016078809 | 7.006235749 |
| RP11-51C14.1 | 0.136179131 | 6.97971142 |
| B4GALNT2P1 | 0.084710869 | 6.976506541 |
| RP11-775L16.1 | 0.147625557 | 6.967398518 |
| RP11-3N13.2 | 0.106361783 | 6.957773166 |
| UPRT | 0.000729014 | 1.466167382 |
| CTC-465D4.1 | 0.013822049 | 6.955591065 |
| AC104383.1 | 0.116552722 | 6.954779731 |
| RP5-978I12.1 | 0.100908077 | 6.953852891 |
| CTD-2001E22.1 | 0.022350988 | 6.928960356 |
| CCNB1IP1P2 | 0.008393522 | 6.905802071 |
| SPCS1 | 0.000735372 | 1.210705413 |
| LINC01222 | 0.014230965 | 6.900514953 |
| HYAL2 | 0.000750791 | 1.226970565 |
| DLGAP1-AS5 | 0.099044993 | 6.879693084 |
| LPIN2 | 0.000761885 | 1.657393684 |
| FAM83C | 0.000767365 | 0.462606245 |
| SACM1L | 0.000772532 | 1.22007787 |
| RP13-653N12.1 | 0.043773228 | 6.790562068 |
| IFT81 | 0.000786383 | 1.388394491 |
| SLC35B2 | 0.000798758 | 1.283151153 |
| RP11-100K18.1 | 0.046392978 | 6.751048341 |
| CFAP69 | 0.000804671 | 1.799837819 |
| RP11-615I16.1 | 0.017737675 | 6.711727293 |
| RP11-379P1.4 | 0.045728506 | 6.704572791 |
| CTA-31J9.2 | 0.016176964 | 6.686423559 |
| AC123023.1 | 0.038273517 | 6.674135285 |
| RP4-675G8.3 | 0.052796893 | 6.65772147 |
| RP11-666O2.1 | 0.002330595 | 6.651577121 |
| RNU6-267P | 0.010768841 | 6.645237109 |
| AC008694.3 | 0.060633261 | 6.636530629 |
| RP11-252P19.1 | 0.004489581 | 6.627498291 |
| ZNF878 | 0.000823994 | 2.03976043 |
| CTB-23I7.1 | 0.043839519 | 6.580864949 |
| LDHAL6EP | 0.010811251 | 6.573517526 |
| NEK4 | 0.000827441 | 1.340436362 |
| RP11-72H11.1 | 0.027209896 | 6.564480176 |
| AC122714.1 | 0.089024165 | 6.559416295 |
| TCAIM | 0.000840243 | 1.288990143 |
| RPL21P109 | 0.014193152 | 6.545827635 |
| RP11-372H2.1 | 0.009949372 | 6.535667622 |
| RNU6-128P | 0.027929928 | 6.526746013 |
| RAB28 | 0.000848095 | 1.212510956 |
| AC004692.4 | 0.033729922 | 6.501573248 |
| PARVA | 0.000853276 | 1.34427424 |
| RP11-296L22.8 | 0.004006243 | 6.476830231 |
| RP11-861L17.3 | 6.11E-05 | 6.474532771 |
| AC068134.8 | 0.002564984 | 6.448216827 |
| AC004878.2 | 0.030121264 | 6.439059358 |
| KPTN | 0.000891665 | 0.762702475 |
| RP11-510M2.4 | 0.143302261 | 6.428028682 |
| RP5-945I17.2 | 0.023621144 | 6.398590086 |
| RP11-567N4.2 | 0.063172735 | 6.376191691 |
| LINC01101 | 0.038947538 | 6.368886213 |
| C11orf97 | 0.01534589 | 6.365467556 |
| RP11-85D18.1 | 0.034024744 | 6.355288188 |
| RP11-573M3.6 | 0.013341582 | 6.350596819 |
| BANCR | 0.149388654 | 6.315691039 |
| AC018643.4 | 0.083502383 | 6.288723359 |
| RP11-364P22.1 | 0.008243875 | 6.287812983 |
| RN7SKP232 | 0.056629116 | 6.286470663 |
| LINC01443 | 0.03949836 | 6.275521412 |
| TRBV22-1 | 0.137734298 | 6.269064162 |
| RAB3IP | 0.000892374 | 1.302598802 |
| RP11-103J17.2 | 0.080292452 | 6.242954755 |
| CLUH | 0.000906259 | 0.799525801 |
| OR2L6P | 0.005631219 | 6.242073193 |
| RP11-616M22.2 | 0.039165757 | 6.241641977 |
| CTD-2544H17.1 | 0.020825869 | 6.233021643 |
| AC011752.1 | 0.082416788 | 6.227579438 |
| RP11-541M12.3 | 0.032844689 | 6.219153122 |
| PDE4D | 0.000915707 | 1.746349404 |
| RP11-116O18.3 | 0.130349369 | 6.2107271 |
| CYCSP28 | 0.004334521 | 6.20507273 |
| MIR6894 | 0.014311977 | 6.200323663 |
| ID2 | 0.000935616 | 1.513951316 |
| uc_338 | 0.099148333 | 6.194720803 |
| CFI | 0.000940747 | 1.918087659 |
| C10orf11 | 0.000942279 | 1.532803905 |
| TMLHE | 0.000961341 | 1.416135841 |
| EXOG | 0.000964776 | 1.278738781 |
| AC005518.2 | 0.106875548 | 6.140929789 |
| BUB1P1 | 0.04136823 | 6.139084108 |
| RP3-407E4.4 | 0.051960351 | 6.104270908 |
| RP11-95H11.1 | 0.000465517 | 6.103983683 |
| RNU6-986P | 0.002987552 | 6.102419214 |
| NTN4 | 0.001010391 | 1.961988256 |
| RP5-1178H5.2 | 0.046833125 | 6.09092952 |
| SNX25 | 0.00101577 | 1.311096522 |
| AC013275.2 | 0.128227485 | 6.088724101 |
| RP11-59N23.3 | 0.090435479 | 6.083628411 |
| RP11-335O4.1 | 0.105313851 | 6.077513691 |
| VN1R76P | 0.099050283 | 6.075275971 |
| SNORA31 | 0.038562036 | 6.073115492 |
| RP11-354I13.1 | 0.083872717 | 6.066612397 |
| TUBE1 | 0.001020796 | 1.249567639 |
| RP11-117D22.1 | 0.048804531 | 6.041848383 |
| RN7SL152P | 0.020894606 | 6.015365519 |
| RNU6-1086P | 0.055213903 | 6.003483469 |
| AC092687.4 | 0.140053858 | 5.999362856 |
| RP11-284A20.1 | 0.067786419 | 5.994576882 |
| RASSF6 | 0.00103022 | 1.886792879 |
| MIR1226 | 0.045308932 | 5.974223009 |
| RP11-318G21.4 | 0.094696321 | 5.967903167 |
| Y_RNA | 0.012452723 | 5.962797845 |
| RP1-258N20.3 | 0.088607294 | 5.952024535 |
| RP1-72E17.2 | 0.022092953 | 5.951557439 |
| OR10AA1P | 0.041129942 | 5.947987628 |
| PTP4A2 | 0.001062732 | 1.194622198 |
| DNAJC22 | 0.001072379 | 2.962371145 |
| BX842568.1 | 0.003210605 | 5.921420482 |
| PPY2 | 0.118089324 | 5.918223865 |
| RP11-510M2.6 | 0.004218135 | 5.913442013 |
| SNORA51 | 0.052644368 | 5.90970724 |
| FAM122C | 0.001087864 | 1.328174662 |
| GORASP1 | 0.001089829 | 1.232974829 |
| RP11-76N22.1 | 0.000403921 | 5.879118715 |
| CTD-2377D24.4 | 0.072035403 | 5.879032935 |
| AC013399.3 | 0.006664475 | 5.877513562 |
| NEDD9 | 0.001093714 | 1.831495862 |
| RP3-366N23.4 | 0.074080643 | 5.858593836 |
| NANOGP10 | 0.094429829 | 5.855990539 |
| AL354720.1 | 0.029947213 | 5.84513888 |
| LINC01312 | 0.035835682 | 5.833490289 |
| PDE12 | 0.001120015 | 1.197872792 |
| AC093698.4 | 0.129604239 | 5.827376505 |
| NHLRC3 | 0.001128656 | 1.318168919 |
| RP11-168K9.2 | 0.009282648 | 5.80909915 |
| CCNI | 0.001141535 | 1.187045264 |
| RP11-269F21.2 | 0.106260303 | 5.799446709 |
| RP11-293B20.2 | 0.012525673 | 5.796977532 |
| AC104654.1 | 0.036708634 | 5.791210239 |
| MORC2 | 0.00114478 | 0.819450376 |
| RP11-268F1.3 | 0.090714955 | 5.78518136 |
| RP11-498M14.1 | 0.026373096 | 5.773982985 |
| AC114814.4 | 0.056399636 | 5.767461198 |
| RP11-30K9.7 | 0.038251694 | 5.760877357 |
| RP11-65L19.4 | 0.050122487 | 5.748025598 |
| RP11-384J4.2 | 0.07826991 | 5.732451362 |
| RP11-299A16.1 | 0.122178264 | 5.721932418 |
| BOK-AS1 | 0.075170721 | 5.714858331 |
| PIWIL1 | 0.001145956 | 4.330429503 |
| RP11-260M19.2 | 0.118732881 | 5.695193518 |
| RP11-248E9.6 | 0.02780259 | 5.695187802 |
| RP11-401P9.4 | 0.127623929 | 5.692390354 |
| FOXN3-AS2 | 0.018362537 | 5.687231398 |
| AC007563.3 | 0.061370654 | 5.680829413 |
| TMSB15A | 0.001149861 | 2.239837465 |
| MIR606 | 0.02527199 | 5.668249033 |
| SLC30A1 | 0.001151139 | 1.359741488 |
| AC080094.1 | 0.03834635 | 5.630632937 |
| RP11-406A20.4 | 0.009712926 | 5.624634261 |
| RP11-85B7.4 | 0.008914573 | 5.608872608 |
| CTC-493P15.2 | 0.04695281 | 5.605163737 |
| GLRXP | 0.055158571 | 5.602761014 |
| SNORA25 | 0.020905443 | 5.600579803 |
| SENP8 | 0.001175356 | 1.246636576 |
| RP11-507B12.2 | 0.140812718 | 5.599472892 |
| AC064847.4 | 0.147747842 | 5.597210322 |
| RP11-132E11.2 | 0.046215956 | 5.590125011 |
| UBA3 | 0.00122879 | 1.197472613 |
| DIAPH3 | 0.001234941 | 1.352042397 |
| CGNL1 | 0.00124315 | 1.884884418 |
| AL136123.1 | 0.013124505 | 5.561437002 |
| TMEM8A | 0.001266361 | 1.387866017 |
| AC108448.3 | 0.053080939 | 5.557272838 |
| RP11-388E23.2 | 0.135116419 | 5.548428327 |
| RP11-243M5.5 | 0.071137652 | 5.534370825 |
| NXF4 | 0.034159832 | 5.530321032 |
| WDR19 | 0.001267296 | 1.35577618 |
| RN7SKP118 | 0.039948168 | 5.511048466 |
| RP11-490O24.2 | 0.040080655 | 5.504959616 |
| RP11-64K10.1 | 0.02434801 | 5.486237119 |
| LINC00351 | 0.101824857 | 5.480671795 |
| ZNF317P1 | 0.011473977 | 5.470688518 |
| LINC00488 | 0.124251766 | 5.468041566 |
| RP11-527H14.1 | 0.07649879 | 5.464649342 |
| CTD-2314B22.1 | 0.137358232 | 5.45715064 |
| AC018359.1 | 0.082618281 | 5.456086308 |
| RP1-73A14.1 | 0.031667767 | 5.453044668 |
| MIR610 | 0.026716556 | 5.443945744 |
| RP11-466P24.6 | 0.098497832 | 5.443778769 |
| CASC22 | 0.119153065 | 5.441641409 |
| CTD-3187F8.11 | 0.088422164 | 5.434550154 |
| RP11-149I23.3 | 0.108579652 | 5.43296135 |
| ARPIN | 0.001302208 | 1.289470999 |
| RP11-347J14.7 | 0.031441255 | 5.417445635 |
| CTC-431G16.3 | 0.042796822 | 5.411025983 |
| CTD-2307P3.1 | 0.037869167 | 5.408703975 |
| RP11-335O13.8 | 0.10040697 | 5.403777155 |
| RP11-554K11.2 | 0.100166212 | 5.400211131 |
| NDUFS5P1 | 0.070518427 | 5.392864213 |
| RP11-400N13.2 | 0.012682077 | 5.391560179 |
| RP11-780M14.1 | 0.003835525 | 5.38562492 |
| AC011995.1 | 0.002237309 | 5.380536126 |
| AC024082.3 | 0.102406349 | 5.371900646 |
| CTD-2582M21.1 | 0.009698838 | 5.35984732 |
| RP11-412H8.2 | 0.030512174 | 5.351284562 |
| RP11-453N3.7 | 0.0556588 | 5.344317897 |
| RP11-512N21.1 | 0.065956314 | 5.341920133 |
| RN7SL456P | 0.034616935 | 5.34120115 |
| AC008834.1 | 0.119063425 | 5.340667613 |
| RP11-756D7.2 | 0.037587885 | 5.318988605 |
| RP5-1028K7.1 | 0.044779827 | 5.295428328 |
| AC005235.1 | 0.107226136 | 5.292186601 |
| RP11-405A12.2 | 0.03842182 | 5.289137621 |
| CTB-78F1.2 | 0.086042902 | 5.279413056 |
| RP11-789C17.3 | 0.004787805 | 5.279254077 |
| ACTR8 | 0.001331435 | 1.185331451 |
| UBXN10 | 0.001340573 | 2.74330091 |
| MICB | 0.001349514 | 1.537706149 |
| RP11-289F5.1 | 0.036787322 | 5.268761221 |
| RP11-136I14.5 | 0.052395623 | 5.262223774 |
| MIR519A1 | 0.082250543 | 5.252314326 |
| SRGAP3-AS2 | 0.139200434 | 5.247183225 |
| FAM47E | 0.001354686 | 2.40128888 |
| RP11-662M24.1 | 0.136918821 | 5.244333945 |
| RP11-245J24.1 | 0.097221308 | 5.230477564 |
| RP11-37C7.2 | 0.03644681 | 5.230209311 |
| RNU6-952P | 0.069499425 | 5.223410202 |
| AP000897.1 | 0.098096315 | 5.212321074 |
| RP11-536N17.1 | 0.029771159 | 5.211459457 |
| PSMD6 | 0.00136613 | 1.179919218 |
| OVCH1-AS1 | 0.085833477 | 5.205539662 |
| RP4-792G4.3 | 0.079921921 | 5.203330504 |
| RP4-717M23.2 | 0.052998893 | 5.202156782 |
| OR7E87P | 0.022136519 | 5.197294599 |
| RBM43P1 | 0.01213708 | 5.194871512 |
| AL358354.1 | 0.08047026 | 5.194613153 |
| Y_RNA | 0.095902746 | 5.192169575 |
| NUP62CL | 0.001375342 | 1.427690614 |
| OSBPL10 | 0.001397995 | 1.493596639 |
| ING1 | 0.001407242 | 1.232831817 |
| C10orf71-AS1 | 0.068525136 | 5.16055134 |
| RP11-551G24.3 | 0.064541992 | 5.158544759 |
| RP11-60L3.6 | 0.044409495 | 5.152949388 |
| TIGD2 | 0.001412509 | 1.279035064 |
| ZBED8 | 0.001425805 | 1.300542861 |
| RP11-554D13.1 | 0.067629469 | 5.139226841 |
| MIR4484 | 0.02933093 | 5.130692284 |
| RP3-407E4.2 | 0.071197955 | 5.129592535 |
| ACSL5 | 0.001441993 | 1.507149212 |
| RP4-736H5.3 | 0.058995493 | 5.114956488 |
| SMOC1 | 0.00145651 | 7.724405297 |
| RP11-542E6.3 | 0.096069887 | 5.111883399 |
| AK9 | 0.001460792 | 1.421112 |
| RP11-457K10.1 | 0.038542653 | 5.098421254 |
| TULP3P1 | 0.100851826 | 5.087122098 |
| EIF6 | 0.001470799 | 0.778415574 |
| LINC01496 | 0.087393017 | 5.077291536 |
| EFCAB1 | 0.001481449 | 3.555422869 |
| SNORA74 | 0.012884769 | 5.074155968 |
| RP11-215C7.3 | 0.027305249 | 5.061158232 |
| MESDC2 | 0.001491092 | 1.158351511 |
| RP11-280O1.2 | 0.064261913 | 5.055933968 |
| RNA5SP259 | 0.046608116 | 5.05209025 |
| RP11-212E8.1 | 0.058581495 | 5.05017747 |
| RP11-219F10.1 | 0.082202078 | 5.047360346 |
| SNX19P1 | 0.082405182 | 5.038506312 |
| RP11-495K9.2 | 0.041486775 | 5.036466259 |
| RN7SKP68 | 0.099621841 | 5.029934439 |
| RP4-597A16.2 | 0.029809102 | 5.025937138 |
| ELMOD2 | 0.001505949 | 1.196240279 |
| SNORA71 | 0.112098384 | 5.021968129 |
| RP11-703H8.9 | 0.043139887 | 5.011651829 |
| LINC01100 | 0.079114621 | 4.998473906 |
| RP11-321E2.4 | 0.093786347 | 4.995616503 |
| ZNF503 | 0.001515846 | 1.36146743 |
| AC147651.1 | 0.002845495 | 4.985041926 |
| NOTCH1 | 0.001590001 | 0.677397267 |
| CRIP1P3 | 0.00793939 | 4.971244393 |
| Metazoa_SRP | 0.030753013 | 4.966828533 |
| AC023158.1 | 0.088038618 | 4.965397645 |
| RP11-119D9.4 | 0.129278794 | 4.962384803 |
| RP11-347E10.1 | 0.00674051 | 4.961416353 |
| MXRA5 | 0.001623822 | 1.678799328 |
| RP11-37O16.6 | 0.066978659 | 4.959346326 |
| AC005307.1 | 0.134000927 | 4.957798492 |
| RP11-499P1.1 | 0.10469702 | 4.952392214 |
| KLHDC7A | 0.001638488 | 4.048533209 |
| CTD-3064C13.1 | 0.07496313 | 4.941414747 |
| RN7SL670P | 0.033975712 | 4.933137088 |
| RP11-138I17.1 | 0.085448092 | 4.926571103 |
| FAM132B | 0.00164 | 2.216649312 |
| RP11-756J15.3 | 0.068123236 | 4.922814446 |
| ARGFXP1 | 0.019471723 | 4.912210856 |
| THUMPD3 | 0.001641898 | 1.185827607 |
| AP000797.2 | 0.065386298 | 4.900198185 |
| AP000345.1 | 0.074621657 | 4.894630439 |
| RN7SKP181 | 0.137605852 | 4.892536507 |
| AIFM1 | 0.001642723 | 1.303488038 |
| GKAP1 | 0.00165244 | 1.394084175 |
| Metazoa_SRP | 0.054271824 | 4.880365884 |
| MIR3180-2 | 0.062103509 | 4.877881801 |
| SMIM15 | 0.001654363 | 1.18085505 |
| FGFRL1 | 0.001654637 | 1.696305652 |
| RP3-382I10.2 | 0.006168613 | 4.861110597 |
| CTA-109P11.1 | 0.126630924 | 4.860069756 |
| XXyac-YM21GA2.3 | 0.129994912 | 4.830917566 |
| OR7A15P | 0.02897471 | 4.826558497 |
| CPD | 0.001658916 | 1.664510928 |
| GLCE | 0.001696925 | 1.306837991 |
| FP236240.2 | 0.066384214 | 4.821401622 |
| FAM47E-STBD1 | 0.001724288 | 3.031834671 |
| RN7SL845P | 0.017344848 | 4.810817933 |
| PGGT1B | 0.001731452 | 1.18092968 |
| AC113608.1 | 0.103201977 | 4.782830301 |
| RP11-457P14.5 | 0.013108539 | 4.780021362 |
| LLNLF-158E9.1 | 0.12431381 | 4.769306168 |
| RP11-568A7.3 | 0.008593795 | 4.764378061 |
| RP11-285G1.15 | 0.106101522 | 4.762172461 |
| THSD4 | 0.001737221 | 2.068796433 |
| OR9H1P | 0.038707523 | 4.752141979 |
| RP11-219H23.2 | 0.125877957 | 4.740690729 |
| RNU6-1181P | 0.069785601 | 4.739098086 |
| RP3-331H24.5 | 0.003648194 | 4.738684089 |
| CTD-2313N18.8 | 0.000454598 | 4.734259171 |
| MAT2B | 0.001738661 | 1.205358907 |
| AC010987.5 | 0.130659808 | 4.728771215 |
| CDYL2 | 0.001739462 | 1.543098272 |
| RNU6-520P | 0.110642693 | 4.721763001 |
| SNX18P23 | 0.144203066 | 4.718930158 |
| SNORA31 | 0.005267302 | 4.713108146 |
| CDC42SE2 | 0.001745036 | 1.276634742 |
| RP11-318G8.2 | 0.047144808 | 4.702775615 |
| OOSP1P1 | 0.122876511 | 4.702243501 |
| AC079325.5 | 0.148281818 | 4.702158351 |
| ATG4AP1 | 0.074301127 | 4.701053274 |
| RP11-116O18.1 | 0.149026596 | 4.696216981 |
| RP3-454B23.1 | 0.111584418 | 4.691834182 |
| RP11-346K17.3 | 0.122482026 | 4.683589965 |
| XPR1 | 0.001746067 | 1.412445421 |
| HMGB1P35 | 0.019427091 | 4.680546149 |
| NME1P1 | 0.076033425 | 4.678251963 |
| RP11-178L8.6 | 0.008989 | 4.677441146 |
| AC067969.2 | 0.09082984 | 4.670487743 |
| NUTF2P3 | 0.089258789 | 4.662014842 |
| MIR5693 | 0.037239961 | 4.661824862 |
| HAUS3 | 0.001749091 | 1.194699955 |
| ANKRD26P2 | 0.103292159 | 4.656247715 |
| RP11-324D17.1 | 0.005784018 | 4.654826208 |
| RP11-862G15.1 | 0.004202599 | 4.649442297 |
| RP11-744K17.7 | 0.03891348 | 4.645724547 |
| Y_RNA | 0.094228947 | 4.644717225 |
| DUSP9 | 0.001764877 | 2.121804389 |
| TMEM115 | 0.001780422 | 1.187263784 |
| MIR6075 | 0.090332286 | 4.637405395 |
| MTND4P32 | 0.008760654 | 4.62695926 |
| RP11-716O23.2 | 0.013740229 | 4.626738508 |
| AL022344.2 | 0.017781635 | 4.624316909 |
| RAP1BP1 | 0.008050238 | 4.622104772 |
| GUSB | 0.001795453 | 1.278231672 |
| LHFPL3-AS1 | 0.028244764 | 4.61533712 |
| RP11-244B22.3 | 0.039476273 | 4.613246558 |
| TCP10L | 0.001813264 | 1.575136704 |
| CCDC40 | 0.001829944 | 2.447260126 |
| AC133749.1 | 0.011852785 | 4.611024947 |
| RP11-345F18.1 | 0.034044152 | 4.610779275 |
| RP1-144F13.3 | 0.005055672 | 4.608003078 |
| RP11-20I23.3 | 0.001844522 | 2.429015913 |
| RN7SL653P | 0.107766363 | 4.598937174 |
| AL356310.1 | 0.004813118 | 4.597104662 |
| RP11-346C4.3 | 0.079080507 | 4.594213098 |
| AL138994.1 | 0.05652731 | 4.59023709 |
| Y_RNA | 0.135555456 | 4.58421263 |
| MIR8070 | 0.072615759 | 4.583081001 |
| RN7SL89P | 0.122882138 | 4.579836143 |
| Y_RNA | 0.035415306 | 4.579063437 |
| OR7E145P | 0.026798406 | 4.577008246 |
| BOD1P1 | 0.053750474 | 4.576358223 |
| RP11-233G1.4 | 0.087337266 | 4.56651507 |
| RP11-441M10.1 | 0.07018804 | 4.564007833 |
| OR52B6 | 0.00185238 | 7.243261413 |
| RP11-138H8.4 | 0.127254586 | 4.562739451 |
| IGHVIV-44-1 | 0.084498594 | 4.562073631 |
| RP11-362A1.1 | 0.071768894 | 4.55924934 |
| AC008268.1 | 0.116914627 | 4.557586771 |
| RNU1-19P | 0.081758078 | 4.555213581 |
| MIR3131 | 0.038107417 | 4.554987121 |
| U8 | 0.024067741 | 4.554579609 |
| CSN1S2AP | 0.096368516 | 4.554252899 |
| MTND4P16 | 0.06395478 | 4.549780759 |
| RP11-197K6.1 | 0.060234425 | 4.547277268 |
| AC007795.1 | 0.096262087 | 4.543859418 |
| RP3-380E11.2 | 0.091203459 | 4.540873154 |
| COX6CP3 | 0.074577784 | 4.535658296 |
| AC105393.1 | 0.008142836 | 4.533131661 |
| RP11-429A20.2 | 0.010434254 | 4.531922349 |
| TBC1D10A | 0.001857353 | 0.781602924 |
| VWA7 | 0.001906594 | 1.828736944 |
| MIR599 | 0.078496761 | 4.523725392 |
| Y_RNA | 0.042683464 | 4.520794614 |
| SEC13 | 0.001926154 | 1.19196291 |
| RP11-1041F24.1 | 0.132860916 | 4.506471856 |
| TUBBP11 | 0.053191842 | 4.499187437 |
| RP11-44L9.1 | 0.077025035 | 4.497016439 |
| RNU6-182P | 0.002215686 | 4.496136642 |
| HSPD1P21 | 0.002495716 | 4.494444113 |
| AC020703.1 | 0.136547203 | 4.478444331 |
| RPL23AP76 | 0.007498834 | 4.474889298 |
| RP11-740N7.3 | 0.034782073 | 4.473363132 |
| LINC01518 | 0.123933769 | 4.472138805 |
| CLCN4 | 0.001926763 | 1.833158797 |
| ANKRD30BP1 | 0.122361912 | 4.465466146 |
| RP3-461P17.6 | 0.043353008 | 4.459942064 |
| ARF4P3 | 0.111623639 | 4.45248749 |
| RP11-109P11.1 | 0.002432036 | 4.445861998 |
| RNU7-46P | 0.130261149 | 4.441338707 |
| RP11-398J10.2 | 0.103774835 | 4.440560876 |
| CSRP1 | 0.001946837 | 1.299674822 |
| CTC-327F10.1 | 0.132413574 | 4.433998574 |
| RNU6-145P | 0.01439463 | 4.429890883 |
| RN7SKP248 | 0.03403507 | 4.428325308 |
| RP11-435O11.5 | 0.005015784 | 4.427284535 |
| EOGT | 0.00195428 | 1.268971416 |
| RBM15B | 0.001975411 | 1.177042222 |
| MIR628 | 0.012259114 | 4.419956827 |
| RPL12P3 | 0.061249261 | 4.414799361 |
| SHBG | 0.001992649 | 1.419014143 |
| RP11-664H17.1 | 0.024818576 | 4.412552299 |
| RP11-331K21.1 | 0.03593046 | 4.411914093 |
| VN1R3 | 0.105053607 | 4.411451836 |
| RP11-300M6.1 | 0.05386877 | 4.409384365 |
| NEIL1 | 0.001998712 | 1.51077836 |
| TARID | 0.021296902 | 4.398504787 |
| GPR113 | 0.002005856 | 1.469730128 |
| RP11-447M12.2 | 0.127323477 | 4.386544169 |
| CD55 | 0.002026888 | 2.299119318 |
| FANK1 | 0.002028137 | 1.5294919 |
| AC013429.4 | 0.028413523 | 4.378105602 |
| RP5-967N21.7 | 0.036655662 | 4.374023307 |
| RP11-363G10.3 | 0.139036984 | 4.368449197 |
| FOXA2 | 0.00204401 | 4.085234457 |
| C3orf22 | 0.002053549 | 3.446716968 |
| AC078889.1 | 0.148801948 | 4.353959382 |
| LINC01392 | 0.054950412 | 4.353548249 |
| SLC6A21P | 0.046152196 | 4.351113519 |
| DPH3 | 0.002079855 | 1.197037806 |
| COQ10A | 0.002130645 | 1.25489634 |
| RP11-587D21.4 | 0.019673469 | 4.347932375 |
| ZIK1P1 | 0.024406267 | 4.344389389 |
| ASB14 | 0.002131139 | 1.291750725 |
| CTB-161M19.2 | 0.04470218 | 4.340823216 |
| CFAP46 | 0.002135713 | 4.10520041 |
| CSNK1A1P3 | 0.006441617 | 4.334677872 |
| RP11-265D20.1 | 0.112373443 | 4.330894716 |
| FAM181A-AS1 | 0.123433316 | 4.330448062 |
| BRINP1 | 0.002141395 | 4.215240914 |
| RNU6-78P | 0.105376876 | 4.32971328 |
| RP11-57J16.1 | 0.062048977 | 4.32174211 |
| RPL7P31 | 0.059310071 | 4.316338625 |
| VTI1BP4 | 0.000636545 | 4.314777064 |
| ZNF215 | 0.002146846 | 1.924198754 |
| TMBIM6 | 0.002168121 | 1.123874376 |
| RP5-905G11.3 | 0.002319519 | 4.309483072 |
| GNAI2 | 0.002171109 | 1.187833804 |
| NOTCH3 | 0.002197566 | 0.659063961 |
| APBB2 | 0.002199327 | 1.350888202 |
| RP11-489M13.3 | 0.002911028 | 4.302665353 |
| LL22NC03-30E12.13 | 0.135926768 | 4.298631233 |
| RP11-299L17.3 | 0.040692713 | 4.294818561 |
| OSTC | 0.002212884 | 1.167742384 |
| AC011899.10 | 0.00999872 | 4.29466836 |
| LINC00588 | 0.117065029 | 4.290613337 |
| AP006261.1 | 0.055226504 | 4.288296676 |
| RP4-650F12.2 | 0.01922994 | 4.283433877 |
| AC027348.1 | 0.037284086 | 4.276510908 |
| RP11-778H2.1 | 0.031081753 | 4.271705103 |
| AC078974.2 | 0.109512287 | 4.270905548 |
| RP11-78A19.2 | 0.114237192 | 4.270197821 |
| CTC-513N18.6 | 0.132012422 | 4.269713486 |
| FLJ33581 | 0.122527313 | 4.269244304 |
| LRSAM1 | 0.0022224 | 0.867252998 |
| AL118496.1 | 0.025802632 | 4.264504053 |
| LRP2BP | 0.002230991 | 1.416156449 |
| SLC25A41 | 0.002275806 | 0.545497798 |
| CTC-529L17.2 | 0.030951858 | 4.261394183 |
| EPHX3 | 0.002279151 | 0.605134895 |
| DRAM1 | 0.002299257 | 1.669560459 |
| SLC46A1 | 0.002309931 | 1.372217426 |
| RNU4-58P | 0.075295449 | 4.242303933 |
| N6AMT1 | 0.002314783 | 1.217650829 |
| RP11-410C4.4 | 0.031869895 | 4.235710049 |
| CDK2 | 0.002323754 | 1.208466615 |
| MAP2K6 | 0.002332552 | 1.845815298 |
| RP3-407E4.3 | 0.09718876 | 4.229260298 |
| BAK1P2 | 0.033978207 | 4.22123037 |
| RP11-746P2.6 | 0.079051674 | 4.221097848 |
| RP11-263E1.1 | 0.058099885 | 4.217807407 |
| AL160011.1 | 0.143043327 | 4.216828019 |
| LCORL | 0.002334692 | 1.248076875 |
| AC016717.1 | 0.043763745 | 4.212681182 |
| MTND4P6 | 0.122548291 | 4.212460298 |
| PCGF3 | 0.002339423 | 1.239705495 |
| ZNF335 | 0.002388496 | 0.846345272 |
| AC022384.1 | 0.105055398 | 4.20876697 |
| HLA-DPA2 | 0.000636608 | 4.205218708 |
| RP11-190G13.4 | 0.150924716 | 4.197727822 |
| OR7E36P | 0.14871801 | 4.18530467 |
| RP13-870H17.3 | 0.049282009 | 4.178511386 |
| NDP | 0.002392875 | 5.16725973 |
| RRM2P4 | 0.117347848 | 4.16632255 |
| RP11-432J9.3 | 0.149492584 | 4.166020773 |
| RP11-89N17.2 | 0.008253476 | 4.160472511 |
| RP11-17A4.3 | 0.022128996 | 4.147758762 |
| AL356261.1 | 0.008786343 | 4.146962895 |
| RP11-686F15.2 | 0.076511957 | 4.14629885 |
| RP11-460B17.3 | 0.139183423 | 4.139016506 |
| LINC00710 | 0.012344888 | 4.137037033 |
| SDR16C6P | 0.056021234 | 4.136245939 |
| MIR575 | 0.092651198 | 4.134857094 |
| CHIAP3 | 0.053414495 | 4.134633356 |
| RP11-554D14.6 | 0.144303596 | 4.129724141 |
| SNORD116-8 | 0.071551118 | 4.128750343 |
| TLR8-AS1 | 0.096127657 | 4.128253707 |
| RNU6-1300P | 0.029861115 | 4.126128106 |
| CH17-478G19.2 | 0.037928583 | 4.124482297 |
| RP11-275N1.1 | 0.027034569 | 4.114433301 |
| RNU6-667P | 0.121970113 | 4.114166678 |
| RP11-438E8.2 | 0.10003604 | 4.113052897 |
| RP11-143E21.3 | 0.038882963 | 4.108239991 |
| ZBED3 | 0.002395333 | 1.605112591 |
| TMEM67 | 0.00240511 | 1.309085666 |
| FAM81B | 0.002464425 | 5.516014088 |
| CTD-2311M21.2 | 0.097016786 | 4.101575312 |
| RP11-17E3.1 | 0.133406783 | 4.100141408 |
| AL138726.1 | 0.072784252 | 4.09629038 |
| MIR548N | 0.007721392 | 4.095560289 |
| RP11-939C17.4 | 0.097565084 | 4.093641119 |
| KIAA1324 | 0.002483779 | 3.08601928 |
| KIAA0125P2 | 0.15065027 | 4.087412228 |
| ASPA | 0.002488484 | 2.164880411 |
| ESRRG | 0.002495025 | 3.203445782 |
| THAP6 | 0.002509932 | 1.17034828 |
| CSPG4P13 | 0.00956902 | 4.082175003 |
| PBRM1 | 0.002511165 | 1.241111808 |
| RN7SL541P | 0.009981239 | 4.078337595 |
| SDC4P | 0.028427676 | 4.078198893 |
| RP11-300G22.2 | 0.009423072 | 4.077937902 |
| RPL36P20 | 0.029757362 | 4.071600269 |
| IGLVVI-25-1 | 0.098336901 | 4.070895242 |
| RP11-200K12.3 | 0.059445407 | 4.067147814 |
| RP11-116N8.4 | 0.115084212 | 4.064186889 |
| MINPP1 | 0.002521946 | 1.277771043 |
| TEKT2 | 0.002524559 | 3.704904376 |
| RP11-357G3.1 | 0.00320175 | 4.056867067 |
| Y_RNA | 0.070089394 | 4.05589259 |
| EFHC2 | 0.002532253 | 2.642007416 |
| FAM99B | 0.108660475 | 4.054508247 |
| FBXL13 | 0.002551936 | 1.873128438 |
| AC016831.5 | 0.020278006 | 4.049603033 |
| PKP1 | 0.002552768 | 0.562809966 |
| DNAH5 | 0.002555665 | 1.940888368 |
| TEX36-AS1 | 0.143935086 | 4.047583182 |
| SLC25A38 | 0.002565171 | 1.253697851 |
| RP11-616M22.1 | 0.032716357 | 4.037390226 |
| NAV2 | 0.002566998 | 1.385241456 |
| ENPP7P3 | 0.047527462 | 4.033805579 |
| TXNP2 | 0.13163175 | 4.029118152 |
| RP11-440G9.1 | 0.062730952 | 4.02745246 |
| SLC39A8 | 0.002584974 | 2.379918211 |
| RP11-390E23.3 | 0.015762336 | 4.015806151 |
| CTD-2233C11.2 | 0.098756153 | 4.011308905 |
| RN7SL771P | 0.098867513 | 4.009302349 |
| AC022872.1 | 0.143551188 | 4.003169471 |
| RP4-666F24.3 | 0.095558895 | 3.993872656 |
| MESTP4 | 0.09776848 | 3.993786501 |
| FBXL5 | 0.002603356 | 1.2033958 |
| LA16c-335H7.2 | 0.1382249 | 3.982296061 |
| DYX1C1-CCPG1 | 0.000525977 | 3.980065201 |
| ATP5EP1 | 0.085943969 | 3.979198375 |
| RN7SL8P | 0.050833893 | 3.976602251 |
| AC093807.1 | 0.103488261 | 3.976162741 |
| RP13-977E11.1 | 0.102022194 | 3.975736398 |
| KRTAP5-4 | 0.002653785 | 4.414630988 |
| GALNT10 | 0.002663148 | 1.452890437 |
| MIR367 | 0.044100178 | 3.963005871 |
| RP11-151G12.2 | 0.086786237 | 3.960855028 |
| RN7SKP130 | 0.02249445 | 3.959333451 |
| RP11-473C19.1 | 0.120142661 | 3.951713137 |
| AL512791.1 | 0.00920845 | 3.950543568 |
| RP11-10A14.6 | 0.021414086 | 3.947977978 |
| ICA1 | 0.002668352 | 1.623424728 |
| IGHVIII-13-1 | 0.070380393 | 3.938604346 |
| EEF1A1P41 | 0.04770453 | 3.93594468 |
| CCPG1 | 0.002681018 | 1.314578749 |
| MTCO2P4 | 0.121435436 | 3.93162896 |
| RP11-547C13.1 | 0.120861703 | 3.928692401 |
| RP1-65J11.5 | 0.030183492 | 3.928268096 |
| AP000654.4 | 0.000881647 | 3.928023651 |
| AC139099.7 | 0.005474022 | 3.924213253 |
| ULK4 | 0.00268514 | 1.307031391 |
| RP11-380P13.1 | 0.10099707 | 3.921076251 |
| RP11-439C15.5 | 0.007932522 | 3.921020129 |
| RP11-673D15.7 | 0.127080195 | 3.9171698 |
| RP5-974N19.1 | 0.151165514 | 3.915092679 |
| CCDC160 | 0.002689842 | 2.79600372 |
| RPL23P4 | 0.022953771 | 3.905659976 |
| AP001619.1 | 0.025622334 | 3.898108567 |
| PHB2P1 | 0.078099088 | 3.896828206 |
| LCA5L | 0.00271215 | 1.472882213 |
| AC098869.1 | 0.031581851 | 3.895900625 |
| RNA5SP210 | 0.113655288 | 3.894026275 |
| RP11-662M24.2 | 0.018745682 | 3.890062763 |
| RP4-788P17.1 | 0.137319105 | 3.888523223 |
| OSBP2 | 0.002718089 | 2.521100666 |
| RP11-158I3.3 | 0.028223618 | 3.883524599 |
| AE000661.36 | 0.131236937 | 3.882080276 |
| RP11-486M23.3 | 0.089160059 | 3.876393109 |
| PTP4A1P1 | 0.004053533 | 3.86388206 |
| RP11-363G10.2 | 0.12995884 | 3.860663903 |
| RP11-399H11.3 | 0.049943482 | 3.857818164 |
| MAK | 0.002756943 | 2.603653756 |
| SHROOM3 | 0.002769195 | 1.68055235 |
| LAP3P1 | 0.007007433 | 3.8524799 |
| RP11-152K4.2 | 0.048133442 | 3.851946062 |
| SNORA36 | 0.130765372 | 3.850171494 |
| RP11-269F21.3 | 0.112185575 | 3.849633841 |
| AC012485.2 | 0.008118448 | 3.846785905 |
| Y_RNA | 0.049337141 | 3.845674613 |
| ZNF214 | 0.002786934 | 1.618720253 |
| RP11-79C6.1 | 0.079049228 | 3.839831934 |
| PPFIA3 | 0.002788486 | 0.672365985 |
| RP11-460I19.2 | 0.067029029 | 3.838471272 |
| SLC26A2 | 0.00280904 | 1.554935059 |
| AC019100.3 | 0.040223725 | 3.831134439 |
| DDIT4 | 0.002810158 | 1.528383122 |
| RP11-543F8.2 | 0.03838825 | 3.828893161 |
| RP11-118K6.2 | 0.112303873 | 3.828884118 |
| RP11-471N19.1 | 0.071675841 | 3.828631632 |
| RP11-78A19.4 | 0.017918887 | 3.823441426 |
| CTD-2530H12.1 | 0.063626562 | 3.818332462 |
| CH507-210P18.3 | 0.056580779 | 3.816690529 |
| IQUB | 0.002810927 | 2.252984838 |
| CTD-2139B15.1 | 0.1474042 | 3.806461818 |
| RP11-642D6.1 | 0.09709134 | 3.799497099 |
| RP11-503N18.4 | 0.032075721 | 3.797198508 |
| RP11-697G4.4 | 0.008472996 | 3.795413547 |
| LINC00507 | 0.127699975 | 3.795006958 |
| RP11-82L7.4 | 0.085856756 | 3.793616065 |
| LINC01533 | 0.023418741 | 3.791704408 |
| RNU6-302P | 0.003777873 | 3.787941573 |
| RP11-314P15.2 | 0.106028414 | 3.784448088 |
| LL0XNC01-39B3.1 | 0.007493946 | 3.784155243 |
| RP11-32B5.8 | 0.071167346 | 3.781938238 |
| RP11-375B1.1 | 0.059081409 | 3.781345019 |
| DYDC1 | 0.002817042 | 3.886800522 |
| MTND2P8 | 0.128762603 | 3.780447067 |
| ALG13 | 0.002821359 | 1.198679094 |
| RN7SL159P | 0.03205548 | 3.773121318 |
| RP11-80B9.4 | 0.049955903 | 3.769384086 |
| RN7SKP240 | 0.045645687 | 3.768511081 |
| AP001048.4 | 0.027203572 | 3.768054362 |
| RP11-302F12.3 | 0.013048881 | 3.766511815 |
| RP11-384C12.1 | 0.035599486 | 3.765614911 |
| RP11-118M12.2 | 0.095413773 | 3.764584045 |
| CTD-2010I22.2 | 0.144398181 | 3.759318853 |
| RP11-1105O14.1 | 0.017939432 | 3.7592655 |
| RP11-402P6.13 | 0.112620041 | 3.758909583 |
| ABCD1P5 | 0.067364007 | 3.757125966 |
| SHROOM1 | 0.002837861 | 1.315823144 |
| CTD-2228A4.1 | 0.027630062 | 3.750516074 |
| RP11-468N14.5 | 0.109500362 | 3.747892161 |
| AC092198.1 | 0.076841947 | 3.740002801 |
| Y_RNA | 0.141606914 | 3.738901195 |
| ARHGAP26 | 0.002854338 | 1.710625553 |
| REXO1L1P | 0.137325577 | 3.729012566 |
| SNORD105 | 0.001586523 | 3.725615546 |
| RNU6-598P | 0.131786293 | 3.724544812 |
| CCDC96 | 0.002855488 | 1.57541102 |
| RP11-15K2.2 | 0.055966435 | 3.720998442 |
| ILF2P2 | 0.010656977 | 3.720362622 |
| CTD-2263F21.1 | 0.0878774 | 3.718140446 |
| Y_RNA | 0.013864101 | 3.716337252 |
| RP11-70F11.8 | 0.024504041 | 3.715739844 |
| RP11-496N12.6 | 0.007639296 | 3.711866894 |
| NHLRC2 | 0.002906246 | 1.206154113 |
| LINC01203 | 0.140701244 | 3.710032245 |
| PIFO | 0.002937206 | 4.338587127 |
| RP11-43F13.3 | 0.071389969 | 3.70720203 |
| SLC12A2 | 0.002960957 | 1.631423079 |
| RP11-7N14.1 | 0.117598678 | 3.702505133 |
| TIGD4 | 0.002963136 | 1.93061749 |
| RP11-323I1.1 | 0.150681988 | 3.696885767 |
| RP11-44H4.1 | 0.109599104 | 3.696570507 |
| RNU6-73P | 0.146784284 | 3.691982954 |
| RP11-147K6.2 | 0.138589972 | 3.691071666 |
| RP11-382N13.6 | 0.088161797 | 3.6875167 |
| RP11-65J3.2 | 0.047756509 | 3.687193721 |
| RP13-488H8.1 | 0.03009059 | 3.686592834 |
| RP1-200K18.1 | 0.072878641 | 3.686057825 |
| CD63 | 0.002982513 | 1.273869324 |
| GOLGA8DP | 0.028920928 | 3.676567762 |
| RPL31P50 | 0.09628657 | 3.673676105 |
| LINC01082 | 0.150620749 | 3.668870706 |
| RP11-461O14.3 | 0.021343991 | 3.668315111 |
| RP11-822E23.6 | 0.050098391 | 3.667400774 |
| LINC01250 | 0.036076455 | 3.667387124 |
| RP11-218D6.3 | 0.138999835 | 3.664638972 |
| AC109465.1 | 0.048274572 | 3.664532982 |
| BVES | 0.002992295 | 1.951244905 |
| RP11-139K4.2 | 0.127883942 | 3.663836266 |
| TBC1D3F | 0.002997345 | 3.065533653 |
| CLK3P2 | 0.02228687 | 3.658747142 |
| RBBP7 | 0.003002704 | 1.216624164 |
| NANOGP9 | 0.094250823 | 3.644282132 |
| RP11-746P2.5 | 0.077832251 | 3.644262293 |
| RP11-809H16.5 | 0.00504935 | 3.642598222 |
| AC104389.28 | 0.098448804 | 3.640706083 |
| FNDC3CP | 0.038105931 | 3.639679466 |
| RNU7-186P | 0.001146059 | 3.639630991 |
| AP000233.3 | 0.050981102 | 3.639473081 |
| NME5 | 0.003019852 | 3.581644681 |
| MRPL48P1 | 7.50E-05 | 3.635553202 |
| MIR1208 | 0.119009039 | 3.633984151 |
| Y_RNA | 0.108310729 | 3.632623651 |
| RP1-302G2.5 | 0.003641992 | 3.631914163 |
| PELO | 0.003024161 | 1.201432688 |
| RNU6-1245P | 0.037485631 | 3.628670156 |
| RN7SKP11 | 0.022911038 | 3.628599437 |
| MIR1288 | 0.145408619 | 3.626617154 |
| RP11-394A14.2 | 0.018655001 | 3.626289203 |
| AP003550.1 | 0.146147798 | 3.620164077 |
| AC114755.4 | 0.070179559 | 3.619572203 |
| RP11-299H22.3 | 0.089895827 | 3.61668133 |
| RP11-431D12.1 | 0.09175038 | 3.616230702 |
| LINC00930 | 0.012989339 | 3.615806284 |
| RP11-126O1.4 | 0.083745841 | 3.611088424 |
| CLDN10-AS1 | 0.01701425 | 3.609922242 |
| TBC1D19 | 0.00303713 | 1.259166449 |
| AZI2 | 0.003059766 | 1.229264184 |
| FTLP19 | 0.140032026 | 3.589688594 |
| AC093162.1 | 0.084132038 | 3.588242693 |
| DNAL1 | 0.003059871 | 1.291979569 |
| PMCHL1 | 0.106256929 | 3.584868911 |
| TCF15 | 0.003065439 | 0.547786121 |
| RP4-598P13.1 | 0.073744314 | 3.580748554 |
| MIR6806 | 0.03393817 | 3.579658147 |
| MAGI1-AS1 | 0.033037532 | 3.579007196 |
| CTD-2023N9.3 | 0.14968034 | 3.577884457 |
| LINC01387 | 0.123869768 | 3.576362061 |
| CYCSP29 | 0.1355897 | 3.575404395 |
| RP11-368I23.2 | 0.138373825 | 3.57414899 |
| Y_RNA | 0.076072818 | 3.572504905 |
| U7 | 0.008449676 | 3.569534555 |
| AC007099.1 | 0.018398203 | 3.567675105 |
| EFHC1 | 0.00306709 | 1.66422559 |
| APPL1 | 0.003091232 | 1.216213004 |
| ALDH1L1-AS2 | 0.001669498 | 3.552997141 |
| AC093911.1 | 0.145765854 | 3.549173739 |
| MSL3 | 0.003109386 | 1.172660955 |
| MTND6P18 | 0.02854214 | 3.546846209 |
| RP11-45K10.2 | 0.086060193 | 3.545370578 |
| MIR3912 | 0.067613991 | 3.542801284 |
| ARRDC3 | 0.003121284 | 1.392767961 |
| RP11-231E4.3 | 0.070187305 | 3.539417458 |
| NR2E3 | 0.003129286 | 1.83614749 |
| snoU13 | 0.016269662 | 3.53468024 |
| RNA5SP103 | 0.122203905 | 3.533701327 |
| RP11-298C2.1 | 0.003163225 | 3.531902039 |
| PTPRG | 0.003131219 | 1.631233109 |
| CTNNB1 | 0.00313841 | 1.29647345 |
| MIR3116-1 | 0.012434948 | 3.527712568 |
| COG6 | 0.003140185 | 1.233760427 |
| CLMP | 0.003159905 | 1.845443162 |
| CTD-2396E7.7 | 0.141789003 | 3.520094721 |
| ZNF662 | 0.003171376 | 1.71950319 |
| RP11-488I20.8 | 0.143328563 | 3.515136115 |
| CCDC173 | 0.003211658 | 3.056481456 |
| INPP5J | 0.003212412 | 1.799754671 |
| LURAP1 | 0.003247029 | 1.583013891 |
| RP11-568A7.1 | 0.095707089 | 3.507832243 |
| FOXO1B | 0.054548262 | 3.504102937 |
| CEBPA | 0.003257938 | 0.680145298 |
| RN7SL583P | 0.015606325 | 3.499361952 |
| SLC38A9 | 0.003276992 | 1.160480257 |
| Metazoa_SRP | 0.00670813 | 3.496801774 |
| LINC01059 | 0.01404566 | 3.496023774 |
| RP11-1396O13.1 | 0.006655461 | 3.494778888 |
| SLC34A2 | 0.003277177 | 3.557940536 |
| CTD-2114J12.1 | 0.00543703 | 3.492637545 |
| SNORA62 | 0.070309121 | 3.492425374 |
| RP11-38O14.6 | 0.1218187 | 3.491152271 |
| RP11-3B12.3 | 0.10599053 | 3.490015344 |
| RP13-210D15.1 | 0.116715661 | 3.488781803 |
| RP1-283K11.2 | 0.048702327 | 3.475216048 |
| RNU6-1064P | 0.005798677 | 3.474599845 |
| ARFIP1 | 0.003283649 | 1.197077472 |
| RNU6-1217P | 0.032239929 | 3.470992385 |
| SLC22A4 | 0.003290662 | 1.682849993 |
| RP4-801G22.2 | 0.005766579 | 3.467256303 |
| RP11-748L13.6 | 0.112081183 | 3.461976966 |
| MIR5703 | 0.135379749 | 3.460100253 |
| C8orf59P2 | 0.006644656 | 3.4542195 |
| RP3-453P22.2 | 0.089341242 | 3.45021333 |
| PPAPDC1B | 0.003308658 | 1.306396383 |
| RP11-177N22.2 | 0.033917216 | 3.450033745 |
| RP11-535E8.2 | 0.032601029 | 3.447757308 |
| FDXR | 0.003337706 | 0.745285087 |
| TUSC8 | 0.073562491 | 3.44685033 |
| DAP | 0.003360875 | 1.250265178 |
| RP11-87G24.3 | 0.010396711 | 3.445175557 |
| AC005255.5 | 0.05512654 | 3.441899834 |
| ZBTB38 | 0.003363808 | 1.369151505 |
| RP4-609E1.2 | 0.013994156 | 3.43800064 |
| RP11-4N23.1 | 0.070352012 | 3.437852978 |
| AL591668.1 | 0.004958503 | 3.437376194 |
| Evf-2_5p | 0.1340099 | 3.435115642 |
| MIR615 | 0.061680187 | 3.429818259 |
| RSL24D1P8 | 0.000223401 | 3.427661943 |
| RP11-139E19.3 | 0.005233896 | 3.426070642 |
| AC067742.1 | 0.05965115 | 3.423610553 |
| ABCD3 | 0.00336718 | 1.232169823 |
| AC008171.1 | 0.047514176 | 3.414861697 |
| RNA5SP229 | 0.016023505 | 3.414752206 |
| RP11-319F12.2 | 0.040276186 | 3.410919655 |
| RP11-301G7.1 | 0.143687461 | 3.409885831 |
| NUPL1P1 | 0.031526193 | 3.409082494 |
| CTSLP8 | 0.11795641 | 3.405404623 |
| SPRY1 | 0.00336756 | 1.73791367 |
| GXYLT1P2 | 0.091474346 | 3.399023265 |
| RNU4-53P | 0.020686498 | 3.397631262 |
| UQCRFS1P3 | 0.122244845 | 3.397476885 |
| B3GNTL1P1 | 0.003490363 | 3.39747656 |
| NAALADL2-AS1 | 0.074776805 | 3.396767768 |
| LINC00469 | 0.12203026 | 3.394700875 |
| RP11-66H6.3 | 0.128753203 | 3.392201882 |
| RP11-102K13.5 | 0.002254417 | 3.391708084 |
| RNU6-1304P | 0.114714717 | 3.390898892 |
| JAG1 | 0.003375868 | 0.667304389 |
| RP11-276H1.2 | 0.068065952 | 3.38271939 |
| RBPMSLP | 0.015890053 | 3.379142748 |
| RP5-857K21.11 | 0.093733096 | 3.377910042 |
| RBM5 | 0.003377524 | 1.191870276 |
| GTF2IRD2 | 0.003403281 | 1.327585488 |
| TMPRSS3 | 0.003410224 | 2.292140058 |
| CASC1 | 0.00341724 | 3.091510816 |
| RP11-240G22.1 | 0.060559704 | 3.366646733 |
| RP11-579O24.3 | 0.00883444 | 3.366591354 |
| CLEC4GP1 | 0.064424101 | 3.364726482 |
| RPL7AP69 | 0.104116354 | 3.364349163 |
| RASD1 | 0.003421651 | 4.088525037 |
| AC234917.1 | 0.090726642 | 3.36019151 |
| CTB-51J22.1 | 0.001841455 | 3.360129212 |
| Y_RNA | 0.132270018 | 3.359404859 |
| RN7SL683P | 0.054731156 | 3.358240824 |
| FAM134B | 0.003433868 | 1.79277957 |
| MIR4450 | 0.018882413 | 3.356371446 |
| NLRP2 | 0.003463783 | 1.816107826 |
| YIF1B | 0.003475693 | 0.783426439 |
| FAM46A | 0.003482788 | 1.43672914 |
| ZNF620 | 0.003522704 | 1.423236529 |
| FGF14-IT1 | 0.001246011 | 3.349673168 |
| AC092168.3 | 0.143462956 | 3.348711182 |
| RP11-1123I8.1 | 0.003296732 | 3.347014936 |
| MIR5092 | 0.144775547 | 3.34694676 |
| Metazoa_SRP | 0.092033864 | 3.337059962 |
| BBS12 | 0.00352548 | 1.343109798 |
| TBC1D3P1-DHX40P1 | 0.096772687 | 3.334610427 |
| LYARP1 | 0.075066159 | 3.333981879 |
| RP11-314B1.2 | 0.029551061 | 3.331815957 |
| RORC | 0.003526197 | 1.634232658 |
| RP5-998N21.7 | 0.07603172 | 3.330585888 |
| WFDC8 | 0.003545046 | 4.362409072 |
| AADACL4 | 0.003554551 | 4.262252236 |
| AC078993.1 | 0.044395733 | 3.328136645 |
| Y_RNA | 0.147355045 | 3.325736619 |
| MIR4671 | 0.097455331 | 3.323902418 |
| C5orf49 | 0.003557414 | 4.245995537 |
| AC005336.5 | 0.148074261 | 3.323044706 |
| BLOC1S4 | 0.003651115 | 1.171925997 |
| RP11-481F24.3 | 0.029326024 | 3.313387398 |
| MIR875 | 0.123028459 | 3.312934449 |
| RNA5SP33 | 0.015544564 | 3.312892335 |
| SFMBT1 | 0.003657873 | 1.225561362 |
| SLC6A6 | 0.003672533 | 1.422173257 |
| CTPS2 | 0.003687809 | 1.284620459 |
| RP11-382B18.5 | 0.135815774 | 3.301540118 |
| PHLDB3 | 0.003697046 | 0.666503794 |
| RP11-259A24.1 | 0.048296282 | 3.301042172 |
| AL356791.1 | 0.143962262 | 3.296328492 |
| NAA20 | 0.003705568 | 0.757976761 |
| RIPK2 | 0.003709881 | 1.260069019 |
| CD99L2 | 0.003739059 | 1.327544374 |
| RP11-789C1.1 | 0.110350939 | 3.291262633 |
| ZNF652P1 | 0.093444088 | 3.290452837 |
| OR1X1P | 0.12820177 | 3.289637653 |
| RUFY1 | 0.003741233 | 1.159490455 |
| RNU6-316P | 0.048976657 | 3.283179885 |
| AC097382.1 | 0.036145062 | 3.279475148 |
| RPGR | 0.003750253 | 1.438740366 |
| HUNK-AS1 | 0.054007054 | 3.273531307 |
| RP11-15E1.6 | 0.036110308 | 3.272264692 |
| CTD-2537I9.15 | 0.008769746 | 3.271221451 |
| RP11-434D2.2 | 0.100784849 | 3.269087625 |
| MTUS2-AS2 | 0.116675291 | 3.267682608 |
| RP11-140M13.1 | 0.045123427 | 3.266342312 |
| RP11-588F10.1 | 0.065018499 | 3.264741529 |
| CTD-2089N3.3 | 0.106830856 | 3.26155846 |
| MTATP6P3 | 0.037491169 | 3.260713014 |
| RP11-438D14.2 | 0.108079585 | 3.259858374 |
| RP11-84N19.1 | 0.006234474 | 3.259392999 |
| RP11-108M9.2 | 0.07278233 | 3.256470056 |
| ZDHHC18 | 0.003765044 | 0.818321488 |
| SHQ1 | 0.003768443 | 1.188720538 |
| DYX1C1 | 0.00376858 | 1.515123289 |
| RP11-844P9.4 | 0.139044778 | 3.252240207 |
| TMEM192 | 0.003815995 | 1.228640378 |
| AC113167.1 | 0.042245571 | 3.25116647 |
| RPL7AP57 | 0.038761794 | 3.250147238 |
| RP4-718N17.2 | 0.147488064 | 3.245289355 |
| RPL12P18 | 0.009174536 | 3.243938915 |
| RP11-90D4.3 | 0.058144033 | 3.238680201 |
| LINC01335 | 0.146656366 | 3.231967263 |
| CASP7 | 0.003823908 | 1.258169727 |
| AC104938.1 | 0.006689867 | 3.228608434 |
| RAD54L2 | 0.003900353 | 1.190775663 |
| PPP2CA | 0.003928475 | 1.125972893 |
| RAB5A | 0.003942232 | 1.187463966 |
| RP11-111E14.2 | 0.115787068 | 3.21786711 |
| GS1-21A4.1 | 0.095651835 | 3.215781442 |
| JADE1 | 0.003968066 | 1.26465872 |
| RP11-359J6.1 | 0.061311035 | 3.214171627 |
| SNORA63 | 0.150547001 | 3.212509201 |
| RP11-472F14.4 | 0.043194966 | 3.20828659 |
| MOSPD1 | 0.003981285 | 1.249041782 |
| LINC00626 | 0.102988022 | 3.207287501 |
| DCP1A | 0.003984176 | 1.188326182 |
| FDX1 | 0.004004272 | 1.216084138 |
| RP11-189E14.5 | 0.064133901 | 3.202753422 |
| GPER1 | 0.004004486 | 2.024206591 |
| RNA5SP469 | 0.015801103 | 3.198718354 |
| CTD-2140G10.2 | 0.069043732 | 3.19851356 |
| MTND2P31 | 0.093192572 | 3.195676649 |
| RP11-51G5.1 | 0.116270288 | 3.195038286 |
| CRYZ | 0.004007966 | 1.378884803 |
| CTD-2272G21.3 | 0.053529412 | 3.189499821 |
| RP4-673D20.6 | 0.077284801 | 3.188854083 |
| AC012512.1 | 0.027686331 | 3.185516987 |
| RPL12P24 | 0.081832164 | 3.183398072 |
| PPIL6 | 0.004010803 | 2.539753438 |
| HMGB1P44 | 0.031034418 | 3.180862207 |
| PHTF1 | 0.004011694 | 1.28724533 |
| PBDC1 | 0.00402795 | 1.226595056 |
| MIR4642 | 0.000305323 | 3.17561474 |
| RNA5SP334 | 0.018895663 | 3.175071171 |
| MICAL2 | 0.004110403 | 1.576114403 |
| RNU6-177P | 0.114226238 | 3.172480314 |
| RP11-144H23.2 | 0.006010607 | 3.170799045 |
| Y_RNA | 0.108289672 | 3.170704011 |
| OR7E162P | 0.031788315 | 3.165917641 |
| EFS | 0.004125052 | 0.70439225 |
| ZFP28 | 0.004130191 | 1.757593296 |
| TRPV1 | 0.004145648 | 1.458794142 |
| RNU6-863P | 0.005782809 | 3.161734398 |
| LINC01225 | 0.005584908 | 3.159164716 |
| BEND7 | 0.004171461 | 1.75317698 |
| MIR548I2 | 0.042062163 | 3.158379638 |
| MKKS | 0.004192441 | 0.832309542 |
| AC083864.4 | 0.098930022 | 3.156289356 |
| IQCH | 0.004203722 | 1.652830334 |
| AC092155.4 | 0.054398835 | 3.154721164 |
| FAM200B | 0.004207964 | 1.230154358 |
| CTD-2008P7.5 | 0.04291794 | 3.154493817 |
| RNU6ATAC38P | 0.032236397 | 3.153622892 |
| RP4-712E4.2 | 0.014892593 | 3.152860089 |
| RP11-462G22.2 | 0.076366005 | 3.149157005 |
| PPP1R2P10 | 0.072827353 | 3.149150901 |
| NOS2P3 | 0.02745005 | 3.143946209 |
| RP11-552D8.1 | 0.00090918 | 3.143245069 |
| GOLM1 | 0.004217511 | 1.556629499 |
| RP11-158I23.1 | 0.008092832 | 3.141775096 |
| RPS15AP34 | 0.109658692 | 3.13932921 |
| RAB36 | 0.00422193 | 1.724417076 |
| SCOC | 0.004225194 | 1.208270461 |
| FENDRR | 0.018278029 | 3.138059684 |
| PBOV1 | 0.004226695 | 2.369553349 |
| RP11-80B9.1 | 0.070119951 | 3.135498961 |
| RP11-326C3.13 | 0.000997101 | 3.134426272 |
| RNU6-514P | 0.075164654 | 3.130196277 |
| AC091878.1 | 0.101858687 | 3.128939064 |
| Y_RNA | 0.047080379 | 3.127399236 |
| RP11-1057B8.2 | 0.021862672 | 3.12666591 |
| RP3-382I10.3 | 0.00405786 | 3.124060995 |
| ARSEP1 | 0.08763144 | 3.12337742 |
| RP11-58A18.1 | 0.071875992 | 3.122404057 |
| CTC-529L17.1 | 0.107837562 | 3.120907453 |
| LINC00237 | 0.018921974 | 3.118560303 |
| NUP54 | 0.004234442 | 1.162943056 |
| RP11-666O2.4 | 0.005317649 | 3.117310823 |
| OR7E122P | 0.102312992 | 3.116096407 |
| C8orf4 | 0.0042473 | 2.140174268 |
| RP1-65P5.5 | 0.014946959 | 3.114904028 |
| LINC01581 | 0.093916883 | 3.111819619 |
| ANKRA2 | 0.004257657 | 1.218210741 |
| AC116050.1 | 0.059046106 | 3.111191768 |
| TATDN2P1 | 0.006742958 | 3.107009235 |
| CENPK | 0.004277806 | 1.255089393 |
| RP11-35J10.7 | 0.002188129 | 3.104412139 |
| RP11-661C8.3 | 0.072563851 | 3.101901756 |
| AC008703.1 | 0.012261301 | 3.099483809 |
| Y_RNA | 0.033049079 | 3.09772665 |
| AC024132.1 | 0.072339064 | 3.096684354 |
| RNU5F-4P | 0.121668072 | 3.095700388 |
| RP11-667K14.10 | 0.003894007 | 3.09368796 |
| RP11-680F20.6 | 0.036514118 | 3.093294678 |
| RP11-298O21.2 | 0.005455604 | 3.092373217 |
| SPRY3 | 0.004279568 | 2.042262174 |
| LINC01552 | 0.015276162 | 3.089533955 |
| RSPH4A | 0.004281427 | 4.423002381 |
| RP3-400B16.3 | 0.075143905 | 3.085085829 |
| ICE2P1 | 0.011702578 | 3.08314687 |
| RP4-683M8.2 | 0.079158802 | 3.080148046 |
| RPS6KA2 | 0.004288239 | 1.469118515 |
| Y_RNA | 0.018786244 | 3.078682053 |
| AC090505.5 | 0.013664934 | 3.075202843 |
| SNORD111B | 0.06597223 | 3.07255967 |
| REPS2 | 0.004309895 | 1.618029311 |
| AC064834.2 | 0.010543099 | 3.069367856 |
| AC107083.1 | 0.022317778 | 3.068911959 |
| SEPT7P3 | 0.057931089 | 3.067471935 |
| CTA-363E6.2 | 0.01397476 | 3.067164364 |
| SRD5A1 | 0.004405982 | 0.664526597 |
| RP11-366L18.1 | 0.033176833 | 3.065084187 |
| MIR4779 | 0.101872033 | 3.063869214 |
| SNORA30 | 0.018081451 | 3.060681599 |
| RP11-211A18.2 | 0.000774788 | 3.060401495 |
| RP11-142I2.1 | 0.06066333 | 3.059128201 |
| MYL12A | 0.004408586 | 1.312153382 |
| AP004550.1 | 0.099439669 | 3.051685737 |
| PDCL3P7 | 0.092569564 | 3.050913485 |
| RPL35AP25 | 0.030115386 | 3.043303211 |
| GRAMD3 | 0.004430287 | 1.323628654 |
| MICC | 0.002125894 | 3.042887753 |
| RP11-289H16.1 | 0.100957119 | 3.040333316 |
| MIR128-1 | 0.067738361 | 3.039840229 |
| MAPT | 0.00444734 | 0.357911272 |
| TTLL10-AS1 | 0.096541063 | 3.039007583 |
| CDY7P | 0.134057048 | 3.038419909 |
| ZNF621 | 0.0044507 | 1.262629259 |
| CHDH | 0.004456294 | 1.594036788 |
| RP1-305G21.1 | 0.038484326 | 3.031530866 |
| RP11-293M10.1 | 0.012017308 | 3.030586375 |
| ATP6V1G1P6 | 0.051962524 | 3.030169038 |
| RP11-43D2.2 | 0.039187768 | 3.030001899 |
| RP11-838N2.5 | 0.009154731 | 3.029493103 |
| RPSAP64 | 0.032413408 | 3.020582806 |
| RP11-732A19.6 | 0.092371603 | 3.019792725 |
| CTD-2210P24.3 | 0.130055504 | 3.0182416 |
| TGFB2 | 0.004475782 | 2.195455724 |
| CTD-2552K11.2 | 0.027756729 | 3.017478758 |
| DISC1-IT1 | 0.011942361 | 3.017322723 |
| RP4-539M6.14 | 0.089765701 | 3.015492676 |
| RP11-38J22.2 | 0.017747376 | 3.013819641 |
| RP3-334F4.2 | 0.019721019 | 3.013676391 |
| RP11-697K23.3 | 0.043602581 | 3.013372928 |
| RP11-687M24.7 | 0.102655106 | 3.01217025 |
| FAHD2P1 | 0.060152816 | 3.012025654 |
| CTD-2335O3.3 | 0.101691195 | 3.010984149 |
| LINC00371 | 0.007092691 | 3.007484639 |
| HN1L | 0.004489395 | 1.184497933 |
| HOXC6 | 0.004501669 | 1.564284441 |
| RP11-813F20.2 | 0.090065937 | 3.006151651 |
| RN7SL278P | 0.114542081 | 3.004614131 |
| CC2D2A | 0.004516125 | 1.515203467 |
| RP11-413P11.1 | 0.080832725 | 3.000682893 |
| SPA17 | 0.004521857 | 1.498748943 |
| RSPH6A | 0.004571737 | 2.165466233 |
| RNU6-1088P | 0.092703195 | 2.994186683 |
| IGHVII-22-1 | 0.13631447 | 2.99321111 |
| SLC25A30 | 0.004575186 | 1.303906974 |
| CDKAL1 | 0.004586958 | 1.197211868 |
| RP4-676L2.1 | 0.003365207 | 2.982009598 |
| SYAP1 | 0.004626631 | 1.181412755 |
| LINC01486 | 0.005389189 | 2.976920606 |
| RIMKLB | 0.004651812 | 1.635523998 |
| RNU6-314P | 0.029399489 | 2.976527952 |
| CPA5 | 0.004658602 | 1.787252426 |
| RP13-7D7.1 | 0.103082885 | 2.970454764 |
| RP11-379C10.1 | 0.130847562 | 2.969997835 |
| SETP7 | 0.005784412 | 2.968712793 |
| LINC01346 | 0.018991461 | 2.968344537 |
| RNA5SP68 | 0.143439072 | 2.965953177 |
| RNU1-94P | 0.034725759 | 2.965771477 |
| SLC16A4 | 0.004660262 | 2.83552447 |
| TOMM70A | 0.004678728 | 0.846806478 |
| RP11-375N15.2 | 0.111820299 | 2.961863685 |
| LINC00621 | 0.056726886 | 2.960908936 |
| RP11-444D13.1 | 0.084330433 | 2.960121219 |
| RN7SKP253 | 0.062922638 | 2.95750558 |
| MIR1293 | 0.07216901 | 2.957307591 |
| CTD-2542C24.3 | 0.008153225 | 2.954564122 |
| AC133106.2 | 0.043535821 | 2.953633562 |
| RP11-53M11.5 | 0.128759748 | 2.95068519 |
| XX-C2158C12.2 | 0.082404237 | 2.947480731 |
| AC018804.7 | 0.025429175 | 2.947435507 |
| AC006445.6 | 0.002229357 | 2.947435044 |
| RP11-40E6.1 | 0.099029385 | 2.945935221 |
| GPR111 | 0.004707516 | 0.477230277 |
| DAG1 | 0.004713329 | 1.236073435 |
| SPPL3 | 0.004724556 | 0.810476486 |
| ANKRD42 | 0.004739095 | 1.32176758 |
| RPL7P53 | 0.079859107 | 2.933954043 |
| AC090453.1 | 0.090894761 | 2.932822654 |
| AC011239.1 | 0.000760049 | 2.932605136 |
| RNA5SP172 | 0.129463055 | 2.931823395 |
| SMIM14 | 0.004741435 | 1.358701415 |
| RP11-164O23.8 | 0.027534988 | 2.931012949 |
| CYP2B7P | 0.026907857 | 2.930185421 |
| AC008427.2 | 0.010299878 | 2.928267918 |
| SLC25A47P1 | 0.063702868 | 2.925035672 |
| CTD-2509G16.1 | 0.066162377 | 2.923797466 |
| RP1-169K13.3 | 0.007087678 | 2.920780777 |
| ENPP7P11 | 0.013334902 | 2.920343688 |
| RP11-100L22.1 | 0.012118842 | 2.919942584 |
| DIRC3-AS1 | 0.046370542 | 2.919411586 |
| ZFYVE27 | 0.00476206 | 1.196499683 |
| SNRPEP7 | 0.111605105 | 2.917029935 |
| MIR7515HG | 0.08129907 | 2.914030433 |
| MIR5003 | 0.054944608 | 2.906928996 |
| RP1-172N19.4 | 0.053932356 | 2.904759307 |
| RP11-365H8.2 | 0.098963592 | 2.904602257 |
| RP11-59N23.1 | 0.023647926 | 2.903214275 |
| ARMCX3 | 0.004782921 | 1.341609389 |
| RP4-806M20.5 | 0.107554944 | 2.898554545 |
| RP11-393I23.4 | 0.130993449 | 2.898214095 |
| RP11-179H18.8 | 0.140712946 | 2.896299413 |
| DEPTOR | 0.004793004 | 2.254614269 |
| MIR6746 | 0.002139673 | 2.89400268 |
| LINC01513 | 0.063553275 | 2.893884052 |
| CTC-508F8.1 | 0.034152071 | 2.890399097 |
| CDC42EP3P1 | 0.020697854 | 2.888018592 |
| CNGB1 | 0.004799429 | 3.66110731 |
| RP1-186E20.1 | 0.114389705 | 2.887399176 |
| RIBC1 | 0.0048022 | 1.900658637 |
| MIR608 | 0.099245658 | 2.885254306 |
| TESK1 | 0.004816699 | 1.159965745 |
| CFAP54 | 0.00481942 | 2.660174115 |
| RNY3P14 | 0.039030109 | 2.883923026 |
| RPL39P26 | 0.020415054 | 2.881986716 |
| CTD-2189E23.2 | 0.08411035 | 2.88115113 |
| TFAP2C | 0.004844242 | 0.719090921 |
| AC073127.1 | 0.086819917 | 2.871645597 |
| STK11 | 0.004859924 | 0.846235798 |
| AC104794.4 | 0.027019517 | 2.870076697 |
| SAR1AP4 | 0.077913509 | 2.868157112 |
| AC079779.5 | 0.053458789 | 2.866139395 |
| AC096664.2 | 0.07187519 | 2.865917794 |
| RNU6-899P | 0.075728597 | 2.865557176 |
| Metazoa_SRP | 0.128560571 | 2.864166685 |
| RP3-429O6.1 | 0.047662639 | 2.863723294 |
| SLC6A1-AS1 | 0.016984005 | 2.863118436 |
| RP11-254I22.3 | 0.014725925 | 2.862413351 |
| GS1-531I17.3 | 0.024903074 | 2.860748291 |
| RP11-316J7.4 | 0.002911064 | 2.858465497 |
| MIR1203 | 0.057752068 | 2.857571697 |
| RP11-269C23.5 | 0.039686105 | 2.85694153 |
| CTD-2651B20.1 | 0.000793493 | 2.856812589 |
| RP11-122F24.1 | 0.099791063 | 2.855872947 |
| RP11-930P14.1 | 0.050492575 | 2.85133238 |
| PCDH7 | 0.004863647 | 1.653312773 |
| RP11-244N9.6 | 0.017068832 | 2.84933312 |
| LINC01093 | 0.023028632 | 2.848925245 |
| RP11-122A21.2 | 0.057681052 | 2.848356279 |
| RP11-129I19.2 | 0.10971934 | 2.847923439 |
| RP11-410N8.3 | 0.135957552 | 2.847447177 |
| GOLGA2P9 | 0.046428165 | 2.847225636 |
| RP11-57A19.4 | 0.036012228 | 2.847217329 |
| RP11-157L3.12 | 0.031601362 | 2.845068293 |
| WBP5 | 0.004881643 | 1.315467694 |
| RP11-349G13.2 | 0.001561366 | 2.843563548 |
| RP11-13K12.5 | 0.096265391 | 2.843131987 |
| SERPINA1 | 0.004887797 | 1.99207122 |
| MIR4692 | 0.114371981 | 2.841748365 |
| FAM167A-AS1 | 0.120858026 | 2.841548126 |
| RP11-38J22.3 | 0.077254653 | 2.837660914 |
| UMODL1-AS1 | 0.093154597 | 2.837546609 |
| RP11-260O18.1 | 0.110190171 | 2.836929326 |
| RP1-32I10.10 | 0.144149919 | 2.835754591 |
| HNRNPH3 | 0.004888783 | 1.125248734 |
| OR13Z2P | 0.040241182 | 2.833192478 |
| RP11-317L10.1 | 0.104859511 | 2.832735687 |
| RP11-567M21.3 | 0.003307249 | 2.832308726 |
| RP11-2N5.2 | 0.069180313 | 2.831273302 |
| WDR63 | 0.004889693 | 2.404001061 |
| RNU1-108P | 0.015714849 | 2.829951976 |
| RN7SL623P | 0.140464147 | 2.826856003 |
| ERI1 | 0.004893474 | 1.198353669 |
| ERBB2IP | 0.004926312 | 1.183053842 |
| CEACAMP9 | 0.04123895 | 2.822805802 |
| DSG3 | 0.00493207 | 0.566808661 |
| RP11-168L22.2 | 0.042046605 | 2.821262467 |
| CYCSP51 | 0.014000953 | 2.81653789 |
| HERC4 | 0.004940096 | 1.173021938 |
| RP11-109D24.1 | 0.136603942 | 2.815619596 |
| C4orf29 | 0.004944866 | 1.213019662 |
| PNISR | 0.004976133 | 1.255232366 |
| RBM12B | 0.004990088 | 1.220650741 |
| RP11-319E12.2 | 0.094078298 | 2.810460568 |
| AC090627.1 | 0.135172546 | 2.808654597 |
| AL109947.2 | 0.06307394 | 2.807694191 |
| C5orf63 | 0.004995489 | 1.319247266 |
| C10orf67 | 0.005015286 | 2.269527966 |
| LINC01565 | 0.059005837 | 2.803309281 |
| WT1-AS_2 | 0.016712741 | 2.802678875 |
| IGLV3-22 | 0.02116544 | 2.801568286 |
| LZTS1-AS1 | 0.099759758 | 2.801113414 |
| RP11-122C21.1 | 0.054422231 | 2.801100399 |
| ERP27 | 0.005037345 | 2.451193536 |
| ALDH1L1 | 0.00504585 | 2.343088246 |
| RP11-49I11.2 | 0.025546613 | 2.798468495 |
| AC092071.1 | 0.079642626 | 2.797554837 |
| BMS1P16 | 0.027448915 | 2.796997234 |
| snoU2-30 | 0.100450293 | 2.796027128 |
| RIIAD1 | 0.00504682 | 4.236680073 |
| CCP110 | 0.005048636 | 1.245501674 |
| TMEM181 | 0.005060255 | 1.223186785 |
| RRM2P3 | 0.000109192 | 2.793986317 |
| RP11-439L8.5 | 0.01080044 | 2.792940825 |
| ROCK2 | 0.00506052 | 1.210319026 |
| AC000111.6 | 0.130509831 | 2.79217023 |
| STT3B | 0.005074904 | 1.207584427 |
| SNORA64 | 0.074150481 | 2.790676283 |
| RP3-521E19.2 | 0.087272168 | 2.789424475 |
| RP1-63G5.5 | 0.047777228 | 2.789228152 |
| RNU6-1327P | 0.015701834 | 2.786236248 |
| GMPR | 0.005081183 | 2.035106313 |
| CEP41 | 0.005104407 | 1.354361508 |
| RP11-19J5.1 | 0.067186662 | 2.783502231 |
| RNU6-1187P | 0.0855076 | 2.778392092 |
| RP5-1173A5.1 | 0.021019771 | 2.778361675 |
| RP5-894D12.5 | 0.020544795 | 2.777275396 |
| AC018890.6 | 0.12707481 | 2.775560582 |
| RP11-356M20.1 | 0.04404321 | 2.774811755 |
| RN7SKP51 | 0.113028721 | 2.773620512 |
| RNA5SP252 | 0.118470874 | 2.772042265 |
| PURA | 0.005111294 | 1.163081094 |
| HNRNPA1P31 | 0.133118728 | 2.771803702 |
| REN | 0.005124985 | 3.207525161 |
| RP11-66C24.1 | 0.05514685 | 2.767223056 |
| Y_RNA | 0.006653503 | 2.7653106 |
| RN7SL498P | 0.108103689 | 2.76394067 |
| SLC30A9 | 0.005131226 | 1.193610533 |
| LINC00890 | 0.093366288 | 2.762743035 |
| BMS1P15 | 0.133383762 | 2.761892775 |
| MAPK1IP1L | 0.005146512 | 1.126483904 |
| RP11-1281K21.1 | 0.079124865 | 2.760556734 |
| KCNQ1 | 0.005161846 | 1.703697897 |
| AC093627.12 | 0.112301997 | 2.759719101 |
| FBLIM1 | 0.005260743 | 1.377378299 |
| RPS2P39 | 0.048101116 | 2.757035017 |
| RP11-422N16.3 | 0.007362346 | 2.756511659 |
| RBBP8 | 0.005265357 | 1.241873746 |
| ENC1 | 0.005267262 | 1.551860038 |
| LAD1 | 0.005275438 | 0.766844544 |
| AC006548.28 | 0.048708668 | 2.753027169 |
| RP11-326C3.10 | 0.075979223 | 2.75111067 |
| AP001628.7 | 0.097323097 | 2.746906988 |
| HSPA8P18 | 0.000297555 | 2.746856833 |
| TMSB4X | 0.005292781 | 1.246714538 |
| RN7SL799P | 0.051284825 | 2.746371254 |
| SPTLC1P4 | 0.097761606 | 2.745767213 |
| BASP1P1 | 0.134008955 | 2.745476411 |
| ERGIC1 | 0.005304922 | 1.390906536 |
| FUNDC2 | 0.005306937 | 1.219451412 |
| CTD-2375G15.1 | 5.31E-05 | 2.742316627 |
| AC092066.6 | 0.009231967 | 2.742306567 |
| AC078864.2 | 0.074169355 | 2.74150893 |
| RN7SL111P | 0.034495948 | 2.741163237 |
| RP13-497K6.1 | 0.100667792 | 2.740886086 |
| RP11-1143G9.5 | 0.078258401 | 2.737392409 |
| RP4-581O6.1 | 0.049274755 | 2.736805137 |
| CCDC113 | 0.005311044 | 1.714447058 |
| RNA5SP207 | 0.018108949 | 2.736560285 |
| RNU6-662P | 0.137338426 | 2.736125575 |
| OR2S1P | 0.150994892 | 2.735941329 |
| RP11-255I10.1 | 0.036542019 | 2.735547903 |
| AC064834.1 | 0.117018431 | 2.734385635 |
| ARMS2 | 0.005328199 | 1.784413915 |
| RP11-648K4.2 | 0.098709049 | 2.732226152 |
| ANAPC16 | 0.005339466 | 1.146435645 |
| PAM | 0.005341367 | 1.369803836 |
| AC116609.1 | 0.014496986 | 2.727074419 |
| BBS7 | 0.00534493 | 1.156968899 |
| CTD-2311M21.3 | 0.005336189 | 2.726362275 |
| RP11-675F6.3 | 0.150479249 | 2.726287723 |
| MIR3939 | 0.035754078 | 2.725912318 |
| RP11-739G5.1 | 0.089972016 | 2.725223238 |
| SLC35G4 | 0.14760276 | 2.722565335 |
| RP4-799P18.2 | 0.059258611 | 2.721466468 |
| SYTL5 | 0.005364094 | 0.355747364 |
| RP11-713M15.2 | 0.106159272 | 2.713974715 |
| RP11-66B24.5 | 0.004235961 | 2.710843034 |
| RP11-320M16.2 | 0.007458391 | 2.709472047 |
| RP11-95M5.1 | 0.075599795 | 2.707990878 |
| RP11-451G4.3 | 0.030245264 | 2.707254557 |
| IGHVIII-76-1 | 0.016447084 | 2.706886385 |
| RNU1-91P | 0.075867411 | 2.70675346 |
| IP6K2 | 0.005414259 | 1.195769955 |
| YWHAEP7 | 0.011638142 | 2.704081509 |
| FER1L5 | 0.005435299 | 2.269505661 |
| VTRNA2-2P | 0.061245713 | 2.701684512 |
| RP11-379K22.3 | 0.035306278 | 2.697281211 |
| MIR5588 | 0.137450875 | 2.696731312 |
| RN7SL404P | 0.086270865 | 2.695869139 |
| AC116562.1 | 0.074304238 | 2.694742135 |
| RP11-62I21.1 | 0.030853822 | 2.692701104 |
| RP11-728F11.4 | 0.001549616 | 2.690119747 |
| RP1-111D6.2 | 0.104440017 | 2.68893731 |
| FAM81A | 0.005456227 | 1.565162519 |
| LSAMP-AS1 | 0.094498541 | 2.687020419 |
| WRBP1 | 0.046086473 | 2.686311605 |
| ARG2 | 0.005460788 | 1.49244207 |
| RN7SKP153 | 0.147133225 | 2.682942383 |
| CDC14A | 0.005483863 | 1.38284065 |
| SLC25A15P3 | 0.067084805 | 2.682818257 |
| MIR34C | 0.135975737 | 2.678330425 |
| AC021218.2 | 0.090443978 | 2.677403435 |
| TRAJ29 | 0.115881115 | 2.676369763 |
| AC140542.2 | 0.020486219 | 2.67631433 |
| RP11-575L7.2 | 0.000187249 | 2.675569362 |
| RP11-715G15.2 | 0.074508818 | 2.674942649 |
| AJ003147.8 | 0.004264022 | 2.673623774 |
| RP11-1072N2.2 | 0.131319613 | 2.668548103 |
| CICP6 | 0.130292578 | 2.668340199 |
| RP11-301N24.3 | 0.066237807 | 2.667175017 |
| RP11-168K11.3 | 0.004932782 | 2.664992811 |
| RP11-281N10.1 | 0.041100062 | 2.663145496 |
| LINC01091 | 0.010074931 | 2.662808032 |
| BBIP1 | 0.005513587 | 1.170832873 |
| RP11-1376P16.1 | 0.000144963 | 2.659871957 |
| RP11-218C23.1 | 0.136583015 | 2.659061433 |
| RP11-206P5.2 | 0.060535358 | 2.657820458 |
| LA16c-329F2.1 | 0.097179573 | 2.656279444 |
| LCN12 | 0.0055488 | 2.299073235 |
| LINC01558 | 0.009801205 | 2.654509993 |
| AC011747.6 | 0.051678324 | 2.65406759 |
| RP11-138H11.1 | 0.125021209 | 2.651365889 |
| RP4-665N4.4 | 0.001575369 | 2.65117539 |
| NPAP1P2 | 0.076992373 | 2.648812685 |
| MIR1256 | 0.066843273 | 2.647414765 |
| CTA-363E6.3 | 0.009240155 | 2.644951656 |
| SOWAHA | 0.005549072 | 2.601914765 |
| RP11-92B11.4 | 0.128141106 | 2.644198911 |
| RP11-798K3.4 | 0.071692552 | 2.644137846 |
| WDR78 | 0.005576741 | 1.931484273 |
| Y_RNA | 0.123726562 | 2.642430647 |
| TTC26 | 0.005621132 | 1.270491589 |
| RP11-607P23.1 | 0.034832761 | 2.641467238 |
| RP11-28P17.3 | 0.060895661 | 2.641284258 |
| PGM5-AS1 | 0.09606374 | 2.640547683 |
| RN7SKP35 | 0.092553343 | 2.640216288 |
| RP11-1081M5.1 | 0.081063524 | 2.637079378 |
| U1 | 0.028902656 | 2.636260276 |
| LINC01198 | 0.137971233 | 2.635476646 |
| RP11-685A21.1 | 0.110899598 | 2.635267915 |
| RP11-234B24.5 | 0.08996151 | 2.634493712 |
| CTD-2138O14.1 | 0.112747503 | 2.634170325 |
| RP11-34H11.3 | 0.126149765 | 2.633913019 |
| RP4-745E8.2 | 0.04491639 | 2.63254422 |
| PERP | 0.005640872 | 0.689985367 |
| SCARNA21 | 0.115649829 | 2.631788327 |
| Six3os1_5 | 0.065218363 | 2.631400348 |
| AC012354.6 | 0.054350251 | 2.631167368 |
| RP11-169N13.4 | 0.113450855 | 2.629377308 |
| RP11-675F6.4 | 0.093789134 | 2.629293436 |
| OR9A3P | 0.021524499 | 2.62919987 |
| RP11-1102P16.1 | 0.048066303 | 2.628750445 |
| RP11-66N24.6 | 0.051097693 | 2.628539888 |
| HDHD1P2 | 0.092797782 | 2.627744681 |
| RP11-844P9.2 | 0.040220836 | 2.624922268 |
| RP11-732M18.4 | 0.008117941 | 2.624164482 |
| RP11-357P18.2 | 0.003183774 | 2.624119721 |
| AC068538.2 | 0.076091264 | 2.623591048 |
| BLOC1S5 | 0.005652697 | 1.180309114 |
| GS1-531I17.2 | 0.092450528 | 2.621165719 |
| AC073621.2 | 0.069522933 | 2.620497664 |
| RP11-81H3.2 | 0.144642499 | 2.617353408 |
| HIGD1AP15 | 0.131098131 | 2.614867592 |
| GNL2P1 | 0.141963635 | 2.614800623 |
| LINC00282 | 0.029656698 | 2.613974569 |
| SEC22C | 0.005655412 | 1.207188514 |
| RP11-349F21.3 | 0.05678132 | 2.613605304 |
| NRON | 0.138911872 | 2.613514968 |
| RP11-13K12.1 | 0.124145714 | 2.612680424 |
| RP11-451N19.3 | 0.103340018 | 2.611794748 |
| RP11-179A7.2 | 0.069381277 | 2.611380397 |
| OR5E1P | 0.025877149 | 2.611354185 |
| CCDC122 | 0.005669304 | 1.276884436 |
| AC104131.1 | 0.149409607 | 2.610271404 |
| LCN2 | 0.005673421 | 2.343471615 |
| FAM231D | 0.005678169 | 2.998562642 |
| DRC1 | 0.005679212 | 3.970117689 |
| LINC00415 | 0.021598837 | 2.608033986 |
| RP11-708L7.6 | 0.003989907 | 2.607043909 |
| THAP10 | 0.005681592 | 1.252828375 |
| EWSAT1 | 0.005472049 | 2.604053468 |
| DCAF16 | 0.005684478 | 1.218737109 |
| RP3-388M5.8 | 0.025542418 | 2.603080962 |
| KRT86 | 0.005699983 | 1.765863295 |
| LINC01036 | 0.086980535 | 2.599707581 |
| LINC01343 | 0.078969027 | 2.599345174 |
| AC012506.3 | 0.131502433 | 2.599234767 |
| MED24 | 0.005711814 | 1.160766659 |
| RP11-354K1.1 | 0.045358695 | 2.597571551 |
| SNORA3 | 0.089193895 | 2.595719201 |
| CGGBP1 | 0.005726806 | 1.165625851 |
| AC092566.1 | 0.045156526 | 2.59456603 |
| RP11-270B14.1 | 0.04377141 | 2.594151672 |
| PTTG1IP | 0.00573486 | 1.22902905 |
| RP11-445L6.3 | 0.009176291 | 2.590562987 |
| RP11-214D15.2 | 0.024590662 | 2.588976917 |
| RNU6-1282P | 0.089526688 | 2.588271416 |
| RP11-494I9.2 | 0.142836488 | 2.588188118 |
| OXSM | 0.005765776 | 1.177309279 |
| MCTP2 | 0.005772189 | 1.402322023 |
| TBX6 | 0.005782875 | 0.668610694 |
| RP1-34H18.1 | 0.020412638 | 2.582711389 |
| NXF3 | 0.005791828 | 6.55606862 |
| RP11-290H9.5 | 0.007101948 | 2.579577734 |
| CYP21A2 | 0.005861499 | 2.539705183 |
| ENPP4 | 0.005872478 | 1.791868264 |
| CHMP1B | 0.005874901 | 1.225719652 |
| RPL23AP50 | 0.004315828 | 2.578094945 |
| NME6 | 0.005877269 | 1.152683886 |
| C11orf16 | 0.005893881 | 3.354203584 |
| AC004538.3 | 0.059545784 | 2.576293101 |
| RP13-347D8.7 | 0.102801624 | 2.572452885 |
| RP11-627G23.1 | 0.08707373 | 2.570928303 |
| MIR550A3 | 0.134206634 | 2.570624113 |
| RP11-302F12.1 | 0.034514213 | 2.568174257 |
| AC112497.2 | 0.015066354 | 2.566844039 |
| AC133141.1 | 0.012701414 | 2.565607296 |
| HMGB3P30 | 0.085135489 | 2.564867614 |
| ATP5F1P5 | 0.000651081 | 2.564614204 |
| RP11-678G15.2 | 0.145166777 | 2.564454147 |
| AC006116.21 | 0.120110568 | 2.562798763 |
| WDR6 | 0.005896541 | 1.24121606 |
| P4HTM | 0.005904457 | 1.3041107 |
| SERPINI2 | 0.005905374 | 3.83875119 |
| RP11-344N17.9 | 0.145623839 | 2.560209646 |
| RP11-436K8.1 | 0.1327399 | 2.557287245 |
| MIR646 | 0.130447033 | 2.553213103 |
| TAF6L | 0.005917303 | 0.873385245 |
| CH17-78J1.1 | 0.032671868 | 2.550758953 |
| LINC01189 | 0.145211447 | 2.548200259 |
| AC068858.1 | 0.14759974 | 2.547760146 |
| RP11-213G6.2 | 0.103671295 | 2.547099532 |
| HRH1 | 0.005949086 | 1.432698851 |
| U3 | 0.044804723 | 2.544693247 |
| LINC01182 | 0.109539164 | 2.544377348 |
| RNU6-106P | 0.032110928 | 2.544035752 |
| RP11-13A1.3 | 0.018169159 | 2.543744591 |
| RP1-229K20.5 | 0.070021036 | 2.542022152 |
| PLCH1-AS1 | 0.035726671 | 2.54119767 |
| OSGIN2 | 0.005959451 | 1.196123702 |
| NEK5 | 0.005977297 | 2.213507632 |
| RP11-58A18.2 | 0.104051473 | 2.539253962 |
| RN7SL25P | 0.058066366 | 2.539209796 |
| AC016768.1 | 0.07076808 | 2.53862624 |
| KRT18P26 | 0.002180243 | 2.537789296 |
| RNU6-589P | 0.089577291 | 2.537740767 |
| CTC-482H14.5 | 0.059948065 | 2.536669534 |
| SH3BGRL | 0.006003123 | 1.336278061 |
| MIR100HG | 0.019833547 | 2.535721316 |
| CTD-2515H24.3 | 0.041316006 | 2.53347757 |
| RPL6P12 | 0.034356276 | 2.533022438 |
| CBX8 | 0.006003887 | 1.258291881 |
| SLC10A5P1 | 0.130822508 | 2.531165251 |
| PPP1R2P2 | 0.142866544 | 2.52919441 |
| AP000344.3 | 0.095865726 | 2.528722773 |
| RP11-115J23.1 | 0.047900906 | 2.527899265 |
| GLRX | 0.006037823 | 1.460334209 |
| PACRG-AS1 | 0.07043914 | 2.527387579 |
| SHANK2-AS3 | 0.099592213 | 2.527260733 |
| AL133244.1 | 0.105066079 | 2.525746329 |
| KIAA1244 | 0.006042091 | 1.90795128 |
| LINC00601 | 0.050099742 | 2.523967577 |
| LINC00303 | 0.048761438 | 2.522780233 |
| TFRC | 0.006057483 | 0.659438355 |
| ZNF852 | 0.006104877 | 1.303149566 |
| CXADRP2 | 0.122754952 | 2.520156263 |
| ANAPC4 | 0.006106546 | 1.164671133 |
| RP1-232L24.3 | 0.016820067 | 2.519099811 |
| RP1-229K20.9 | 0.096619434 | 2.519037349 |
| RPL35AP | 0.058785688 | 2.516323826 |
| NANOGP7 | 0.035131351 | 2.514838958 |
| RP11-469N6.1 | 0.062707053 | 2.513880562 |
| RP11-605F22.2 | 0.144928138 | 2.512736474 |
| RP11-763F8.1 | 0.147022034 | 2.511855612 |
| CTD-2351A16.1 | 0.054735452 | 2.510716042 |
| AL590226.1 | 0.087546281 | 2.509782055 |
| TPT1P14 | 0.009474378 | 2.509689776 |
| RP11-434H6.2 | 0.028371762 | 2.509255736 |
| CTD-2034I21.2 | 0.008858814 | 2.508924212 |
| RP11-418J17.3 | 0.119220104 | 2.508733723 |
| Six3os1_1 | 0.065025919 | 2.508435558 |
| PCSK1 | 0.006112893 | 3.420262537 |
| MKRN2OS | 0.006138136 | 1.367652624 |
| RP11-554E23.2 | 0.004170396 | 2.507437513 |
| CFAP36 | 0.006151481 | 1.261168058 |
| TESC | 0.006163891 | 6.885162932 |
| RP11-526N18.1 | 0.126659489 | 2.502978555 |
| CTD-2595P9.4 | 0.005815321 | 2.502803467 |
| RN7SL140P | 0.108590703 | 2.501364293 |
| RP11-478J18.2 | 0.141980056 | 2.501017226 |
| RP11-739N10.1 | 0.089475172 | 2.500009233 |
| ARHGEF3 | 0.006226726 | 1.232155522 |
| RP11-3P22.2 | 0.119932541 | 2.498275685 |
| SLC12A7 | 0.006247979 | 1.583477315 |
| HMGN1P17 | 0.07081478 | 2.496627094 |
| AC022173.2 | 0.059429157 | 2.494811011 |
| LINC01317 | 0.017433756 | 2.493934456 |
| SNORD105B | 0.073003758 | 2.493293273 |
| TSPAN31 | 0.006249062 | 1.157606599 |
| RP11-775B15.2 | 0.105514875 | 2.493152726 |
| RP11-439K3.1 | 0.000324044 | 2.491991755 |
| RN7SKP57 | 0.07677291 | 2.489053662 |
| CTBP1 | 0.006276228 | 1.149698539 |
| SNRPGP17 | 0.006461469 | 2.486155016 |
| RP11-138B4.1 | 0.117745822 | 2.481891269 |
| PPARGC1A | 0.006284164 | 2.884290773 |
| AC002064.4 | 0.003067308 | 2.479789252 |
| Y_RNA | 0.072909559 | 2.479034131 |
| CASC6 | 0.140101694 | 2.477070212 |
| TCEB2P4 | 0.141481135 | 2.476470171 |
| RP11-76C10.3 | 0.017604831 | 2.475777723 |
| SOX4 | 0.006297045 | 1.381654933 |
| RP11-117L5.1 | 0.005724224 | 2.473911741 |
| RP11-486A14.1 | 0.029104323 | 2.472818597 |
| FKBP1AP3 | 0.074837128 | 2.472288498 |
| MAFB | 0.006324036 | 0.670325824 |
| CRELD1 | 0.006331576 | 1.27212378 |
| TRDC | 0.041089916 | 2.468659587 |
| RP11-876N24.1 | 0.034570292 | 2.467396972 |
| KIAA0141 | 0.006332145 | 1.164891736 |
| CRIM1 | 0.00633498 | 1.580014265 |
| IFNL3P1 | 0.003880812 | 2.465068075 |
| CYCSP27 | 0.100477333 | 2.464777898 |
| RP11-432J24.3 | 0.029839374 | 2.464271254 |
| PDIA3 | 0.006335125 | 1.161846722 |
| KRT17P4 | 0.020142942 | 2.463312252 |
| CDK5PS | 0.082329249 | 2.463007311 |
| LINC01219 | 0.084709238 | 2.460963261 |
| RP11-269G24.8 | 0.025425102 | 2.459937746 |
| RP11-469A15.2 | 0.010464564 | 2.459834601 |
| RP11-213H15.1 | 0.00168567 | 2.459176935 |
| SPATS2 | 0.006340035 | 1.191976988 |
| CD276 | 0.006357179 | 1.309416051 |
| RP11-757G1.6 | 0.007968313 | 2.453248613 |
| CYP39A1 | 0.00635768 | 1.498346927 |
| AC012454.4 | 0.024406131 | 2.451915128 |
| SECISBP2L | 0.006364142 | 1.225201311 |
| STEAP2 | 0.006365143 | 1.506022514 |
| CFAP221 | 0.00637149 | 3.202478475 |
| RNA5SP53 | 0.114867647 | 2.446954444 |
| RN7SL141P | 0.027353613 | 2.446108179 |
| WARSP1 | 0.068628165 | 2.445801252 |
| RP11-168L7.3 | 0.093693489 | 2.444530995 |
| CTBP2P3 | 0.11266609 | 2.443481037 |
| CTD-3051D23.3 | 0.057800941 | 2.442521837 |
| KRASP1 | 0.115289519 | 2.442500798 |
| LL0XNC01-250H12.3 | 0.105116819 | 2.442444016 |
| MRPS15P1 | 0.05558214 | 2.441188776 |
| CTD-2587H24.5 | 0.004288775 | 2.441163074 |
| POU6F1 | 0.006432751 | 1.284093428 |
| SNORA70 | 0.131032252 | 2.44069469 |
| C1QTNF6 | 0.006443242 | 1.527255976 |
| RP11-143E21.6 | 0.026740721 | 2.440236155 |
| RP11-491H19.1 | 0.025219107 | 2.440204188 |
| RP11-399K21.5 | 0.052643664 | 2.439585311 |
| OR7E99P | 0.035422083 | 2.43907338 |
| VWA3B | 0.006448649 | 2.504501064 |
| RP11-550I15.1 | 0.107522724 | 2.437759104 |
| RP11-59E19.3 | 0.140148613 | 2.437709391 |
| LINC00927 | 0.11381735 | 2.436178652 |
| RP11-486F17.1 | 0.028123598 | 2.436132121 |
| PPP1CB | 0.006489274 | 1.186683654 |
| GPR125 | 0.006503114 | 1.317683488 |
| DLGAP1-AS4 | 0.074085848 | 2.432891168 |
| SNORA81 | 0.061535949 | 2.432403948 |
| RP5-1066H13.4 | 0.037881905 | 2.430734873 |
| RP11-760H22.2 | 0.002679917 | 2.430651106 |
| GPAA1P2 | 0.03877665 | 2.429181813 |
| PIP5K1B | 0.006510686 | 2.084691195 |
| ART2P | 0.067972264 | 2.428848941 |
| RN7SL413P | 0.052609735 | 2.426188938 |
| BZRAP1 | 0.006519018 | 1.633617771 |
| EXOSC8 | 0.006537146 | 1.180065944 |
| AL021918.2 | 0.016368538 | 2.423493705 |
| RPL37P4 | 0.126699404 | 2.423221264 |
| RNU6-608P | 0.118220114 | 2.4230047 |
| Y_RNA | 0.122225309 | 2.422905108 |
| MORF4L2 | 0.00654432 | 1.153087638 |
| RP11-703M24.5 | 0.06712466 | 2.42264297 |
| RN7SL391P | 0.036394877 | 2.42141269 |
| GMPPB | 0.006549843 | 1.220593646 |
| ALDH18A1 | 0.006552857 | 1.212911343 |
| MRFAP1L1 | 0.006571804 | 1.170835478 |
| Y_RNA | 0.092462583 | 2.419709047 |
| CTD-2143L24.1 | 0.042443885 | 2.417096988 |
| RN7SL718P | 0.08176023 | 2.41585817 |
| LRRC24 | 0.006590591 | 0.31724783 |
| LINC01115 | 0.139274554 | 2.412923106 |
| SLC41A2 | 0.00661318 | 1.547955545 |
| RNA5SP330 | 0.03785882 | 2.411503362 |
| RP11-446F17.3 | 0.009517615 | 2.410836742 |
| RPL37P25 | 0.05279263 | 2.410548791 |
| RP11-98D18.7 | 0.055551689 | 2.410052289 |
| SYCP2L | 0.006615146 | 2.803328728 |
| METTL15 | 0.006688559 | 1.178607294 |
| SPECC1 | 0.006693242 | 1.399006651 |
| AC116614.1 | 0.10560855 | 2.407211363 |
| ALG1L5P | 0.009724646 | 2.407158199 |
| GTF2IRD2B | 0.006713642 | 1.252819125 |
| RIMBP2 | 0.006769281 | 3.25471348 |
| RNU7-160P | 0.028946495 | 2.403298839 |
| SYNE1-AS1 | 0.085795804 | 2.402071632 |
| TCTN1 | 0.00681444 | 1.326102159 |
| LRRCC1 | 0.006875339 | 1.238575975 |
| AC017006.2 | 0.004766412 | 2.399495772 |
| CAPSL | 0.006922032 | 6.863393786 |
| Six3os1_2 | 0.104881745 | 2.398312932 |
| RNU6-406P | 0.054549495 | 2.39826871 |
| PPIAP20 | 0.002197364 | 2.398173891 |
| RP11-49I11.3 | 0.003560716 | 2.397687385 |
| FAM170B-AS1 | 0.101617045 | 2.396805959 |
| KIRREL3-AS1 | 0.056761284 | 2.396216352 |
| KRT87P | 0.002942463 | 2.396074744 |
| AF241726.2 | 0.119016516 | 2.394743196 |
| RP1-290I10.2 | 0.150234113 | 2.393682555 |
| MIR559 | 0.002523213 | 2.39345599 |
| ABHD17A | 0.006923156 | 0.867377931 |
| NFKBIB | 0.006963487 | 0.80545666 |
| AHI1 | 0.007013336 | 1.250794648 |
| RP13-143G15.4 | 0.013820491 | 2.385887506 |
| LINC00524 | 0.014773147 | 2.385025786 |
| CCDC148-AS1 | 0.021836809 | 2.384798121 |
| RP11-609L3.2 | 0.08215688 | 2.384418389 |
| RP11-61J19.2 | 0.018382815 | 2.383602513 |
| RN7SL664P | 0.01198526 | 2.381372801 |
| HOXD13 | 0.007030254 | 0.505715281 |
| GBE1 | 0.00705012 | 1.238528082 |
| MIR548P | 0.127713774 | 2.379710488 |
| ART3 | 0.007073304 | 2.358577597 |
| PHLDA1 | 0.007096081 | 1.573175335 |
| CCDC33 | 0.007104129 | 4.30764306 |
| SLC25A24 | 0.007118569 | 1.230691135 |
| RP11-857B24.3 | 0.029488321 | 2.37660063 |
| TRAK1 | 0.007147979 | 1.399125305 |
| AC024028.1 | 0.125201466 | 2.373479708 |
| REEP4 | 0.007149339 | 0.779961453 |
| AC083899.1 | 0.042305972 | 2.37281732 |
| KRT90P | 0.09521987 | 2.372123222 |
| C11orf63 | 0.007217905 | 1.709207833 |
| RP11-117L5.4 | 0.045625102 | 2.370925718 |
| SMIM10 | 0.007219946 | 1.612633621 |
| RP11-397J20.1 | 0.122644341 | 2.369247638 |
| AC112249.2 | 0.068860968 | 2.368887747 |
| RP11-302F12.2 | 0.093935192 | 2.368483124 |
| ZNF721 | 0.007220937 | 1.221058238 |
| TNC | 0.007233052 | 2.013432245 |
| RP11-834C11.6 | 0.00095724 | 2.365607924 |
| RP11-611E13.3 | 0.056724484 | 2.363492634 |
| RP11-22A3.1 | 0.032878661 | 2.362860283 |
| DOK7 | 0.0072596 | 2.368262274 |
| CTC-431G16.2 | 0.115532746 | 2.361373268 |
| RPS16P8 | 0.090800244 | 2.361234552 |
| RP11-56A10.1 | 0.086339473 | 2.360193726 |
| AC004160.4 | 0.09085338 | 2.359536645 |
| MOB1A | 0.007274255 | 1.124811114 |
| RN7SL284P | 0.136941702 | 2.356760274 |
| AC005229.5 | 0.13731042 | 2.356274277 |
| CRYM | 0.007297943 | 4.53005713 |
| AC112220.2 | 0.075458407 | 2.354609402 |
| SNCA-AS1 | 0.015844516 | 2.354239116 |
| CH507-42P11.5 | 0.094185428 | 2.352174657 |
| UFM1 | 0.007323751 | 1.190458502 |
| ATP4B | 0.007327355 | 2.993186019 |
| TATDN2 | 0.007334115 | 1.160488498 |
| SARS2 | 0.007361111 | 0.744781372 |
| RPEP4 | 0.018989516 | 2.349220772 |
| RNU6-460P | 0.147493793 | 2.349174616 |
| C4B | 0.00739537 | 2.187198366 |
| RNA5SP174 | 0.091284437 | 2.348709292 |
| WDR93 | 0.007416779 | 2.178465155 |
| RP11-285G1.2 | 0.010990626 | 2.347224159 |
| BAG1 | 0.007446543 | 1.277868449 |
| AC008264.4 | 0.017361934 | 2.345552685 |
| RP11-268G13.1 | 0.083661348 | 2.34516063 |
| MIR3199-2 | 0.085784865 | 2.344586147 |
| ZFYVE16 | 0.007471161 | 1.155938691 |
| THOC7 | 0.007488844 | 1.179807148 |
| INTU | 0.007519228 | 1.262575452 |
| MIR196A2 | 0.056902461 | 2.340257026 |
| RP11-573N10.1 | 0.03922194 | 2.340011847 |
| MIR1268A | 0.026496463 | 2.339053898 |
| PKP3 | 0.00759715 | 0.78731085 |
| MIB2 | 0.00761146 | 0.803273451 |
| C9orf64 | 0.007620648 | 1.252867275 |
| SHANK2-AS1 | 0.05858093 | 2.334960223 |
| RP11-674E16.4 | 0.025874317 | 2.333233838 |
| RP1-196A12.1 | 0.144086135 | 2.332964992 |
| RNU6-20P | 0.122247846 | 2.332595632 |
| C22orf29 | 0.007676387 | 1.216739927 |
| CAPN13 | 0.0077384 | 2.151030301 |
| AGPAT6 | 0.007739646 | 1.155441322 |
| C5orf15 | 0.007745543 | 1.182729211 |
| ARHGAP42P2 | 0.140834076 | 2.325768883 |
| ZNF627 | 0.007782303 | 1.219955036 |
| RP11-448G4.4 | 0.100785581 | 2.324868423 |
| AC007009.2 | 0.047376352 | 2.324210093 |
| RP11-7C6.1 | 0.143348878 | 2.32016517 |
| RNU4-29P | 0.022007139 | 2.319376708 |
| ZNF512B | 0.007784504 | 0.762054687 |
| NIFKP3 | 0.01483984 | 2.318666282 |
| RP11-167N4.2 | 0.052276983 | 2.318067685 |
| RP11-95I16.2 | 0.092733123 | 2.317522843 |
| ACKR4 | 0.007802954 | 1.968212982 |
| RP11-1260E13.3 | 0.02212591 | 2.31523013 |
| CTB-35F21.4 | 0.111381954 | 2.314232936 |
| RP11-521M14.1 | 0.075008396 | 2.313187152 |
| METTL8 | 0.007830804 | 0.795061935 |
| RP5-947P14.2 | 0.136263624 | 2.313089643 |
| AC016735.2 | 0.109787248 | 2.31110547 |
| RP11-1250I15.1 | 0.031785977 | 2.31049392 |
| LINC00683 | 0.115952907 | 2.309376725 |
| RNF39 | 0.007848016 | 1.533070232 |
| TPTE2P3 | 0.007404237 | 2.304888051 |
| AL358815.1 | 0.006323478 | 2.30346169 |
| SP6 | 0.007871806 | 2.229709848 |
| TARDBPP2 | 0.068842318 | 2.30288478 |
| ANKRD20A5P | 0.019844671 | 2.302632941 |
| RP11-1127D7.1 | 0.033282455 | 2.300781585 |
| NUS1P3 | 0.095855406 | 2.300742828 |
| RP3-406A7.1 | 0.149193338 | 2.299794622 |
| U3 | 0.050048896 | 2.299775454 |
| VWA3A | 0.007918623 | 6.521690101 |
| TMCO3 | 0.007943043 | 1.297158532 |
| CH507-24F1.2 | 0.002186825 | 2.298503867 |
| RP11-65J3.3 | 0.010246174 | 2.298134934 |
| WDR86-AS1 | 0.002859695 | 2.298119794 |
| RP11-71E19.1 | 0.007463226 | 2.297008238 |
| PAUPAR | 0.029224322 | 2.29633451 |
| PROX1-AS1 | 0.103334844 | 2.295881767 |
| RP11-399H11.2 | 0.071481966 | 2.294797488 |
| AC069213.1 | 0.001260716 | 2.292848482 |
| SRRM3 | 0.007980398 | 0.603434695 |
| RP11-162I7.1 | 0.100671223 | 2.292047296 |
| PXK | 0.008016948 | 1.225764531 |
| DNAH7 | 0.008022422 | 2.465431222 |
| C1RL | 0.008089475 | 1.211599276 |
| FAM204CP | 0.0890611 | 2.289772878 |
| TCF7L2 | 0.008108166 | 1.202229077 |
| RP11-857B24.2 | 0.104337459 | 2.286912846 |
| SIRT2 | 0.008130422 | 0.77188722 |
| CDKN1C | 0.008135158 | 1.59126837 |
| CTD-2538A21.1 | 0.022119336 | 2.283775853 |
| LINC00472 | 0.002301374 | 2.283058442 |
| ZNF90P2 | 0.120618145 | 2.282681577 |
| RP5-991C6.4 | 0.032154758 | 2.28266397 |
| RP3-436N22.3 | 0.051074929 | 2.28084877 |
| RP11-35J10.4 | 0.017277158 | 2.280288969 |
| RNF4 | 0.008142501 | 1.16932427 |
| RNU6-469P | 0.038003701 | 2.278741442 |
| RP11-703H8.7 | 0.053186604 | 2.277518903 |
| RP11-25L3.3 | 0.061534028 | 2.277067964 |
| RNF14 | 0.008145841 | 1.139044189 |
| RHOT1P1 | 0.018640718 | 2.276876494 |
| RP11-744N12.3 | 0.130835996 | 2.276488797 |
| NOP56P2 | 0.069713783 | 2.276378006 |
| RP11-345K9.3 | 0.049599405 | 2.27626552 |
| SEPT4-AS1 | 0.01348305 | 2.276087695 |
| ARIH2 | 0.008184244 | 1.170992807 |
| KCTD14 | 0.008206195 | 1.701139684 |
| HMGB1P28 | 0.006133803 | 2.274425139 |
| AC012307.2 | 0.099169965 | 2.271628127 |
| CALML5 | 0.008226254 | 0.492438939 |
| ME3 | 0.008255043 | 1.507850965 |
| AC091038.1 | 0.14507295 | 2.268055115 |
| RNA5SP342 | 0.078452585 | 2.267704062 |
| RP11-350E12.4 | 0.024170201 | 2.267314341 |
| Y_RNA | 0.127823698 | 2.266868178 |
| MRPL50P2 | 0.05265267 | 2.266322779 |
| METTL15P3 | 0.118819629 | 2.26512017 |
| RNU6-826P | 0.103184929 | 2.26398249 |
| SULT1C2P1 | 0.107163093 | 2.262514208 |
| AC093928.1 | 0.093266027 | 2.260576081 |
| RP4-569M23.2 | 0.003614247 | 2.259887387 |
| RPL15P21 | 0.054167054 | 2.259488839 |
| HMGB1P7 | 0.062352706 | 2.259379162 |
| RNU5A-8P | 0.041236925 | 2.258245245 |
| PTPMT1 | 0.008267532 | 1.169346414 |
| SEL1L3 | 0.008332789 | 1.442439701 |
| AC099757.1 | 0.100928843 | 2.257651301 |
| RP11-179K3.2 | 0.123475018 | 2.256592918 |
| LDLRAD1 | 0.008348707 | 3.368240994 |
| CCDC62 | 0.00838522 | 1.489505948 |
| NME9 | 0.008386818 | 2.408317586 |
| SAR1A | 0.00841055 | 1.11385551 |
| RP11-466G12.3 | 0.012939423 | 2.251344515 |
| RP5-837I24.4 | 0.138640634 | 2.251102917 |
| GUCY2GP | 0.026059983 | 2.250902487 |
| CTD-3023L14.2 | 0.094753309 | 2.250596534 |
| TGFBR2 | 0.008430161 | 1.40913021 |
| OR7E11P | 0.074889592 | 2.249358868 |
| CTC-820M8.1 | 0.053302472 | 2.249296079 |
| SLC6A19 | 0.008449845 | 6.089984238 |
| PSMD8 | 0.008470878 | 0.798562407 |
| RP11-205A8.3 | 0.040871585 | 2.246458402 |
| RP11-410N8.1 | 0.096613993 | 2.245951264 |
| RPL5P26 | 0.028765689 | 2.245884237 |
| RP11-647F2.2 | 0.017711277 | 2.244631138 |
| RP11-284H19.1 | 0.070356321 | 2.244532448 |
| TMEM107 | 0.008482203 | 1.248904872 |
| MYPN | 0.008515025 | 4.682544022 |
| JUP | 0.008521569 | 0.752338558 |
| RP11-439C15.4 | 0.059278018 | 2.242343492 |
| AC008592.8 | 0.078106274 | 2.24166239 |
| RP11-21C17.1 | 0.130134983 | 2.241656427 |
| MIR8083 | 0.119819642 | 2.241493208 |
| CTB-85P21.1 | 0.039733167 | 2.241303471 |
| RP3-503A6.2 | 0.092539481 | 2.240427481 |
| METTL21AP1 | 0.067362346 | 2.239856488 |
| MNS1 | 0.008532963 | 1.521139553 |
| GPX2 | 0.008551634 | 0.325220647 |
| EFHB | 0.008554628 | 1.789703341 |
| IQCE | 0.008601168 | 1.342502888 |
| ASNSP6 | 0.099417959 | 2.237398249 |
| RN7SL604P | 0.085762578 | 2.236124946 |
| FAM8A6P | 0.089316075 | 2.235379115 |
| RP11-192C21.3 | 0.131013146 | 2.23483461 |
| MIR3189 | 0.025704648 | 2.234534005 |
| SNRPGP1 | 0.084453371 | 2.233794943 |
| HNRNPLL | 0.008609698 | 1.1569115 |
| GS1-124K5.7 | 0.040769027 | 2.231587193 |
| RP11-472N19.3 | 0.123222509 | 2.23134788 |
| EPM2AIP1 | 0.008645199 | 1.239009628 |
| MAGI1 | 0.008658229 | 1.330603004 |
| IP6K1 | 0.008694875 | 1.157471873 |
| RP11-556O9.3 | 0.012113223 | 2.228830271 |
| AL117209.1 | 0.066514912 | 2.228595606 |
| RP11-320G24.1 | 0.021715181 | 2.228332642 |
| RP11-103B5.4 | 0.025559103 | 2.227734122 |
| LINC00499 | 0.107035543 | 2.227503711 |
| RP11-344B5.2 | 0.003300706 | 2.227436578 |
| TGFB2-AS1 | 0.012513017 | 2.227055679 |
| AP000289.6 | 0.129081223 | 2.226884796 |
| MLK7-AS1 | 0.005540956 | 2.226069695 |
| AC034110.1 | 0.131521457 | 2.225930773 |
| SFT2D1 | 0.008695902 | 1.155570054 |
| CACNA1B | 0.008697801 | 0.314146085 |
| AC019117.1 | 0.114211897 | 2.225591657 |
| GPC4 | 0.008727313 | 3.190396808 |
| RNF7P1 | 0.034189433 | 2.22237294 |
| AC017002.4 | 0.005258302 | 2.222104444 |
| OR9N1P | 0.023857011 | 2.221857161 |
| NDUFB1P2 | 0.002119658 | 2.221494938 |
| DSC3 | 0.008741197 | 0.645777405 |
| KHDC1P1 | 0.096475964 | 2.220759332 |
| RP1-65J11.1 | 0.020704778 | 2.219505647 |
| TCL6 | 0.054209316 | 2.218876032 |
| LINC00309 | 0.060856283 | 2.217194503 |
| RP11-304L19.12 | 0.001993788 | 2.217042557 |
| CFAP74 | 0.008775613 | 4.642908507 |
| RN7SL45P | 0.024389298 | 2.214588258 |
| CSNK1E | 0.008792125 | 1.2013844 |
| RP11-713P14.1 | 0.046581695 | 2.213502343 |
| TRDJ3 | 0.076415492 | 2.213365995 |
| AC104809.3 | 0.008795766 | 5.642839451 |
| RNU6-1093P | 0.075203807 | 2.212376908 |
| EIF4EP3 | 0.066746024 | 2.211990787 |
| RP11-141M3.6 | 0.036199927 | 2.211882124 |
| CTD-2085J24.3 | 0.085124882 | 2.211525488 |
| PIK3C2A | 0.008809375 | 1.208759813 |
| RP4-710M3.2 | 0.009597075 | 2.211417651 |
| VEPH1 | 0.008873107 | 2.42423531 |
| C20orf78 | 0.129885217 | 2.211076822 |
| LRRC43 | 0.008901116 | 2.546116311 |
| SNORA51 | 0.001767677 | 2.210775049 |
| RP11-446J8.1 | 0.139034434 | 2.210231474 |
| ACSM3 | 0.008921047 | 2.007717113 |
| TTLL3 | 0.00895611 | 1.360580755 |
| ENPP3 | 0.008974456 | 6.902542749 |
| RP11-326E22.1 | 0.100113972 | 2.205486875 |
| RHOB | 0.008985254 | 1.625450807 |
| RP11-219O3.2 | 0.078759844 | 2.203105643 |
| RNU6-1065P | 0.140779242 | 2.201941788 |
| NTAN1P3 | 0.083543547 | 2.201018137 |
| RP11-611O2.2 | 0.067100903 | 2.199844539 |
| Y_RNA | 0.131365816 | 2.198532579 |
| RP1-102G20.4 | 0.039313494 | 2.198184244 |
| MIR5572 | 0.018101095 | 2.196863408 |
| C9orf92 | 0.008990785 | 2.524464402 |
| RP11-405F3.4 | 0.016446107 | 2.194019613 |
| RP1-191J18.65 | 0.043383099 | 2.193492138 |
| CP | 0.008991253 | 3.353103434 |
| HHATL-AS1 | 0.037817258 | 2.193035486 |
| AC013722.1 | 0.083630543 | 2.192731997 |
| RP11-809H16.3 | 0.019661352 | 2.191568363 |
| EVPL | 0.00900118 | 0.697145229 |
| AP000445.1 | 0.056239535 | 2.191457591 |
| RP11-77I22.4 | 0.012016289 | 2.191330818 |
| RP11-1109M24.5 | 0.145486004 | 2.189553763 |
| RP11-203H19.2 | 0.048380241 | 2.188677263 |
| PGM2L1 | 0.009023149 | 1.443229621 |
| RNU6-1160P | 0.010651294 | 2.188020119 |
| TRIM17 | 0.009030112 | 1.805594918 |
| RPL18AP14 | 0.102485785 | 2.186616585 |
| GUCA1A | 0.009087198 | 2.80619058 |
| RP11-403A3.2 | 0.039521007 | 2.185790982 |
| RNU6-194P | 0.124121398 | 2.185120467 |
| ZNF638 | 0.009090732 | 1.12162436 |
| RP11-480G7.2 | 0.015062411 | 2.180093267 |
| CR1L | 0.009093453 | 1.829722234 |
| SLFN11 | 0.009185849 | 1.437227763 |
| C4BPB | 0.009195133 | 7.441829343 |
| CADM1 | 0.009209411 | 2.276889357 |
| C12orf50 | 0.009210241 | 1.735295164 |
| RP13-494C23.1 | 0.049350035 | 2.177907532 |
| DHRS7B | 0.009226181 | 1.198078865 |
| RP11-466F5.3 | 0.005637318 | 2.177364043 |
| RP11-156L14.1 | 0.005124298 | 2.176543012 |
| KLHL8 | 0.009236384 | 1.30699765 |
| CTC-490E21.13 | 0.096333319 | 2.17299284 |
| RP11-528A4.2 | 0.022875627 | 2.172701043 |
| RPL39P25 | 0.063402627 | 2.172022609 |
| RP11-1105G2.4 | 0.000614506 | 2.17170135 |
| AL138706.1 | 0.080080963 | 2.171140429 |
| RP11-14C10.4 | 0.117290862 | 2.17091733 |
| CTD-2363C16.1 | 0.112110048 | 2.170077886 |
| RP3-340N1.2 | 0.092674081 | 2.16854534 |
| TTC25 | 0.009236825 | 2.536125965 |
| GS1-345D13.1 | 0.093830798 | 2.167710427 |
| CTB-54I1.1 | 0.046133464 | 2.165753096 |
| RP11-1080G15.2 | 0.010844153 | 2.165560339 |
| CHRNB1 | 0.009243576 | 1.330393762 |
| SELK | 0.009299788 | 1.167488524 |
| ZNF880 | 0.009327206 | 1.606341899 |
| LINC01124 | 0.02536676 | 2.163762972 |
| AC079781.7 | 0.034218696 | 2.162652843 |
| Metazoa_SRP | 0.119566883 | 2.160463897 |
| RP11-1084J3.3 | 0.128714337 | 2.160370181 |
| USP43 | 0.009349164 | 1.302534003 |
| RP11-525G12.1 | 0.052092019 | 2.158674449 |
| CNOT7P2 | 0.135823852 | 2.158511632 |
| GS1-519E5.1 | 0.116286323 | 2.156620986 |
| RP2 | 0.00935603 | 1.238487563 |
| OR2T32P | 0.083792081 | 2.153704894 |
| NFE2 | 0.009360395 | 2.479941031 |
| GS1-122H1.1 | 0.118697404 | 2.152590881 |
| AC009502.1 | 0.0323396 | 2.152122167 |
| EPPIN-WFDC6 | 0.009395391 | 3.279184326 |
| LMO7DN-IT1 | 0.039847256 | 2.151024121 |
| AP001625.4 | 0.050949441 | 2.150193426 |
| HMGB3P31 | 0.120832822 | 2.150113583 |
| RP11-65B23.3 | 0.035331313 | 2.149381364 |
| RP11-470B22.1 | 0.041323739 | 2.148096889 |
| RP11-370B11.3 | 0.040144 | 2.147203209 |
| CTD-2196E14.8 | 0.065473494 | 2.146983129 |
| LINC00589 | 0.001320754 | 2.146674066 |
| EPOR | 0.009457269 | 1.698250348 |
| RP11-998D10.4 | 0.129192505 | 2.145423936 |
| FAM189A2 | 0.009469624 | 1.885951592 |
| RSL24D1P3 | 0.093520132 | 2.145264017 |
| ATF1 | 0.009485397 | 1.137394972 |
| CDS1 | 0.009498979 | 1.210733183 |
| MIR196A1 | 0.00107674 | 2.142802338 |
| MARK3P1 | 0.122063252 | 2.142037316 |
| ENKUR | 0.009499836 | 2.799931298 |
| ARMC3 | 0.009500276 | 3.497552567 |
| ARL6IP1P3 | 0.019359982 | 2.140559654 |
| SUCLG2 | 0.009508174 | 1.241917862 |
| AL133244.2 | 0.065638397 | 2.139983707 |
| PRKRIRP8 | 0.068607587 | 2.139627438 |
| TRAJ28 | 0.101619619 | 2.138048398 |
| RN7SL344P | 0.102447612 | 2.137451778 |
| RNA5SP399 | 0.116100524 | 2.136139889 |
| SPATA17 | 0.009527872 | 2.067635526 |
| AC007163.3 | 0.041312752 | 2.134619319 |
| TCL1B | 0.009533483 | 5.697858885 |
| DNAJA1P2 | 0.134931707 | 2.132504544 |
| CYP21A1P | 0.051286595 | 2.132426166 |
| TAPT1 | 0.009557632 | 1.17727518 |
| FAM122B | 0.009558359 | 1.23062619 |
| LINC01226 | 0.054278914 | 2.128974604 |
| RP11-93G5.1 | 0.000643519 | 2.128623723 |
| CTB-22K14.1 | 0.086751771 | 2.127068553 |
| RP11-677M24.1 | 0.013988617 | 2.127014321 |
| RP11-355F22.1 | 0.143135245 | 2.126862703 |
| RP11-167P22.3 | 0.128033065 | 2.126341883 |
| AC011816.1 | 0.004754054 | 2.125744342 |
| RBM6 | 0.009568527 | 1.213397909 |
| FBXO7 | 0.009577072 | 1.110861973 |
| RAB33B | 0.009585156 | 1.159128471 |
| FLJ40194 | 0.039326715 | 2.124781278 |
| ZBTB7B | 0.009622463 | 0.787449206 |
| OXNAD1 | 0.009639192 | 1.187533449 |
| TRDJ2 | 0.094279894 | 2.120527246 |
| ETV2 | 0.009649002 | 0.664630897 |
| RPL37P1 | 0.007943146 | 2.117475261 |
| SLC16A2 | 0.009672307 | 1.783169708 |
| AC226118.1 | 0.040275044 | 2.116150775 |
| RP11-578F21.6 | 0.06894447 | 2.115310807 |
| RNU4-48P | 0.042921841 | 2.115099569 |
| RCN1P1 | 0.142993688 | 2.114936265 |
| RP11-514F3.5 | 0.013320569 | 2.11408772 |
| DISC1 | 0.009704803 | 1.466351017 |
| RAB23 | 0.009729187 | 1.29759099 |
| PDE10A | 0.009775843 | 2.125353088 |
| MIR582 | 0.10597571 | 2.112334278 |
| HMGB1P41 | 0.003067673 | 2.110178794 |
| RP11-312A15.3 | 0.13447016 | 2.10829822 |
| AP000998.2 | 0.104673935 | 2.108157903 |
| CMB9-22P13.2 | 0.048099456 | 2.108137961 |
| RP11-44K6.3 | 0.020729519 | 2.107445936 |
| RP11-512N21.3 | 0.066385921 | 2.106578818 |
| RP11-35J10.6 | 0.109422793 | 2.106137178 |
| RP11-61J19.3 | 0.022166181 | 2.105341146 |
| CTD-2113L7.1 | 0.109326216 | 2.104100923 |
| RP11-669C19.1 | 0.022683479 | 2.10378909 |
| Y_RNA | 0.010799024 | 2.103444962 |
| RP11-451O13.1 | 0.063233688 | 2.103353697 |
| AC000367.1 | 0.044971846 | 2.101556795 |
| Y_RNA | 0.128106213 | 2.101011453 |
| PCGF5 | 0.009781612 | 1.19279065 |
| AC068137.5 | 0.102877973 | 2.100578137 |
| ABCC6P1 | 0.071322403 | 2.100232872 |
| PPP1R26 | 0.009810877 | 1.246121315 |
| COL18A1-AS1 | 0.020409032 | 2.100022404 |
| RP11-466L17.1 | 0.037647782 | 2.099572086 |
| RP11-845M18.7 | 0.002677082 | 2.099357456 |
| RP11-4C20.3 | 0.01343994 | 2.09862952 |
| CTD-3105H18.10 | 0.120078045 | 2.098447964 |
| ADCY6 | 0.009822878 | 1.36411162 |
| BMPR1APS2 | 0.004301198 | 2.097184717 |
| MRPS18CP6 | 0.008876962 | 2.097052822 |
| CTD-2314G24.2 | 0.018073206 | 2.096658498 |
| ANO10 | 0.009825848 | 1.214148066 |
| METTL14 | 0.009833143 | 1.132370141 |
| AC016582.2 | 0.133217628 | 2.093380661 |
| SMPDL3B | 0.009839081 | 1.421062181 |
| CCDC108 | 0.009855365 | 3.226974602 |
| FNDC1-IT1 | 0.029008232 | 2.092647027 |
| MIR5195 | 0.049477751 | 2.091859741 |
| RP11-813N20.3 | 0.133748825 | 2.091725101 |
| MIR1-1HG | 0.047585808 | 2.091477405 |
| HPDL | 0.009856736 | 0.670627363 |
| RP11-399N22.3 | 0.060848823 | 2.091034761 |
| RP5-1065P14.2 | 0.025967278 | 2.090048534 |
| G3BP2 | 0.00986729 | 1.17468753 |
| AC011239.2 | 0.072652073 | 2.089566213 |
| RP11-248G5.9 | 0.077275379 | 2.089198923 |
| U82695.9 | 0.091063164 | 2.088960254 |
| RNU6-851P | 0.034897258 | 2.088538264 |
| AC091849.1 | 0.08341495 | 2.087653674 |
| B3GALTL | 0.009910429 | 1.199265198 |
| RP11-48F14.1 | 0.094324002 | 2.086939821 |
| AL157902.3 | 0.042066303 | 2.086838046 |
| RP11-520A21.1 | 0.100090037 | 2.086022541 |
| EFNA3 | 0.009924905 | 0.571276971 |
| TPM1 | 0.009928392 | 1.476708505 |
| RP11-274J7.3 | 0.007267256 | 2.085419682 |
| KIAA1191 | 0.009928462 | 1.141386749 |
| RNU6-944P | 0.096596822 | 2.084942346 |
| RP11-111H3.3 | 0.04341871 | 2.084830135 |
| FNDC3A | 0.009955822 | 1.338244293 |
| FOXE3 | 0.010040393 | 0.49146287 |
| MIR6810 | 0.14170319 | 2.083410351 |
| RN7SL525P | 0.010984678 | 2.083301367 |
| RN7SL513P | 0.054288434 | 2.083060292 |
| ACTBP8 | 0.079151576 | 2.082890776 |
| RP11-374M1.5 | 0.004301383 | 2.082427618 |
| AC073842.19 | 0.009536906 | 2.082181755 |
| ITPR1-AS1 | 0.000316501 | 2.081777315 |
| TRGVB | 0.031284983 | 2.081573135 |
| MIR576 | 0.103016849 | 2.081302509 |
| NSUN7 | 0.010051572 | 1.515527356 |
| KBTBD11-OT1 | 0.055330122 | 2.079632191 |
| IL6 | 0.010123995 | 2.378602195 |
| AC016735.1 | 0.133817686 | 2.078667626 |
| RP13-210D15.4 | 0.023201212 | 2.078400296 |
| DNM1P38 | 0.145144646 | 2.078146767 |
| LINC01460 | 0.002685065 | 2.077758335 |
| AC139099.4 | 0.029716035 | 2.077671405 |
| RP11-290H9.4 | 0.010239391 | 2.076671459 |
| RN7SKP25 | 0.14764218 | 2.076373803 |
| AC090420.1 | 0.023683375 | 2.075507988 |
| RNU6-354P | 0.110466996 | 2.074762343 |
| AC097468.4 | 0.107179436 | 2.073184763 |
| OTUD1 | 0.010173367 | 1.346476011 |
| ZSCAN9 | 0.010184432 | 1.230226412 |
| RP11-253I19.4 | 0.03681136 | 2.072283028 |
| RP11-218M11.7 | 0.077018791 | 2.071985433 |
| HSPD1P11 | 0.015388786 | 2.071662952 |
| RPL36AP33 | 0.040703803 | 2.071406617 |
| ZNF443 | 0.010185563 | 1.321772317 |
| RP11-661C8.2 | 0.049912016 | 2.070785476 |
| TRBV6-7 | 0.11538258 | 2.07005735 |
| RNU6-136P | 0.091513996 | 2.069899838 |
| SPATA13 | 0.041716487 | 2.069562379 |
| ACBD7 | 0.01018635 | 1.829019819 |
| PPM1B | 0.010223115 | 1.161664996 |
| ACTL6B | 0.010238963 | 0.453037722 |
| RPL21P107 | 0.044937434 | 2.067285106 |
| AL033378.1 | 0.039327219 | 2.066395304 |
| RP11-443B7.2 | 0.004458629 | 2.066294707 |
| GPALPP1 | 0.010239009 | 1.165956431 |
| HSBP1P2 | 0.084017194 | 2.063627798 |
| RP11-944C7.1 | 0.126184805 | 2.063608425 |
| MTND4P9 | 0.001279593 | 2.060992212 |
| RP11-384K6.4 | 0.010305029 | 2.059961123 |
| TMEM110 | 0.01027032 | 1.183219673 |
| RP11-686D22.2 | 0.146547703 | 2.059384446 |
| HOTAIR_5 | 0.047913582 | 2.059014894 |
| PLA2G2E | 0.010295751 | 3.586911878 |
| RP1-124C6.1 | 0.089920341 | 2.057341563 |
| FAM20C | 0.010334176 | 1.360797327 |
| LRRC19 | 0.010345288 | 3.361415611 |
| RP11-136C24.1 | 0.093865878 | 2.056880727 |
| RN7SL510P | 0.002064398 | 2.055490563 |
| CCAR1 | 0.010381148 | 1.136321574 |
| RP11-12A2.3 | 0.112599459 | 2.053416942 |
| RP11-609L23.2 | 0.134887951 | 2.052263242 |
| C2orf73 | 0.010388006 | 3.352469442 |
| C7orf57 | 0.010391264 | 3.178041814 |
| RP11-240G22.4 | 0.030002517 | 2.050185646 |
| CTC-311G1.1 | 0.112648732 | 2.049097843 |
| AL359314.1 | 0.020876438 | 2.04819878 |
| CTD-3105H18.9 | 0.016476671 | 2.047342822 |
| RP11-157L3.6 | 0.020653103 | 2.04728006 |
| KIF27 | 0.010407692 | 1.322688585 |
| RNU6-188P | 0.099987095 | 2.046638641 |
| SPATA24 | 0.010413201 | 1.255736501 |
| EFCAB10 | 0.01041722 | 1.688002749 |
| RP11-157L3.9 | 0.004036546 | 2.043614556 |
| PBLD | 0.010462874 | 1.742647768 |
| RPL21P54 | 0.006892235 | 2.042479144 |
| RCHY1 | 0.010463263 | 1.172821295 |
| AC024569.1 | 0.090532908 | 2.042256943 |
| ARHGEF38 | 0.010464494 | 2.319317083 |
| RP13-631K18.3 | 0.018566568 | 2.042138486 |
| MIR150 | 0.123821475 | 2.041871276 |
| RP11-445K13.2 | 0.102395366 | 2.041373846 |
| LINC00477 | 0.120909221 | 2.041328252 |
| RP4-673D20.4 | 0.100191447 | 2.041231957 |
| AC008268.2 | 0.141511344 | 2.040965811 |
| CTA-363E6.6 | 0.028371456 | 2.040821199 |
| BMPR1A | 0.01047682 | 1.188198222 |
| RP11-259O18.4 | 0.144064381 | 2.038783919 |
| PPIAP24 | 0.05216798 | 2.038309193 |
| IL32 | 0.010495003 | 1.492012842 |
| RP11-244F12.3 | 0.017697504 | 2.035950059 |
| AC245407.1 | 0.009095448 | 2.035142422 |
| NKPD1 | 0.010516083 | 0.499156552 |
| PDCD6IP | 0.01052443 | 1.152371049 |
| U6 | 0.101677518 | 2.03475189 |
| RP5-935K16.2 | 0.000329196 | 2.034450291 |
| GGT3P | 0.111259787 | 2.033951588 |
| GOLGA2P4 | 0.075636348 | 2.033137242 |
| TCF12 | 0.010530946 | 1.234790705 |
| AC137590.1 | 0.030616257 | 2.031175046 |
| RN7SL262P | 0.001995012 | 2.030439432 |
| PAPPA | 0.010550735 | 3.039270167 |
| SEMA4A | 0.010560426 | 0.7901443 |
| POLR3GP1 | 0.068698717 | 2.029591031 |
| RP1-38C16.2 | 0.070607766 | 2.029051812 |
| LINC00939 | 0.038191146 | 2.028952896 |
| RP11-218I21.2 | 0.076596231 | 2.028325339 |
| PGAM1P5 | 0.002944388 | 2.028184214 |
| RP11-820K3.4 | 0.104271308 | 2.027253899 |
| AC017076.1 | 0.104109862 | 2.025905139 |
| AC005592.1 | 0.078848999 | 2.024764676 |
| RDH8 | 0.010620435 | 2.317443834 |
| CTD-2199O4.4 | 0.12501127 | 2.023728661 |
| RP11-141O11.1 | 0.077245515 | 2.023660265 |
| RP11-23E19.2 | 0.139143715 | 2.023289722 |
| KRT18P2 | 0.037139183 | 2.022708249 |
| RP11-193M21.1 | 0.129630825 | 2.0221174 |
| GS1-594A7.3 | 0.01396082 | 2.021962366 |
| BVES-AS1 | 0.024596903 | 2.021866044 |
| AC004924.1 | 0.062792456 | 2.021582102 |
| ALPK3 | 0.010676947 | 1.919260411 |
| RP11-706D8.3 | 0.024235405 | 2.019727716 |
| MIR30C2 | 0.035148486 | 2.01956397 |
| RP11-22A3.2 | 0.007207695 | 2.018615078 |
| ALOX12P1 | 0.033879602 | 2.018538986 |
| ARMC2-AS1 | 0.06492405 | 2.018171339 |
| MED4 | 0.010686483 | 1.144051895 |
| CTD-2234B20.1 | 0.14934443 | 2.016435808 |
| RP11-38O14.5 | 0.06184862 | 2.015775471 |
| AC195454.1 | 0.011944339 | 2.015443723 |
| RP11-322E11.2 | 0.023269165 | 2.014936397 |
| RP11-221J22.1 | 0.113072821 | 2.014783449 |
| RP11-73E6.2 | 0.033948221 | 2.014584087 |
| RAD17P2 | 0.021755234 | 2.014269471 |
| DIXDC1 | 0.010693609 | 1.323136754 |
| BMP7 | 0.010720711 | 0.605829012 |
| RN7SL375P | 0.121904535 | 2.012227527 |
| C17orf47 | 0.010760511 | 1.841282626 |
| TSPAN6 | 0.010778158 | 1.270710386 |
| Y_RNA | 0.119142587 | 2.011568531 |
| RP11-7I15.4 | 0.019574098 | 2.011415591 |
| CTA-363E6.1 | 0.146012609 | 2.011088694 |
| RP11-104J23.1 | 0.084675233 | 2.010112329 |
| RP11-315O6.1 | 0.07721332 | 2.008548343 |
| SNORA24 | 0.057336198 | 2.008221663 |
| LINC01342 | 0.103725047 | 2.007733247 |
| LINGO3 | 0.010784103 | 0.629438209 |
| RNU6-553P | 0.004774787 | 2.006797384 |
| KHK | 0.010814998 | 0.750287744 |
| RP1-193H18.2 | 0.009768273 | 2.004911076 |
| RYBP | 0.010852778 | 1.174425886 |
| RP11-386M24.6 | 0.074807686 | 2.004162282 |
| RP11-768F21.1 | 0.040500628 | 2.002699767 |
| RP11-263C24.1 | 0.038605613 | 2.002485249 |
| RNA5SP187 | 0.007103615 | 2.002393883 |
| CH507-145C22.1 | 0.032550838 | 2.000356713 |
| RP11-106E7.1 | 0.144800805 | 1.999953994 |
| LINC01529 | 0.124575697 | 1.999585025 |
| RP11-632L2.2 | 0.051121628 | 1.999519129 |
| GLRXP3 | 0.013032427 | 1.998771294 |
| ZNF281 | 0.010885607 | 1.23844584 |
| DBI | 0.010908067 | 0.779677729 |
| CTC-436K13.1 | 0.111038105 | 1.998047726 |
| MRPL38 | 0.010916821 | 0.814301672 |
| CACNA1C-IT2 | 0.087259174 | 1.995858041 |
| RNU2-70P | 0.08909408 | 1.995845448 |
| RP11-115D19.3 | 0.044128275 | 1.994432177 |
| RP5-875H18.9 | 0.027528414 | 1.994106315 |
| RP11-125O18.1 | 0.030466388 | 1.993241192 |
| NPM1P49 | 0.072393363 | 1.992451863 |
| MIR4497 | 0.077358289 | 1.992292201 |
| PHAX | 0.010958433 | 1.122083648 |
| RP11-834C11.7 | 0.000168608 | 1.99117773 |
| RP11-141M1.3 | 0.001185466 | 1.990710618 |
| RN7SL671P | 0.004926604 | 1.990525856 |
| RP11-1069G10.2 | 0.019594023 | 1.990238231 |
| RP11-266A24.1 | 0.010866525 | 1.990155173 |
| RNU6-29P | 0.053271882 | 1.990033153 |
| AC007405.8 | 0.063388669 | 1.988333625 |
| MIR4525 | 0.1203599 | 1.988224496 |
| RP11-53M11.3 | 0.018294752 | 1.988191262 |
| MIR135A1 | 0.008347495 | 1.987676925 |
| COMMD10 | 0.010988325 | 1.182781643 |
| VWA5A | 0.010990006 | 1.447683209 |
| LRRC2-AS1 | 0.026645036 | 1.985101967 |
| RN7SL455P | 0.057502422 | 1.984867621 |
| LINC00908 | 0.024501866 | 1.984442465 |
| RP11-46C24.3 | 0.113299517 | 1.984022586 |
| AC079354.3 | 0.024196425 | 1.98258537 |
| IL17RB | 0.011057097 | 1.464322947 |
| AC016737.1 | 0.149924939 | 1.981787387 |
| CLDN15 | 0.011058173 | 0.807099 |
| RP11-21J18.1 | 0.035914067 | 1.98105183 |
| RPL29P11 | 0.062617975 | 1.98091775 |
| SLC9A3P3 | 0.052337741 | 1.980891017 |
| FOLH1 | 0.011058462 | 1.908043761 |
| SLC9A6 | 0.011081453 | 1.187115544 |
| ROPN1L | 0.011092621 | 3.256171255 |
| SNRPCP9 | 0.050879896 | 1.979558447 |
| PGAM4P1 | 0.087494462 | 1.979107539 |
| CFAP43 | 0.011098055 | 2.824744406 |
| SNORA40 | 0.081259108 | 1.978276983 |
| RP11-449J21.5 | 0.002820976 | 1.978232396 |
| RP3-525N10.2 | 0.140064765 | 1.977798754 |
| RP11-546B8.1 | 0.018128331 | 1.977670805 |
| SLMAP | 0.011134411 | 1.192904053 |
| RP11-359E8.5 | 0.101224722 | 1.977345796 |
| RP5-1065J22.4 | 0.001137727 | 1.975796343 |
| EPCAM | 0.011161126 | 1.578579789 |
| WHSC1 | 0.011168414 | 1.19304034 |
| MIR7974 | 0.083028698 | 1.974769086 |
| RP11-182N22.7 | 0.055632063 | 1.974719234 |
| F8A1 | 0.011188192 | 1.296433234 |
| VN1R85P | 0.089775575 | 1.972867047 |
| RNA5SP297 | 0.074251118 | 1.971910177 |
| C1GALT1C1 | 0.011192009 | 1.183044169 |
| RND1 | 0.011204212 | 1.58528692 |
| RP4-533D7.3 | 0.047759474 | 1.971460435 |
| RNU6-1019P | 0.050292406 | 1.9714352 |
| TRPM5 | 0.011249141 | 3.988816778 |
| ZBTB8A | 0.011249414 | 1.166000895 |
| RP3-331H24.6 | 0.009655216 | 1.970620245 |
| MSX1 | 0.011275635 | 3.923021341 |
| WBP1LP1 | 0.129677409 | 1.968956252 |
| FOXQ1 | 0.011288875 | 1.433868186 |
| ZSWIM6 | 0.011327495 | 1.200036673 |
| OR7E12P | 0.110252905 | 1.967632033 |
| RP11-115D19.1 | 0.081417263 | 1.967071345 |
| PARD3B | 0.011345428 | 1.506530919 |
| RP11-616K22.2 | 0.027681685 | 1.966125318 |
| RP11-165M1.3 | 0.055643915 | 1.965997848 |
| RN7SKP69 | 0.098899257 | 1.96598562 |
| AL138721.2 | 0.078349739 | 1.965926129 |
| PGAM1P4 | 0.132541401 | 1.965701078 |
| RP11-759A24.3 | 0.076309885 | 1.965428411 |
| TTC21A | 0.011361973 | 1.51553117 |
| SNORA51 | 0.034207176 | 1.964252061 |
| ACTR5 | 0.01137723 | 0.818930672 |
| RP11-87G24.2 | 0.133050086 | 1.963518451 |
| RNU6-644P | 0.087353778 | 1.963346068 |
| NHLRC1 | 0.011379517 | 0.720554732 |
| SMS | 0.011392617 | 1.281599824 |
| LINC01501 | 0.055213632 | 1.962357237 |
| REEP5 | 0.01139825 | 1.142228782 |
| CCT8L1P | 0.031544401 | 1.960806811 |
| RERG-IT1 | 0.013249914 | 1.960463374 |
| MIR548I1 | 0.129659763 | 1.959717172 |
| RP13-726E6.2 | 0.020804902 | 1.959119815 |
| RP11-110G21.2 | 0.01617806 | 1.958707133 |
| RP1-140K8.5 | 0.107704578 | 1.957868637 |
| KB-1254G8.1 | 0.021982246 | 1.956644332 |
| TM9SF3 | 0.011451176 | 1.186915775 |
| Y_RNA | 0.068770412 | 1.95616979 |
| BX842568.4 | 0.118037655 | 1.956069591 |
| RP11-46A10.2 | 0.026775568 | 1.956043002 |
| SLC26A6 | 0.011503273 | 1.316706381 |
| FTLP12 | 0.038661439 | 1.955669133 |
| RNU1-11P | 0.149324524 | 1.955530151 |
| AL353805.1 | 0.017188752 | 1.955344966 |
| AC016831.7 | 0.035938627 | 1.953884684 |
| UBBP3 | 0.002198532 | 1.953630072 |
| RNF182 | 0.011507722 | 13.08447624 |
| HNRNPA1P22 | 0.011801931 | 1.953090679 |
| RP11-324I22.2 | 0.071658983 | 1.952581168 |
| HMGXB3 | 0.011510041 | 1.125633289 |
| RP11-834C11.4 | 0.058461392 | 1.95063245 |
| RP11-349H17.2 | 0.027047132 | 1.950591549 |
| AC008277.1 | 0.000416565 | 1.949541153 |
| ZNF738 | 0.011517222 | 1.591350346 |
| RPL7P60 | 0.031230836 | 1.947412848 |
| LINC00846 | 0.003046846 | 1.945880097 |
| RP11-154D3.1 | 0.051757215 | 1.944320952 |
| RP13-228J13.10 | 0.117760477 | 1.943619693 |
| RP11-543N12.1 | 0.128440519 | 1.943311238 |
| AC053503.11 | 0.073147329 | 1.942806115 |
| NAV2-AS2 | 0.113240259 | 1.941111781 |
| SETD2 | 0.01154971 | 1.182837946 |
| RP11-2I17.4 | 0.144618621 | 1.940869937 |
| RP11-550I24.3 | 0.0660803 | 1.940859688 |
| RP11-157L3.4 | 0.053444055 | 1.940423328 |
| L29074.3 | 0.02661978 | 1.940179375 |
| LIN7B | 0.011549809 | 0.779555188 |
| PPP1R3B | 0.011563723 | 1.401779134 |
| Y_RNA | 0.079611039 | 1.937960816 |
| ACTBP1 | 0.148653362 | 1.936863977 |
| RP11-619J20.1 | 0.01132063 | 1.936114671 |
| RP11-503N18.1 | 0.067829071 | 1.93594797 |
| TIAM1 | 0.011584626 | 0.765374583 |
| MIR4783 | 0.123123418 | 1.935438178 |
| AC024560.2 | 0.077132661 | 1.935089465 |
| IGHVII-78-1 | 0.061734536 | 1.932832187 |
| MINK1 | 0.011592289 | 0.814484894 |
| RP3-340B19.3 | 0.085866405 | 1.93237739 |
| RP11-172C16.4 | 0.032878289 | 1.932061069 |
| CNOT8 | 0.01166866 | 1.135704441 |
| NSFL1C | 0.011672629 | 0.844406127 |
| SDC1 | 0.01168401 | 0.739743932 |
| MIR4635 | 0.012507538 | 1.929386612 |
| RP11-141M1.4 | 0.011528885 | 1.928148978 |
| RNU6-57P | 0.079814456 | 1.928072916 |
| RP1-90K10.4 | 0.112660485 | 1.928015619 |
| AC093063.2 | 0.012574585 | 1.927740233 |
| CCDC50 | 0.011695297 | 1.198312567 |
| RP11-236L14.1 | 0.124642038 | 1.92753773 |
| AC109335.1 | 0.09333997 | 1.926998232 |
| RP11-298D21.1 | 0.079855017 | 1.924903515 |
| ESR1 | 0.011713607 | 1.870246357 |
| STARD13-IT1 | 0.076957129 | 1.924209693 |
| FHL2 | 0.011747045 | 1.349283922 |
| RP11-708L7.7 | 0.075834524 | 1.924131979 |
| CTC-503J8.4 | 0.049141511 | 1.923750041 |
| HIST3H2BA | 0.148693317 | 1.923719193 |
| ZNF75D | 0.011875744 | 1.259820478 |
| RP11-1174L13.2 | 0.09814436 | 1.923211709 |
| RP1-8B1.4 | 0.102107373 | 1.923197021 |
| RP5-998N21.10 | 0.149963331 | 1.923035039 |
| NAALADL2-AS3 | 0.126864798 | 1.923008169 |
| SMIM2-IT1 | 0.121087445 | 1.922946435 |
| RP11-205M3.3 | 0.076715588 | 1.921505307 |
| RP11-439K3.3 | 0.013237856 | 1.919761978 |
| SEC63 | 0.011897077 | 1.134846358 |
| GPRC5C | 0.011915414 | 1.47248022 |
| RP13-977J11.3 | 0.048223596 | 1.919258823 |
| FAM198A | 0.011918527 | 1.721939568 |
| TBCK | 0.011923044 | 1.202464348 |
| MGME1 | 0.011931484 | 0.829563295 |
| SPAG16 | 0.011942535 | 1.348863229 |
| RP11-31H5.2 | 0.037941375 | 1.915794066 |
| TEKT1 | 0.011953852 | 5.889138962 |
| DIRAS3 | 0.012009849 | 1.794414234 |
| PHBP13 | 0.043007475 | 1.915170144 |
| RP11-540B6.3 | 0.099478585 | 1.915105787 |
| CTA-363E6.5 | 0.056781855 | 1.91447272 |
| RP11-438L7.1 | 0.111878984 | 1.914445954 |
| RP11-399D15.1 | 0.074212102 | 1.913133879 |
| PTPLA | 0.012010515 | 1.881758908 |
| IGLV2-33 | 0.06547831 | 1.911444456 |
| AC012363.13 | 0.101638278 | 1.911260075 |
| HOTAIR | 0.056354101 | 1.911220603 |
| PKHD1L1 | 0.012035222 | 12.4357411 |
| RP1-17K7.2 | 0.072458385 | 1.909806002 |
| Y_RNA | 0.056747565 | 1.909528336 |
| CTC-498J12.1 | 0.06075934 | 1.908160179 |
| RP11-302F12.10 | 0.062120044 | 1.908090658 |
| ZNF433 | 0.012036581 | 1.494959477 |
| RP11-56M3.1 | 0.000260721 | 1.908039273 |
| OVCH2 | 0.012038402 | 2.746524005 |
| EXOSC7 | 0.012061404 | 1.173419318 |
| SETD8 | 0.012064513 | 0.841909996 |
| TEKT5 | 0.012065036 | 1.521060375 |
| CSAG3 | 0.146092517 | 1.906155694 |
| RP11-46D1.2 | 0.06640235 | 1.905079715 |
| RP11-736N17.8 | 0.01631545 | 1.904278932 |
| CCDC71 | 0.012075453 | 1.171062565 |
| KB-1471A8.1 | 0.016481234 | 1.902694904 |
| LINC01032 | 0.089692117 | 1.901640798 |
| SH3BGRL2 | 0.01211156 | 1.606574563 |
| LINC00837 | 0.145063564 | 1.901378277 |
| ALMS1 | 0.012148729 | 1.191430627 |
| FOXRED2 | 0.012156049 | 0.769986851 |
| AC004066.3 | 0.062984459 | 1.900487858 |
| GNPDA2 | 0.0121615 | 1.18255131 |
| AP001187.9 | 0.140282955 | 1.899123237 |
| ATP11A-AS1 | 0.010486015 | 1.898578882 |
| TTLL12 | 0.012191454 | 0.777664588 |
| RP3-467K16.2 | 0.026197415 | 1.897284502 |
| Y_RNA | 0.05695225 | 1.896707234 |
| KRT18P58 | 0.024823907 | 1.896627138 |
| AF131215.1 | 0.042974184 | 1.896540003 |
| AC007405.4 | 0.005295776 | 1.896193516 |
| AC104634.2 | 0.094246165 | 1.895595773 |
| TRMT11 | 0.012198322 | 1.191996037 |
| LINC00571 | 7.61E-06 | 1.894756838 |
| MIR3197 | 0.123469166 | 1.894669863 |
| AC084859.1 | 0.034206558 | 1.894386724 |
| RP11-274M17.1 | 0.09635917 | 1.893831684 |
| RP1-67A8.3 | 0.041233768 | 1.893048188 |
| CTD-2028E8.2 | 0.135292568 | 1.891924542 |
| SPAG8 | 0.012207146 | 2.113998139 |
| AC007391.2 | 0.016273237 | 1.891187258 |
| RNU1-61P | 0.064703539 | 1.890319253 |
| AC105053.3 | 0.05901964 | 1.888437942 |
| MPL | 0.012213792 | 1.336997072 |
| RP11-720N19.2 | 0.143090031 | 1.887345932 |
| LRRC2 | 0.012216056 | 2.643944699 |
| CNOT10 | 0.012234065 | 1.146490294 |
| TTC17 | 0.012256322 | 1.140363233 |
| GFM2 | 0.012275616 | 1.15016842 |
| RP11-325E5.4 | 0.013517711 | 1.885510029 |
| CTA-797E19.2 | 0.04905969 | 1.885381811 |
| ISOC1 | 0.012297769 | 1.208764431 |
| RP11-331F9.3 | 0.005266522 | 1.884380859 |
| ANAPC10P1 | 0.025593646 | 1.884051884 |
| VN1R12P | 0.090971849 | 1.884006661 |
| LINC00839 | 0.143885863 | 1.883812543 |
| MTND1P10 | 0.081306369 | 1.882987111 |
| AC079753.4 | 0.006342473 | 1.882209979 |
| RP5-1121E10.2 | 0.041840115 | 1.882044648 |
| TCTN3 | 0.012302112 | 1.202669524 |
| CTD-2600O9.1 | 0.026744104 | 1.879618785 |
| AC005009.1 | 0.131991422 | 1.878403467 |
| AC024592.9 | 0.110084631 | 1.878292406 |
| GRSF1 | 0.012303068 | 1.144667529 |
| RP11-359D14.2 | 0.000600817 | 1.877821587 |
| RP11-145A3.4 | 0.025692113 | 1.877343697 |
| SNORA48 | 0.103117638 | 1.877285373 |
| ZNF847P | 0.064255885 | 1.877152562 |
| RP11-167N4.4 | 0.009356906 | 1.876145121 |
| IMMP1LP2 | 0.056027026 | 1.876047556 |
| PHF10 | 0.012346133 | 1.191619341 |
| RP11-219A15.4 | 0.128180812 | 1.873759796 |
| CTD-2545G14.4 | 0.011942727 | 1.873364326 |
| GAS1 | 0.012366703 | 1.965174128 |
| SNX6P1 | 0.060361842 | 1.87262671 |
| AC073316.2 | 0.034532151 | 1.871884089 |
| Metazoa_SRP | 0.021583816 | 1.87154427 |
| RP11-490G8.1 | 0.070084997 | 1.870378839 |
| RP11-696F12.1 | 0.059913098 | 1.870321369 |
| HIGD1A | 0.012409147 | 1.177372512 |
| CTD-3001H11.1 | 0.0591337 | 1.870037048 |
| RP11-240L7.4 | 0.067769924 | 1.869712414 |
| AP000797.1 | 0.017552287 | 1.869097342 |
| PHBP10 | 0.055917901 | 1.868273845 |
| TRIM31-AS1 | 0.017546116 | 1.867547036 |
| ARHGAP26-AS1 | 0.147115064 | 1.866618583 |
| RP11-64C12.1 | 0.033549956 | 1.866512212 |
| C10orf32 | 0.012435741 | 1.165223664 |
| HFM1 | 0.012530334 | 1.726892267 |
| CTD-2525P14.5 | 0.034366842 | 1.865994742 |
| CTB-134H23.3 | 0.057251939 | 1.865137671 |
| LINC01267 | 0.020181132 | 1.865112034 |
| RP11-158G18.1 | 0.150961422 | 1.864116565 |
| CDKL4 | 0.012532905 | 2.079852867 |
| KNCN | 0.012570961 | 4.173428002 |
| MPP3 | 0.012591786 | 0.805053074 |
| LA16c-380H5.2 | 0.05079388 | 1.85982076 |
| AC073257.2 | 0.028145138 | 1.858680975 |
| AC072031.1 | 0.063357113 | 1.858202353 |
| U1 | 0.068802736 | 1.857568653 |
| TM4SF19 | 0.01261104 | 0.447434715 |
| RNVU1-20 | 0.019326585 | 1.856772456 |
| DUSP8P4 | 0.092727734 | 1.856586931 |
| CNIH2 | 0.012634797 | 0.636323688 |
| PCBP3-OT1 | 0.060794978 | 1.856435476 |
| MIR320D1 | 0.133784635 | 1.856118519 |
| RNU6-696P | 0.100271164 | 1.855568748 |
| AC011498.1 | 0.013238507 | 1.85547059 |
| RP11-276H19.1 | 0.004391813 | 1.855352005 |
| CH17-140K24.3 | 0.128202106 | 1.854403292 |
| Y_RNA | 0.017693246 | 1.85409415 |
| AC016772.4 | 0.139347707 | 1.853327465 |
| AC000124.1 | 0.104066952 | 1.853295256 |
| KRBOX1-AS1 | 0.124029595 | 1.851169402 |
| EIF4E3 | 0.012645195 | 1.239071533 |
| C3orf38 | 0.012677765 | 1.157725527 |
| ARHGEF38-IT1 | 0.068158645 | 1.850162992 |
| RP11-588G21.2 | 0.04527342 | 1.850106704 |
| RP11-407B7.3 | 0.04703012 | 1.849724711 |
| RP11-100G15.7 | 0.068364312 | 1.849487279 |
| SEC24D | 0.012681954 | 1.275711819 |
| RP11-387H17.6 | 0.016613927 | 1.849024729 |
| RP11-700H6.2 | 0.060408972 | 1.847868922 |
| CTD-2516K3.3 | 0.090970088 | 1.847863395 |
| RP11-403I13.4 | 0.093850532 | 1.847158255 |
| CCDC58P5 | 0.141101561 | 1.847118446 |
| SEPSECS | 0.012692472 | 1.211255123 |
| RP4-555D20.1 | 0.049265682 | 1.846152095 |
| FBXL4 | 0.012717856 | 1.187521106 |
| SIRT1 | 0.012723227 | 1.214899592 |
| BMPR1B | 0.012735984 | 2.045208197 |
| RP1-292B18.3 | 0.036473866 | 1.845395386 |
| RP11-546K22.1 | 0.039632195 | 1.845348971 |
| RP11-762B21.5 | 0.101721305 | 1.845041901 |
| CTD-2126E3.3 | 0.025631762 | 1.844868127 |
| RP11-757O6.1 | 0.073529996 | 1.844432466 |
| RP11-968O1.5 | 0.001768439 | 1.844185618 |
| AHNAK2 | 0.012764923 | 0.677981577 |
| RP11-570L14.1 | 0.134284683 | 1.843182549 |
| CTC-507E2.2 | 0.099701201 | 1.842081148 |
| IL13RA2 | 0.012766228 | 2.057287269 |
| PF4V1 | 0.012810758 | 5.42934028 |
| RNU4ATAC12P | 0.027777152 | 1.839427102 |
| KB-1589B1.4 | 0.072277818 | 1.839323266 |
| CTD-2012J19.2 | 0.01255378 | 1.838714623 |
| GPR135 | 0.012866592 | 1.442856471 |
| RP11-326C3.15 | 0.043475911 | 1.837526358 |
| RP11-963H4.3 | 0.109870045 | 1.837175986 |
| ZNF664 | 0.012873368 | 1.1975855 |
| FMO8P | 0.150083239 | 1.83620252 |
| RP11-576N17.2 | 0.009933199 | 1.836149744 |
| ZNF182 | 0.012889562 | 1.211819175 |
| RP11-403A21.3 | 0.14736332 | 1.835596833 |
| RP3-331H24.7 | 0.01820471 | 1.834537255 |
| RP11-354K1.2 | 0.07973233 | 1.834423003 |
| PGBD4P7 | 0.004356289 | 1.834187067 |
| HIGD1AP12 | 0.141878493 | 1.834109076 |
| RP5-1033H22.2 | 0.069089333 | 1.833679954 |
| SGOL1 | 0.012890115 | 1.210843996 |
| ASB9 | 0.012900702 | 1.778623353 |
| MAML2 | 0.012952876 | 1.35077456 |
| AC002069.5 | 0.03587192 | 1.832172213 |
| AP001626.2 | 0.143922619 | 1.832170891 |
| RP11-386M24.9 | 0.0490066 | 1.832156999 |
| RP11-497G19.3 | 0.063769355 | 1.831631922 |
| RP4-781K5.5 | 0.035092049 | 1.831536367 |
| LUC7L | 0.01295415 | 1.184941298 |
| IVL | 0.012977985 | 0.412732845 |
| SUMO4 | 0.012995455 | 1.36030784 |
| RNU6-833P | 0.080306277 | 1.83007242 |
| USPL1 | 0.013017148 | 1.155910007 |
| RP11-10J21.4 | 0.00167339 | 1.829936694 |
| RBPMS2 | 0.013035512 | 1.583747655 |
| C1orf210 | 0.013049823 | 1.381765198 |
| CDK8 | 0.013109825 | 1.162030593 |
| CYP11A1 | 0.013127531 | 3.439007649 |
| CTD-2200P10.1 | 0.058929273 | 1.828834928 |
| COLCA1 | 0.081397998 | 1.828537854 |
| RAI2 | 0.013191133 | 1.571655775 |
| RP1-77H15.1 | 0.056990907 | 1.828163351 |
| C12orf80 | 0.010921509 | 1.827844715 |
| LRRC66 | 0.013202079 | 1.991299053 |
| AC010974.3 | 0.108587592 | 1.825522258 |
| RP11-771K4.3 | 0.00080093 | 1.825153067 |
| RALY | 0.013202136 | 0.816959774 |
| IQCK | 0.013239441 | 1.31129422 |
| DSC2 | 0.013257999 | 0.576135742 |
| ARL5AP3 | 0.131615191 | 1.823310272 |
| RP11-37L2.1 | 0.004570555 | 1.822266241 |
| ABCC6P2 | 0.084046262 | 1.821686191 |
| UQCRBP2 | 0.094153351 | 1.821635028 |
| CTD-2245E15.3 | 0.058446491 | 1.821600887 |
| CXXC1P1 | 0.143419065 | 1.821227496 |
| ZBTB20-AS4 | 0.006974337 | 1.820409995 |
| TVP23C | 0.013281153 | 1.255764842 |
| TUBBP9 | 0.087590577 | 1.820066405 |
| CTC-347C20.2 | 0.102644645 | 1.819958702 |
| DRAXIN | 0.013294012 | 2.237837348 |
| INHBA-AS1 | 0.032930673 | 1.819379129 |
| BASP1 | 0.013298938 | 2.155107223 |
| CTD-2008P7.10 | 0.024654757 | 1.819311855 |
| EFCAB2 | 0.013314069 | 1.328365936 |
| AC122718.1 | 0.096561415 | 1.81891449 |
| NISCH | 0.013377811 | 1.180495888 |
| ANXA3 | 0.013389646 | 1.39352485 |
| RP11-416I2.1 | 0.102469715 | 1.818514487 |
| RNU6-677P | 0.059930746 | 1.818067574 |
| RP11-1109F11.3 | 0.001016142 | 1.817712408 |
| RP11-57C13.6 | 0.005719553 | 1.817573787 |
| HMGB1P27 | 0.076845131 | 1.817302869 |
| CCDC176 | 0.013436341 | 1.329165992 |
| ZXDA | 0.01344121 | 1.258309225 |
| MIR31 | 0.13695262 | 1.815553496 |
| LINC01111 | 0.13568665 | 1.815128509 |
| ANXA5 | 0.013480178 | 1.19344207 |
| AGAP7P | 0.022615399 | 1.814290063 |
| KIAA1143 | 0.013482231 | 1.159306004 |
| KCTD15 | 0.013483785 | 0.755174783 |
| IL5RA | 0.013501779 | 2.841973 |
| COL24A1 | 0.013518263 | 1.612873452 |
| NUBP1 | 0.013535796 | 1.232752164 |
| RP11-159D12.3 | 0.058294655 | 1.809686823 |
| MYADM | 0.013545511 | 1.630570488 |
| AP001631.9 | 0.015909885 | 1.809117947 |
| RP11-710C12.1 | 0.099530784 | 1.807901607 |
| RP11-270M14.5 | 0.00873929 | 1.807884906 |
| CTD-2532N20.1 | 0.055578563 | 1.807785093 |
| RP4-684O24.5 | 0.14939936 | 1.807229563 |
| AC067940.1 | 0.075555776 | 1.807179806 |
| SNRPCP16 | 0.061784111 | 1.806663207 |
| RNA5SP123 | 0.056761774 | 1.806265386 |
| RP11-112J3.16 | 0.031509227 | 1.806263493 |
| HMGB3P2 | 0.060236493 | 1.805752192 |
| SERPINB5 | 0.013600016 | 0.639905067 |
| FRAS1 | 0.013606602 | 1.727036049 |
| RNU6-937P | 0.08020463 | 1.804445579 |
| MIR4530 | 0.070708792 | 1.803678811 |
| AC078941.1 | 0.040966527 | 1.803550203 |
| RP11-380I10.2 | 0.107301828 | 1.802841997 |
| RNU6-403P | 0.114468283 | 1.802624818 |
| RP4-565E6.1 | 0.017176861 | 1.802609264 |
| AC064852.4 | 0.131501568 | 1.802507828 |
| MAPK9 | 0.013645494 | 1.143276935 |
| AC004066.2 | 0.135076915 | 1.802182201 |
| LMBRD1 | 0.013665541 | 1.16569689 |
| RNU6-1315P | 0.115844231 | 1.801810751 |
| RGS8 | 0.013688498 | 1.6732244 |
| HNRNPMP1 | 0.023569364 | 1.801286959 |
| OR1F2P | 0.067393114 | 1.800841115 |
| CTAGE5 | 0.013698946 | 1.24004498 |
| RP5-1174J21.2 | 0.083568129 | 1.79978806 |
| INSIG2 | 0.013749013 | 1.162168451 |
| PTP4A1P2 | 0.141904761 | 1.799373447 |
| NANOS3 | 0.013772911 | 1.654054777 |
| MANF | 0.013790934 | 1.20928278 |
| FLT1P1 | 0.102016523 | 1.797761433 |
| CCDC65 | 0.013791195 | 1.581463172 |
| U6 | 0.102676697 | 1.797175347 |
| RPL21P122 | 0.023768004 | 1.797104897 |
| RP11-252A24.8 | 0.10388727 | 1.796224769 |
| LINC01585 | 0.00257107 | 1.795674906 |
| THAP9 | 0.01381745 | 1.180921131 |
| RP11-161H23.9 | 0.020106154 | 1.794613657 |
| FRMD4B | 0.013830564 | 1.280733702 |
| SUPT20H | 0.013836515 | 1.174672444 |
| SEPT7P8 | 0.103539044 | 1.793879983 |
| RP1-127H14.3 | 0.002612597 | 1.793848148 |
| TMEM159 | 0.01384499 | 0.798243534 |
| RP11-143J12.3 | 0.03585314 | 1.792792952 |
| TTC23L | 0.013862354 | 1.571290096 |
| RP11-96C23.9 | 0.05067906 | 1.792735212 |
| MYO10 | 0.013874392 | 1.295579395 |
| MAP4K2 | 0.013883497 | 0.849412324 |
| MIR4469 | 0.053749111 | 1.791589257 |
| SH2D6 | 0.013955477 | 2.220774843 |
| NHS | 0.013993723 | 1.441992163 |
| KLHL32 | 0.01405696 | 2.17933896 |
| CTD-2199O4.1 | 0.06462966 | 1.790543324 |
| ENPP7P7 | 0.032347081 | 1.790188149 |
| MAP3K7 | 0.014071392 | 1.124695474 |
| TSC22D1 | 0.014135841 | 1.368583681 |
| LINC00987 | 0.070020618 | 1.789403396 |
| BMS1P10 | 0.017458183 | 1.78910865 |
| AC083843.2 | 0.057795136 | 1.788808614 |
| RP11-673E1.1 | 0.042510538 | 1.788587278 |
| AMZ2P2 | 0.057401192 | 1.788474791 |
| RP11-149I9.2 | 0.066315274 | 1.788447986 |
| CALR | 0.014136002 | 1.139758603 |
| RNF150 | 0.014142084 | 2.30326246 |
| LYPD3 | 0.014157407 | 0.45828174 |
| C4orf46 | 0.014187236 | 1.188285437 |
| SVIP | 0.014214642 | 1.366050847 |
| RP11-700H6.4 | 0.007719853 | 1.786573955 |
| TRMT61A | 0.014220169 | 0.862125126 |
| Y_RNA | 0.046989903 | 1.785357373 |
| CCNG1 | 0.014226571 | 1.204023991 |
| AC092574.1 | 0.012711611 | 1.784393069 |
| RNF187 | 0.014230047 | 0.872088806 |
| RN7SL125P | 0.097197304 | 1.783976817 |
| RP13-565O16.2 | 0.069491505 | 1.783703791 |
| TMEM92 | 0.014250233 | 1.573595506 |
| SPATA20P1 | 0.007608041 | 1.782869835 |
| BMS1P7 | 0.028328199 | 1.782752585 |
| RP3-431P23.5 | 0.013690375 | 1.782600557 |
| RN7SL745P | 0.032496638 | 1.782052224 |
| PTGES2-AS1 | 0.029474255 | 1.782034928 |
| RP11-63P12.7 | 0.124959462 | 1.781721618 |
| CTD-2278I10.1 | 0.123324218 | 1.781706159 |
| AC011499.1 | 0.082965299 | 1.781078679 |
| STIM2 | 0.014257149 | 1.222027789 |
| WDR49 | 0.014276262 | 3.722610125 |
| LINC01515 | 0.007484626 | 1.777017625 |
| TPRG1L | 0.014284678 | 0.841016854 |
| MT1M | 0.014296022 | 2.420358966 |
| RP11-219G17.4 | 0.088404232 | 1.776634318 |
| DNALI1 | 0.014309813 | 2.336461579 |
| C6orf165 | 0.014312611 | 2.401811204 |
| RP11-335I12.2 | 0.05768291 | 1.775773521 |
| RP11-479G22.8 | 0.012287671 | 1.774501992 |
| RP11-132N15.3 | 0.081941684 | 1.7744077 |
| LINC01050 | 0.049594187 | 1.774191951 |
| C1orf194 | 0.014314613 | 3.502531056 |
| RNU6-1010P | 0.030439457 | 1.772794851 |
| SNORA19 | 0.070416805 | 1.772012045 |
| C20orf197 | 0.129633369 | 1.77179957 |
| RP11-130C6.1 | 0.113111266 | 1.771648913 |
| NIFKP6 | 0.007927715 | 1.771382735 |
| ATP6V0E1P2 | 0.057095297 | 1.770613769 |
| RP5-828H9.1 | 0.121937691 | 1.770406589 |
| TRBV8-2 | 0.027300386 | 1.770327608 |
| AVPI1 | 0.014354412 | 1.406148661 |
| RP11-713C5.1 | 0.121056905 | 1.769995609 |
| AC115115.3 | 0.07869546 | 1.769791635 |
| RP11-245P10.4 | 0.007205591 | 1.768618117 |
| RP11-48B3.4 | 0.015701472 | 1.767337038 |
| USP38 | 0.014361594 | 1.177357536 |
| LINC00525 | 0.102364811 | 1.767029987 |
| CSGALNACT1 | 0.014386791 | 1.514093997 |
| YDJC | 0.014399609 | 0.791323453 |
| USP5 | 0.01446048 | 0.832211775 |
| SNORA19 | 0.067892501 | 1.765368097 |
| ZBBX | 0.014472444 | 7.739276538 |
| TRMT2B-AS1 | 0.075578565 | 1.764143732 |
| ORAOV1P1 | 0.075421875 | 1.764067254 |
| KRT222 | 0.014487294 | 1.726428162 |
| MRPS36P2 | 0.117610392 | 1.76372825 |
| FGFR1OP | 0.01453952 | 1.204976301 |
| HGSNAT | 0.014541721 | 1.194128201 |
| SALRNA2 | 0.077043273 | 1.761451858 |
| RP11-433C9.2 | 0.095193272 | 1.76135212 |
| RP11-153K11.3 | 0.099903191 | 1.761056235 |
| Y_RNA | 0.075028428 | 1.761035072 |
| CTC-563A5.4 | 0.148420903 | 1.760410813 |
| AC007618.3 | 0.136524858 | 1.759849123 |
| SNORD70 | 0.088160312 | 1.759745178 |
| LINC00694 | 0.07977278 | 1.759723909 |
| RP11-326K13.3 | 0.142115199 | 1.759676863 |
| FAM102A | 0.014594452 | 0.783043887 |
| RP11-764K9.4 | 0.04891774 | 1.758550672 |
| RP11-399K21.12 | 0.149140295 | 1.75849792 |
| AQP6 | 0.014700552 | 1.972892449 |
| RP11-474B12.1 | 0.045067852 | 1.757665401 |
| PIGR | 0.014705829 | 3.182204944 |
| FOXP1-AS1 | 0.001394367 | 1.757498198 |
| WBP4 | 0.014726747 | 1.142881059 |
| RP11-472M19.2 | 0.050525029 | 1.757139125 |
| RAE1 | 0.014764798 | 0.864671891 |
| NHS-AS1 | 0.084217693 | 1.75601328 |
| NBPF14 | 0.014798269 | 0.772501107 |
| RP11-775D22.2 | 0.018349932 | 1.754681919 |
| LEPR | 0.014870838 | 1.425013811 |
| RP11-267N12.2 | 0.081510006 | 1.753207838 |
| RNA5SP101 | 0.144078117 | 1.753185856 |
| NDFIP1 | 0.014918334 | 1.113670573 |
| RPSAP51 | 0.099881168 | 1.751976776 |
| MAN2B2 | 0.014920749 | 1.209066864 |
| RP11-713N11.6 | 0.032625824 | 1.750739591 |
| C9orf50 | 0.014927279 | 1.671760153 |
| NHLRC4 | 0.014941818 | 1.816273321 |
| CHCHD4P4 | 0.080731727 | 1.750184923 |
| AC092580.1 | 0.024962682 | 1.749860921 |
| MIR3188 | 0.090051118 | 1.749793576 |
| UBR1 | 0.014945742 | 1.208987934 |
| RP1-167G20.1 | 0.087811093 | 1.749492534 |
| ACOT4 | 0.014972322 | 1.296292878 |
| PPP1R42 | 0.014983692 | 2.935620959 |
| SNORA75 | 0.068644327 | 1.749161797 |
| RP11-883G14.1 | 0.091352755 | 1.748860078 |
| EEF1DP7 | 0.042657309 | 1.74885883 |
| RPL21P127 | 0.048169989 | 1.747787446 |
| ZNF750 | 0.014992657 | 0.657791782 |
| SLC16A12 | 0.015016522 | 12.84771422 |
| AC093826.1 | 0.005841936 | 1.746051205 |
| RNU6-228P | 0.101002704 | 1.745509455 |
| HMGB1 | 0.015081784 | 1.127775135 |
| TFR2 | 0.015088767 | 0.67776461 |
| HMGN1P28 | 0.006020836 | 1.743831977 |
| SNORA74B | 0.076613966 | 1.743076953 |
| DNAH6 | 0.015112547 | 2.399493295 |
| RP11-353N14.2 | 0.023225618 | 1.742971726 |
| RP11-525K10.3 | 0.012448238 | 1.742750975 |
| QPCT | 0.015147411 | 2.051317391 |
| CNOT6 | 0.015187317 | 1.124360926 |
| RP11-234G16.5 | 0.026892619 | 1.742507733 |
| TNXA | 0.07197746 | 1.742237361 |
| DLX2-AS1 | 0.141189963 | 1.741948811 |
| KIAA0895 | 0.015235536 | 1.332117796 |
| RP11-489G11.3 | 0.035605646 | 1.741898419 |
| SACS-AS1 | 0.067920876 | 1.741327524 |
| RP11-856M7.6 | 0.10266821 | 1.741117072 |
| RN7SL390P | 0.120482678 | 1.740709312 |
| CTA-331P3.1 | 0.115802507 | 1.740621893 |
| PPA1 | 0.015266807 | 1.188070255 |
| MIR544B | 0.067352539 | 1.740275331 |
| RN7SL825P | 0.109241064 | 1.739874626 |
| RP11-10K17.3 | 0.125322304 | 1.73977858 |
| PARP3 | 0.01526689 | 1.264818037 |
| MCCD1P1 | 0.061755067 | 1.739551752 |
| AC133965.1 | 0.133932877 | 1.739464396 |
| GLYCTK-AS1 | 0.013111436 | 1.739432558 |
| RP11-459C13.1 | 0.13440492 | 1.738792588 |
| SLC35E1P1 | 0.018813181 | 1.738681184 |
| SAPCD1-AS1 | 0.005518545 | 1.738537734 |
| CH17-408M7.1 | 0.03621286 | 1.738196111 |
| HR | 0.015336095 | 0.63594951 |
| PIGU | 0.015404461 | 0.791385777 |
| RP11-36N20.1 | 0.093539328 | 1.737600219 |
| RP11-354E23.4 | 0.072449091 | 1.737513281 |
| RP11-109D20.1 | 0.075212531 | 1.737068721 |
| AP001271.3 | 0.090930235 | 1.736831225 |
| CSNK1D | 0.01543416 | 1.102941381 |
| MICF | 0.078111732 | 1.736423464 |
| RP11-119F7.5 | 0.041745225 | 1.736294684 |
| RP11-195L15.2 | 0.013289184 | 1.736152059 |
| RP11-428G5.5 | 0.023813756 | 1.735871573 |
| WDR74 | 0.015438066 | 0.836108606 |
| BTBD2 | 0.015446453 | 0.861196687 |
| NOS1AP | 0.015472189 | 1.429407973 |
| STK32A | 0.015479829 | 3.115575617 |
| TPT1P5 | 0.003237458 | 1.732816523 |
| RP11-576N17.3 | 0.036823132 | 1.732647584 |
| RP11-47I22.2 | 0.004638196 | 1.732513612 |
| CD177P1 | 0.136967833 | 1.732370632 |
| RP11-1260E13.2 | 0.122828006 | 1.732350927 |
| RP3-512E2.2 | 0.039694082 | 1.732189158 |
| RBMS3-AS3 | 0.055223771 | 1.731350189 |
| HOXC5 | 0.01550901 | 2.247871962 |
| DENND2C | 0.015514938 | 0.633479746 |
| RP11-1396O13.2 | 0.022303812 | 1.730577353 |
| AC006116.13 | 0.010335633 | 1.730324634 |
| FRMPD2L2 | 0.116914979 | 1.730211819 |
| AC008697.1 | 0.041902943 | 1.730143619 |
| CFAP70 | 0.015603582 | 2.11262098 |
| CICP23 | 0.105722269 | 1.729103289 |
| RP11-196H14.4 | 0.111443487 | 1.72850441 |
| ZNF330 | 0.015607953 | 1.140067761 |
| TAS2R63P | 0.041229251 | 1.727620095 |
| PRKAR1A | 0.015616961 | 1.163191328 |
| LMNTD1 | 0.015621416 | 11.00295235 |
| ZNF774 | 0.015698468 | 1.41448732 |
| HOMER2 | 0.015704893 | 1.450287398 |
| CLASP2 | 0.015706519 | 1.183435934 |
| RP11-242C24.2 | 0.046020692 | 1.726193292 |
| CTD-3105H18.8 | 0.032237912 | 1.725364986 |
| CTD-2282P23.2 | 0.066232069 | 1.725239799 |
| AC010884.1 | 0.082957277 | 1.724884129 |
| SEMA3B-AS1 | 0.140554262 | 1.724863431 |
| RP4-680D5.9 | 0.057894934 | 1.72447053 |
| CSPG4 | 0.01570963 | 1.759286346 |
| RP3-388N13.5 | 0.035818172 | 1.724230023 |
| RP11-3J1.1 | 0.143925871 | 1.723816319 |
| AC099684.1 | 0.150157603 | 1.723803162 |
| RP11-826F13.1 | 0.026288173 | 1.723777179 |
| AC008753.6 | 0.075467995 | 1.723662405 |
| KRT18P25 | 0.030662244 | 1.722892785 |
| RP11-1109F11.5 | 0.0022609 | 1.722070551 |
| AC009303.3 | 0.010587175 | 1.721985771 |
| MYOM1 | 0.015786875 | 1.776728648 |
| CCDC7 | 0.015859163 | 1.349181925 |
| RP11-8P13.5 | 0.03851966 | 1.721573127 |
| RP11-119H12.1 | 0.021646854 | 1.721501281 |
| RP11-556N21.1 | 0.064524544 | 1.72046069 |
| RP11-815M8.1 | 0.09081429 | 1.719993048 |
| DNAJC6 | 0.015891877 | 1.66839371 |
| WDR60 | 0.015904527 | 1.210534312 |
| RP5-875H18.4 | 0.05814313 | 1.719485512 |
| RP11-158I9.5 | 0.019138592 | 1.719365643 |
| RP11-544D21.2 | 0.100162889 | 1.719014176 |
| C1orf27 | 0.015950068 | 1.147786627 |
| CTD-2027I19.2 | 0.016105178 | 1.718416909 |
| RP5-875O13.7 | 0.135932984 | 1.718347575 |
| MIR563 | 0.10481776 | 1.717833215 |
| RP11-627G18.2 | 0.132167785 | 1.717634506 |
| RP11-686D22.6 | 0.061281461 | 1.716890479 |
| RP11-85L21.4 | 0.142656983 | 1.716326254 |
| ADH7 | 0.015971737 | 0.419374764 |
| DDAH1 | 0.015986412 | 1.60375117 |
| AC006042.6 | 0.009610944 | 1.715221346 |
| PWWP2A | 0.015987154 | 1.15631548 |
| C8orf82 | 0.016014477 | 0.759085309 |
| EFTUD1P1 | 0.073480238 | 1.714207078 |
| MTND1P32 | 0.130935931 | 1.713754341 |
| Y_RNA | 0.095435843 | 1.713655734 |
| RP11-204E9.1 | 0.086674298 | 1.713479993 |
| PTBP1P | 0.063791175 | 1.713173609 |
| GFPT1 | 0.016018553 | 1.403357453 |
| CTD-3157E16.2 | 0.001768375 | 1.711618629 |
| AC009303.1 | 0.128388124 | 1.711427219 |
| RP11-735A19.3 | 0.05147771 | 1.711393014 |
| LINC01449 | 0.062973286 | 1.711361769 |
| RP11-15P13.1 | 0.107710911 | 1.711291448 |
| TMIE | 0.01602857 | 1.730717812 |
| FECH | 0.016060681 | 0.833258239 |
| FREM2 | 0.016086706 | 2.337576194 |
| GLYCTK | 0.016117858 | 1.739712362 |
| AXIN1 | 0.016144065 | 1.125697675 |
| RP11-728K20.1 | 0.001979489 | 1.709087467 |
| RP11-517P14.7 | 0.09091039 | 1.708220474 |
| CDH24 | 0.016190105 | 0.819936051 |
| RP5-979D14.1 | 0.098333154 | 1.708129573 |
| RP3-523C21.2 | 0.029581041 | 1.70798152 |
| AC079776.3 | 0.145765616 | 1.706841768 |
| AADAT | 0.016208856 | 1.441654533 |
| RP5-856G1.1 | 0.026961909 | 1.706485363 |
| CCDC170 | 0.016262543 | 2.70401381 |
| SMR3B | 0.016274749 | 4.758207555 |
| RP1-232L24.2 | 0.071771686 | 1.70592502 |
| RLIMP1 | 0.014885856 | 1.705810206 |
| TUBB8P2 | 0.017935063 | 1.70470517 |
| AC100821.1 | 0.121783487 | 1.704453848 |
| NOTCH2 | 0.016306838 | 0.754008265 |
| PRR26 | 0.016333252 | 2.420031797 |
| NEK3 | 0.016346801 | 1.267919439 |
| PLCB2-AS1 | 0.11390022 | 1.703264601 |
| RP11-83A16.1 | 0.131528161 | 1.702647088 |
| LINC00323 | 0.043181051 | 1.702584418 |
| RP11-230G5.2 | 0.084546721 | 1.702430227 |
| RPS3AP15 | 0.086518892 | 1.702115013 |
| OR5M11 | 0.016353712 | 0.227209756 |
| RP4-744I24.4 | 0.016747064 | 1.7016776 |
| NFS1 | 0.016361701 | 0.850286501 |
| LINC00856 | 0.056396821 | 1.701430713 |
| KLRF1 | 0.016375687 | 1.766804689 |
| RP11-507K2.2 | 0.004879994 | 1.700399686 |
| TCTN2 | 0.016410598 | 1.224193127 |
| Y_RNA | 0.139084977 | 1.699846771 |
| RP11-54C4.2 | 0.041239345 | 1.699779217 |
| LINC00336 | 0.037503971 | 1.699192282 |
| C9orf171 | 0.016579836 | 4.425815961 |
| RNU6-1079P | 0.070723822 | 1.698414426 |
| MRFAP1 | 0.016579854 | 1.137946311 |
| SLC6A2 | 0.016636019 | 3.534926526 |
| C10orf91 | 0.10745555 | 1.697967404 |
| RANBP17 | 0.016667648 | 1.413150293 |
| CTBP2 | 0.016681791 | 1.184744409 |
| RP11-356M20.2 | 0.058420929 | 1.697225823 |
| AP000783.1 | 0.085449035 | 1.696770457 |
| RP11-295G24.4 | 0.055176398 | 1.69671635 |
| SCARB2 | 0.016698752 | 1.187211781 |
| RP11-65J21.1 | 0.106153019 | 1.696223451 |
| GS1-165B14.2 | 0.089977415 | 1.695904132 |
| AP000354.4 | 0.075587836 | 1.695814268 |
| HIVEP3 | 0.016780838 | 0.707570237 |
| RP11-1100L3.8 | 0.037139915 | 1.695302252 |
| EAF1 | 0.016793049 | 1.178636564 |
| WDR48 | 0.016799739 | 1.159478864 |
| SRSF8 | 0.016822068 | 1.204293199 |
| AC004510.3 | 0.144720562 | 1.693612359 |
| ZNF542P | 0.016889775 | 1.69355489 |
| RP11-524F11.3 | 0.017587662 | 1.69354479 |
| LPGAT1 | 0.01692121 | 1.441489879 |
| CTB-158D10.3 | 0.144817035 | 1.692488565 |
| HOXB9 | 0.016923018 | 2.011929292 |
| SNORD19B | 0.107178015 | 1.690861201 |
| AL139398.1 | 0.069712346 | 1.690770724 |
| RP11-6I2.3 | 0.037300368 | 1.690434168 |
| RP11-884K10.6 | 0.133818149 | 1.69027551 |
| RP11-350N15.6 | 0.116122266 | 1.689814437 |
| QRICH1 | 0.016926321 | 1.140524003 |
| TMEM31 | 0.016934995 | 1.650574736 |
| MIR548AN | 0.068460763 | 1.689235172 |
| AL450226.2 | 0.10712238 | 1.688940479 |
| KLHL30-AS1 | 0.137796975 | 1.688011325 |
| IPPK | 0.01700056 | 0.716425291 |
| OR52V1P | 0.149778664 | 1.687497117 |
| ITGA1 | 0.017006992 | 1.599392222 |
| RP11-350J20.9 | 0.132974115 | 1.686612754 |
| ACY3 | 0.017048098 | 2.682823726 |
| RTCB | 0.017128679 | 0.878068239 |
| RP11-47J17.2 | 0.008273016 | 1.684336275 |
| RP11-564C24.1 | 0.115382352 | 1.683554142 |
| RP11-34D15.2 | 0.11813673 | 1.68300984 |
| SMYD1 | 0.017179048 | 3.494514843 |
| TMEM42 | 0.017190594 | 1.17112201 |
| AIRN | 0.048574699 | 1.68256184 |
| RPL19P14 | 0.140735156 | 1.682473427 |
| RNA5SP507 | 0.077499939 | 1.682243617 |
| RP11-20L24.1 | 0.035445916 | 1.681808125 |
| RP11-1081L13.4 | 0.005920613 | 1.681535561 |
| LL0XNC01-221F2.2 | 0.148728996 | 1.681366229 |
| LINC00313 | 0.02623686 | 1.681234628 |
| RP11-705C15.4 | 0.013720768 | 1.681168354 |
| RP11-90D4.4 | 0.139313255 | 1.680845864 |
| ATOH8 | 0.017222129 | 1.588968763 |
| AC131011.1 | 0.06303505 | 1.680453654 |
| POLR3F | 0.017233714 | 0.8477982 |
| AC114763.1 | 0.105009492 | 1.6799091 |
| RP11-532F6.3 | 0.003978774 | 1.679774687 |
| AC005592.2 | 0.035104084 | 1.679702305 |
| RP11-75C10.7 | 0.068332621 | 1.679616983 |
| AC007392.3 | 0.07900903 | 1.679502618 |
| TYRO3 | 0.017245481 | 0.819537106 |
| PNCK | 0.017322962 | 1.998055811 |
| OR10A4 | 0.017406954 | 4.407377083 |
| RNU5D-1 | 0.119868633 | 1.677860504 |
| RP11-352M15.1 | 0.148382694 | 1.677638501 |
| GABARAPL3 | 0.031304416 | 1.677432034 |
| AP000721.1 | 0.135953822 | 1.676969008 |
| RP11-400N9.1 | 0.033420455 | 1.676628424 |
| RP11-267M23.6 | 0.009706774 | 1.676539204 |
| RP11-861E21.1 | 0.005670797 | 1.676467453 |
| RP11-492D6.3 | 0.089601352 | 1.676057939 |
| RP11-241K18.2 | 0.08885756 | 1.675424429 |
| RP11-301L8.2 | 0.116816223 | 1.675344239 |
| RP11-727A23.8 | 0.149290235 | 1.675204747 |
| KCNQ1-AS1 | 0.072098704 | 1.673988234 |
| ZSCAN20 | 0.017414176 | 1.184312947 |
| TRIM68 | 0.017458891 | 1.307254955 |
| RP11-166N6.1 | 0.045269878 | 1.673052301 |
| KIF28P | 0.009094911 | 1.672513196 |
| ERRFI1 | 0.017487885 | 1.422245404 |
| LHX2 | 0.017495883 | 0.607279989 |
| MAP3K19 | 0.017521002 | 7.56220546 |
| RP11-667K14.13 | 0.130857046 | 1.671744036 |
| GS1-293C5.1 | 0.006161884 | 1.671332002 |
| ZFHX3 | 0.017551765 | 1.283069557 |
| RP5-849H19.2 | 0.008470877 | 1.670858328 |
| RP1-290I10.7 | 0.007467713 | 1.670635599 |
| AC018761.1 | 0.102026116 | 1.670216972 |
| CTC-248O19.1 | 0.136553223 | 1.670122774 |
| MIR3180-4 | 0.127618507 | 1.670117471 |
| CTD-2017D11.2 | 0.011867547 | 1.669908041 |
| CTD-2005H7.2 | 0.016483588 | 1.669642184 |
| SUSD4 | 0.017608391 | 0.55577153 |
| RP11-7F17.3 | 0.120121891 | 1.66954379 |
| CTD-3236F5.1 | 0.014587755 | 1.669445874 |
| ABHD14A | 0.017610208 | 1.335134164 |
| TMEM184C | 0.017637788 | 1.165053549 |
| CSNK1G2 | 0.017638894 | 0.886782994 |
| AC007228.5 | 0.124437635 | 1.668253013 |
| RN7SL16P | 0.139498975 | 1.667012873 |
| CISH | 0.017660506 | 1.338271403 |
| RP4-742J24.2 | 0.065094728 | 1.665430716 |
| RPL7P27 | 0.074593974 | 1.664939156 |
| RPSAP56 | 0.095601623 | 1.66488291 |
| ADAMTS6 | 0.017663917 | 1.963830824 |
| CTD-3105H18.7 | 0.038801725 | 1.664288498 |
| RNY1P16 | 0.029913702 | 1.664267662 |
| RNF133 | 0.017691308 | 1.515598499 |
| WT1-AS | 0.073802021 | 1.663481541 |
| TSPAN12 | 0.017747924 | 1.627553061 |
| CRY1 | 0.01775237 | 1.212794371 |
| LSMEM2 | 0.01781624 | 2.586845628 |
| SENCR | 0.092702725 | 1.66204418 |
| FAM166B | 0.017878705 | 2.685105317 |
| CXCL3 | 0.017885071 | 2.071284664 |
| SNORA80B | 0.151052591 | 1.66147105 |
| TMEM41B | 0.01789673 | 1.141486593 |
| RP11-489M13.1 | 0.082841312 | 1.661050804 |
| RP11-16K12.2 | 0.035194651 | 1.660729721 |
| RP3-416H24.1 | 0.010516925 | 1.660603352 |
| APOC4 | 0.017943935 | 4.511790093 |
| RP11-508N22.13 | 0.005766317 | 1.660075079 |
| STC1 | 0.017963043 | 3.173544719 |
| OGG1 | 0.017974327 | 1.161981895 |
| GTPBP2 | 0.018050813 | 1.168836961 |
| RP11-34A14.3 | 0.081360255 | 1.658059163 |
| TMEM231 | 0.018130936 | 1.636994552 |
| ZDHHC20 | 0.018150476 | 1.174605023 |
| RP11-242D8.2 | 0.004986822 | 1.656938281 |
| RP11-285E23.2 | 0.098543538 | 1.656268249 |
| CTD-2083E4.4 | 0.031275229 | 1.656162063 |
| CFAP61 | 0.018154113 | 2.207756118 |
| uc_338 | 0.008845324 | 1.655759417 |
| CNNM1 | 0.018158161 | 0.560242622 |
| CLK3 | 0.018165723 | 1.097852387 |
| TEC | 0.018185059 | 1.311590191 |
| DBNL | 0.018200463 | 0.872636252 |
| RP11-73M11.3 | 0.062089121 | 1.654943995 |
| STEAP2-AS1 | 0.054874854 | 1.654649282 |
| RP11-166P13.4 | 0.051831952 | 1.654422407 |
| RPL17P43 | 0.019881655 | 1.654395103 |
| SPATA6 | 0.018219065 | 1.451254282 |
| AL590762.7 | 0.009856825 | 1.65361941 |
| GOLPH3 | 0.018261856 | 1.201497036 |
| RNU5A-1 | 0.043351205 | 1.653154125 |
| XXbac-BPG299F13.14 | 0.14096587 | 1.652839839 |
| CATIP | 0.018264964 | 3.389753691 |
| RP11-654C22.2 | 0.149217656 | 1.651716809 |
| ITPK1-AS1 | 0.019489933 | 1.651367327 |
| RNA5SP465 | 0.146770051 | 1.651252326 |
| RP11-214N15.5 | 0.107080575 | 1.650873711 |
| CALD1 | 0.018316172 | 1.335166675 |
| RCC2P6 | 0.048046542 | 1.650741098 |
| DLG3 | 0.018341183 | 1.207102749 |
| C9orf152 | 0.018343066 | 2.141583951 |
| ZNF572 | 0.018427126 | 1.327985668 |
| SPA17P1 | 0.024209067 | 1.649761824 |
| U3 | 0.088447129 | 1.64880433 |
| RP11-243A14.1 | 0.127972202 | 1.648689446 |
| RP11-797A18.5 | 0.090572472 | 1.648391035 |
| C4orf47 | 0.018456261 | 1.702105389 |
| RP11-1124B17.1 | 0.019059264 | 1.647996306 |
| RP5-919F19.5 | 0.055137954 | 1.64760684 |
| TMEM229B | 0.01845722 | 1.456715044 |
| RP11-324D17.3 | 0.110390245 | 1.6470724 |
| ZNF300P1 | 0.089830698 | 1.646971969 |
| RP11-48B3.5 | 0.001312465 | 1.645438888 |
| RP11-519G16.3 | 0.038218462 | 1.645104494 |
| RP11-53B2.4 | 0.035337128 | 1.643921632 |
| KRT18P27 | 0.091155574 | 1.64384936 |
| RP5-1120P11.3 | 0.063011052 | 1.643560356 |
| RP11-573M3.2 | 0.065246548 | 1.643502336 |
| SNX14 | 0.018486965 | 1.202422265 |
| AC012531.25 | 0.085468327 | 1.6423444 |
| RP4-673D20.3 | 0.046288958 | 1.642048127 |
| RP5-875H18.10 | 0.077690985 | 1.641936779 |
| RPS3AP40 | 0.040827783 | 1.641920917 |
| LINC00535 | 0.004381199 | 1.641294647 |
| RP11-728E14.3 | 0.066101796 | 1.640755313 |
| C10orf107 | 0.018495185 | 3.043043441 |
| RN7SL751P | 0.111740185 | 1.640553569 |
| DNAJC9-AS1 | 0.020115604 | 1.640519876 |
| ERICH3 | 0.018496323 | 9.197313083 |
| CTD-2555A7.2 | 0.125851123 | 1.639371549 |
| CTDSPL | 0.018505637 | 1.198328515 |
| EPHX2 | 0.018518619 | 0.67487241 |
| RHOXF2 | 0.018523074 | 7.961522136 |
| AC007405.6 | 0.017661471 | 1.638818364 |
| C8orf44 | 0.018544737 | 1.201889998 |
| PLXNB1 | 0.018563113 | 1.2283857 |
| C21orf91-OT1 | 0.029176481 | 1.637689021 |
| AC002064.5 | 0.126951061 | 1.637574439 |
| CABP4 | 0.018573584 | 1.733171534 |
| HIST2H2BB | 0.130396573 | 1.637377007 |
| TNF | 0.018575141 | 1.827264188 |
| RNU5E-1 | 0.046960616 | 1.636881588 |
| RP11-582J16.3 | 0.100261063 | 1.636309543 |
| ABHD4 | 0.018581896 | 0.749772398 |
| LSINCT5 | 0.123739086 | 1.6355953 |
| BCL2L10 | 0.018599478 | 0.559258947 |
| RP11-443B7.1 | 0.042401927 | 1.634728601 |
| Metazoa_SRP | 0.041476583 | 1.634515002 |
| TMEM128 | 0.018664251 | 1.171710629 |
| CCDC30 | 0.018676646 | 1.444909477 |
| RUSC2 | 0.018685921 | 1.225056422 |
| RBMS2 | 0.018691816 | 1.278159188 |
| KRT7 | 0.018709538 | 1.518323483 |
| MBL1P | 0.029892104 | 1.633265872 |
| CTC-457L16.2 | 0.092562007 | 1.63322026 |
| RP11-104L21.3 | 0.02125911 | 1.633124536 |
| AC013410.1 | 0.109566153 | 1.632049102 |
| LRRC72 | 0.018724217 | 4.949814806 |
| DNAJB7 | 0.01874564 | 1.300240289 |
| STEAP1B | 0.018765404 | 0.519245282 |
| GRK7 | 0.01878332 | 0.640900039 |
| PACRG | 0.018786729 | 2.844392931 |
| RP11-81K2.2 | 0.137012849 | 1.630039606 |
| RP11-444D3.1 | 0.062826226 | 1.630034714 |
| RP11-44F21.4 | 0.143037894 | 1.629937657 |
| RP11-713M15.1 | 0.142217675 | 1.62984476 |
| C1orf168 | 0.018816494 | 2.592217122 |
| RPL32P34 | 0.078302386 | 1.629360237 |
| NBAT1 | 0.133002919 | 1.62916602 |
| AP000688.11 | 0.114307984 | 1.628724215 |
| RP11-326C3.12 | 0.027799893 | 1.628644506 |
| LRRD1 | 0.018827524 | 1.44190984 |
| RP11-76E16.2 | 0.02720466 | 1.628272277 |
| RP11-85I17.2 | 0.00167988 | 1.628197859 |
| RP11-263K19.4 | 0.017323629 | 1.628072849 |
| RP11-54O7.3 | 0.101317076 | 1.627978029 |
| MIR548D2 | 0.04642073 | 1.627798694 |
| LGR4 | 0.018876181 | 1.335463454 |
| RP11-288K12.1 | 0.028872571 | 1.627356178 |
| MFSD12 | 0.01889671 | 1.261988948 |
| NOL3 | 0.018925924 | 0.778882175 |
| SLC25A35 | 0.018971438 | 1.250132315 |
| CTD-2256P15.5 | 0.122560414 | 1.625777253 |
| OXT | 0.018991419 | 0.36188489 |
| EPC1 | 0.018993628 | 1.13682274 |
| RP11-191N8.2 | 0.103244717 | 1.625145356 |
| SNORA81 | 0.082217209 | 1.625119517 |
| LINC00581 | 0.149599141 | 1.625087901 |
| RP11-42O15.3 | 0.010208345 | 1.625036902 |
| EIF3IP1 | 0.046810967 | 1.624980179 |
| FARP1 | 0.018996567 | 1.385081818 |
| AC006195.2 | 0.060247831 | 1.624226378 |
| RNU4-23P | 0.039864316 | 1.624010841 |
| SLC4A7 | 0.019049257 | 1.28251893 |
| RP11-55L4.2 | 0.029957291 | 1.623500763 |
| TMEM2 | 0.019082701 | 1.3464553 |
| RP11-667M19.10 | 0.042065691 | 1.62330965 |
| YIPF3 | 0.019119518 | 1.128587071 |
| MIR4261 | 0.129402532 | 1.622575679 |
| RP11-309M23.1 | 0.134257084 | 1.622539647 |
| BLZF2P | 0.093376084 | 1.622377368 |
| GUF1 | 0.019138545 | 1.146274979 |
| NUCB2 | 0.019167346 | 1.330913702 |
| RP11-469H8.6 | 0.14160244 | 1.621195583 |
| Y_RNA | 0.12650202 | 1.621140102 |
| DDR1-AS1 | 0.02807212 | 1.620647796 |
| RP11-620J15.2 | 0.086256123 | 1.619948768 |
| RP11-548P2.2 | 0.131376419 | 1.619702742 |
| RP11-261P13.5 | 0.052580212 | 1.619269894 |
| AC104169.1 | 0.111487402 | 1.618936243 |
| HMGN5 | 0.019207493 | 1.600681344 |
| PENK | 0.019324473 | 5.588363876 |
| ZC3H8 | 0.019357801 | 0.856565021 |
| ANGPT2 | 0.019367224 | 1.484485615 |
| RP11-420L9.2 | 0.008556634 | 1.618450885 |
| ALKBH5 | 0.019392802 | 1.127362219 |
| PACERR | 0.081157413 | 1.617426416 |
| BTN2A1 | 0.019406057 | 1.186083274 |
| RP11-529H2.2 | 0.075914345 | 1.616433676 |
| TBC1D1 | 0.019430814 | 1.182990932 |
| RP11-306G20.1 | 0.001890182 | 1.61634956 |
| TNFSF11 | 0.01944236 | 2.895212966 |
| RP11-712L6.7 | 0.040732223 | 1.616124565 |
| CTC-461F20.1 | 0.093264496 | 1.615828208 |
| RP11-376O6.2 | 0.018037479 | 1.615803112 |
| HES2 | 0.019457552 | 0.54763669 |
| CDC37P1 | 0.050277137 | 1.615313517 |
| SLC25A30-AS1 | 0.039047897 | 1.615197104 |
| PSMC4 | 0.01948435 | 0.844139728 |
| GSTO3P | 0.144958656 | 1.614857939 |
| RP11-616M22.7 | 0.056780481 | 1.614622125 |
| CTD-2135D7.5 | 0.045975876 | 1.614401897 |
| LINC00184 | 0.0053257 | 1.614044269 |
| CTD-2008P7.9 | 0.023089551 | 1.613354745 |
| KLHL2 | 0.019514556 | 1.193026898 |
| DKFZP434A062 | 0.044409664 | 1.613151855 |
| RP11-399K21.10 | 0.113717716 | 1.613079679 |
| ZNF502 | 0.019521754 | 1.466953729 |
| RP11-121E16.1 | 0.140605848 | 1.612827462 |
| MYB-AS1 | 0.086540347 | 1.612702869 |
| TIAM2 | 0.01953419 | 1.274698555 |
| HMGN1P4 | 0.010370363 | 1.611695773 |
| SLC16A3 | 0.019565232 | 1.351167557 |
| OMP | 0.019619431 | 2.411591116 |
| RPL36AP48 | 0.049844301 | 1.609793578 |
| BNIP3P16 | 0.097758155 | 1.60853901 |
| HCG9P5 | 0.051077704 | 1.608404296 |
| ZNF204P | 0.000128199 | 1.607797926 |
| AC107079.1 | 0.129663319 | 1.607729638 |
| AC023283.1 | 0.001500306 | 1.607386442 |
| LINC01239 | 0.12627363 | 1.606850215 |
| FBXL2 | 0.019663921 | 1.441808518 |
| Metazoa_SRP | 0.016691847 | 1.606344324 |
| HEMK1 | 0.019702294 | 1.18067318 |
| RP11-231E4.2 | 0.148814786 | 1.606178074 |
| RNU6-130P | 0.117867083 | 1.606070264 |
| U73166.2 | 0.002692659 | 1.605690173 |
| SLC25A14 | 0.019764246 | 1.194515862 |
| CXCL11 | 0.019794176 | 2.168169537 |
| ACTR3B | 0.019841251 | 1.264696826 |
| LLPH-AS1 | 0.003638331 | 1.604662341 |
| CREM | 0.019852119 | 1.186148945 |
| TDGF1 | 0.019874405 | 9.502670903 |
| Y_RNA | 0.110478697 | 1.603550176 |
| ENO4 | 0.019878625 | 1.981165432 |
| RN7SL619P | 0.044525938 | 1.602697426 |
| RP11-328J14.1 | 0.045780592 | 1.601665638 |
| PCAT18 | 0.10200441 | 1.601037429 |
| PDXDC1 | 0.019945905 | 1.293892648 |
| RP11-818O24.2 | 0.019758326 | 1.600202148 |
| EPPIN | 0.019958332 | 4.367354805 |
| MXRA5Y | 0.047976561 | 1.599624024 |
| CTC-359D24.3 | 0.044634825 | 1.599420851 |
| RIMS3 | 0.019987122 | 0.55991389 |
| NFYB | 0.019992111 | 1.129576926 |
| RP3-467K16.4 | 0.018424027 | 1.598666184 |
| COX7BP2 | 0.147445909 | 1.598666045 |
| RP11-568K15.2 | 0.013177626 | 1.598641776 |
| BMS1P11 | 0.098635537 | 1.598590446 |
| RP4-625H18.2 | 0.078469271 | 1.598485163 |
| ACTG1P2 | 0.077310211 | 1.598399425 |
| AC016722.3 | 0.059638806 | 1.598201945 |
| RP11-326G21.1 | 0.022221044 | 1.597997549 |
| RP11-665C16.8 | 0.071642661 | 1.597745027 |
| CTD-3030D20.1 | 0.077094182 | 1.59770284 |
| RP11-17E2.2 | 0.037046681 | 1.59749832 |
| GPR115 | 0.020019931 | 0.68046071 |
| E2F3P2 | 0.048313141 | 1.596849781 |
| RP11-145A3.1 | 0.13088477 | 1.596725697 |
| ALG1L14P | 0.115562743 | 1.596652894 |
| RP11-551L14.6 | 0.090802218 | 1.596303062 |
| AC005077.8 | 0.138175115 | 1.596237111 |
| RP11-358M14.2 | 0.146301373 | 1.596197352 |
| ZNF451 | 0.020024136 | 1.129455365 |
| RP11-65I12.1 | 0.018523609 | 1.596076921 |
| AC009495.3 | 0.049125101 | 1.595748128 |
| CXorf23 | 0.020029369 | 1.212597959 |
| DDX11L10 | 0.043353556 | 1.595384824 |
| RPS20P24 | 0.090244742 | 1.595120866 |
| HCG20 | 0.014487056 | 1.594806613 |
| PDLIM7 | 0.020052575 | 1.28411564 |
| PRMT9 | 0.020076232 | 1.13416682 |
| PCBP2 | 0.020104917 | 1.110169039 |
| RP11-439E19.6 | 0.138801452 | 1.59406422 |
| TRADD | 0.020105993 | 0.849409437 |
| AC007750.5 | 0.074733109 | 1.594005422 |
| EEF1DP8 | 0.075800656 | 1.593994604 |
| FAM83D | 0.020108314 | 0.771462206 |
| TMEM91 | 0.020113881 | 1.406295807 |
| RP5-1147A1.2 | 0.039448212 | 1.592936557 |
| OSBPL10-AS1 | 0.008966743 | 1.592771844 |
| RP11-216N21.2 | 0.050323973 | 1.591656795 |
| RP11-92C4.3 | 0.030147941 | 1.591639665 |
| MYCBPAP | 0.020192579 | 1.479791055 |
| RPL12P44 | 0.046613777 | 1.591271344 |
| HESX1 | 0.020245937 | 1.314484286 |
| RANGAP1 | 0.0202468 | 0.862616167 |
| RP11-157L3.5 | 0.098103619 | 1.590506135 |
| RP11-364P2.2 | 0.0815809 | 1.590236612 |
| RNA5SP268 | 0.133731519 | 1.589855945 |
| RAB20 | 0.02025363 | 1.375411685 |
| NFU1 | 0.020273717 | 1.180337758 |
| RP11-6J21.2 | 0.128820816 | 1.588562371 |
| REST | 0.020285141 | 1.143396589 |
| CLRN1-AS1 | 0.135040292 | 1.588095029 |
| AC108004.2 | 0.062326188 | 1.587915053 |
| AC022182.1 | 0.072329433 | 1.587911383 |
| SNORA40 | 0.091133178 | 1.586668264 |
| RP11-44D5.2 | 0.040070239 | 1.586653481 |
| PROSER2 | 0.020297872 | 1.685366315 |
| CTC-367F4.1 | 0.099729537 | 1.585976406 |
| CCDC13-AS1 | 0.031416506 | 1.585638038 |
| LURAP1L | 0.020386839 | 0.581984896 |
| RP11-91H12.1 | 0.092036685 | 1.584822438 |
| TRPC5OS | 0.091385457 | 1.584595006 |
| AC007255.8 | 0.106101359 | 1.584426572 |
| RP11-70C1.3 | 0.065530434 | 1.58417198 |
| SNORA7 | 0.086286874 | 1.584153209 |
| RP11-376P6.3 | 0.056477168 | 1.583991322 |
| TMED4 | 0.020416256 | 1.110926806 |
| SMARCA5 | 0.020425961 | 1.136495136 |
| BMI1 | 0.020483749 | 1.197514765 |
| AQP10 | 0.020486732 | 3.142675364 |
| RP11-468H14.1 | 0.144284873 | 1.583170779 |
| RP11-10L12.1 | 0.079082425 | 1.583127589 |
| PKP2 | 0.020500593 | 1.433404658 |
| RGS7BP | 0.020529188 | 6.600710535 |
| CTD-2011F17.2 | 0.029057548 | 1.582054908 |
| RAPGEF2 | 0.020537178 | 1.222907604 |
| TPGS1 | 0.020575872 | 0.725745186 |
| AC005618.6 | 0.022370083 | 1.581419646 |
| RP11-100G15.3 | 0.034833747 | 1.581393086 |
| SLC41A3 | 0.020678575 | 1.176664771 |
| CLDN25 | 0.020684168 | 5.07867281 |
| SCGB1D2 | 0.020722822 | 6.739053148 |
| CHST5 | 0.020752964 | 1.97895821 |
| RP1-292B18.1 | 0.11648281 | 1.579824714 |
| Metazoa_SRP | 0.068927729 | 1.579740652 |
| RP11-433O3.1 | 0.053838776 | 1.579609826 |
| AKR1E2 | 0.020762294 | 0.740272466 |
| SLC7A1 | 0.020944453 | 1.241850397 |
| RP11-57H14.3 | 0.112259273 | 1.579126888 |
| RP11-779O18.2 | 0.037068751 | 1.578920632 |
| LINC00271 | 0.008940925 | 1.57891092 |
| COBLL1 | 0.02094728 | 0.723756632 |
| POU1F1 | 0.021045799 | 1.765375076 |
| RNU6ATAC16P | 0.067898793 | 1.578024947 |
| GOLGA2P5 | 0.007059295 | 1.577861358 |
| RP11-627G18.1 | 0.094203158 | 1.577586474 |
| RNA5SP195 | 0.080904886 | 1.577565401 |
| RP11-804H8.7 | 0.138134542 | 1.577561276 |
| CTD-2377D24.6 | 0.020216061 | 1.577545562 |
| MIR6772 | 0.047771898 | 1.577407996 |
| HMGN2P46 | 0.046793387 | 1.576946283 |
| NBPF19 | 0.021047135 | 0.791346385 |
| ING2 | 0.021055972 | 1.172342383 |
| NIPSNAP3B | 0.021068522 | 1.298366619 |
| FAM13B | 0.021078861 | 1.184330963 |
| WDR38 | 0.021083422 | 3.331478131 |
| CACNB1 | 0.021092039 | 1.458978704 |
| GSTT2 | 0.112652644 | 1.575072327 |
| RNA5SP71 | 0.089808459 | 1.574642465 |
| AP001625.6 | 0.015821128 | 1.574220392 |
| POLR3B | 0.021167257 | 0.845202825 |
| RP11-86H7.7 | 0.017305795 | 1.573962468 |
| DUSP16 | 0.021208015 | 1.213911402 |
| RP11-567J20.2 | 0.039525428 | 1.573351825 |
| TTC19 | 0.021250892 | 1.181246049 |
| Y_RNA | 0.101059131 | 1.57300889 |
| RP11-290D2.3 | 0.05931842 | 1.572321572 |
| RP11-359M6.1 | 0.057591526 | 1.572286374 |
| GALNT4 | 0.021312967 | 1.824520651 |
| HMGN1 | 0.021344095 | 1.138436008 |
| CASC2 | 0.004509668 | 1.571569774 |
| RP11-27G24.1 | 0.032044758 | 1.571531478 |
| UBE2B | 0.021354914 | 1.133089933 |
| RNU6-583P | 0.008189722 | 1.571037927 |
| CTD-2561J22.2 | 0.051060119 | 1.570708878 |
| RP4-595K12.1 | 0.034683743 | 1.570554187 |
| GLDC | 0.021358544 | 2.223045817 |
| RP11-423E7.1 | 0.075417502 | 1.570294493 |
| RPS3AP29 | 0.027025161 | 1.570269021 |
| RP11-227F19.5 | 0.091529226 | 1.569668718 |
| TCEB1P21 | 0.078344712 | 1.569187257 |
| ABRACL | 0.021399242 | 1.203880624 |
| KIRREL-IT1 | 0.12183915 | 1.569016108 |
| MKRNP2 | 0.113819777 | 1.568919038 |
| DEAR | 0.061289952 | 1.568477304 |
| LARS2 | 0.021429769 | 1.185151155 |
| RP11-575G13.2 | 0.095032527 | 1.567853096 |
| USP32P3 | 0.073494278 | 1.567512228 |
| LIMD1 | 0.021435422 | 1.183736319 |
| BNIP3P27 | 0.085828869 | 1.567146654 |
| RP11-551G24.2 | 0.078323486 | 1.566967764 |
| RP11-823E8.3 | 0.090717123 | 1.566964959 |
| CTD-3157E16.1 | 0.034480394 | 1.566018607 |
| AFAP1 | 0.021444825 | 1.303180117 |
| RP11-99A14.1 | 0.070125126 | 1.56580331 |
| RP11-834C11.14 | 0.103478003 | 1.565439059 |
| FAM156B | 0.021521067 | 1.661501067 |
| LRRC74B | 0.021533829 | 4.248422273 |
| TK2 | 0.021538056 | 1.198516289 |
| RDH14 | 0.021578972 | 1.145690982 |
| RP11-235E17.4 | 0.052561937 | 1.563378168 |
| ATP6V0A2 | 0.021579465 | 1.134849212 |
| TMCC2 | 0.021602889 | 0.589357845 |
| RNF126 | 0.02166657 | 0.828450536 |
| AC092168.2 | 0.027170878 | 1.562290168 |
| RP11-334A14.2 | 0.064913032 | 1.562054141 |
| RARSP1 | 0.081903882 | 1.561848792 |
| RP11-227G15.9 | 0.115241177 | 1.561555621 |
| RP5-943J3.2 | 0.031410809 | 1.561103046 |
| C20orf166-AS1 | 0.077606995 | 1.560760986 |
| LINC00989 | 0.118602472 | 1.560648256 |
| RP3-388N13.2 | 0.013666889 | 1.560338927 |
| RP11-317B7.2 | 0.129156892 | 1.560327414 |
| MIR601 | 0.115832151 | 1.560128865 |
| DNM1 | 0.021686665 | 0.686164604 |
| STX3 | 0.021719307 | 1.17696752 |
| LRRC6 | 0.021766595 | 1.548555191 |
| DUSP5P2 | 0.066161373 | 1.558856726 |
| RP11-471J7.1 | 0.101181797 | 1.558707062 |
| HOXB-AS4 | 0.008863136 | 1.558599911 |
| LUC7L3 | 0.021767725 | 1.147559476 |
| RP11-557F20.2 | 0.125009044 | 1.55816377 |
| CH507-154B10.1 | 0.125571245 | 1.558157289 |
| IL1RL2 | 0.02177546 | 0.750347098 |
| RANBP20P | 0.075562051 | 1.558008771 |
| RP11-67L14.1 | 0.028124137 | 1.557114565 |
| A2ML1 | 0.02178759 | 0.477008657 |
| KANSL1L | 0.021840223 | 1.314677494 |
| KIFC3 | 0.021846955 | 1.389198168 |
| MIR181A2HG | 0.120628586 | 1.555968196 |
| RP5-884M6.1 | 0.128067789 | 1.555928107 |
| RP11-178C3.6 | 0.09725885 | 1.555646894 |
| C4orf22 | 0.021849979 | 3.301171987 |
| PIGG | 0.021878751 | 1.144529612 |
| RP11-981P6.1 | 0.001755189 | 1.553984029 |
| RP11-522N14.2 | 0.053187141 | 1.55377981 |
| AP000233.4 | 0.131306298 | 1.553312637 |
| USP6 | 0.021880854 | 1.897848967 |
| ZNF793-AS1 | 0.066811957 | 1.553140123 |
| RP11-420H19.3 | 0.078476695 | 1.553008384 |
| RP11-178D12.2 | 0.118671756 | 1.552872208 |
| CASC4 | 0.021883042 | 1.195145458 |
| ALG1L7P | 0.004013338 | 1.551902652 |
| MYZAP | 0.021899363 | 1.426611903 |
| AC253576.2 | 0.011988036 | 1.55180219 |
| STEAP3 | 0.021931499 | 0.814436887 |
| RP11-354M20.3 | 0.065979652 | 1.551076696 |
| RP11-35O15.1 | 0.096593303 | 1.55092544 |
| RP11-48G14.1 | 0.054013284 | 1.55043749 |
| CYCSP10 | 0.011732296 | 1.550317657 |
| LRRC59 | 0.02196366 | 1.154823528 |
| C1orf87 | 0.021984597 | 3.895905917 |
| AC009502.4 | 0.051046298 | 1.549757337 |
| KB-1615E4.3 | 0.134634844 | 1.549576966 |
| MIR6838 | 0.059352644 | 1.549489775 |
| FLJ44511 | 0.014087523 | 1.549435642 |
| RP11-119D9.1 | 0.133213867 | 1.549162503 |
| SRP19 | 0.021984982 | 1.148656797 |
| DPY19L2P4 | 0.091177822 | 1.548504449 |
| IL12A-AS1 | 0.086869387 | 1.548154283 |
| GPR64 | 0.02202705 | 3.367498636 |
| CTB-40H15.4 | 0.046387188 | 1.547443347 |
| GCKR | 0.022034777 | 2.732921651 |
| ZNF816 | 0.022072608 | 1.346667283 |
| HTATSF1 | 0.022081174 | 1.176400416 |
| FAM169A | 0.022099233 | 1.466333472 |
| RAET1G | 0.022135346 | 1.58011397 |
| ADAMTSL4 | 0.022189728 | 1.798230224 |
| RPL21P65 | 0.030045049 | 1.545334322 |
| RP11-231I16.1 | 0.016263962 | 1.545180048 |
| EPHA1 | 0.022259394 | 0.726855156 |
| ABCF2P1 | 0.05074751 | 1.54394243 |
| Y_RNA | 0.021032629 | 1.543390262 |
| TCAF2 | 0.022263958 | 0.761807757 |
| CTC-457E21.1 | 0.11880842 | 1.542776523 |
| RP11-626G3.1 | 0.029347259 | 1.542732367 |
| LA16c-312E8.4 | 0.126711132 | 1.542476109 |
| CH17-189H20.1 | 0.002068974 | 1.54219209 |
| AC006480.1 | 0.055634187 | 1.542144314 |
| LINC00327 | 0.122572389 | 1.541849858 |
| RP11-388B24.4 | 0.088451517 | 1.541699216 |
| DLL1 | 0.022330865 | 1.655600041 |
| RP11-69L16.6 | 0.074572395 | 1.541670011 |
| ODF2L | 0.022341512 | 1.208112711 |
| RP11-631M21.7 | 0.068823691 | 1.54154785 |
| RP11-266K4.14 | 0.119937735 | 1.541112685 |
| RP11-440K22.1 | 0.072629003 | 1.540771365 |
| RP11-16K12.1 | 0.09086641 | 1.540589349 |
| EML3 | 0.022380402 | 0.90051166 |
| RP11-803D5.4 | 0.031399225 | 1.540365105 |
| CTD-2047H16.4 | 0.008635698 | 1.540065552 |
| MEIS1-AS2 | 0.004403697 | 1.539522177 |
| LINC00954 | 0.076157352 | 1.538579876 |
| RP1-117B12.4 | 0.023703786 | 1.538040173 |
| CTD-2325P2.4 | 0.052715146 | 1.537722751 |
| RNF41 | 0.022421568 | 1.099876704 |
| RP11-465K1.2 | 0.019405413 | 1.537612317 |
| CTD-2377O17.1 | 0.0554494 | 1.537383211 |
| RP11-677N16.1 | 0.017092554 | 1.536746626 |
| TMEM259 | 0.022422415 | 0.873479848 |
| SPINK4 | 0.022428012 | 3.07871098 |
| ZNF22 | 0.022450907 | 1.188863943 |
| HOXD11 | 0.022462017 | 0.693367861 |
| CLUAP1 | 0.022468002 | 1.232990202 |
| RP11-426D19.1 | 0.019059049 | 1.535217404 |
| NPM1P34 | 0.032270197 | 1.534672172 |
| LINC01348 | 0.027814294 | 1.534437053 |
| ERICH6-AS1 | 0.039118284 | 1.533978618 |
| RNU2-7P | 0.048226554 | 1.533971881 |
| RP11-299M14.2 | 0.048740635 | 1.533816171 |
| PALM3 | 0.022469392 | 2.587363789 |
| UBBP4 | 0.022496751 | 1.182756068 |
| RP11-946P6.4 | 0.130304474 | 1.533055125 |
| TMC5 | 0.022536307 | 1.980621822 |
| ENPP7P4 | 0.019404668 | 1.532702619 |
| ZCCHC9 | 0.022559655 | 1.106720074 |
| RP11-665C16.5 | 0.074084114 | 1.531973898 |
| RP11-680G10.1 | 0.068434907 | 1.531876932 |
| RP1-261D10.1 | 0.059774908 | 1.531205002 |
| SCARA5 | 0.022580438 | 2.57801304 |
| AC104076.3 | 0.077403114 | 1.531180559 |
| SLC35D2 | 0.022581396 | 1.160186061 |
| LRPAP1 | 0.022618046 | 1.180349598 |
| PAX8 | 0.022685343 | 2.145419958 |
| PDE3A | 0.022724849 | 3.292208097 |
| AC023115.4 | 0.122585368 | 1.52978309 |
| KDELC1P1 | 0.086045294 | 1.529698438 |
| RP11-383C5.3 | 0.079024846 | 1.52951966 |
| SLC3A1 | 0.022814817 | 11.81602678 |
| PDE5A | 0.022824146 | 1.435352669 |
| AC004846.1 | 0.084484712 | 1.529255877 |
| CCDC155 | 0.022824252 | 1.917898535 |
| LL22NC03-2H8.5 | 0.002530295 | 1.528720972 |
| RP11-492E3.51 | 0.007437527 | 1.528485579 |
| PCYT1A | 0.022825692 | 0.780032574 |
| DNAI1 | 0.022840626 | 3.328712535 |
| LRRC27 | 0.022870052 | 1.238321708 |
| RGL3 | 0.02287705 | 1.971620718 |
| ATP6V1H | 0.022887195 | 1.125502146 |
| AF064858.10 | 0.071689059 | 1.526839175 |
| RP11-573M3.3 | 0.002680247 | 1.525929657 |
| RP13-1016M1.2 | 0.03505184 | 1.525733027 |
| RP11-632K5.2 | 0.103890735 | 1.525705597 |
| RP11-130F10.1 | 0.110634469 | 1.525383706 |
| RP3-337H4.10 | 0.004652961 | 1.525031731 |
| SNORA47 | 0.110518957 | 1.524574534 |
| HOXB-AS3 | 0.117040494 | 1.524526723 |
| HMGB1P21 | 0.051889807 | 1.524428614 |
| COX7A2P1 | 0.006884613 | 1.523265992 |
| ZNF69 | 0.022899339 | 1.738031719 |
| RP11-274H2.3 | 0.090325585 | 1.522657254 |
| NAT1 | 0.022920319 | 1.564722438 |
| XXbac-BPG252P9.10 | 0.019611114 | 1.522064141 |
| ZNF503-AS1 | 0.027555607 | 1.521909305 |
| RP11-834C11.11 | 0.144444737 | 1.521762305 |
| RP11-295M3.4 | 0.140302153 | 1.521618883 |
| MSRB2 | 0.023004315 | 1.3265653 |
| KLHL35 | 0.023016598 | 0.749245001 |
| RP11-379K22.2 | 0.142526567 | 1.520894453 |
| HTT | 0.023049478 | 1.144445724 |
| TECR | 0.023051943 | 0.872420266 |
| RP3-388E23.2 | 0.009432729 | 1.520074636 |
| LRRC4 | 0.023072433 | 0.535817916 |
| AC044907.1 | 0.080870763 | 1.519672396 |
| MCAM | 0.023108617 | 1.508024733 |
| RP11-861L17.4 | 0.082702342 | 1.519275599 |
| RP11-319G9.4 | 0.147689793 | 1.519140072 |
| RNA5SP21 | 0.144790667 | 1.519003444 |
| CTNNA1 | 0.023127934 | 1.132846739 |
| MAEA | 0.023134867 | 1.126243738 |
| CCDC129 | 0.023153843 | 4.619292738 |
| ATXN7 | 0.023153875 | 1.168946685 |
| RNU4-78P | 0.109958044 | 1.517396191 |
| SLC25A25-AS1 | 0.018225006 | 1.517388873 |
| NUDT9 | 0.02329378 | 1.152206599 |
| ENPP7P2 | 0.08126193 | 1.516143626 |
| RP11-50D16.4 | 0.004183731 | 1.515963261 |
| RP1-292B18.4 | 0.047725162 | 1.51591241 |
| CCDC13 | 0.023337854 | 1.594290482 |
| DCTN5 | 0.023417835 | 0.886207038 |
| ZNF248 | 0.023424077 | 1.164146435 |
| C1QTNF9B-AS1 | 0.041776915 | 1.515379763 |
| SLC27A4 | 0.023501539 | 0.825252878 |
| BTBD3 | 0.023534514 | 1.411561737 |
| RP11-757A13.1 | 0.091011346 | 1.514917154 |
| RP1-209B5.2 | 0.117013713 | 1.514848242 |
| CTB-30L5.1 | 0.103238377 | 1.514697232 |
| AC097461.4 | 0.007253274 | 1.514377488 |
| MITD1 | 0.023545958 | 1.107776061 |
| ERBB3 | 0.023599682 | 1.234188767 |
| RN7SKP239 | 0.02079083 | 1.513847386 |
| RP11-25G10.2 | 0.143912528 | 1.513651905 |
| KCNMB2 | 0.023609208 | 5.945768551 |
| AC015849.14 | 0.068229934 | 1.512975891 |
| RP11-538D16.3 | 0.074255091 | 1.512697512 |
| RP11-423E7.2 | 0.016558327 | 1.512362678 |
| CMTM7 | 0.023620104 | 1.559840934 |
| CTC-455F18.3 | 0.053463107 | 1.511713442 |
| KIZ-AS1 | 0.108381662 | 1.511392253 |
| AC011284.3 | 0.132272788 | 1.51103139 |
| EIF3K | 0.023621243 | 0.781143736 |
| HIF1A | 0.023635032 | 1.262071952 |
| FZD3 | 0.023679829 | 1.758294517 |
| RP11-26F2.1 | 0.131622964 | 1.510287197 |
| RP11-5G9.5 | 0.046935272 | 1.510017509 |
| FER1L4 | 0.091948695 | 1.509824684 |
| RMDN2-AS1 | 0.058564079 | 1.508884566 |
| RP11-181E10.3 | 0.085764504 | 1.508724097 |
| ASCL3 | 0.023693598 | 4.234688038 |
| AL513327.1 | 0.023100271 | 1.508686607 |
| RPS12P2 | 0.079186517 | 1.508657099 |
| MRPL15P1 | 0.095883631 | 1.508372916 |
| LRRC37A11P | 0.123135312 | 1.50815802 |
| RHOQ | 0.023749613 | 1.228969597 |
| RP11-474I11.8 | 0.118018935 | 1.507896451 |
| DYNLT1 | 0.023756506 | 1.160816811 |
| MBD2 | 0.023811371 | 0.859935704 |
| AC114776.1 | 0.00565063 | 1.5074356 |
| RP11-401N16.2 | 0.11385075 | 1.507288296 |
| IQCD | 0.023852564 | 1.662604154 |
| TMEM220-AS1 | 0.045978761 | 1.507013832 |
| RP11-126K1.9 | 0.080179331 | 1.506739597 |
| TMED10P2 | 0.050743103 | 1.506633484 |
| RNU6-30P | 0.006104076 | 1.506558149 |
| ZNF76 | 0.023855828 | 1.150440948 |
| ZNF415P1 | 0.096228206 | 1.506378935 |
| WASIR2 | 0.150298043 | 1.506042706 |
| TRIM31 | 0.023886409 | 2.578780377 |
| SOX5 | 0.023897764 | 1.861332248 |
| RP11-686D22.5 | 0.032145722 | 1.505339948 |
| FLJ38576 | 0.02150051 | 1.505215413 |
| GAK | 0.023905701 | 1.152574558 |
| RP11-230F18.6 | 0.010885457 | 1.504664922 |
| RP11-366L20.2 | 0.069069557 | 1.504634163 |
| MAPK8IP2 | 0.024001249 | 0.614377227 |
| RP4-536B24.4 | 0.065867759 | 1.503814206 |
| C5orf24 | 0.024013927 | 1.155435211 |
| snoU13 | 0.150998028 | 1.503362366 |
| GLI4 | 0.024036847 | 0.816650337 |
| CYP4V2 | 0.024057582 | 1.250180951 |
| RP11-48G14.2 | 0.14172322 | 1.502514502 |
| UQCRHP1 | 0.139359365 | 1.502386072 |
| AC008391.1 | 0.067980455 | 1.50218262 |
| RN7SL477P | 0.146359936 | 1.501769588 |
| CBX1P4 | 0.150259153 | 1.501497454 |
| RP13-514E23.2 | 0.098367023 | 1.501463869 |
| RP11-73M7.1 | 0.01615796 | 1.501424704 |
| ZFP64 | 0.024063855 | 0.825028729 |
| ABCC5 | 0.024117165 | 0.618167867 |
| RIC1 | 0.024136512 | 0.594782298 |
| CFL1P6 | 0.029267805 | 1.500623196 |
| SNRPGP5 | 0.058665703 | 1.500445819 |
| GLRB | 0.02416314 | 1.762885203 |
| RP4-710M3.1 | 0.148127795 | 1.500232503 |
| AC064836.3 | 0.020539288 | 1.499900538 |
| RP11-611L7.2 | 0.047850592 | 1.499834451 |
| DYNLRB2 | 0.024184996 | 1.802460314 |
| LINC01423 | 0.121456389 | 1.499723977 |
| NUDT15 | 0.024196609 | 1.144479631 |
| RPS2P35 | 0.059272873 | 1.49941939 |
| OR9A2 | 0.02419841 | 2.785726279 |
| SMARCA1 | 0.024204128 | 1.393380208 |
| RP11-154H23.3 | 0.010432795 | 1.499245765 |
| RNU6-204P | 0.088565569 | 1.499079208 |
| SIPA1L2 | 0.02424288 | 0.696577874 |
| CTAGE10P | 0.120328204 | 1.498519051 |
| CCDC94 | 0.02424957 | 0.853682773 |
| COG3 | 0.024251423 | 1.164847483 |
| LRP5 | 0.024272757 | 1.296997961 |
| DLGAP1-AS1 | 0.000697051 | 1.498387101 |
| PSMC2 | 0.024281198 | 0.887887387 |
| BCL2L14 | 0.024316466 | 1.453862401 |
| SURF4 | 0.024337389 | 1.114913889 |
| RP11-354P11.8 | 0.101812485 | 1.497555515 |
| AC058791.1 | 0.025226151 | 1.497306591 |
| UGDH-AS1 | 0.000963252 | 1.497112965 |
| SPIN1 | 0.02438253 | 1.137723776 |
| MIR6804 | 0.103588761 | 1.49703683 |
| RP11-535A5.1 | 0.068265649 | 1.496611044 |
| CTC-534B23.1 | 0.064299091 | 1.49573708 |
| RP11-227G15.11 | 0.010441443 | 1.495334152 |
| RP11-371F15.3 | 0.040213734 | 1.495212689 |
| EIF4HP2 | 0.000790595 | 1.49519536 |
| TMEM109 | 0.024388004 | 0.864427111 |
| KIAA1598 | 0.02438904 | 1.199454804 |
| RASSF8-AS1 | 0.042340749 | 1.494572014 |
| UROC1 | 0.024404576 | 2.745266581 |
| RP11-755B10.3 | 0.05168725 | 1.493833886 |
| GLP2R | 0.024416478 | 5.112837508 |
| LNX1-AS2 | 0.033425301 | 1.493324935 |
| LRRC47 | 0.024427576 | 0.897685791 |
| LDHAP7 | 0.030724723 | 1.492843785 |
| RP11-775A3.1 | 0.088105901 | 1.492745121 |
| RP11-706O15.3 | 0.037971268 | 1.492696859 |
| RP3-414A15.10 | 0.037582554 | 1.492444617 |
| RHOBTB2 | 0.024519101 | 1.436701272 |
| CH507-154B10.2 | 0.014125302 | 1.492202282 |
| RP11-214K3.5 | 0.146465538 | 1.492138711 |
| RP11-26P13.2 | 0.091188706 | 1.492085374 |
| FOXF1 | 0.024578178 | 1.648166531 |
| CTB-46B19.1 | 0.087867911 | 1.491886887 |
| LILRP2 | 0.037024233 | 1.49172692 |
| RP11-166B2.5 | 0.026468182 | 1.491210771 |
| RP1-136B1.1 | 0.01881882 | 1.491075393 |
| AF064858.8 | 0.030582773 | 1.490950234 |
| RP11-316K19.3 | 0.009261832 | 1.490842816 |
| SNORA75 | 0.145439345 | 1.490458471 |
| RN7SKP97 | 0.005633743 | 1.490011921 |
| RP11-160H22.3 | 0.10173246 | 1.489621764 |
| GID8 | 0.024597243 | 0.875499743 |
| RP11-290L1.3 | 0.133117946 | 1.489335139 |
| TREM1 | 0.024630791 | 2.004377732 |
| RP11-403P17.4 | 0.036553191 | 1.48877742 |
| ANGPT1 | 0.024647356 | 1.616240894 |
| XX-C2158C6.3 | 0.129246662 | 1.488634874 |
| LL0XNC01-240C2.1 | 0.127768417 | 1.488082235 |
| RP11-370A5.2 | 0.105133834 | 1.48781212 |
| ULK4P2 | 0.145053003 | 1.487774305 |
| AP001434.2 | 0.082312141 | 1.487484711 |
| GFI1B | 0.024665357 | 7.03399182 |
| RCL1 | 0.024670599 | 0.574946898 |
| RP11-762L8.6 | 0.022351062 | 1.487151282 |
| ZNF48 | 0.02467181 | 1.21286336 |
| MEIS3P1 | 0.084635268 | 1.486724924 |
| FOXN1 | 0.02476325 | 0.620892308 |
| RP11-380M21.1 | 0.082328362 | 1.486348909 |
| RP1-122O8.7 | 0.002309101 | 1.485694918 |
| CA8 | 0.024800068 | 3.531732791 |
| RP11-739N20.2 | 0.12741582 | 1.485618744 |
| CYP2U1 | 0.02482968 | 1.269331909 |
| RP11-876N24.5 | 0.010992026 | 1.485422874 |
| RNU6-519P | 0.083543282 | 1.485246081 |
| LINC00894 | 0.015631096 | 1.485211709 |
| AC079354.6 | 0.149992285 | 1.48460839 |
| ARL6IP5 | 0.024852036 | 1.167154071 |
| ARMC2 | 0.024866014 | 1.35851488 |
| ZNF219 | 0.024965302 | 0.832999969 |
| IRAK2 | 0.024982049 | 1.34551894 |
| TMPPE | 0.024985965 | 1.247181362 |
| AC004543.2 | 0.144851745 | 1.483429885 |
| RP11-504I13.3 | 0.033771461 | 1.483415125 |
| SPAG1 | 0.024990051 | 1.304812997 |
| CTSLP2 | 0.098579666 | 1.482561999 |
| NLRP14 | 0.025032452 | 1.605621575 |
| UFL1-AS1 | 0.137020768 | 1.481535145 |
| CFL1P3 | 0.054786493 | 1.481065497 |
| TXNDC16 | 0.025038055 | 1.233820453 |
| AC097635.5 | 0.037903885 | 1.480449048 |
| RP11-9M16.3 | 0.12458407 | 1.480214546 |
| FAM149B1 | 0.025040786 | 1.131548172 |
| SLC28A2 | 0.025041841 | 4.306582396 |
| UBP1 | 0.025044727 | 1.155277073 |
| RP11-802E16.3 | 0.009987945 | 1.479087691 |
| CTD-2575K13.6 | 0.075328647 | 1.478964 |
| HAPLN1 | 0.02505785 | 1.836648323 |
| UBXN2B | 0.025061976 | 1.147256675 |
| METTL10 | 0.025101021 | 1.146650057 |
| RP11-1094H24.4 | 0.005333776 | 1.477685049 |
| RP11-13A1.1 | 0.143655254 | 1.477620004 |
| HRASLS | 0.025108441 | 0.600327956 |
| AC073636.1 | 0.108929886 | 1.476954885 |
| BMPER | 0.025112652 | 3.158813143 |
| OR2D2 | 0.025119976 | 3.777813673 |
| OR10AB1P | 0.111305753 | 1.476421198 |
| RPL23AP10 | 0.07655598 | 1.475477075 |
| CTC-308K20.1 | 0.021707862 | 1.475333068 |
| CTB-134F13.1 | 0.053620558 | 1.474794443 |
| HMGB3P32 | 0.111552986 | 1.474700548 |
| SNRPEP5 | 0.026768846 | 1.474696493 |
| FAM85B | 0.043632336 | 1.474678455 |
| RP11-976B16.1 | 0.071346911 | 1.474091464 |
| RP11-641A6.9 | 0.101949451 | 1.473965742 |
| SNORD117 | 0.071820826 | 1.473752744 |
| ZMYM4-AS1 | 0.027647188 | 1.473001631 |
| BZW1 | 0.025211669 | 1.17512004 |
| CTC-498M16.2 | 0.118295019 | 1.472717048 |
| VPS13C | 0.02521727 | 1.231357509 |
| RP3-415N12.1 | 0.080367753 | 1.472261065 |
| RP11-35O15.2 | 0.087406758 | 1.472027353 |
| FOXP1-IT1 | 0.037459209 | 1.471966141 |
| RP11-492E3.2 | 0.135625018 | 1.471479148 |
| KRTAP5-1 | 0.025271511 | 1.829988232 |
| HOXD10 | 0.025275231 | 0.695502517 |
| MARVELD3 | 0.025321187 | 1.190175977 |
| AC105009.1 | 0.025359049 | 1.546487909 |
| KB-1742H10.3 | 0.07205949 | 1.470257891 |
| PDPN | 0.025361098 | 1.580905941 |
| ZNF630-AS1 | 0.052605994 | 1.469470492 |
| RNU6-1209P | 0.047514485 | 1.469358591 |
| LMNB1 | 0.025367795 | 1.160498826 |
| RP11-141C7.3 | 0.079004115 | 1.469023248 |
| AC007796.1 | 0.145580908 | 1.468979433 |
| TMEM72-AS1 | 0.06188988 | 1.468809761 |
| RN7SL154P | 0.14228113 | 1.468668012 |
| CTGLF8P | 0.085850965 | 1.468616599 |
| RP11-517H2.6 | 0.016093313 | 1.468560362 |
| RP5-1042K10.12 | 0.051997896 | 1.468200996 |
| RP11-648O15.1 | 0.137827155 | 1.468095825 |
| KRT8P18 | 0.056040429 | 1.468043162 |
| RP11-4C20.4 | 0.126477161 | 1.467542603 |
| OR5A1 | 0.025393896 | 7.216107015 |
| OR2K2 | 0.025394454 | 0.357757201 |
| SNRPCP3 | 0.041110132 | 1.466903363 |
| CTD-2006C1.2 | 0.025116728 | 1.466812196 |
| CEMIP | 0.025410842 | 2.097968948 |
| MORF4L2-AS1 | 0.016638738 | 1.466447824 |
| ZDHHC4 | 0.025415474 | 1.17013517 |
| SLC17A7 | 0.025437672 | 3.96626692 |
| TRIM55 | 0.025458034 | 2.266703005 |
| HNRNPA0 | 0.025472413 | 1.107955245 |
| RASSF8 | 0.025485674 | 1.381348882 |
| RP3-388N13.3 | 0.060761903 | 1.465864442 |
| RP11-112J1.3 | 0.080418932 | 1.46551226 |
| TEX36 | 0.025520392 | 6.242439113 |
| RNU6-548P | 0.05415734 | 1.464652203 |
| HIF1A-AS1 | 0.116007288 | 1.464533669 |
| EFCAB13 | 0.025561562 | 1.261638367 |
| RP11-2H3.7 | 0.056063066 | 1.463828407 |
| RPS23P2 | 0.126870208 | 1.463634077 |
| SPRY4 | 0.025590722 | 1.593764453 |
| RP11-486G15.1 | 0.147520458 | 1.462925115 |
| MYL6P3 | 0.034613433 | 1.462924008 |
| VPS13A | 0.02561417 | 1.185041308 |
| TMEM189 | 0.025624922 | 0.853259273 |
| RP11-243J18.2 | 0.132522352 | 1.462300403 |
| RP11-250B2.4 | 0.037825429 | 1.462218347 |
| ATP1A3 | 0.025645543 | 0.389456372 |
| MTHFD1P1 | 0.018519981 | 1.46214283 |
| PDILT | 0.025666665 | 11.19684045 |
| VPS4B | 0.025697724 | 0.832066373 |
| ALKBH3 | 0.025700235 | 0.810395776 |
| LINC00345 | 0.082056633 | 1.461431686 |
| LINC00299 | 0.038551907 | 1.461414157 |
| HNRNPA1P32 | 0.108258103 | 1.461306129 |
| RP11-662I13.2 | 0.117820519 | 1.461091976 |
| C20orf27 | 0.025761196 | 0.742074013 |
| RP11-255E6.6 | 0.051104293 | 1.460797274 |
| PGM5P2 | 0.004995939 | 1.460555723 |
| C2orf70 | 0.025789638 | 2.257927921 |
| CSRNP1 | 0.025797097 | 1.308018112 |
| RP11-16B13.1 | 0.14364331 | 1.459718692 |
| TMEM55A | 0.02582908 | 1.358461311 |
| ODF3L1 | 0.025874766 | 1.477076871 |
| PIGB | 0.025892625 | 1.146148787 |
| AMIGO2 | 0.025913156 | 1.750490373 |
| FRMD8 | 0.025928188 | 0.826610945 |
| RP11-128N14.5 | 0.034468461 | 1.458772439 |
| RPSAP63 | 0.051773326 | 1.458557311 |
| AQP5 | 0.026057149 | 2.560417846 |
| RP5-878I13.2 | 0.117063662 | 1.457968678 |
| TGDS | 0.026060594 | 1.157777666 |
| LMO7 | 0.026060988 | 1.369857537 |
| ADGB | 0.026088496 | 6.142693398 |
| MFSD6L | 0.026166446 | 1.980430044 |
| RP11-432J24.2 | 0.135579543 | 1.45686782 |
| SNX9 | 0.026202118 | 1.191736455 |
| AL157871.2 | 0.15110132 | 1.456574003 |
| RP11-15B17.1 | 0.045224249 | 1.456464252 |
| AC097724.3 | 0.019283542 | 1.456369343 |
| RP11-259P15.4 | 0.087448658 | 1.456146109 |
| SHC1P2 | 0.03958453 | 1.455995147 |
| MIA | 0.026224823 | 5.100909187 |
| RIMKLBP1 | 0.031389034 | 1.455368395 |
| HCP5B | 0.103489365 | 1.455318927 |
| RP11-290F5.2 | 0.020962439 | 1.455272203 |
| RIT1 | 0.026274452 | 0.794446759 |
| CTD-2515H24.2 | 0.138499042 | 1.454869437 |
| RP11-177G23.1 | 0.045548399 | 1.454375731 |
| SQLE | 0.02632227 | 0.718182263 |
| LRRC10B | 0.026333519 | 2.285295384 |
| MYH10 | 0.026390025 | 1.412715905 |
| FAM13A | 0.026469614 | 1.331259241 |
| LXN | 0.026505765 | 1.570393543 |
| RP1-142L7.9 | 0.115385938 | 1.452318669 |
| RP11-541M12.6 | 0.091456163 | 1.452057397 |
| RP11-583F2.7 | 0.056287031 | 1.452025637 |
| TIMM44 | 0.026523938 | 0.844008087 |
| CYCSP34 | 0.00868682 | 1.451408805 |
| AC139143.2 | 0.006586752 | 1.451315431 |
| GAD2 | 0.026525193 | 1.801571589 |
| RP11-230F18.5 | 0.000499129 | 1.451199324 |
| RP11-380L11.4 | 0.079586947 | 1.450706642 |
| Y_RNA | 0.135430762 | 1.450697354 |
| RP11-235E17.6 | 0.001056682 | 1.450446787 |
| RP3-405J10.3 | 0.018756525 | 1.450367524 |
| NEK10 | 0.02654078 | 1.824524338 |
| RP11-583F2.1 | 0.007402184 | 1.449736696 |
| DDX39BP2 | 0.13482132 | 1.44918193 |
| RP3-408N23.4 | 0.129157121 | 1.44878323 |
| RP11-269M20.3 | 0.036130245 | 1.448295239 |
| NR2C1 | 0.026565246 | 1.160472041 |
| RP11-283G6.6 | 0.126207237 | 1.448277449 |
| RP11-165D6.1 | 0.035446644 | 1.447985881 |
| AC068657.2 | 0.093106753 | 1.447913404 |
| TEX14 | 0.026570965 | 1.530657163 |
| HNRNPA1P46 | 0.130733103 | 1.44746685 |
| DNAAF3 | 0.026574745 | 1.622847032 |
| MICU1 | 0.026582343 | 1.149891996 |
| RP11-107F6.4 | 0.107619685 | 1.446897888 |
| RP11-260E18.1 | 0.08263687 | 1.446790077 |
| SUCLG2-AS1 | 0.004398235 | 1.446014323 |
| ZNF890P | 0.058775775 | 1.446011673 |
| AJAP1 | 0.026588929 | 2.693656287 |
| RPS23P5 | 0.090422238 | 1.445486924 |
| UQCC1 | 0.026591131 | 0.862795566 |
| SPPL2A | 0.026612741 | 1.134998648 |
| CCDC14 | 0.026616843 | 1.240454153 |
| RN7SL153P | 0.098480884 | 1.444666733 |
| RP11-266K22.2 | 0.060905695 | 1.44425033 |
| XXbac-BPG181B23.7 | 0.003455746 | 1.444182807 |
| GALNT7 | 0.026622611 | 1.401612127 |
| C10orf95 | 0.003853309 | 1.443478593 |
| CYP4F3 | 0.026650331 | 0.50446415 |
| ZGRF1 | 0.026660402 | 1.182911581 |
| RP11-517I3.1 | 0.051155533 | 1.443102422 |
| RP11-175P13.2 | 0.090893953 | 1.442863907 |
| TMEM14C | 0.026723532 | 1.196881202 |
| C1orf158 | 0.026740569 | 6.49641307 |
| GNAQ | 0.026801778 | 1.172632334 |
| COX6CP2 | 0.122691899 | 1.442561413 |
| CTH | 0.026819756 | 1.501101819 |
| ALG13-AS1 | 0.059884149 | 1.442176066 |
| RNU1-100P | 0.126014226 | 1.442028421 |
| MAP4 | 0.026835655 | 1.143348403 |
| TRIM60P18 | 0.030094482 | 1.44193781 |
| TMEM234 | 0.026856311 | 1.147423258 |
| LA16c-380H5.5 | 0.122629469 | 1.441827061 |
| PHF11 | 0.026871658 | 1.16786128 |
| TGM2 | 0.026894057 | 1.637769989 |
| CHCHD2P11 | 0.121425907 | 1.441633345 |
| CDK7 | 0.026953885 | 1.118793223 |
| TMEM56 | 0.026957435 | 1.375923002 |
| CTD-2647E9.3 | 0.13351324 | 1.441102126 |
| EIF1AX-AS1 | 0.101814532 | 1.440691778 |
| LRRIQ1 | 0.027013118 | 2.178981815 |
| ARF3 | 0.027013842 | 1.098682489 |
| NXNL2 | 0.027081009 | 2.046736938 |
| RP11-717F1.2 | 0.062883314 | 1.439795182 |
| ZNF564 | 0.027105275 | 1.195832464 |
| CTC-806A22.1 | 0.105591198 | 1.438995183 |
| ZNF195 | 0.027155761 | 1.188376326 |
| RP11-78J21.6 | 0.074888984 | 1.438674131 |
| RP11-371A19.2 | 0.068883896 | 1.438333069 |
| RP11-736K20.5 | 0.103347199 | 1.438119348 |
| SEC13P1 | 0.087766657 | 1.437997432 |
| ZNF252P-AS1 | 0.057655845 | 1.437397934 |
| DYRK4 | 0.027184132 | 1.210977028 |
| RP11-1148O4.1 | 0.068493093 | 1.437103995 |
| RP11-84A19.4 | 0.051668514 | 1.437033723 |
| RP11-126K1.6 | 0.023540099 | 1.4370083 |
| RP1-102D24.5 | 0.049125213 | 1.436806718 |
| SPATA7 | 0.027209983 | 1.248817046 |
| NPPA | 0.027224808 | 1.345935639 |
| YPEL5P2 | 0.057587497 | 1.436676191 |
| RP13-131K19.1 | 0.01254169 | 1.436556754 |
| TFPI | 0.027253404 | 1.754900871 |
| EMP1 | 0.027277468 | 0.687669272 |
| OR2L5 | 0.027288226 | 6.166941166 |
| INSC | 0.027303915 | 3.001864339 |
| RP1-69D17.3 | 0.051592284 | 1.436235552 |
| RP11-737O24.5 | 0.023723603 | 1.436225113 |
| KIF9-AS1 | 0.002814662 | 1.435531779 |
| SUGT1P | 0.014047279 | 1.435474317 |
| FOXP1 | 0.027311783 | 1.221443682 |
| RP11-677M14.5 | 0.011599813 | 1.435099499 |
| COX20P1 | 0.147088928 | 1.434897469 |
| AC090181.1 | 0.121762193 | 1.434697232 |
| ALG5 | 0.027332908 | 1.155424129 |
| Metazoa_SRP | 0.038923628 | 1.434479726 |
| RP11-353N4.6 | 0.034803368 | 1.434297127 |
| RP11-359I18.5 | 0.090495577 | 1.434245927 |
| RPL5P30 | 0.004030061 | 1.433893067 |
| RP11-290C10.1 | 0.141727802 | 1.43368912 |
| RP11-568K15.1 | 0.01155383 | 1.433590184 |
| CLGN | 0.027451922 | 2.01410696 |
| ROM1 | 0.027467918 | 0.805423125 |
| DHCR24 | 0.027550306 | 0.798983775 |
| UBBP2 | 0.022669898 | 1.433120708 |
| VHL | 0.02755473 | 1.203328225 |
| LA16c-329F2.2 | 0.009731587 | 1.432543768 |
| RP4-639F20.1 | 0.058603407 | 1.432528473 |
| ANXA9 | 0.027653243 | 1.45746613 |
| RPL15P20 | 0.020179731 | 1.432084013 |
| KCNH8 | 0.027674762 | 2.644818799 |
| CRYL1 | 0.027677142 | 1.389013291 |
| C15orf54 | 0.138954672 | 1.430976877 |
| SLC9A3R1 | 0.027694802 | 0.76254642 |
| LRP5L | 0.027697766 | 1.275959073 |
| TADA2B | 0.027768876 | 1.130983878 |
| KRT15 | 0.027820818 | 0.571891561 |
| Y_RNA | 0.046826904 | 1.42918643 |
| TRI-TAT2-2 | 0.044743781 | 1.429107163 |
| HIPK1 | 0.027853394 | 1.168384229 |
| RANP8 | 0.05989153 | 1.428955027 |
| MMP23A | 0.123054196 | 1.428773826 |
| PFN1P7 | 0.084463176 | 1.428660805 |
| ESPN | 0.027864373 | 0.757474947 |
| EIF4EBP1 | 0.027883485 | 0.797938816 |
| SLC16A6P1 | 0.03925991 | 1.428035018 |
| ZNF259P1 | 0.102382181 | 1.428019089 |
| CTC-297N7.9 | 0.103163041 | 1.427918336 |
| PCDH11X | 0.027907897 | 2.463723305 |
| CSNK1G3 | 0.027918243 | 1.130139169 |
| RP11-671E7.1 | 0.007935756 | 1.427652218 |
| AL355480.3 | 0.034468904 | 1.427483789 |
| RP11-370I10.6 | 0.047523968 | 1.427325755 |
| KRT18P31 | 0.038063261 | 1.427064851 |
| CTD-3214K23.1 | 0.018585455 | 1.427002477 |
| SUMF1 | 0.027919758 | 1.175483426 |
| RP11-341G23.4 | 0.023667535 | 1.426127281 |
| RP11-554F20.1 | 0.033218266 | 1.426045307 |
| RP11-736K20.4 | 0.082809283 | 1.425898849 |
| RP11-382A20.6 | 0.05687227 | 1.425869774 |
| MEIS2 | 0.027929619 | 1.274398181 |
| KRT8P51 | 0.073868429 | 1.425516516 |
| HS3ST6 | 0.027931058 | 0.389377322 |
| RP11-435O5.4 | 0.023126304 | 1.425264983 |
| TAS2R46 | 0.027959474 | 1.657834936 |
| ITGB3 | 0.027989001 | 2.373091722 |
| RP11-532F6.4 | 0.075882468 | 1.424928657 |
| RP11-809F4.2 | 0.012614138 | 1.424906346 |
| CTC-428G20.2 | 0.063956288 | 1.424788221 |
| WDR81 | 0.027992068 | 0.855655644 |
| ME1 | 0.028036589 | 0.458373274 |
| RP11-575L7.4 | 0.044107649 | 1.424476867 |
| HMGN2P36 | 0.108704646 | 1.424230484 |
| TNFSF14 | 0.028040087 | 1.617085373 |
| RP11-37B2.1 | 4.47E-05 | 1.423853454 |
| PRKAR2A | 0.028181909 | 1.155959574 |
| MIR4512 | 0.111474029 | 1.423645115 |
| GOT1L1 | 0.028198617 | 4.140412832 |
| RP3-405J10.5 | 0.124251637 | 1.423580702 |
| RP11-1029J19.4 | 0.097001506 | 1.423571752 |
| RNA5SP203 | 0.030775595 | 1.423537273 |
| RP11-227G15.10 | 0.016127274 | 1.423443937 |
| RP11-710F7.3 | 0.012891865 | 1.423325546 |
| RP11-214K3.19 | 0.018652958 | 1.423238258 |
| RSPH1 | 0.028239211 | 2.145661368 |
| RP11-9N20.3 | 0.09233029 | 1.4231908 |
| RP11-933H2.4 | 0.113400253 | 1.423064354 |
| RP11-445N20.2 | 0.131848392 | 1.422458892 |
| TCEAL8 | 0.02825062 | 1.245773866 |
| THAP11 | 0.028328074 | 0.889645109 |
| CCDC154 | 0.028333495 | 2.036447287 |
| RP11-91A18.4 | 0.135070982 | 1.422032842 |
| RP11-302L19.3 | 0.12433955 | 1.421758057 |
| RP11-407N8.6 | 0.063475232 | 1.421280085 |
| PGM3 | 0.028339173 | 1.17671875 |
| FTSJ1 | 0.028373904 | 1.133098785 |
| GJB5 | 0.02840394 | 0.775737476 |
| LINGO2 | 0.028412184 | 0.418676867 |
| AC092641.2 | 0.023016002 | 1.42077071 |
| SLC25A4 | 0.028422547 | 1.219825488 |
| CFAP99 | 0.028572354 | 3.701996878 |
| MTATP8P1 | 0.146892136 | 1.420627605 |
| ALOX12P2 | 0.033052747 | 1.420535273 |
| SNORA36 | 0.140964442 | 1.420381211 |
| RPL7AP4 | 0.063399253 | 1.420143336 |
| HIGD1AP16 | 0.123169565 | 1.420139815 |
| MARK2 | 0.028577991 | 0.881734793 |
| RP11-118H4.1 | 0.130158041 | 1.419754485 |
| KANK3 | 0.028606884 | 0.750694468 |
| USP45 | 0.028625368 | 1.166186019 |
| RP4-630A11.3 | 0.033913914 | 1.419228635 |
| SART1 | 0.02865495 | 0.884282613 |
| LINC00412 | 0.09173789 | 1.419076862 |
| TMEM123 | 0.028677949 | 1.863265194 |
| TSPO | 0.028711174 | 0.854310436 |
| PAN3 | 0.028715228 | 1.131990786 |
| MPPED2 | 0.028821337 | 1.82912843 |
| RP11-258F22.1 | 0.026064971 | 1.418290663 |
| IGFBP7-AS1 | 0.057882879 | 1.418102195 |
| SYP | 0.028851057 | 0.587246987 |
| RP1-159A19.3 | 0.006898597 | 1.417875845 |
| RP11-4B16.1 | 0.051557194 | 1.417831845 |
| ZNF33B | 0.028903317 | 1.193096825 |
| RBMS3-AS2 | 0.071890301 | 1.417682755 |
| C6orf195 | 0.02895278 | 1.390859434 |
| RP11-642A1.1 | 0.117636813 | 1.417432459 |
| MGAM | 0.029034143 | 2.88486294 |
| RP11-817I4.1 | 0.006837176 | 1.417412581 |
| LINC00907 | 0.059575971 | 1.416987797 |
| HNRNPKP3 | 0.123083087 | 1.416823368 |
| RP11-834C11.10 | 0.079191003 | 1.416755591 |
| LINC01068 | 0.056676957 | 1.416675119 |
| RP11-540B6.2 | 0.034078565 | 1.416447391 |
| C4orf27 | 0.029043413 | 1.156049714 |
| CENPO | 0.029049449 | 1.135385923 |
| RP11-432I5.6 | 0.112489265 | 1.416180785 |
| KCNJ6 | 0.029061517 | 2.413513537 |
| ATP11A | 0.029105259 | 1.303614853 |
| RP11-651P23.2 | 0.023008949 | 1.415610858 |
| RP5-1092A3.5 | 0.058088546 | 1.415322478 |
| IMP4 | 0.029117626 | 0.882810493 |
| RP11-181K3.4 | 0.039379921 | 1.414866389 |
| RP11-82L18.2 | 0.093234818 | 1.414685038 |
| OPN1SW | 0.029141795 | 1.231861119 |
| RP11-458D21.1 | 0.021434498 | 1.414579183 |
| LINC01540 | 0.041136099 | 1.414507751 |
| COL6A6 | 0.02915052 | 1.62342038 |
| RP11-175P13.3 | 0.06313051 | 1.414394208 |
| RP11-452J21.2 | 0.086412103 | 1.414198211 |
| AC013717.3 | 0.039248076 | 1.414015247 |
| HNRNPA1L2 | 0.029157791 | 1.19325629 |
| LCNL1 | 0.029283202 | 0.515876054 |
| RP11-2H3.6 | 0.042908425 | 1.41366556 |
| RP11-656D10.7 | 0.013228899 | 1.413619211 |
| CTD-2235C13.1 | 0.109962824 | 1.413540444 |
| LINC00322 | 0.116524872 | 1.413287766 |
| SLC30A2 | 0.029297431 | 3.629820416 |
| MCMDC2 | 0.02931371 | 0.794803269 |
| KIAA1644 | 0.029317804 | 1.969796581 |
| RP1 | 0.029333696 | 2.435494396 |
| C3orf35 | 0.005762686 | 1.412923421 |
| RCN1 | 0.029361603 | 1.222772586 |
| PDK3 | 0.029393722 | 1.204633019 |
| RP11-83B20.3 | 0.121455249 | 1.41243318 |
| XRCC2 | 0.029427548 | 1.192834486 |
| LINC01057 | 0.015135045 | 1.412159862 |
| CTB-111H14.1 | 0.140831289 | 1.411918547 |
| RP11-599J14.2 | 0.109947156 | 1.411588752 |
| PLA2G12A | 0.029452648 | 1.14674367 |
| SERPINA3 | 0.029468456 | 2.623481388 |
| SPATA5 | 0.029474167 | 1.142587206 |
| CDC14B | 0.029482876 | 1.280241615 |
| TAS2R12 | 0.078663583 | 1.410583499 |
| THBD | 0.029525266 | 0.64401976 |
| USP4 | 0.029536669 | 1.135476581 |
| SCARNA9 | 0.04451563 | 1.410189988 |
| THSD4-AS1 | 0.113306822 | 1.409778486 |
| RP11-50B3.4 | 0.048902834 | 1.409729171 |
| RP11-248J18.2 | 0.000430429 | 1.409337046 |
| FAM217B | 0.029548626 | 0.781815446 |
| PFN1P3 | 0.09837842 | 1.409105941 |
| CSTL1 | 0.029575835 | 0.612379179 |
| VPRBP | 0.029652895 | 1.15732403 |
| ZKSCAN8 | 0.029653529 | 1.220514296 |
| RP5-1132H15.3 | 0.057133857 | 1.407477556 |
| CTD-2021J15.1 | 0.128174576 | 1.407203414 |
| RP11-348P10.2 | 0.003090014 | 1.407025768 |
| BEAN1-AS1 | 0.080808721 | 1.406656343 |
| FURIN | 0.029740689 | 1.444122927 |
| RP3-500L14.2 | 0.045943068 | 1.406266875 |
| SRGAP1 | 0.029752705 | 1.272748794 |
| ZNF799 | 0.029799481 | 1.237137725 |
| HSD17B7 | 0.029801303 | 0.876138409 |
| OSCP1 | 0.029808457 | 1.287236387 |
| OR7E128P | 0.021180531 | 1.404983562 |
| ANKRD20A18P | 0.119569817 | 1.40475754 |
| DCDC1 | 0.029811518 | 1.953588091 |
| ARHGAP42 | 0.029819126 | 1.73462814 |
| CTD-2248H3.1 | 0.068487596 | 1.404416564 |
| HNRNPA3P15 | 0.111713356 | 1.404177707 |
| RP11-170N16.3 | 0.020133348 | 1.404092714 |
| CTD-2083E4.5 | 0.098204 | 1.403978866 |
| OPTN | 0.029863029 | 1.177057921 |
| UBE2K | 0.02986699 | 1.122945906 |
| PEX2 | 0.02992837 | 1.133271427 |
| RP11-121A14.2 | 0.035033393 | 1.403682121 |
| OFD1 | 0.029931409 | 1.174032641 |
| DCAF13P3 | 0.013669026 | 1.40333614 |
| RP11-814H16.2 | 0.06466772 | 1.40322926 |
| RP11-303E16.7 | 0.107142582 | 1.40321105 |
| ZCCHC10 | 0.029932833 | 1.101638805 |
| THOC7-AS1 | 0.11983635 | 1.402924327 |
| AL360176.1 | 0.080643549 | 1.4023752 |
| PKN3 | 0.029940692 | 1.300685091 |
| AL354828.2 | 0.029967178 | 0.349508106 |
| AC009237.11 | 0.139778179 | 1.402257124 |
| CSRP3 | 0.02997415 | 4.821763422 |
| LMBR1L | 0.029982167 | 1.127227567 |
| EPHB6 | 0.02998823 | 0.60899713 |
| RNF225 | 0.02999278 | 0.550344517 |
| C5orf58 | 0.030004743 | 1.792393445 |
| OGFOD2 | 0.03005684 | 0.865183663 |
| RP4-534N18.2 | 0.127290981 | 1.401331639 |
| NOTCH2NL | 0.03008546 | 0.748727926 |
| RP1-257A7.5 | 0.086350447 | 1.400588654 |
| CNN2P9 | 0.011753278 | 1.399981737 |
| RP11-405F3.2 | 0.133973556 | 1.399463805 |
| UBQLNL | 0.030087483 | 1.425045794 |
| RP11-29H23.6 | 0.129671706 | 1.399326074 |
| RAD17 | 0.030129435 | 1.108383758 |
| ATP5SL | 0.030132322 | 0.898351789 |
| RHOV | 0.030134473 | 0.719080377 |
| HMGB1P9 | 0.10299168 | 1.398787986 |
| EIF3S5P1 | 0.068708822 | 1.398737219 |
| RP4-725G10.3 | 0.099014552 | 1.398650182 |
| EFCAB12 | 0.03018789 | 2.1440708 |
| AP002954.3 | 0.07156974 | 1.398461632 |
| AC007952.5 | 0.142610995 | 1.398265295 |
| ZNF619 | 0.030200209 | 1.192276094 |
| SGPP1 | 0.030201811 | 1.306831361 |
| SEMA3D | 0.030226859 | 2.33798068 |
| PDS5A | 0.030235852 | 1.130038374 |
| OSBPL2 | 0.030269734 | 0.87871239 |
| LL21NC02-21A1.1 | 0.129787095 | 1.396845629 |
| AGBL2 | 0.030351211 | 1.419216271 |
| RNU6-853P | 0.093032155 | 1.395754669 |
| HPS3 | 0.030368403 | 1.185004677 |
| CMB9-55F22.1 | 0.014169921 | 1.394629504 |
| AC007050.17 | 0.1292326 | 1.394152318 |
| PIGN | 0.03049902 | 0.840243629 |
| C9orf173-AS1 | 0.133763409 | 1.393806435 |
| RP11-58H15.1 | 0.027518198 | 1.393805946 |
| IDSP1 | 0.144254593 | 1.393733378 |
| ZBED3-AS1 | 0.041646517 | 1.39368716 |
| MMD | 0.030517799 | 1.281191565 |
| RWDD4 | 0.030518864 | 1.136446244 |
| CPM | 0.03053466 | 1.499432863 |
| SDR9C7 | 0.030595589 | 0.409391735 |
| ACHE | 0.030740495 | 2.29027704 |
| FAT2 | 0.030742295 | 0.697990309 |
| SAP30L | 0.030762373 | 1.131987028 |
| SERPINB3 | 0.030779095 | 0.556413749 |
| ZNF583 | 0.03081023 | 1.483852377 |
| RP11-285F7.2 | 0.021810235 | 1.392094583 |
| RP4-724E13.2 | 0.094904707 | 1.391297067 |
| GLIPR1 | 0.030871937 | 1.325504927 |
| RP11-219B4.7 | 0.150454793 | 1.390929084 |
| CEP120 | 0.03095482 | 1.153569196 |
| RP11-245P10.6 | 0.08957957 | 1.390882825 |
| TRAPPC2 | 0.030996541 | 1.158924713 |
| RP11-268P4.6 | 0.122682896 | 1.390708932 |
| RP11-366L5.1 | 0.103150098 | 1.390700762 |
| KRT81 | 0.031062503 | 41.99252718 |
| PEX6 | 0.031072999 | 1.225192074 |
| RP11-228B15.4 | 0.070237123 | 1.390414904 |
| PDHA1 | 0.031110702 | 1.138699021 |
| ARSJ | 0.031171563 | 1.470397341 |
| TMEM260 | 0.031233037 | 1.304670709 |
| RP11-863K10.4 | 0.141104374 | 1.389356485 |
| AC104113.3 | 0.038667106 | 1.389240908 |
| CHIC1 | 0.031275131 | 1.236736042 |
| CTD-2024F21.1 | 0.097094283 | 1.389067433 |
| PCP4 | 0.031310816 | 4.611914078 |
| NDUFAF4P1 | 0.14204931 | 1.388836477 |
| RP11-474P2.4 | 0.105070789 | 1.388812183 |
| RP4-597J3.1 | 0.102754925 | 1.388411387 |
| BICD1 | 0.031326364 | 1.261420173 |
| RNF5P1 | 0.151115478 | 1.388337721 |
| ANAPC7 | 0.031342819 | 0.890697618 |
| HMGB1P20 | 0.00200516 | 1.387694071 |
| RP11-686D22.7 | 0.022279204 | 1.387690811 |
| RP11-365F18.3 | 0.056333728 | 1.38754037 |
| RP11-4L24.3 | 0.061419681 | 1.387519818 |
| RP11-373N22.3 | 0.077965703 | 1.38726306 |
| EIF5AP3 | 0.075627915 | 1.387148372 |
| RN7SL403P | 0.083249521 | 1.387034823 |
| RP11-331F4.5 | 0.081755446 | 1.386679257 |
| RP4-782L23.1 | 0.017926313 | 1.386547277 |
| MZT1P2 | 0.151025915 | 1.386124434 |
| AC018442.1 | 0.116978424 | 1.385304411 |
| SEC31A | 0.03144117 | 1.128922001 |
| RP1-130G2.1 | 0.053876299 | 1.385190619 |
| SLC1A4 | 0.03145062 | 1.253029315 |
| RP11-530N7.2 | 0.096159137 | 1.385019741 |
| PAG1 | 0.031467015 | 0.728803897 |
| PRR7 | 0.031573952 | 0.735737641 |
| FLJ26850 | 0.149422196 | 1.384846579 |
| RP11-429P3.4 | 0.079017827 | 1.384771411 |
| CTD-2331H12.5 | 0.129331305 | 1.384717243 |
| RP11-477D19.2 | 0.002475134 | 1.384658207 |
| RP11-346C20.3 | 0.027699315 | 1.384535492 |
| CTC-497E21.3 | 0.03733137 | 1.38446541 |
| RPS3AP4 | 0.107594313 | 1.384163679 |
| SNORA75 | 0.097961513 | 1.384145005 |
| Y_RNA | 0.033852922 | 1.384034646 |
| PRORSD1P | 0.057902159 | 1.383955351 |
| RP11-5G9.6 | 0.11769644 | 1.383837335 |
| RP11-563J2.3 | 0.134153803 | 1.38365381 |
| RP11-531A24.7 | 0.132443537 | 1.383529072 |
| RP4-730K3.3 | 0.012262894 | 1.383249377 |
| AF064858.11 | 0.10793222 | 1.38307281 |
| RP11-324O2.3 | 0.061137866 | 1.382852212 |
| DYRK2 | 0.03158025 | 1.207382963 |
| RP11-75C10.9 | 0.127280223 | 1.381926535 |
| ACAP3 | 0.031580531 | 0.800574747 |
| DDAH2 | 0.031623924 | 1.305053251 |
| AC005077.12 | 0.121704178 | 1.381592198 |
| TADA3 | 0.031650465 | 1.147162424 |
| NEU1 | 0.031662462 | 1.196686149 |
| DIRAS2 | 0.031699998 | 8.563937822 |
| HSD52 | 0.131493418 | 1.380934677 |
| RP11-1365D11.1 | 0.026916957 | 1.380407294 |
| RP11-276E15.4 | 0.021455803 | 1.380288333 |
| CTD-2186M15.3 | 0.005288605 | 1.380117776 |
| RP11-632F7.3 | 0.116218446 | 1.379412207 |
| MAP2K3 | 0.031705763 | 1.194623379 |
| TAPBP | 0.031707112 | 1.201310906 |
| LINC00961 | 0.035628893 | 1.378563325 |
| HNRNPA1P49 | 0.07025964 | 1.378409301 |
| GUSBP5 | 0.100498054 | 1.377730244 |
| RP11-462G12.1 | 0.086605054 | 1.377576806 |
| UBL3 | 0.031726716 | 1.184075697 |
| ERICH6B | 0.031732554 | 1.312896406 |
| RP11-90M2.5 | 0.120214243 | 1.377339701 |
| RP11-452H21.1 | 0.080798611 | 1.377178419 |
| RP11-85G18.6 | 0.140043886 | 1.37712291 |
| RSPH3 | 0.031733127 | 1.153350021 |
| RNU6-925P | 0.069588123 | 1.377067028 |
| AC087380.14 | 0.091601393 | 1.377021456 |
| CTD-2210P15.2 | 0.019494859 | 1.376911664 |
| OGFOD1P1 | 0.082648268 | 1.376911086 |
| HELQ | 0.031739314 | 1.117145892 |
| AC093627.10 | 0.022237549 | 1.376385411 |
| RPS17P5 | 0.066611751 | 1.376341248 |
| MUC6 | 0.031798338 | 9.299470054 |
| RP3-406P24.4 | 0.07986743 | 1.375512723 |
| IMMP1L | 0.031808641 | 1.146327359 |
| PCBP2P1 | 0.114365786 | 1.375366176 |
| AC006273.5 | 0.115243004 | 1.375361468 |
| SERAC1 | 0.031875337 | 1.22443866 |
| CLCF1 | 0.031897893 | 1.48421342 |
| AF196970.3 | 0.060068966 | 1.375081295 |
| ZP3 | 0.031957485 | 0.751101036 |
| RP11-533E19.3 | 0.043476304 | 1.374647681 |
| RP11-413H22.3 | 0.054770276 | 1.374104365 |
| RP11-810P12.1 | 0.118115055 | 1.373844823 |
| PROSER1 | 0.031973202 | 1.15799948 |
| PRPS2 | 0.03200876 | 1.204737632 |
| C9orf117 | 0.032021308 | 1.895139064 |
| SEC11A | 0.032046578 | 1.10835569 |
| C12orf75 | 0.0320721 | 1.397175001 |
| SERTAD4-AS1 | 0.074988558 | 1.373241796 |
| C21orf33 | 0.032074069 | 0.50162014 |
| GATA6-AS1 | 0.138667163 | 1.372732303 |
| RP11-47L3.1 | 0.144356455 | 1.372613511 |
| CYP3A7 | 0.032080085 | 1.498407657 |
| PTMAP4 | 0.038162486 | 1.372245637 |
| KRT83 | 0.032106002 | 3.593138946 |
| ACSL6 | 0.032111381 | 0.429581163 |
| AC012358.7 | 0.061661593 | 1.371977281 |
| HEXDC-IT1 | 0.032686204 | 1.37187404 |
| IL11 | 0.032115207 | 2.938887421 |
| RP11-1024P17.1 | 0.01640687 | 1.371477347 |
| HNRNPA1P14 | 0.015749878 | 1.371471182 |
| RP11-323J4.1 | 0.018527518 | 1.371436108 |
| MAGI1-IT1 | 0.044921218 | 1.371381521 |
| SAP30L-AS1 | 0.009779065 | 1.37137969 |
| CTBP2P8 | 0.03601516 | 1.371287193 |
| AC000078.5 | 0.088954682 | 1.371187177 |
| VSX2 | 0.032120817 | 0.494548285 |
| SCARNA21 | 0.098689593 | 1.370744007 |
| CNPY2 | 0.032146611 | 1.182177495 |
| ASPHD2 | 0.032154069 | 1.270816061 |
| AC005329.7 | 0.140343723 | 1.370326412 |
| ZNF441 | 0.03218219 | 1.271127066 |
| AC093901.1 | 0.150667584 | 1.369908966 |
| ENOPH1 | 0.032235882 | 1.126435098 |
| FMR1 | 0.032236141 | 1.188428591 |
| TMEM220 | 0.032271093 | 1.369188698 |
| RP11-435F17.3 | 0.003510653 | 1.369752714 |
| RNU6-564P | 0.118708964 | 1.369730732 |
| TSPYL4 | 0.032309379 | 1.247569375 |
| DHCR7 | 0.03235259 | 0.790316286 |
| LRRC17 | 0.03236584 | 3.811010397 |
| RNF180 | 0.032368732 | 2.35030141 |
| MIR548AR | 0.075342914 | 1.368826492 |
| RP11-553D4.2 | 0.038045008 | 1.36864546 |
| FOXP4 | 0.032392237 | 1.220261604 |
| CH17-472G23.2 | 0.006552217 | 1.368426569 |
| Y_RNA | 0.032378049 | 1.368390967 |
| CTA-313A17.2 | 0.144528395 | 1.368352044 |
| TBC1D17 | 0.032426543 | 0.868663069 |
| AIM1L | 0.032434074 | 0.699571266 |
| LINC00869 | 0.014226295 | 1.368059687 |
| RTFDC1 | 0.032447055 | 0.90552113 |
| C18orf65 | 0.032516311 | 1.399418302 |
| RP4-539M6.22 | 0.066398256 | 1.367410634 |
| RP11-619I22.1 | 0.070373854 | 1.367332344 |
| LINC01341 | 0.127155962 | 1.367209594 |
| ATP6V0A1 | 0.032520533 | 0.870658659 |
| NPM1P35 | 0.143426761 | 1.366637241 |
| SLC35B3 | 0.032558194 | 1.17542139 |
| TOM1L2 | 0.032605422 | 0.792490551 |
| AE000661.37 | 0.1009305 | 1.366123077 |
| CINP | 0.032615136 | 0.857914687 |
| PRKAB1 | 0.032620492 | 1.300301499 |
| AC004449.6 | 0.091423221 | 1.366017057 |
| RP3-395C13.1 | 0.045493367 | 1.366004571 |
| SPACA6P-AS | 0.114312116 | 1.365577087 |
| RP11-2E11.5 | 0.052689546 | 1.365456801 |
| TPD52L1 | 0.032651639 | 0.752323405 |
| RP11-530C5.2 | 0.092251491 | 1.365099926 |
| CCDC28A | 0.03269351 | 1.158200376 |
| ACTR3C | 0.032715851 | 1.231374388 |
| MIR3176 | 0.10634275 | 1.364795543 |
| HOXC13-AS | 0.131319161 | 1.364461236 |
| CTC-459M5.2 | 0.106149169 | 1.364429254 |
| ACTB | 0.032743939 | 1.112929574 |
| GUCA1B | 0.032769539 | 1.423706055 |
| GOLGA4 | 0.032807845 | 1.179238434 |
| ZNF709 | 0.032819743 | 1.368106504 |
| ZC3H13 | 0.032831877 | 1.172896631 |
| SSUH2 | 0.032832862 | 1.4044881 |
| RP11-383H13.1 | 0.073906028 | 1.363185465 |
| ASS1P10 | 0.107398581 | 1.363002232 |
| RP11-439E19.10 | 0.074384846 | 1.362800541 |
| FBXO16 | 0.032869371 | 1.932771139 |
| RP11-797A18.6 | 0.111799615 | 1.362522221 |
| MLLT4-AS1 | 0.099244519 | 1.362489868 |
| LINC00242 | 0.058604423 | 1.36235661 |
| RP11-666A8.7 | 0.091057778 | 1.362194099 |
| RP11-98D18.16 | 0.036823044 | 1.362187598 |
| RP11-568J23.4 | 0.108522474 | 1.36208402 |
| RUNDC3B | 0.032896621 | 1.630675362 |
| PHYHD1 | 0.032897883 | 1.575902251 |
| C1orf143 | 0.150024533 | 1.36119983 |
| RP11-712B9.2 | 0.002483671 | 1.360978153 |
| AHCY | 0.032902509 | 0.796419065 |
| TSPYL1 | 0.032904924 | 1.13239292 |
| AP4B1-AS1 | 0.059545939 | 1.360461001 |
| CABP5 | 0.032945492 | 2.237867237 |
| SLC39A13 | 0.032945569 | 1.182661769 |
| CTD-2017F17.1 | 0.093839185 | 1.360047973 |
| RP11-79P5.3 | 0.028512006 | 1.359848774 |
| MIR320B2 | 0.059081617 | 1.359799846 |
| MCM8-AS1 | 0.086085053 | 1.359775714 |
| CXorf58 | 0.032959128 | 1.418456045 |
| PXMP4 | 0.032970391 | 0.747238452 |
| CH17-258A22.4 | 0.037020718 | 1.35960406 |
| RPS26P35 | 0.132254877 | 1.359221242 |
| SIX3 | 0.033005225 | 3.069684739 |
| CEP162 | 0.03303417 | 1.205624965 |
| SSX2IP | 0.033056621 | 1.203510746 |
| RNA5SP37 | 0.11604359 | 1.358859541 |
| BLCAP | 0.033058088 | 0.867721768 |
| RP4-751H13.5 | 0.004659255 | 1.358632234 |
| MB21D1 | 0.033095345 | 1.182728187 |
| CTNNBL1 | 0.033117483 | 0.835768795 |
| RP11-214K3.21 | 0.068897361 | 1.358255465 |
| ZC3H11B | 0.135257654 | 1.358144605 |
| DCP1B | 0.033120352 | 1.203634704 |
| RP11-337C18.9 | 0.020569341 | 1.357410011 |
| CTD-2376I4.1 | 0.1154002 | 1.357329752 |
| MRGPRG | 0.033180061 | 20.56026945 |
| RP11-817O13.9 | 0.016228897 | 1.356825435 |
| CXXC4 | 0.033209193 | 1.910938046 |
| Y_RNA | 0.082903006 | 1.356495555 |
| PSMC5 | 0.033214876 | 0.881302592 |
| FOLR1 | 0.033250975 | 2.850141203 |
| RP11-88H12.2 | 0.06199438 | 1.356227619 |
| AP1B1 | 0.033268421 | 0.905071253 |
| RP11-7F18.2 | 0.123358355 | 1.355719353 |
| AC009237.8 | 0.088436345 | 1.355696489 |
| TMC2 | 0.033268914 | 0.644233235 |
| ASCC1 | 0.033280142 | 1.131508644 |
| TSPAN3 | 0.033315962 | 1.353144868 |
| RP11-510N19.5 | 0.122756984 | 1.355218411 |
| CSF2 | 0.033318704 | 2.100827403 |
| ALMS1-IT1 | 0.020685165 | 1.354907985 |
| LANCL3 | 0.033320127 | 0.679647986 |
| RP11-285E9.6 | 0.062956007 | 1.354752613 |
| SCARNA11 | 0.128057899 | 1.354427134 |
| LDHBP2 | 0.145199216 | 1.35438055 |
| VGLL3 | 0.033321657 | 1.939113813 |
| LILRA4 | 0.033348831 | 0.599436382 |
| RP11-367H1.1 | 0.024611853 | 1.354164852 |
| RP11-1007O24.4 | 0.090351077 | 1.353872932 |
| GNL3LP1 | 0.013171204 | 1.353777297 |
| CTD-2619J13.3 | 0.022757717 | 1.353524626 |
| RP11-380G5.3 | 0.081919434 | 1.353458994 |
| RP11-777F6.3 | 0.074349174 | 1.353357945 |
| RP11-94C24.13 | 0.072950715 | 1.353205261 |
| TTC3-AS1 | 0.022184676 | 1.353157359 |
| MAP2K7 | 0.033350136 | 0.892785226 |
| CTD-2024P10.2 | 0.097955376 | 1.353067599 |
| OR5BK1P | 0.13896354 | 1.352961295 |
| LINC01572 | 0.132861746 | 1.352718702 |
| AP006216.10 | 0.087317998 | 1.352589077 |
| STAP2 | 0.033353907 | 0.824424103 |
| RP11-262D11.2 | 0.02542089 | 1.352344378 |
| RBM3 | 0.033354667 | 1.147881017 |
| TRIM29 | 0.033395336 | 0.776949164 |
| PRICKLE2 | 0.033432215 | 1.373387527 |
| CTD-2006C1.6 | 0.015460655 | 1.35194469 |
| PDP1 | 0.033476123 | 1.248532214 |
| RP11-420A6.2 | 0.138836417 | 1.35182656 |
| RP11-727A23.7 | 0.071893943 | 1.351570992 |
| ZMAT1 | 0.033599124 | 1.467322436 |
| LPIN3 | 0.033613546 | 0.804188468 |
| VIT | 0.03362393 | 4.03581878 |
| RP11-384K6.8 | 0.089442865 | 1.351017318 |
| CTC-303L1.2 | 0.035626938 | 1.350908344 |
| SLC36A4 | 0.033629585 | 1.199526147 |
| PDHA2 | 0.033639672 | 5.599632083 |
| ANPEP | 0.03365159 | 2.378256433 |
| TPTE2P5 | 0.078685566 | 1.350515455 |
| KIR2DL4 | 0.033672077 | 1.689411672 |
| ELOVL1 | 0.033698597 | 0.874147088 |
| PRRC1 | 0.033718604 | 1.12407635 |
| RP11-134K13.2 | 0.036843481 | 1.349454112 |
| RP11-120B7.1 | 0.079413787 | 1.349389728 |
| PPP3CB | 0.033777948 | 1.129069706 |
| EXOSC9 | 0.03377824 | 1.130963592 |
| CSF3 | 0.033796918 | 4.987605525 |
| RP11-15A1.8 | 0.070500009 | 1.349061503 |
| RP11-138H8.6 | 0.142004168 | 1.348946926 |
| RP11-193E15.4 | 0.056306253 | 1.348932462 |
| RP3-453C12.15 | 0.117477944 | 1.348922397 |
| TUBB3 | 0.033822601 | 1.875796735 |
| NELFCD | 0.03383084 | 0.884885528 |
| IFT74 | 0.033851362 | 1.167907778 |
| KCNQ1OT1 | 0.022769509 | 1.348379206 |
| RP11-686D22.4 | 0.054808916 | 1.348359006 |
| VDAC1P8 | 0.001333656 | 1.348152514 |
| SHOX | 0.033852287 | 1.708136599 |
| WDR1 | 0.033895808 | 1.132244 |
| VIM-AS1 | 0.062452989 | 1.347793208 |
| RP1-101K10.6 | 0.122255508 | 1.347691975 |
| CBR4 | 0.033928196 | 1.194266024 |
| RP11-443B7.3 | 0.073996448 | 1.347018672 |
| ADRM1 | 0.033954438 | 0.839624989 |
| RP11-367B6.2 | 0.113032305 | 1.346651539 |
| RP11-736K20.6 | 0.094821614 | 1.346501122 |
| PFN4 | 0.03399096 | 1.308531459 |
| ABCC6 | 0.034015896 | 1.741913248 |
| LINC00637 | 0.097069139 | 1.346283898 |
| CCL2 | 0.034084956 | 2.100204563 |
| OTX1 | 0.034109453 | 0.730263198 |
| RGS3 | 0.034114232 | 1.162048373 |
| ISL1 | 0.034118605 | 1.736737773 |
| RP11-523H20.3 | 0.111403748 | 1.345534919 |
| BTRC | 0.034125458 | 0.863133298 |
| STARD4-AS1 | 0.077323324 | 1.345488585 |
| RP11-308D16.2 | 0.044179502 | 1.345259286 |
| PACRGL | 0.034196276 | 1.152737588 |
| OXGR1 | 0.034212847 | 2.498109855 |
| CTD-2017F17.2 | 0.132998482 | 1.344870687 |
| WWC1 | 0.034234675 | 1.269390484 |
| SNORA12 | 0.135092201 | 1.344187808 |
| CCDC114 | 0.034250723 | 2.290707454 |
| HLA-DMA | 0.034260294 | 1.348554475 |
| RP11-239L20.6 | 0.100816048 | 1.343592124 |
| ZDHHC6 | 0.03426451 | 1.11418638 |
| RP11-20B24.5 | 0.126937334 | 1.343123359 |
| RNU6-658P | 0.118699897 | 1.343122381 |
| EPHB3 | 0.034282783 | 0.739573228 |
| RP11-93K22.6 | 0.139545541 | 1.34305253 |
| RP11-143I21.1 | 0.093210293 | 1.343043978 |
| UBE2V1P1 | 0.144176348 | 1.34278417 |
| FAM92A1P1 | 0.029847056 | 1.342642867 |
| ITGA3 | 0.034290967 | 1.373047917 |
| NNT | 0.03429385 | 1.221742865 |
| RP11-1105G2.3 | 0.041121299 | 1.342363294 |
| RP11-156K23.3 | 0.00268548 | 1.342328845 |
| ID2-AS1 | 0.033113027 | 1.341924049 |
| NXF5 | 0.034298579 | 2.055175094 |
| CTB-50L17.14 | 0.034334127 | 1.996523918 |
| HNRNPA1P2 | 0.045658159 | 1.341524314 |
| CDC20P1 | 0.059873662 | 1.341306486 |
| HNRNPA1P28 | 0.057093293 | 1.341040344 |
| RNASEH2B-AS1 | 0.010196598 | 1.340766073 |
| RP11-192M23.1 | 0.04037302 | 1.340759078 |
| RP11-30K9.4 | 0.101135076 | 1.340646721 |
| AP000266.7 | 0.106157398 | 1.340486841 |
| CSMD3 | 0.034407622 | 3.154627547 |
| IFT80 | 0.034419613 | 1.218558475 |
| RP11-490O6.2 | 0.079774573 | 1.339689045 |
| LCA5 | 0.034421639 | 1.261652104 |
| GALNT12 | 0.034425531 | 1.453765319 |
| PIGHP1 | 0.010275887 | 1.339266481 |
| DPY19L1P2 | 0.012363667 | 1.339096632 |
| RP3-466P17.1 | 0.069696812 | 1.338934117 |
| RP11-602N24.3 | 0.10984831 | 1.338491657 |
| TMEM125 | 0.034442676 | 1.459452444 |
| RAB3A | 0.034446498 | 0.788538404 |
| RP11-431K24.1 | 0.075817167 | 1.338208655 |
| PTMAP1 | 0.057953884 | 1.338105015 |
| WDR82 | 0.034453431 | 1.134786545 |
| VDAC3P1 | 0.110333377 | 1.337838444 |
| RP11-227D13.5 | 0.141155438 | 1.337806394 |
| CCDC85B | 0.034463232 | 0.655413817 |
| RP11-65J3.6 | 0.139023318 | 1.337617151 |
| RP11-17E13.2 | 0.066612542 | 1.337490279 |
| CFAP52 | 0.034480291 | 2.349673877 |
| TIGD6 | 0.034506912 | 1.12111142 |
| MLPH | 0.034529611 | 1.679077369 |
| RP1-99E18.2 | 0.027967649 | 1.336698377 |
| RP11-253M7.4 | 0.057759956 | 1.336415392 |
| YAF2 | 0.034604105 | 1.114488955 |
| ALG3 | 0.034638976 | 0.860323482 |
| CH507-254M2.3 | 0.131400463 | 1.336145125 |
| RP11-134K13.4 | 0.035609878 | 1.336126408 |
| MUC16 | 0.034667023 | 1.730764167 |
| CTD-2313D3.1 | 0.107795427 | 1.336052928 |
| TTC21B-AS1 | 0.149562876 | 1.335996091 |
| ATP13A5 | 0.034782834 | 0.375891828 |
| RP11-147L13.7 | 0.113381288 | 1.335794406 |
| HMGB1P10 | 0.001391452 | 1.335659878 |
| PLK2 | 0.03479574 | 1.408695527 |
| GRM2 | 0.034832497 | 1.444731292 |
| RP11-96O20.5 | 0.101171217 | 1.33545888 |
| RP11-612B6.2 | 0.010870583 | 1.335250906 |
| FEM1C | 0.034907376 | 1.149115769 |
| FEZ1 | 0.034923485 | 1.749778261 |
| RP5-1092A3.4 | 0.022925184 | 1.335056365 |
| RP11-1094M14.1 | 0.116616611 | 1.334856978 |
| CTD-2621I17.3 | 0.093991325 | 1.334222204 |
| RP11-61J19.5 | 0.091837582 | 1.334085795 |
| WNT2B | 0.034950876 | 0.626103336 |
| RP11-666F17.1 | 0.063318225 | 1.333495998 |
| RP11-423C15.3 | 0.066685837 | 1.333327009 |
| RP5-1050D4.5 | 0.054994202 | 1.333171128 |
| TUBA4A | 0.034956814 | 0.772762223 |
| SATL1 | 0.034982736 | 0.479104965 |
| SIK2 | 0.035000849 | 1.160027042 |
| RP11-253I19.3 | 0.086830119 | 1.33251518 |
| ZNF445 | 0.035006416 | 1.200382191 |
| KIAA0753 | 0.035012066 | 1.156170947 |
| AC104650.2 | 0.035020153 | 1.659006526 |
| SBF2-AS1 | 0.098522249 | 1.331860445 |
| RN7SKP150 | 0.095069631 | 1.331629209 |
| RP3-329A5.8 | 0.059634053 | 1.331540693 |
| NRXN3 | 0.035051895 | 2.613762561 |
| FAM131C | 0.035077782 | 0.676658773 |
| ANKRD10-IT1 | 0.08653973 | 1.330753814 |
| MYL12B | 0.035082419 | 1.241431536 |
| TGFBR3 | 0.035089449 | 1.751053833 |
| ACSL1 | 0.035137911 | 0.750836902 |
| RP11-373D23.2 | 0.068349677 | 1.330156413 |
| AC009948.5 | 0.006404552 | 1.330074909 |
| RP11-677M14.7 | 0.057930664 | 1.32985551 |
| RP11-43F13.1 | 0.117127631 | 1.329782474 |
| RN7SKP16 | 0.100932493 | 1.329736504 |
| LA16c-390E6.3 | 0.143498957 | 1.329592069 |
| AC005052.1 | 0.06879706 | 1.329534252 |
| CTD-2284J15.1 | 0.046533087 | 1.329509566 |
| ALG9 | 0.035213835 | 1.15425816 |
| RP4-706L14.2 | 0.088375859 | 1.329097524 |
| HSD17B3 | 0.03525956 | 2.050304421 |
| SFR1 | 0.035300464 | 1.177674094 |
| RP11-109E12.1 | 0.137439919 | 1.328563644 |
| TUSC3 | 0.035322026 | 1.479652313 |
| RP11-357N13.6 | 0.117094854 | 1.328377778 |
| CDKN2B | 0.035338118 | 0.755994125 |
| RP11-390B4.3 | 0.092961898 | 1.328311141 |
| PPIB | 0.035363053 | 1.15019639 |
| MORN5 | 0.035379906 | 3.528283712 |
| C3orf30 | 0.035394706 | 3.830699703 |
| FBXO44 | 0.035435779 | 0.832120415 |
| LL22NC03-2H8.4 | 0.131295934 | 1.327821441 |
| TGFBR1 | 0.035521859 | 1.318682652 |
| GNG13 | 0.035531349 | 1.857402038 |
| ISL2 | 0.035533061 | 0.309723426 |
| ADD3-AS1 | 0.127978784 | 1.327372877 |
| MRPS12 | 0.035555949 | 0.763479526 |
| RP11-710F7.2 | 0.060298872 | 1.327217281 |
| CRBN | 0.035574346 | 1.132019933 |
| CAPN6 | 0.035594124 | 4.268125457 |
| CTA-212A2.1 | 0.091043607 | 1.326975846 |
| N4BP1 | 0.03567025 | 0.854503805 |
| PRR27 | 0.035690087 | 3.664295376 |
| FZD10-AS1 | 0.088343547 | 1.326478506 |
| NUDT6 | 0.035690634 | 1.243862316 |
| FAM179A | 0.035690722 | 1.691760266 |
| RP11-138H8.8 | 0.051952703 | 1.325780605 |
| RP11-686D22.8 | 0.040692834 | 1.32571606 |
| RP11-513G19.1 | 0.037518086 | 1.32567762 |
| MUC5B | 0.035719148 | 2.408385831 |
| LA16c-325D7.2 | 0.070955764 | 1.325411985 |
| RP11-755B10.2 | 0.060280888 | 1.325375144 |
| SAG | 0.035724911 | 1.598994978 |
| NDRG2 | 0.035765644 | 0.768413075 |
| RP11-111M22.4 | 0.11811871 | 1.324964454 |
| NR1I3 | 0.035765764 | 0.793617962 |
| CTC-450M9.1 | 0.102861306 | 1.324401591 |
| RP11-672L10.6 | 0.020263781 | 1.324380422 |
| TMEM38A | 0.035766552 | 1.356332158 |
| RGMA | 0.035786874 | 0.665542494 |
| RP11-817I4.2 | 0.013664253 | 1.323752487 |
| CBY1 | 0.035841839 | 1.170868317 |
| RP11-599B13.8 | 0.118534256 | 1.323444575 |
| FZD10 | 0.035862615 | 1.518675532 |
| RP11-707G14.8 | 0.076334245 | 1.323029252 |
| ZBTB11 | 0.035884151 | 0.870838821 |
| HCCS | 0.035897951 | 1.140300693 |
| EIF4A2P2 | 0.035928329 | 1.322587468 |
| SPATA31A6 | 0.035899039 | 0.058616363 |
| RP3-406A7.7 | 0.036814598 | 1.322027375 |
| PGD | 0.035906449 | 0.739809022 |
| ZPBP | 0.03597589 | 2.186593933 |
| GIPC2 | 0.03598872 | 0.737630175 |
| Y_RNA | 0.093056239 | 1.321674691 |
| RP11-650L12.4 | 0.092256014 | 1.32157226 |
| RNU6-875P | 0.068473811 | 1.321334999 |
| RPS3AP34 | 0.10215328 | 1.321293825 |
| RNF219-AS1 | 0.051994887 | 1.321283059 |
| KLC3 | 0.036026342 | 0.681800571 |
| RP11-775D22.3 | 0.012959573 | 1.320974438 |
| RP11-45A17.4 | 0.065367582 | 1.320859971 |
| AMOT | 0.036063239 | 1.531184553 |
| RP11-504P24.9 | 0.065053745 | 1.319765973 |
| RP11-265E18.1 | 0.065997829 | 1.319755149 |
| RP11-78J21.7 | 0.010927288 | 1.319616847 |
| NPM1P26 | 0.044880296 | 1.31955432 |
| RP11-562A8.5 | 0.050920295 | 1.319394648 |
| CSRNP2 | 0.036094649 | 1.155221875 |
| RP11-686O6.1 | 0.14349289 | 1.319173808 |
| ZNF177 | 0.036104988 | 1.888159849 |
| RPS2P41 | 0.130486252 | 1.318762402 |
| COG1 | 0.036110455 | 1.129554876 |
| LDHAP5 | 0.030195431 | 1.31842147 |
| RP11-326C3.7 | 0.012350777 | 1.318245016 |
| RP11-303E16.8 | 0.029101467 | 1.31820941 |
| TMEM194A | 0.036180698 | 1.151207165 |
| RNU5B-2P | 0.051320353 | 1.318032669 |
| RP11-10C24.3 | 0.000758886 | 1.318018394 |
| LINC01277 | 0.056520347 | 1.317919444 |
| UBE2I | 0.036281858 | 1.080541928 |
| RP11-651P23.5 | 0.090596056 | 1.317810962 |
| YIPF5 | 0.036305286 | 1.107648853 |
| ARHGAP30 | 0.036325291 | 0.751571098 |
| CAMK1G | 0.036329076 | 1.417546822 |
| PPP1R9A | 0.036339122 | 1.813562454 |
| GK-AS1 | 0.097452921 | 1.317386715 |
| ZC4H2 | 0.036399973 | 1.776877087 |
| BMS1P1 | 0.032343584 | 1.316839717 |
| FERMT1 | 0.036510738 | 0.756381602 |
| RPL34-AS1 | 0.039037407 | 1.316579367 |
| FAM149A | 0.036557186 | 1.583777316 |
| AC004383.3 | 0.05719083 | 1.316525059 |
| RP11-318C24.2 | 0.029604609 | 1.31618828 |
| RP11-661A12.12 | 0.054701191 | 1.316143591 |
| RP11-734K2.4 | 0.029823804 | 1.316134217 |
| IL23A | 0.03657408 | 1.686257808 |
| DLGAP1 | 0.036581031 | 3.323669386 |
| AGR2 | 0.036610068 | 1.819344176 |
| SREK1 | 0.036619534 | 1.136354395 |
| TMSB4XP1 | 0.025005376 | 1.315529187 |
| FAM193A | 0.036640056 | 1.148011303 |
| PCDHGA9 | 0.036679373 | 1.48454664 |
| RPRD1B | 0.036708066 | 0.867753177 |
| SCARNA8 | 0.120452778 | 1.315099776 |
| RP11-333E1.2 | 0.106074829 | 1.314724818 |
| MLXIP | 0.036724389 | 1.180366397 |
| PDLIM1 | 0.036741721 | 1.212003594 |
| AC007950.2 | 0.136845037 | 1.314506502 |
| ITGA10 | 0.036745871 | 2.753280296 |
| CTD-2035E11.5 | 0.107074601 | 1.314478981 |
| AIFM2 | 0.036765596 | 1.221043451 |
| LINC00398 | 0.132413852 | 1.31435206 |
| CTD-3035K23.7 | 0.068174098 | 1.314328232 |
| DPY19L1P1 | 0.007122824 | 1.31427285 |
| AC005779.2 | 0.036778178 | 1.503683422 |
| SNORD14E | 0.124259024 | 1.313966471 |
| RP11-242D8.3 | 0.091656231 | 1.313899473 |
| EMC3-AS1 | 0.011568865 | 1.31354271 |
| RP11-927P21.2 | 0.147622577 | 1.313419907 |
| C2orf74 | 0.036778608 | 0.725370239 |
| KRT18P63 | 0.034501701 | 1.313145551 |
| RP11-351D16.3 | 0.029998039 | 1.31298146 |
| SLC39A14 | 0.036787489 | 1.27216894 |
| DUSP8P3 | 0.12326148 | 1.312714225 |
| HERC2P10 | 0.084884163 | 1.312396379 |
| AKAP6 | 0.036918708 | 1.318872775 |
| RIPPLY2 | 0.036936643 | 0.342118514 |
| PFDN1 | 0.036981487 | 1.100224151 |
| RP11-503E24.2 | 0.025685716 | 1.312055148 |
| RP11-544A12.4 | 0.098385671 | 1.311957766 |
| STRA6 | 0.036990043 | 1.569072083 |
| STXBP6 | 0.037014082 | 2.99955291 |
| RP11-2E11.9 | 0.013954879 | 1.311584746 |
| AC007038.7 | 0.076133414 | 1.31141016 |
| ZBTB10 | 0.037031241 | 1.433561876 |
| RP11-264J4.8 | 0.128920656 | 1.311289862 |
| LINC01353 | 0.055331392 | 1.311133503 |
| DPF1 | 0.037033996 | 0.510549598 |
| MAD2L1BP | 0.037050035 | 1.1376946 |
| YIPF6 | 0.037119292 | 1.119359723 |
| FAM83H | 0.037143867 | 0.838288565 |
| RP11-314O13.1 | 0.143156075 | 1.30997456 |
| CHPF2 | 0.037193183 | 1.156606126 |
| OR7E7P | 0.015781968 | 1.309538696 |
| CH17-262A2.1 | 0.054263529 | 1.309430251 |
| SYBU | 0.037213709 | 1.458083131 |
| RP11-214O14.1 | 0.131747466 | 1.309227383 |
| TTC29 | 0.037248669 | 9.360437543 |
| TSGA10 | 0.037260208 | 1.253610412 |
| RP11-305O6.4 | 0.061062059 | 1.309016395 |
| FBXL19-AS1 | 0.037034842 | 1.308964186 |
| EVI5 | 0.037275084 | 1.148725079 |
| RP11-440L14.1 | 0.078771336 | 1.30849705 |
| RP11-806L2.6 | 0.129104408 | 1.308308051 |
| RP11-884K10.7 | 0.000433668 | 1.308227159 |
| KBTBD13 | 0.037293897 | 2.387317873 |
| FOXB1 | 0.037343359 | 2.507332271 |
| RP11-739B23.1 | 0.109171768 | 1.307377626 |
| RPL12P15 | 0.060488309 | 1.307341814 |
| RBM47 | 0.037348776 | 1.233550274 |
| ATP6V0E2-AS1 | 0.082558956 | 1.307171149 |
| RP13-216E22.4 | 0.015430614 | 1.307081276 |
| WDR73 | 0.037379821 | 1.152094494 |
| PLCB1 | 0.037397171 | 1.365361735 |
| RPL3P7 | 0.126651404 | 1.30696991 |
| CH17-118O6.2 | 0.024426948 | 1.306890716 |
| CLDN12 | 0.037416292 | 1.187736166 |
| SAMD9 | 0.037432888 | 0.68632027 |
| RP11-368I7.6 | 0.09810753 | 1.3066017 |
| PLD2 | 0.0375038 | 0.832693797 |
| CTD-2116N20.1 | 0.082660484 | 1.306317697 |
| NXT1 | 0.037507114 | 0.847146091 |
| RP11-342F17.1 | 0.136499673 | 1.305754438 |
| SNORD19 | 0.072211288 | 1.305673231 |
| ENSA | 0.037608675 | 0.886794517 |
| TXNDC2 | 0.037663165 | 1.381699251 |
| AKT1S1 | 0.037686077 | 0.882458001 |
| TMEM79 | 0.037703896 | 0.729264556 |
| C9orf153 | 0.037736307 | 1.477959047 |
| ITGA5 | 0.037772337 | 1.483224263 |
| TMEM92-AS1 | 0.145559678 | 1.30458927 |
| RP11-312J18.5 | 0.014164917 | 1.304573305 |
| PRR5L | 0.037863477 | 1.366082081 |
| RHOQP2 | 0.050769396 | 1.304066925 |
| CD44 | 0.037909342 | 0.797251839 |
| RP11-420C9.1 | 0.067915015 | 1.30400677 |
| OR2A4 | 0.037969866 | 4.209460042 |
| ZNF876P | 0.05965156 | 1.303784806 |
| GSS | 0.03801312 | 0.877189201 |
| RP11-359P5.1 | 0.118898714 | 1.30361218 |
| CDKN2B-AS | 0.097596021 | 1.303551321 |
| GBP6 | 0.038021892 | 0.554754929 |
| RP11-632F7.1 | 0.116038824 | 1.303314444 |
| MUC1 | 0.038058297 | 1.605078686 |
| FAM110A | 0.038062514 | 0.826988609 |
| RCN1P2 | 0.048152982 | 1.303076443 |
| RP11-65L3.2 | 0.099907576 | 1.303033576 |
| NADK2-AS1 | 0.12989812 | 1.302969476 |
| MIR548AA1 | 0.081102211 | 1.3029453 |
| SEPT7P9 | 0.076368595 | 1.302745353 |
| CTC-343N3.1 | 0.100336877 | 1.302689931 |
| TXN2 | 0.038072659 | 0.880599994 |
| KCNU1 | 0.038083095 | 2.47570596 |
| XXbac-BPG248L24.12 | 0.021055952 | 1.302509145 |
| RP11-177C12.4 | 0.127338239 | 1.302162383 |
| RP11-380G5.2 | 0.097203045 | 1.302071404 |
| RP1-167A14.2 | 0.066038611 | 1.301998652 |
| RP11-324I22.3 | 0.020671471 | 1.301988732 |
| SORBS2 | 0.038211412 | 1.786825529 |
| RP11-326C3.16 | 0.068264587 | 1.301590077 |
| PHF3 | 0.038253438 | 1.157159794 |
| TRIQK | 0.038257473 | 1.173353561 |
| TTPAL | 0.038285595 | 0.858412591 |
| RINL | 0.038341908 | 0.757703118 |
| RNF183 | 0.038389977 | 1.856465045 |
| RP11-713D19.1 | 0.097880026 | 1.300590958 |
| ACTN1-AS1 | 0.102150001 | 1.300554702 |
| EMC6 | 0.038407849 | 0.827588385 |
| ZNF785 | 0.038437787 | 1.187606559 |
| COL9A2 | 0.038457241 | 1.655075014 |
| RP13-314C10.5 | 0.108860902 | 1.299995383 |
| RCBTB1 | 0.038484996 | 1.299809367 |
| AC011330.5 | 0.099381458 | 1.299887277 |
| C2orf16 | 0.038529162 | 1.220623218 |
| CTC-523E23.3 | 0.018886223 | 1.299740095 |
| FGD1 | 0.038586958 | 1.19454501 |
| AP000925.2 | 0.117230534 | 1.299275733 |
| BTD | 0.038587305 | 1.142837793 |
| RP11-764K9.1 | 0.148556753 | 1.299199054 |
| RP11-395I6.3 | 0.034026301 | 1.299039677 |
| PXN | 0.038635071 | 1.218713586 |
| RP11-109N23.4 | 0.040294307 | 1.298645854 |
| PTMAP3 | 0.069881303 | 1.298400491 |
| CLN5 | 0.038685271 | 1.165862312 |
| AL590762.11 | 0.067633587 | 1.298285309 |
| RP11-1277A3.2 | 0.111455986 | 1.298262699 |
| DEPDC1-AS1 | 0.033582416 | 1.298094482 |
| CCDC107 | 0.038699284 | 1.177963513 |
| SERPINA5 | 0.038721751 | 2.457332851 |
| RP4-657D16.6 | 0.110013945 | 1.29764132 |
| ZBTB7C | 0.038776924 | 0.730658929 |
| CTD-2013N24.2 | 0.0032038 | 1.297561486 |
| SLC25A27 | 0.038786038 | 1.439901072 |
| LDHAP4 | 0.089066172 | 1.29725433 |
| MANSC1 | 0.038816234 | 1.271666568 |
| KAL1 | 0.038827962 | 1.54168385 |
| RPL17 | 0.038838237 | 0.871750802 |
| H2AFY | 0.038874904 | 1.103236862 |
| TRNT1 | 0.038880115 | 1.178362928 |
| OR6A2 | 0.038881797 | 2.812820254 |
| DUSP4 | 0.038883632 | 1.901467649 |
| RP11-473C18.3 | 0.149709175 | 1.296358506 |
| SOX17 | 0.038925938 | 5.14912098 |
| PTP4A2P2 | 0.122583875 | 1.295980651 |
| GALR3 | 0.038987988 | 0.687121186 |
| PPA2 | 0.039019376 | 1.123800761 |
| CNDP1 | 0.039027053 | 3.31926444 |
| AP006621.9 | 0.140750913 | 1.29544445 |
| HNRNPA1P30 | 0.077844106 | 1.295201405 |
| IMPDH1P6 | 0.035838598 | 1.2951084 |
| MIR573 | 0.112217907 | 1.294894924 |
| RP11-213G2.3 | 0.005625985 | 1.294857608 |
| RP11-500G22.4 | 0.13668837 | 1.29478976 |
| RP11-23J9.5 | 0.144254532 | 1.294646488 |
| MORC2-AS1 | 0.142290483 | 1.294533747 |
| RP11-13N13.5 | 0.06626504 | 1.294532341 |
| RP11-422P24.9 | 0.149614263 | 1.294406263 |
| RP11-81H14.3 | 0.101091461 | 1.294404421 |
| FOXC2 | 0.039059419 | 1.907774173 |
| TMEM30B | 0.039090792 | 1.209821371 |
| FUBP1 | 0.039099112 | 1.115096454 |
| SNORD69 | 0.032268448 | 1.293817568 |
| RP11-325L7.1 | 0.142531414 | 1.293696871 |
| RP11-118K6.3 | 0.035200107 | 1.293372661 |
| HSPA12A | 0.039099959 | 1.522895678 |
| LINC00539 | 0.097358864 | 1.292729271 |
| NKX6-3 | 0.039102037 | 31.32958987 |
| CRTC3 | 0.039102737 | 1.163972226 |
| SH3BP1 | 0.039117668 | 0.829201797 |
| CTA-390C10.10 | 0.049877892 | 1.291665689 |
| MYOC | 0.039129644 | 2.438978191 |
| CSE1L | 0.039144503 | 0.884890507 |
| PBX2 | 0.039159193 | 1.140744734 |
| GALK1 | 0.039196409 | 0.725244809 |
| RP11-259P15.3 | 0.125498299 | 1.291050515 |
| SNORA11 | 0.133591777 | 1.290920545 |
| RP11-267M23.1 | 0.022545897 | 1.29047826 |
| RMDN2 | 0.039259607 | 1.229649137 |
| RP11-225B17.2 | 0.001203534 | 1.290300061 |
| RP11-337C18.8 | 0.018842057 | 1.290289809 |
| RP11-261C10.5 | 0.109207681 | 1.29025583 |
| RP11-346C20.4 | 0.07102321 | 1.289849783 |
| RP1-228P16.1 | 0.070729786 | 1.289755413 |
| SLC23A1 | 0.039283617 | 1.732928874 |
| RP1-179N16.6 | 0.137806234 | 1.289704147 |
| RP11-166B2.8 | 0.09138323 | 1.289643253 |
| MTURN | 0.039289173 | 1.370906866 |
| RP11-798M19.3 | 0.102815928 | 1.289413173 |
| HNRNPH1P1 | 0.149862418 | 1.289241327 |
| RP11-9E17.1 | 0.075103139 | 1.289222528 |
| C4BPA | 0.039301163 | 4.812054306 |
| MAMDC2-AS1 | 0.051321072 | 1.289070017 |
| ABL1 | 0.039311949 | 1.135078425 |
| HOXD9 | 0.039314248 | 0.774292966 |
| RP5-1148A21.3 | 0.028542415 | 1.288584286 |
| APOL4 | 0.039346768 | 1.36396616 |
| DLL4 | 0.039361172 | 1.312387963 |
| MEIS1 | 0.039380142 | 1.332073429 |
| LMO3 | 0.039430897 | 2.75481599 |
| CC2D2B | 0.039440193 | 1.351826831 |
| RCAN1 | 0.039486289 | 1.533294965 |
| GAL3ST4 | 0.039492826 | 0.746468421 |
| SLC35A2 | 0.039525894 | 1.128431032 |
| C4orf17 | 0.039535526 | 0.408675288 |
| MIR429 | 0.130424753 | 1.287019618 |
| RP3-391O22.3 | 0.059399566 | 1.286961688 |
| IER3 | 0.039628158 | 1.441271711 |
| MIR3153 | 0.142793322 | 1.28693403 |
| GGNBP1 | 0.02819791 | 1.286873869 |
| RP11-536K7.3 | 0.029728806 | 1.286840493 |
| SERPINB13 | 0.039686807 | 0.570995726 |
| RP11-485M7.1 | 0.068539466 | 1.286466977 |
| RP11-629B11.5 | 0.035838159 | 1.286442767 |
| RP11-90B9.2 | 0.06278702 | 1.286441037 |
| NUDT16P1 | 0.040185991 | 1.286170769 |
| AC011747.4 | 0.131890515 | 1.286057275 |
| ASMTL-AS1 | 0.027225216 | 1.285904355 |
| RP11-333E13.4 | 0.106720019 | 1.28589271 |
| RP11-647O20.1 | 0.025072441 | 1.285885222 |
| CTC-559E9.8 | 0.148300951 | 1.285879519 |
| RP1-20N2.8 | 0.053232157 | 1.285803192 |
| TYW3 | 0.039721271 | 1.118069266 |
| TMSB4XP8 | 0.089742991 | 1.285585519 |
| LALBA | 0.039726966 | 0.470080193 |
| CTD-2210P15.3 | 0.048855052 | 1.284710154 |
| MYLK | 0.039749304 | 1.698249764 |
| KRT16 | 0.039834977 | 0.547676458 |
| CDK5R1 | 0.039861574 | 0.729888671 |
| AGBL3 | 0.039862476 | 1.249728642 |
| RP11-424I19.2 | 0.072563903 | 1.283990461 |
| MAGOH2P | 0.122268574 | 1.283971515 |
| RP11-627K11.6 | 0.080951271 | 1.283791249 |
| RP11-677M14.6 | 0.128291413 | 1.283611818 |
| PRCC | 0.03989651 | 0.912406701 |
| SAMD11 | 0.039898623 | 2.341731441 |
| RP11-708J19.1 | 0.00529942 | 1.283152321 |
| ANKRD28 | 0.039909543 | 1.176321887 |
| RAF1 | 0.039915937 | 1.121124912 |
| KLHDC9 | 0.039969945 | 1.434573836 |
| CTB-36H16.2 | 0.055331014 | 1.282726279 |
| ATG10 | 0.039979337 | 1.140573996 |
| DCDC2B | 0.039994418 | 1.661547104 |
| AC061992.2 | 0.11651939 | 1.282658265 |
| CPE | 0.040009417 | 2.125284339 |
| RP11-164J13.1 | 0.058128898 | 1.282209669 |
| ADAM32 | 0.040056136 | 1.442825608 |
| SNORA31 | 0.081735528 | 1.281760602 |
| LGALSL | 0.04006787 | 0.723641555 |
| CTC-512J14.7 | 0.011411386 | 1.281319901 |
| EVI5L | 0.04009311 | 0.864114389 |
| LRRC23 | 0.040100187 | 1.378602685 |
| FAM227A | 0.040131201 | 1.718638826 |
| GRAP | 0.040162213 | 1.337117595 |
| CHRM3-AS2 | 0.143974517 | 1.280763035 |
| CHGA | 0.040169368 | 34.56997627 |
| RP11-326I11.5 | 0.050894134 | 1.280650807 |
| CTC-436P18.1 | 0.021097103 | 1.280523175 |
| PITRM1 | 0.040173118 | 1.131649556 |
| DNAH9 | 0.040175111 | 2.178751005 |
| LRRC48 | 0.040189827 | 1.368864055 |
| TRAPPC5 | 0.040212244 | 0.702940801 |
| GATA2-AS1 | 0.146167827 | 1.279778961 |
| OXSR1 | 0.040226547 | 1.161022164 |
| SPATA20 | 0.040292557 | 1.204748974 |
| RP11-382D12.2 | 0.137472423 | 1.279494347 |
| LINC01011 | 0.03181218 | 1.279473611 |
| HNRNPDP1 | 0.086432926 | 1.279357494 |
| MCOLN1 | 0.040293111 | 0.896532107 |
| PVRL4 | 0.040306687 | 0.796589808 |
| GNG12-AS1 | 0.027788241 | 1.278876597 |
| NUMBL | 0.040338722 | 0.744162016 |
| PTTG2 | 0.0403624 | 1.416214751 |
| LZTS3 | 0.040503767 | 1.486919175 |
| SDS | 0.040528834 | 0.767125027 |
| KCNE2 | 0.04053056 | 0.774172536 |
| RP11-20B24.7 | 0.068953283 | 1.278400514 |
| RP13-270P17.2 | 0.010179996 | 1.278361178 |
| TEX43 | 0.040539542 | 0.525120857 |
| PLA2G4B | 0.040567128 | 0.71105629 |
| NPL | 0.040601405 | 0.699164273 |
| DRC7 | 0.040617872 | 3.682743399 |
| TVP23C-CDRT4 | 0.040623131 | 1.296827253 |
| DCUN1D4 | 0.04062938 | 1.12379238 |
| RP11-298I3.3 | 0.056631664 | 1.2781261 |
| AL160471.6 | 0.140525936 | 1.278035776 |
| MMP20 | 0.040633085 | 2.203754563 |
| ZCCHC18 | 0.040635202 | 1.448290064 |
| DNAH12 | 0.040678753 | 4.055748239 |
| LINC00106 | 0.070223503 | 1.277186833 |
| C6orf211 | 0.040704036 | 1.143915849 |
| RP11-177J6.1 | 0.084597089 | 1.276846048 |
| TMEM9B-AS1 | 0.015692557 | 1.276836966 |
| PLOD1 | 0.040708052 | 1.218250217 |
| B4GALT4 | 0.040710608 | 0.68144085 |
| RP11-399C16.3 | 0.081014157 | 1.276555698 |
| PPP1R15B | 0.040819238 | 1.124759745 |
| FTH1P1 | 0.140699324 | 1.276240459 |
| RP11-383C6.2 | 0.146057275 | 1.276232338 |
| RAB19 | 0.040858486 | 1.41101089 |
| RP11-139H15.7 | 0.104816956 | 1.275980963 |
| IFT20 | 0.040896388 | 1.112980206 |
| LPAR5 | 0.04090849 | 0.786460229 |
| ARHGEF4 | 0.040922836 | 0.666525817 |
| RP11-10C24.1 | 0.003465212 | 1.275770198 |
| RAPGEF5 | 0.040940301 | 0.725390368 |
| AP000640.2 | 0.124254739 | 1.275675745 |
| TRIM9 | 0.040946965 | 0.535213897 |
| AL138898.1 | 0.140354965 | 1.27545057 |
| RP3-406P24.5 | 0.070050681 | 1.275196606 |
| RP11-359E3.4 | 0.031811831 | 1.274867827 |
| RP11-460I13.6 | 0.064207188 | 1.274773447 |
| RPL36AP43 | 0.064222564 | 1.274753633 |
| PCCA | 0.04095861 | 1.413704839 |
| RP3-425C14.5 | 0.095910843 | 1.274678461 |
| RTP2 | 0.041054595 | 1.971266474 |
| CFH | 0.041097326 | 1.398216363 |
| ZNF713 | 0.041123977 | 1.282701496 |
| SCCPDH | 0.041133127 | 1.345026353 |
| AP2B1 | 0.041155427 | 1.287655521 |
| ETV1 | 0.041190449 | 1.662089455 |
| RP11-603J24.5 | 0.123823301 | 1.27344866 |
| NDUFAF7 | 0.041198103 | 1.113900623 |
| IMPACT | 0.041210179 | 0.854933615 |
| CISD2 | 0.041233281 | 1.111132292 |
| HNRNPH2 | 0.041247478 | 1.122172875 |
| ETV3 | 0.041313604 | 0.813694633 |
| H1FOO | 0.041351401 | 4.31329318 |
| CT62 | 0.041355296 | 1.791163911 |
| PACSIN1 | 0.041463029 | 2.242438524 |
| RP11-365P13.5 | 0.127647397 | 1.272095572 |
| ASRGL1 | 0.041497967 | 1.798180831 |
| CETN2 | 0.041504216 | 1.261461168 |
| IRX4 | 0.041569954 | 0.482837221 |
| LIMCH1 | 0.041608816 | 1.444839436 |
| RP11-158M2.5 | 0.099858264 | 1.271008827 |
| RP13-131K19.2 | 0.110725596 | 1.270948519 |
| RP11-934B9.3 | 0.041616848 | 1.625626545 |
| WASF3 | 0.041652641 | 0.677616602 |
| GMDS-AS1 | 0.012349967 | 1.27061252 |
| ARSH | 0.041703925 | 1.498409875 |
| NNT-AS1 | 0.024538958 | 1.270309601 |
| PPIHP1 | 0.104222197 | 1.270299596 |
| CTD-2515A14.1 | 0.107377283 | 1.270288207 |
| GS1-358P8.4 | 0.020717288 | 1.270233085 |
| CTD-2525I3.2 | 0.092819413 | 1.270011412 |
| LINC01278 | 0.021501912 | 1.26997161 |
| ATP9B | 0.041748788 | 0.873616312 |
| RAB22A | 0.041775305 | 0.878014789 |
| BNIP3P5 | 0.038640605 | 1.269174082 |
| FAM9B | 0.041776028 | 1.727808126 |
| Y_RNA | 0.142913227 | 1.268765479 |
| BTN2A3P | 0.016987607 | 1.268757265 |
| Y_RNA | 0.062663559 | 1.268615606 |
| ANKRD32 | 0.041817977 | 1.152058594 |
| SNRNP27 | 0.041833145 | 1.107409836 |
| TMEM133 | 0.041879401 | 1.618864413 |
| RP5-1159O4.2 | 0.144704302 | 1.267982914 |
| CA5B | 0.041884335 | 1.201090487 |
| CTD-2283N19.1 | 0.025502519 | 1.267749596 |
| SYCE3 | 0.041906236 | 0.58927582 |
| RP11-1143G9.2 | 0.09383945 | 1.266833714 |
| TMEM62 | 0.041941172 | 1.275822232 |
| CXorf49 | 0.041963026 | 2.422704972 |
| FAM189B | 0.041995248 | 0.865119563 |
| PCMTD1 | 0.042016043 | 1.159326107 |
| DDX50P1 | 0.033262589 | 1.266160198 |
| RP11-110I1.5 | 0.138508968 | 1.265798349 |
| TMPRSS11CP | 0.108298738 | 1.265709176 |
| HMGN1P38 | 0.058060325 | 1.265708573 |
| RRH | 0.042044956 | 1.315236082 |
| MRPS31P5 | 0.045192083 | 1.265505742 |
| RP11-109N23.6 | 0.052277938 | 1.265475619 |
| RP11-176D17.3 | 0.104418245 | 1.265462876 |
| AP000442.4 | 0.119323481 | 1.264925974 |
| CARHSP1 | 0.042112218 | 1.333510984 |
| LA16c-431H6.7 | 0.08190844 | 1.264828658 |
| ALDH1B1 | 0.042152521 | 1.265529064 |
| ESM1 | 0.042186148 | 1.655182443 |
| MYT1 | 0.042220262 | 2.354760624 |
| MPC1 | 0.042252558 | 1.194322867 |
| B3GNTL1 | 0.042281995 | 1.182131714 |
| MTMR9LP | 0.09886929 | 1.263518552 |
| RP11-93H24.3 | 0.05457157 | 1.262920232 |
| CTC-463A16.1 | 0.011082647 | 1.262917172 |
| HMSD | 0.042345651 | 0.686005786 |
| FAM182B | 0.042374864 | 0.598457177 |
| ZNF66 | 0.042442467 | 1.726491597 |
| SVIL | 0.042482553 | 0.799627695 |
| MTSS1 | 0.042575027 | 0.731269277 |
| ATRX | 0.04258735 | 1.166277635 |
| THOC5 | 0.042633707 | 0.855444199 |
| ZNF200 | 0.042663266 | 1.119183551 |
| PROM1 | 0.042679178 | 2.225591683 |
| ZNF33A | 0.042730235 | 1.134675257 |
| HCG4P3 | 0.046704453 | 1.261439746 |
| LIMA1 | 0.042741666 | 1.268264978 |
| RP11-46D6.1 | 0.018104363 | 1.261383609 |
| LINC00216 | 0.054683727 | 1.261345043 |
| RP11-181C3.1 | 0.097143329 | 1.261219528 |
| AVEN | 0.042781914 | 1.147831314 |
| PDE8B | 0.04280071 | 1.682597019 |
| RP11-754B17.1 | 0.09522503 | 1.260973617 |
| ABCF3 | 0.042813338 | 0.840260093 |
| PLEKHA8P1 | 0.002080324 | 1.260718615 |
| XXbac-BPG249D20.9 | 0.141081737 | 1.26064832 |
| TPT1P6 | 0.105202656 | 1.260585268 |
| SNORA53 | 0.076606275 | 1.260514294 |
| MKRN2 | 0.042825642 | 1.150153259 |
| RP11-713H12.1 | 0.146681691 | 1.260255848 |
| AL133245.2 | 0.11928916 | 1.26022214 |
| KB-1507C5.2 | 0.138098497 | 1.260151058 |
| ARPC3P2 | 0.13716969 | 1.260129991 |
| STS | 0.042832306 | 1.39826253 |
| MAPKAPK3 | 0.042840752 | 1.136420377 |
| RP4-695O20.1 | 0.112525789 | 1.259818344 |
| WWC2 | 0.042868008 | 1.325091251 |
| RP11-326I19.2 | 0.117474542 | 1.259179222 |
| GDAP1 | 0.042871677 | 0.718122381 |
| CREB3L1 | 0.042931824 | 1.775859982 |
| KLHL30 | 0.042987526 | 1.923478896 |
| CTD-2044J15.1 | 0.13857938 | 1.258965617 |
| PTGDS | 0.042997587 | 0.483345567 |
| CAMSAP1 | 0.043015607 | 0.823391615 |
| LINC01123 | 0.101158995 | 1.258713717 |
| RP11-10O17.3 | 0.026464251 | 1.258591745 |
| RGS13 | 0.043056955 | 1.420690854 |
| MYT1L | 0.043069072 | 4.242599016 |
| TYMP | 0.043085869 | 0.786555819 |
| WDR18 | 0.04309758 | 0.842520041 |
| CTC-428G20.1 | 0.052629982 | 1.257592624 |
| LTB4R2 | 0.043120098 | 0.735025615 |
| HMGN2P18 | 0.125543867 | 1.257397983 |
| ZNF771 | 0.043144134 | 0.754055739 |
| ERAP1 | 0.043144489 | 1.211951573 |
| MDM1 | 0.043167791 | 1.173266326 |
| ERICD | 0.064132937 | 1.257027802 |
| ARAP3 | 0.043182686 | 1.196500715 |
| HCN1 | 0.043198512 | 3.309954495 |
| PI4K2B | 0.043285048 | 1.137720206 |
| DNAJC15 | 0.043323132 | 1.366156128 |
| RP11-454E5.4 | 0.015548929 | 1.256524666 |
| DLEU2L | 0.073867539 | 1.256403769 |
| RP11-401L13.4 | 0.098481885 | 1.256389694 |
| TGM5 | 0.043365181 | 0.458558113 |
| ZNF638-IT1 | 0.135114399 | 1.256102565 |
| PLD1 | 0.043390574 | 0.716857791 |
| NETO1 | 0.043468532 | 2.191474348 |
| LIN54 | 0.043550088 | 1.146846149 |
| CANT1 | 0.043607779 | 1.158297521 |
| RP11-57H14.2 | 0.132394296 | 1.255565832 |
| TEX9 | 0.043627509 | 1.321161626 |
| DIP2B | 0.043643203 | 0.802065615 |
| EPB41L4A | 0.043646326 | 1.227229067 |
| AMOTL2 | 0.043651414 | 1.301429424 |
| SATB1 | 0.043653643 | 1.308175463 |
| SUPT6H | 0.043695465 | 0.911420677 |
| ZNF483 | 0.04369599 | 1.376597354 |
| WWTR1-AS1 | 0.064783517 | 1.25459035 |
| EGFL8 | 0.043709241 | 1.24110829 |
| RP11-314C16.1 | 0.111105247 | 1.254465523 |
| ZNRD1-AS1 | 0.032622428 | 1.254126893 |
| AC000068.5 | 0.045247207 | 1.253800393 |
| FAXDC2 | 0.043722505 | 1.366957849 |
| RP11-277A4.4 | 0.095461744 | 1.253705043 |
| CBX6 | 0.043724158 | 0.755505889 |
| RP1-232L22__B.1 | 0.142177399 | 1.253618534 |
| XAB2 | 0.043789206 | 0.85197144 |
| RP11-380O24.1 | 0.091958594 | 1.253501045 |
| CTD-2270L9.3 | 0.148103267 | 1.253427305 |
| RP11-427L15.2 | 0.056272374 | 1.25339243 |
| C10orf111 | 0.058743368 | 1.253351805 |
| RARA | 0.043789437 | 1.182265744 |
| GLIDR | 0.049570794 | 1.253145761 |
| ANO5 | 0.043861121 | 2.034988154 |
| RP11-240G22.5 | 0.113151515 | 1.253055754 |
| RP11-334C17.3 | 0.054449082 | 1.253049741 |
| ALDH7A1 | 0.043870803 | 1.272111975 |
| NUPL1 | 0.043931466 | 1.15211527 |
| CHMP2B | 0.043985391 | 1.152036723 |
| TTLL6 | 0.043986008 | 2.210944454 |
| TMEM213 | 0.044025211 | 4.926317069 |
| TEX21P | 0.070860786 | 1.252661281 |
| RP11-159N11.4 | 0.055010655 | 1.252633547 |
| NEAT1 | 0.061234438 | 1.252588353 |
| SRL | 0.044048848 | 2.1798103 |
| C11orf24 | 0.044084875 | 1.196233924 |
| DSG1 | 0.044096987 | 0.21979618 |
| FAM98C | 0.044123036 | 0.849285467 |
| CCDC37 | 0.044132651 | 4.902506983 |
| RP11-59H7.3 | 0.065364158 | 1.251799651 |
| MRPS17 | 0.044151133 | 1.462565898 |
| PTP4A2P1 | 0.061686643 | 1.251488682 |
| RBM5-AS1 | 0.105899956 | 1.251429601 |
| CDCA7 | 0.044154403 | 1.242614563 |
| LINC01410 | 0.109874617 | 1.251388245 |
| AC100830.3 | 0.098715233 | 1.251375313 |
| CTU1 | 0.044188724 | 0.791103705 |
| IL18R1 | 0.044198547 | 1.343617443 |
| TP53RK | 0.044225018 | 0.87229172 |
| MIS18A-AS1 | 0.111104674 | 1.250797353 |
| SPDEF | 0.044244951 | 1.977512201 |
| ATP5G2P4 | 0.092490009 | 1.250383535 |
| RP11-317N8.4 | 0.136512488 | 1.250335041 |
| CALCOCO1 | 0.044255263 | 1.146426837 |
| GRHL3 | 0.044278988 | 0.696612068 |
| SRBD1 | 0.044281598 | 1.128848191 |
| RP11-539I5.1 | 0.033380098 | 1.250090266 |
| TCTE3 | 0.044298604 | 1.370460664 |
| GPX8 | 0.044310897 | 1.290305425 |
| MAOB | 0.044336348 | 1.689515188 |
| RP11-650L12.2 | 0.078515048 | 1.249726591 |
| AC114494.1 | 0.04436501 | 1.408161297 |
| ZNF665 | 0.044429654 | 1.456885633 |
| PGPEP1 | 0.044468266 | 1.364252328 |
| HES5 | 0.0444726 | 0.440461219 |
| SLC6A20 | 0.044515911 | 2.758301388 |
| HEG1 | 0.044522798 | 1.511850408 |
| ZNF141 | 0.044539155 | 1.224857471 |
| NUDT4 | 0.044628223 | 1.183965536 |
| ZNF788 | 0.044633113 | 1.61502739 |
| CTB-79E8.3 | 0.031071994 | 1.248538884 |
| TCP11 | 0.04463774 | 1.486462818 |
| QSOX1 | 0.044667642 | 1.219786471 |
| RP3-522J7.7 | 0.099314007 | 1.248283235 |
| RP3-426I6.6 | 0.080523789 | 1.248200433 |
| SEMA6A-AS1 | 0.094758308 | 1.248107702 |
| CD74 | 0.044669516 | 1.352322296 |
| RPSAP9 | 0.111873911 | 1.247722044 |
| FLJ46284 | 0.078100313 | 1.247681682 |
| ATP2B2 | 0.044684433 | 4.085012147 |
| PCK1 | 0.044691196 | 5.907905478 |
| KIAA0319L | 0.044783384 | 1.139159717 |
| ARHGAP6 | 0.044807704 | 1.63912788 |
| FAM169B | 0.044832561 | 11.37089773 |
| HDAC7 | 0.044861365 | 1.138228008 |
| LBX2-AS1 | 0.144026526 | 1.247032119 |
| SPTBN4 | 0.044864397 | 0.602912076 |
| PPM1F | 0.044871065 | 0.818462166 |
| GPI | 0.044890583 | 0.832270518 |
| GH1 | 0.044894976 | 0.598723063 |
| FXYD7 | 0.044952378 | 0.684817076 |
| CTD-2286N8.2 | 0.041516235 | 1.245682401 |
| FNDC3B | 0.044968278 | 1.181594511 |
| TFAP2A-AS1 | 0.067591829 | 1.245391636 |
| GRTP1-AS1 | 0.114829855 | 1.245372645 |
| ARSD | 0.044972279 | 1.327106751 |
| AC112497.1 | 0.009261555 | 1.245068062 |
| DUS3L | 0.045052286 | 0.861141963 |
| MANEA-AS1 | 0.116358144 | 1.244373648 |
| RP11-321F8.4 | 0.099708959 | 1.244371192 |
| RP11-504G3.4 | 0.120989247 | 1.244315821 |
| TTC22 | 0.045072671 | 0.699646981 |
| FOXJ1 | 0.045103602 | 1.9073408 |
| OR10Q1 | 0.045116318 | 3.007323924 |
| LINC00883 | 0.129404476 | 1.243427715 |
| HPS1 | 0.045122916 | 1.122332526 |
| RP11-17M16.2 | 0.066685082 | 1.243094248 |
| SH3BP5-AS1 | 0.063678309 | 1.243007037 |
| ACVR2B-AS1 | 0.067723006 | 1.242953765 |
| VN1R108P | 0.124191662 | 1.242899724 |
| HMGN2P7 | 0.12842043 | 1.242795446 |
| MBD3L1 | 0.045136933 | 2.005411393 |
| RP11-148B6.1 | 0.087928762 | 1.242604342 |
| PDIA4 | 0.045181083 | 1.165258806 |
| RP11-387M24.5 | 0.092139132 | 1.242361939 |
| AC004067.5 | 0.024648014 | 1.241929519 |
| AFF1 | 0.045204085 | 1.197015704 |
| C8orf46 | 0.045256415 | 1.391253579 |
| PIGA | 0.045282814 | 1.161418353 |
| OR8T1P | 0.11252423 | 1.241687731 |
| NIT2 | 0.045411705 | 0.886858794 |
| BRCC3 | 0.045449483 | 1.186834608 |
| EXOSC2 | 0.045482871 | 0.899916033 |
| ACER1 | 0.04549984 | 0.371731899 |
| HOXC4 | 0.045578354 | 1.517632772 |
| GSTCD | 0.045583579 | 1.161187422 |
| RP11-1060G2.1 | 0.14154656 | 1.240876738 |
| FAHD2CP | 0.059721158 | 1.240726214 |
| CDC5L | 0.045590901 | 1.124581414 |
| CAPRIN2 | 0.045621791 | 1.193748787 |
| TBCB | 0.045730879 | 0.820239539 |
| KLF3-AS1 | 0.129399758 | 1.240311966 |
| NDUFS2 | 0.045734933 | 0.909789464 |
| RP11-93O14.2 | 0.096637086 | 1.240015967 |
| STMND1 | 0.045743062 | 3.139230242 |
| RP11-500G22.5 | 0.063144718 | 1.239932747 |
| ZDHHC16 | 0.045781682 | 1.110397545 |
| GYG2 | 0.04581693 | 1.551355325 |
| RP11-715F3.2 | 0.02761539 | 1.239372146 |
| RP11-333E1.1 | 0.062556435 | 1.239360913 |
| DUXA | 0.045884383 | 13.82084149 |
| NDUFB8P2 | 0.142725845 | 1.239193735 |
| SNTB1 | 0.045906141 | 1.655366585 |
| GLUD1P7 | 0.144076722 | 1.239069997 |
| SHANK2 | 0.045910784 | 1.436246747 |
| RP11-705C15.3 | 0.034668076 | 1.238998728 |
| C10orf99 | 0.045918084 | 0.515040869 |
| RP5-858B6.3 | 0.12558665 | 1.23863339 |
| TBCE | 0.045931067 | 0.874407954 |
| EXD1 | 0.045950889 | 0.74090298 |
| NUTM2B | 0.045988903 | 1.283520215 |
| INPPL1 | 0.04599197 | 1.163183943 |
| SLC6A14 | 0.045992727 | 2.044211579 |
| SH3GL1 | 0.046026782 | 0.892764584 |
| SRGAP2D | 0.070790674 | 1.238232524 |
| RP11-398C13.6 | 0.090185645 | 1.238082198 |
| KIAA1919 | 0.04608997 | 1.185556475 |
| SLC43A2 | 0.046146919 | 1.198687161 |
| OCIAD2 | 0.046172778 | 1.246780828 |
| RP11-139H15.6 | 0.129557029 | 1.237717277 |
| SIPA1L1 | 0.046202411 | 1.142858283 |
| IDH3B | 0.046215189 | 0.850797408 |
| RP11-344H11.4 | 0.110661597 | 1.237220549 |
| FLJ31104 | 0.076345234 | 1.237168284 |
| ARHGAP23 | 0.046224237 | 0.764602077 |
| RP11-539G18.3 | 0.143785755 | 1.237045028 |
| RP11-334C17.5 | 0.1010325 | 1.236960616 |
| GCNT1 | 0.046247326 | 1.36808434 |
| RP5-1112D6.4 | 0.124907555 | 1.236766448 |
| DZIP1L | 0.046288522 | 1.339426482 |
| SLC37A1 | 0.046299575 | 1.218970145 |
| GINM1 | 0.046338464 | 1.123354947 |
| CTC-351M12.1 | 0.04511134 | 1.235626392 |
| ST7-OT4 | 0.135083273 | 1.235613145 |
| ALKBH2 | 0.046354786 | 0.840187912 |
| NPM1P9 | 0.043681878 | 1.235109766 |
| FAM46D | 0.046399452 | 3.165602797 |
| HNRNPA1P39 | 0.07930946 | 1.234940301 |
| AC012074.2 | 0.100234233 | 1.234938817 |
| SLC35G1 | 0.046406282 | 0.592286594 |
| RP11-119F19.5 | 0.078084722 | 1.234886381 |
| RP11-395C3.1 | 0.141677123 | 1.23487679 |
| RP11-730A19.9 | 0.099172733 | 1.23485138 |
| ACAA1 | 0.046412766 | 1.129709093 |
| ZNF594 | 0.04646479 | 1.266925388 |
| MAP3K12 | 0.046494101 | 1.252369452 |
| AP1M1 | 0.046509822 | 0.912197522 |
| ZNF610 | 0.046544593 | 1.915663887 |
| DNAJC24 | 0.046567445 | 1.165057127 |
| EIF3B | 0.046648289 | 0.872815041 |
| RBMS2P1 | 0.127029063 | 1.233612591 |
| RP11-61L19.3 | 0.020373825 | 1.23358877 |
| OR2T33 | 0.046659802 | 2.823740165 |
| PIH1D2 | 0.046859614 | 1.355237613 |
| MCOLN3 | 0.046865818 | 1.544276096 |
| CORO6 | 0.046873351 | 1.529841766 |
| RBPJ | 0.04689154 | 1.124079842 |
| RP11-299G20.2 | 0.130894221 | 1.232691469 |
| RP11-490K7.4 | 0.126917607 | 1.232493557 |
| SPACA6P | 0.130401777 | 1.232397714 |
| VN1R81P | 0.136760955 | 1.232246791 |
| AKR1D1 | 0.046935737 | 0.498622608 |
| ARL14EP | 0.04701022 | 1.13206329 |
| SNORD19 | 0.09539372 | 1.232006049 |
| RP5-1112D6.8 | 0.048630935 | 1.231990213 |
| ARSB | 0.047014887 | 1.204241264 |
| SCAP | 0.047078959 | 1.125097727 |
| HOXB6 | 0.047151334 | 1.56280447 |
| STX18-AS1 | 0.017148518 | 1.231799321 |
| BOLA2P2 | 0.120711089 | 1.231789912 |
| MED20 | 0.047274693 | 1.12624751 |
| Y_RNA | 0.10607876 | 1.231601309 |
| AC006160.5 | 0.13944411 | 1.231573656 |
| RP11-179G5.1 | 0.119141723 | 1.231474918 |
| C2orf43 | 0.047293247 | 1.256698052 |
| SLC44A3 | 0.04730501 | 1.274192018 |
| SLC9A9 | 0.04733417 | 0.512782548 |
| AC091633.3 | 0.119391054 | 1.231270832 |
| DUTP6 | 0.095464443 | 1.231136305 |
| INCA1 | 0.04748625 | 1.225101012 |
| IGSF3 | 0.047583007 | 0.766557359 |
| PRSS58 | 0.047601036 | 4.294786442 |
| LIPE | 0.047670872 | 0.769142222 |
| CYSTM1 | 0.047704494 | 1.403863141 |
| C8orf76 | 0.047743332 | 1.110144218 |
| HERC2P2 | 0.029546036 | 1.230473447 |
| RP5-855D21.1 | 0.07578008 | 1.230229218 |
| NPIPA8 | 0.047762018 | 2.072516018 |
| RP11-700J17.1 | 0.02150995 | 1.230191725 |
| DCK | 0.047768986 | 1.1547711 |
| KIAA0232 | 0.047792122 | 1.175775313 |
| AC115617.2 | 0.148270735 | 1.229659551 |
| TPT1-AS1 | 0.047044768 | 1.229652384 |
| ZMYM4 | 0.047793586 | 1.128745352 |
| VPS51 | 0.047813749 | 0.883696931 |
| RP11-97C16.1 | 0.042064483 | 1.229343831 |
| KDM4C | 0.047826527 | 0.638538721 |
| SCRN3 | 0.047847922 | 0.852098659 |
| RP11-1348G14.4 | 0.067663515 | 1.229066853 |
| LCTL | 0.047915451 | 1.249096681 |
| FAM19A1 | 0.047968907 | 0.636408707 |
| AMN | 0.047976363 | 0.661415441 |
| DMKN | 0.047996258 | 0.606848374 |
| SLIT3 | 0.048083205 | 1.673387766 |
| ZRANB2 | 0.048103716 | 1.111067115 |
| ANAPC10 | 0.048131902 | 1.11942143 |
| RP5-901A4.1 | 0.142809071 | 1.228018403 |
| HMGB3P22 | 0.107348422 | 1.227630465 |
| RP11-697N18.3 | 0.107725645 | 1.227564156 |
| TFAP2E | 0.048164045 | 0.739384118 |
| CTD-3065B20.3 | 0.132017466 | 1.227288294 |
| MYO5A | 0.048176279 | 0.767243859 |
| SLC40A1 | 0.048221123 | 1.831115685 |
| GLUD1P3 | 0.041079267 | 1.22708764 |
| ZNF43 | 0.048306267 | 1.485575971 |
| ARRDC3-AS1 | 0.053522812 | 1.226938643 |
| RP11-570G20.1 | 0.119732221 | 1.226816701 |
| TAS2R30 | 0.048320226 | 1.552610351 |
| THEM6 | 0.048387057 | 0.816767799 |
| TMEM89 | 0.048448213 | 1.497585166 |
| TLR3 | 0.048471556 | 1.260269295 |
| TNFAIP1 | 0.048486956 | 1.116860271 |
| BRD8 | 0.048548446 | 1.143657968 |
| DGCR6 | 0.048575099 | 0.65200084 |
| CECR2 | 0.048615892 | 0.506418639 |
| CHCHD4 | 0.048633775 | 1.151108825 |
| SELENBP1 | 0.048645382 | 1.545789018 |
| FANCD2 | 0.048661844 | 1.161061713 |
| ATP5A1 | 0.048684361 | 0.905492213 |
| RN7SL181P | 0.129080019 | 1.224975429 |
| HYOU1 | 0.048727965 | 1.188909859 |
| FBXW4P1 | 0.065365223 | 1.224475394 |
| PLEKHA4 | 0.048737813 | 1.31029269 |
| RP11-521C22.2 | 0.04301276 | 1.22434969 |
| LA16c-380F5.3 | 0.034590051 | 1.224238907 |
| RP11-45L9.1 | 0.089114616 | 1.22422519 |
| BICD2 | 0.048757663 | 0.706758896 |
| MYLIP | 0.04876599 | 1.217990934 |
| MOCOS | 0.048851949 | 1.332855331 |
| GUSBP2 | 0.084689514 | 1.223861246 |
| COBL | 0.048909559 | 1.431892834 |
| PSMG3-AS1 | 0.132687287 | 1.22336566 |
| KLRAP1 | 0.124756724 | 1.223330173 |
| MRGBP | 0.048938836 | 0.875394513 |
| TAS2R14 | 0.048940738 | 1.249503453 |
| PRSS23 | 0.04895239 | 1.405149053 |
| RPS15 | 0.048956665 | 0.806314108 |
| S100A14 | 0.048958398 | 0.808333697 |
| CMTM5 | 0.048993738 | 0.511530648 |
| RP11-420L9.5 | 0.078035757 | 1.221768431 |
| ZNF652 | 0.049015532 | 1.144633423 |
| FSIP1 | 0.049056437 | 1.34925364 |
| RP11-465N4.5 | 0.080413093 | 1.22169277 |
| PRDM13 | 0.049101437 | 0.53682846 |
| ALDH3B2 | 0.049111773 | 0.682523215 |
| CH507-513H4.1 | 0.135318393 | 1.221391316 |
| RNU6-415P | 0.082146113 | 1.221190078 |
| TCP11X2 | 0.0492553 | 8.136018054 |
| ELN | 0.04925575 | 2.043177413 |
| USF2 | 0.049260613 | 0.845750399 |
| PRODH | 0.049277507 | 0.653587358 |
| BNC2 | 0.0492802 | 1.479161847 |
| ZNF346-IT1 | 0.139126949 | 1.220550348 |
| FYCO1 | 0.04929842 | 1.183768738 |
| RP11-4O1.2 | 0.055247227 | 1.220408241 |
| IFI27L2 | 0.049326962 | 0.71085865 |
| RFT1 | 0.049332009 | 1.111213029 |
| RFXAP | 0.049336022 | 1.208838966 |
| KIAA1468 | 0.049372451 | 0.825202498 |
| FHL3 | 0.049378284 | 1.180020842 |
| DCPS | 0.049384351 | 1.256745976 |
| LINC01003 | 0.041231678 | 1.219820871 |
| DRD5 | 0.049396621 | 0.340471887 |
| FMN1 | 0.049401655 | 1.392265886 |
| RP11-726G1.1 | 0.121521927 | 1.219564066 |
| SAMD15 | 0.049416352 | 1.355300817 |
| RP11-644F5.11 | 0.012070086 | 1.219324477 |
| NFE2L2 | 0.049434821 | 0.794031725 |
| C1orf123 | 0.049451062 | 1.101312309 |
| AP000487.6 | 0.10607251 | 1.218951616 |
| PUSL1 | 0.049470644 | 0.816204554 |
| ZNF436 | 0.049485072 | 0.810013299 |
| NPHP1 | 0.049492318 | 1.458988697 |
| SLC22A5 | 0.049513677 | 1.250826564 |
| RP11-100N21.1 | 0.062349114 | 1.218536419 |
| SEC24B-AS1 | 0.040732547 | 1.218290006 |
| CTD-3199J23.6 | 0.040932251 | 1.218275841 |
| NUDT5 | 0.049587297 | 1.104632974 |
| GNE | 0.049631195 | 1.446996118 |
| CTC-510F12.2 | 0.073041793 | 1.218168676 |
| NPPA-AS1 | 0.130575984 | 1.218147012 |
| PAPSS2 | 0.049676069 | 1.423613969 |
| LL0XNC01-237H1.2 | 0.116501487 | 1.217978789 |
| RP11-74J13.9 | 0.035177492 | 1.217855635 |
| RP11-712L6.5 | 0.025352216 | 1.217700087 |
| NOXRED1 | 0.049756892 | 1.219948365 |
| PLEKHA2 | 0.049776958 | 1.167368233 |
| FAM163A | 0.049791414 | 13.29070968 |
| NPM1P37 | 0.058246471 | 1.217118908 |
| AGR3 | 0.049833406 | 2.08734291 |
| PPTC7 | 0.050036001 | 0.846451149 |
| RPL14P1 | 0.112221421 | 1.216702463 |
| NOD2 | 0.050069224 | 0.665910069 |
| KIF16B | 0.050218646 | 0.811614055 |
| RP11-642P15.1 | 0.023451706 | 1.216343983 |
| AHNAK | 0.050300604 | 0.825338052 |
| PRR29 | 0.050300769 | 1.849035418 |
| OR10A3 | 0.050351179 | 2.795617388 |
| RRN3P1 | 0.120549665 | 1.215920134 |
| TOLLIP | 0.050394185 | 0.85839642 |
| MYLK3 | 0.050536945 | 3.654644424 |
| OIT3 | 0.050580588 | 1.745160492 |
| DFNB59 | 0.050614957 | 1.296380939 |
| LINC01024 | 0.036415016 | 1.214877267 |
| TMEM232 | 0.050658541 | 2.112697253 |
| FGD5-AS1 | 0.005344974 | 1.214733613 |
| AC091492.2 | 0.079695789 | 1.214510195 |
| DHRS4L2 | 0.050668679 | 0.855055272 |
| IBTK | 0.050674599 | 1.154394408 |
| SLC37A3 | 0.050703633 | 1.133641575 |
| ZNF213-AS1 | 0.019868562 | 1.214268036 |
| CCND3 | 0.050722211 | 1.116694162 |
| C17orf85 | 0.050732876 | 1.111211226 |
| RP11-585P4.6 | 0.090886573 | 1.213870844 |
| CTC-308K20.3 | 0.138189671 | 1.21367121 |
| SLC22A16 | 0.050781346 | 2.057352195 |
| MAGEB4 | 0.050865047 | 6.156501541 |
| RP11-212I21.4 | 0.132301855 | 1.213287865 |
| RP11-161M6.6 | 0.030517217 | 1.213207414 |
| PLEKHN1 | 0.050894515 | 0.746426533 |
| TEN1 | 0.050894847 | 0.761057558 |
| LRRC63 | 0.050968452 | 1.60321262 |
| RDX | 0.051115977 | 1.195322943 |
| PEBP1P2 | 0.131589677 | 1.21280982 |
| PCDHB12 | 0.051288074 | 1.431053392 |
| TAS2R15P | 0.13484251 | 1.212641315 |
| USP44 | 0.051307204 | 2.306781312 |
| NKTR | 0.051344278 | 1.189561032 |
| THUMPD3-AS1 | 0.022025869 | 1.21248164 |
| RAB40AL | 0.051376015 | 1.328470867 |
| KRI1 | 0.051445038 | 0.86556994 |
| AP000560.3 | 0.1031307 | 1.212218569 |
| C6orf89 | 0.051465865 | 1.10398047 |
| OR10W1 | 0.051499415 | 6.572729701 |
| CYBRD1 | 0.051620206 | 1.299928561 |
| AC009495.4 | 0.143760276 | 1.211857579 |
| PGRMC2 | 0.051630788 | 1.114669904 |
| PLCD3 | 0.051678031 | 1.291438736 |
| NCALD | 0.051733603 | 1.79756529 |
| C10orf120 | 0.051751844 | 3.856896918 |
| PTX4 | 0.051763221 | 3.351989289 |
| AC084117.3 | 0.149322293 | 1.211504336 |
| CFL1P5 | 0.084735987 | 1.211442466 |
| GABRA2 | 0.051820774 | 2.720948669 |
| RASA4 | 0.051855186 | 0.757924939 |
| TRIM38 | 0.051864734 | 1.158010625 |
| PHF6 | 0.051981892 | 1.166369025 |
| TMEM33 | 0.052035463 | 1.14030055 |
| HNRNPD | 0.052083836 | 1.097502444 |
| TM4SF20 | 0.052112141 | 2.519584246 |
| HEXB | 0.052154055 | 1.117967703 |
| KLHL23 | 0.052158855 | 1.288195919 |
| ARMC4 | 0.052200422 | 1.777355759 |
| HMGA1P2 | 0.112219394 | 1.210105446 |
| MED4-AS1 | 0.131534373 | 1.209948492 |
| SLC25A12 | 0.052219074 | 0.892479101 |
| SYNJ2BP | 0.052315207 | 1.116459322 |
| ACTBL2 | 0.052389139 | 1.985551064 |
| RP11-646I6.5 | 0.120025818 | 1.209632099 |
| CTC-444N24.6 | 0.059532928 | 1.209556319 |
| CTD-2587H24.10 | 0.131057377 | 1.209491893 |
| TMEM55B | 0.05240998 | 0.90724417 |
| ZEB1-AS1 | 0.078520047 | 1.209120305 |
| CLHC1 | 0.052502655 | 1.178294675 |
| MTG2 | 0.052513705 | 0.890672185 |
| DCAF8L1 | 0.052549916 | 5.586259591 |
| AOC1 | 0.05255619 | 2.560800197 |
| AC007566.10 | 0.056319655 | 1.208786595 |
| LACC1 | 0.052574472 | 1.146872716 |
| WNT8A | 0.052615105 | 2.975682403 |
| C1orf177 | 0.052623475 | 0.658349717 |
| TMEM135 | 0.0526669 | 1.143674513 |
| ACTBP9 | 0.132957969 | 1.20835035 |
| ANKRD45 | 0.0526837 | 2.470388007 |
| RASAL2 | 0.052787482 | 0.813049919 |
| CEP83-AS1 | 0.101747695 | 1.207997051 |
| SGMS1-AS1 | 0.048386894 | 1.207767776 |
| MSX2 | 0.052796874 | 3.230725428 |
| C2orf50 | 0.052814518 | 2.031866979 |
| APOC2 | 0.052829504 | 0.632210962 |
| PSMF1 | 0.052910789 | 0.896878034 |
| MEIOB | 0.052939451 | 0.527324901 |
| FAM71E2 | 0.052994434 | 4.711972354 |
| TIPARP-AS1 | 0.140384349 | 1.207016933 |
| MECP2 | 0.053013531 | 1.145432101 |
| UXT-AS1 | 0.04612918 | 1.206904554 |
| DALRD3 | 0.053036298 | 1.154520068 |
| MLIP | 0.05307207 | 0.570425215 |
| FGF11 | 0.053094497 | 0.685115383 |
| NXF2B | 0.053121942 | 16.41782557 |
| ASIC4 | 0.053124359 | 0.674960764 |
| EPHA7 | 0.053125269 | 4.309896697 |
| AOX1 | 0.053146936 | 1.688223343 |
| HPGDS | 0.053154538 | 0.711698225 |
| SLC39A7 | 0.053308857 | 1.13132922 |
| NKX1-1 | 0.053330445 | 4.863629384 |
| BCL2L15 | 0.053358974 | 1.935815557 |
| AASDH | 0.053396271 | 1.12986957 |
| AL133243.2 | 0.021223713 | 1.204837301 |
| DDX23 | 0.053412304 | 1.083310527 |
| CCDC6 | 0.053432657 | 1.168777132 |
| SUGT1 | 0.053440185 | 1.125194097 |
| BACE2 | 0.053475894 | 1.36481319 |
| RP11-474P2.6 | 0.135408512 | 1.204252189 |
| EFTUD1 | 0.053514272 | 1.120610523 |
| PCDHGA2 | 0.053519904 | 1.390423127 |
| SLC35F3 | 0.053521304 | 2.768725922 |
| C9orf173 | 0.053531592 | 1.565827989 |
| PHLPP1 | 0.053609432 | 0.822177919 |
| RP1-20C7.6 | 0.13432368 | 1.203417196 |
| ANTXR1 | 0.053626543 | 1.286511163 |
| OR1D2 | 0.053641637 | 0.08879807 |
| DNAJC10 | 0.053825519 | 1.206530377 |
| RP11-359B12.2 | 0.14637898 | 1.20330265 |
| ANKRD1 | 0.053897848 | 2.976668137 |
| SLC30A7 | 0.053942286 | 1.109208401 |
| C15orf57 | 0.053995988 | 1.129346008 |
| GRM8 | 0.054013921 | 3.294196823 |
| HLA-DOB | 0.054040603 | 1.412421098 |
| PI4K2B | 0.054129595 | 1.968660567 |
| CEP135 | 0.054196623 | 1.171723127 |
| LSM11 | 0.05421057 | 1.112550794 |
| PTPN1 | 0.054220221 | 0.857690546 |
| LINC00094 | 0.070810351 | 1.201663075 |
| MORN4 | 0.054270322 | 1.354176278 |
| PAK6 | 0.054274317 | 0.769365121 |
| C9orf24 | 0.054281068 | 2.963315987 |
| C6orf136 | 0.054311479 | 1.100859501 |
| RP11-977G19.12 | 0.102260951 | 1.201264785 |
| HIST1H4A | 0.05438102 | 0.619076413 |
| RP11-529E10.6 | 0.122107401 | 1.200648914 |
| ITGA9 | 0.054445326 | 1.348094641 |
| RP11-216B9.9 | 0.111660685 | 1.200325266 |
| RP11-380B4.3 | 0.07039744 | 1.200119115 |
| SCAMP5 | 0.054446004 | 0.730503229 |
| CIDEB | 0.054468107 | 0.827898721 |
| CFAP58 | 0.054492022 | 1.609796882 |
| LINC00467 | 0.10355891 | 1.199834844 |
| RP11-950C14.3 | 0.134366379 | 1.199756478 |
| TRIM23 | 0.054531451 | 1.111457123 |
| PLA2G4F | 0.054582906 | 0.61115077 |
| GPR12 | 0.054619663 | 9.173277597 |
| DYNC1LI1 | 0.054752188 | 1.121821566 |
| NEXN | 0.054777881 | 1.373705256 |
| SURF6 | 0.054787051 | 0.901943765 |
| SRD5A3-AS1 | 0.134665008 | 1.198760632 |
| NES | 0.05481143 | 0.749155098 |
| EVA1A | 0.054842873 | 1.700127987 |
| RP5-991G20.1 | 0.069060997 | 1.198631355 |
| RP11-242D8.1 | 0.080016875 | 1.198554083 |
| TIA1 | 0.0548574 | 1.130913085 |
| AC022007.5 | 0.098354727 | 1.1984735 |
| TRAM2-AS1 | 0.065391855 | 1.198378278 |
| ALDH3B1 | 0.054907945 | 1.466272587 |
| ABHD1 | 0.054926286 | 0.726086818 |
| YTHDC1 | 0.054929327 | 1.097867805 |
| ALG11 | 0.05492942 | 1.179862505 |
| GJD3 | 0.054934766 | 0.462407129 |
| SLC35A1 | 0.055066627 | 1.164434025 |
| UGDH | 0.055107183 | 1.422373298 |
| USP22 | 0.055143605 | 1.133691037 |
| LARP7 | 0.055147046 | 1.109332788 |
| ALG1L6P | 0.120051532 | 1.19786644 |
| SLC38A6 | 0.055153734 | 0.868749967 |
| HHIPL1 | 0.055185069 | 0.50961393 |
| TJAP1 | 0.055213002 | 1.131510523 |
| MEN1 | 0.055241315 | 0.909737632 |
| ZNF365 | 0.05526997 | 0.577729865 |
| WEE2-AS1 | 0.12572022 | 1.197292423 |
| CTD-3195I5.3 | 0.084378169 | 1.197280132 |
| ARCN1 | 0.055355829 | 1.118582892 |
| RP11-206L10.1 | 0.08468345 | 1.197152391 |
| RELL2 | 0.055430809 | 0.752859288 |
| RP11-30K9.6 | 0.13523159 | 1.197107991 |
| ABCC3 | 0.055462949 | 1.57404921 |
| MOB3C | 0.055480479 | 0.852993959 |
| CENPJ | 0.055501955 | 1.143915021 |
| ARPC5 | 0.055588158 | 1.076713126 |
| SBSN | 0.055648486 | 0.330909019 |
| FAIM2 | 0.055661045 | 2.085906874 |
| OR52N2 | 0.055669708 | 4.34373913 |
| TLE1 | 0.055756768 | 1.180347774 |
| LRRC10 | 0.055780649 | 4.786538012 |
| FAM27C | 0.055814454 | 1.424730124 |
| HDAC9 | 0.055887347 | 1.866014171 |
| HINT1 | 0.05591912 | 1.125054206 |
| NPPB | 0.055971119 | 4.060053994 |
| UCP1 | 0.05599403 | 2.141941757 |
| UTP20 | 0.056019957 | 1.158527214 |
| SH3BGR | 0.05603208 | 1.310092786 |
| FSTL4 | 0.056121993 | 0.572614553 |
| RP5-994D16.9 | 0.012862023 | 1.195349519 |
| ANGPTL6 | 0.056139948 | 0.828689573 |
| LYZL4 | 0.056151908 | 2.377261202 |
| MIR3685 | 0.138241259 | 1.195300827 |
| AC007228.9 | 0.085889283 | 1.195253039 |
| ADCY1 | 0.056159896 | 0.628430205 |
| RNF139-AS1 | 0.085684492 | 1.195161148 |
| GORAB | 0.056245613 | 1.157537743 |
| RP4-622L5.7 | 0.126049734 | 1.194992819 |
| DYNAP | 0.0562677 | 0.372860244 |
| RNF186 | 0.056276257 | 2.784298379 |
| SERINC4 | 0.056290962 | 1.709343974 |
| REL | 0.056299592 | 0.714264301 |
| STK19 | 0.056371522 | 1.105414021 |
| CHAT | 0.056466804 | 0.107966638 |
| CTD-2623N2.3 | 0.118819226 | 1.194415056 |
| CDRT4 | 0.056513388 | 1.301825108 |
| C1orf234 | 0.056568068 | 0.707806493 |
| FAM86DP | 0.059040848 | 1.194216734 |
| TPT1 | 0.056571135 | 1.151496261 |
| MEOX1 | 0.056678267 | 0.633518621 |
| ST7-AS1 | 0.112202785 | 1.194017926 |
| HLA-F | 0.056692462 | 1.295630586 |
| RAB40A | 0.05672005 | 2.176501592 |
| IFT172 | 0.056722413 | 1.193709602 |
| C12orf76 | 0.056734858 | 1.119495734 |
| DSC1 | 0.056824386 | 0.30063702 |
| NUDT12 | 0.056832891 | 1.212392856 |
| FBXO21 | 0.056864886 | 1.112978323 |
| GK | 0.05697026 | 1.230487721 |
| HSP90B1 | 0.056978951 | 1.113254471 |
| ERAS | 0.057104244 | 1.638053891 |
| ITPR3 | 0.057140535 | 1.193163758 |
| FZD4 | 0.057171014 | 1.703888935 |
| DNAH17 | 0.057178028 | 0.407376826 |
| GLYATL1 | 0.057179705 | 2.759872436 |
| SLC25A1 | 0.057251626 | 0.874368557 |
| RP11-424C20.2 | 0.099178994 | 1.192937096 |
| VASN | 0.057321411 | 1.335613158 |
| PHLDB1 | 0.057348645 | 1.278603089 |
| SLC10A5 | 0.057357919 | 1.341717609 |
| MGAT4A | 0.057364592 | 1.256365825 |
| RP5-1024G6.2 | 0.02735585 | 1.192641376 |
| CTC-241N9.1 | 0.046152382 | 1.192616856 |
| PDIA3P1 | 0.036309519 | 1.192570537 |
| RP11-79P5.10 | 0.070050527 | 1.192546449 |
| LTB4R | 0.057375411 | 0.749697322 |
| SPATA2 | 0.057408647 | 0.892913727 |
| RP11-10N23.2 | 0.132362678 | 1.192268713 |
| RP11-337N6.3 | 0.052703438 | 1.192209807 |
| ALPP | 0.057416318 | 9.650564477 |
| CAAP1 | 0.057486151 | 1.113474344 |
| VAV2 | 0.057535469 | 1.218837017 |
| NRGN | 0.057704259 | 1.377348912 |
| ACSM5 | 0.057822607 | 0.48516496 |
| ZNF20 | 0.057911972 | 1.24726227 |
| ING3 | 0.057945149 | 1.126850735 |
| ZFP2 | 0.057970664 | 1.643294279 |
| RP11-81A1.6 | 0.132218874 | 1.191638285 |
| LETM2 | 0.058000829 | 1.364871307 |
| LINC00899 | 0.05940377 | 1.191218804 |
| STK24P1 | 0.085454032 | 1.191113329 |
| RP4-613B23.1 | 0.118180466 | 1.191075634 |
| GMEB2 | 0.058117097 | 0.907923863 |
| CCNG2 | 0.05814383 | 1.210347457 |
| NREP | 0.058182982 | 1.292697125 |
| SLC25A44 | 0.058196659 | 0.909317273 |
| ABI2 | 0.058249846 | 1.190193222 |
| RP11-120M18.2 | 0.133206558 | 1.190125088 |
| AP001258.4 | 0.023780192 | 1.190123352 |
| AL357515.1 | 0.140359285 | 1.190121476 |
| RP1-234P15.4 | 0.112564353 | 1.190051891 |
| RBM12B-AS1 | 0.150265656 | 1.189921968 |
| LINC00672 | 0.102641025 | 1.189855557 |
| KHSRP | 0.058276524 | 0.889795034 |
| C16orf71 | 0.058296666 | 1.390348749 |
| RP1-12G14.7 | 0.119659032 | 1.189479728 |
| RPP40 | 0.058408885 | 0.820516288 |
| CEP85 | 0.058439463 | 0.869500782 |
| RP11-142E9.1 | 0.090051913 | 1.189045897 |
| CYP26A1 | 0.058470952 | 0.333136674 |
| FZD5 | 0.058502966 | 1.297781857 |
| GPR137C | 0.058566272 | 1.258825613 |
| GALNT6 | 0.058580357 | 1.375052576 |
| SPATA31A3 | 0.05859311 | 0 |
| MYRIP | 0.058639143 | 1.820335704 |
| DACT2 | 0.058727969 | 1.794980067 |
| C17orf75 | 0.058731344 | 1.105993182 |
| SMPDL3A | 0.058770392 | 0.721624645 |
| RAB9B | 0.058858261 | 1.578792012 |
| CORO2B | 0.058872769 | 1.637380419 |
| TMX4 | 0.0588783 | 1.20893622 |
| SLBP | 0.058974542 | 1.120470751 |
| NEK1 | 0.058978152 | 1.14974638 |
| CMTM4 | 0.059016294 | 1.221491235 |
| PITRM1-AS1 | 0.054591263 | 1.188024558 |
| RP11-544A12.8 | 0.148549967 | 1.187884246 |
| GAREM | 0.059075153 | 0.827377408 |
| CLUHP3 | 0.080155706 | 1.187772171 |
| SNX5 | 0.05909799 | 0.908170166 |
| APOO | 0.059105071 | 1.136032683 |
| MTHFS | 0.082327016 | 1.187550914 |
| EFTUD2 | 0.059105423 | 0.918814585 |
| TMEM104 | 0.059115071 | 0.880659825 |
| EIF2S2 | 0.059139966 | 0.881104383 |
| MSI2 | 0.059228762 | 1.205113314 |
| OTOA | 0.05925118 | 0.526093685 |
| SNRPGP14 | 0.147067112 | 1.187423248 |
| SH3BP4 | 0.05929281 | 1.202441801 |
| MARCKSL1 | 0.059305157 | 1.228553458 |
| RNU6-722P | 0.085949089 | 1.18717812 |
| CTD-2256P15.4 | 0.141353449 | 1.187134622 |
| WHAMM | 0.059340319 | 1.113481128 |
| RP11-392P7.6 | 0.142248431 | 1.187061939 |
| NIPSNAP3A | 0.059345315 | 1.133508972 |
| CPOX | 0.059363692 | 0.852737058 |
| ANGPTL1 | 0.059373676 | 1.93187882 |
| GTF2H4 | 0.059385058 | 1.142232854 |
| ZNF254 | 0.059402751 | 1.349704369 |
| FBXW7 | 0.059455988 | 1.115962031 |
| RP11-673C5.1 | 0.020891081 | 1.186510418 |
| NTSR1 | 0.059458343 | 1.966301212 |
| RP11-799M12.2 | 0.137935748 | 1.186441171 |
| LINC01560 | 0.059691994 | 1.186189205 |
| MYOM3 | 0.059478099 | 1.742987357 |
| ADAT3 | 0.059508015 | 0.800777272 |
| RP11-5C23.1 | 0.066238178 | 1.18604048 |
| LRRC37A16P | 0.061470805 | 1.185987377 |
| FAM3C2 | 0.144192755 | 1.18595627 |
| NMB | 0.059565603 | 0.783263585 |
| FMOD | 0.059568075 | 1.436351281 |
| KYNU | 0.059594513 | 1.480997121 |
| RP11-562A8.4 | 0.106704549 | 1.185517664 |
| RP3-368A4.6 | 0.115969407 | 1.185498165 |
| AC008155.1 | 0.140596717 | 1.18536551 |
| RP11-10K16.1 | 0.110618663 | 1.185354784 |
| CASQ2 | 0.059605827 | 0.644553866 |
| LAMB4 | 0.05962473 | 2.440582207 |
| MVD | 0.059635732 | 0.822023064 |
| GNL3 | 0.059666784 | 1.157276434 |
| RP13-270P17.1 | 0.097857984 | 1.185023678 |
| SDHA | 0.059671962 | 1.230518308 |
| CUEDC1 | 0.059674817 | 1.277770639 |
| FKBP1B | 0.059685981 | 1.352394748 |
| CTC-529I10.2 | 0.078987817 | 1.184822411 |
| TANK | 0.059723069 | 1.110229158 |
| HMGN2P17 | 0.063516409 | 1.184672176 |
| CDC42EP3 | 0.05979366 | 1.355341116 |
| MMP25-AS1 | 0.128732407 | 1.184597253 |
| SLC14A1 | 0.059837728 | 5.246676423 |
| SUZ12 | 0.060001471 | 1.11429576 |
| SOAT1 | 0.060017862 | 1.261005932 |
| CATSPERD | 0.060067421 | 2.728134386 |
| C1orf35 | 0.06011286 | 0.882645654 |
| EPHA4 | 0.060159075 | 0.661110308 |
| NPR1 | 0.060231475 | 2.507672159 |
| IFT88 | 0.060256212 | 1.198274094 |
| RFX5 | 0.060297233 | 1.161767617 |
| DHX34 | 0.060308045 | 0.877283596 |
| DNAJB13 | 0.060432307 | 1.87814196 |
| FAM127B | 0.060446693 | 1.147141103 |
| FAM199X | 0.060497277 | 1.133746472 |
| RWDD2A | 0.060546142 | 1.184604334 |
| HYDIN | 0.060593645 | 2.885482436 |
| WDFY1 | 0.060633565 | 1.16180271 |
| EPB41L1 | 0.060648493 | 0.793938649 |
| CPSF4L | 0.060673785 | 1.663049269 |
| LENG1 | 0.060689595 | 0.843827042 |
| KRT39 | 0.060694104 | 5.146007175 |
| PTPN23 | 0.060772155 | 1.121331323 |
| SLC25A20 | 0.060866944 | 1.166765773 |
| ATG4A | 0.060910982 | 1.12720433 |
| MRPS31P4 | 0.096824486 | 1.182643261 |
| C9orf135 | 0.060930786 | 6.218515008 |
| MAATS1 | 0.060935906 | 2.085245974 |
| KIAA1614 | 0.060944867 | 1.257189128 |
| CCDC175 | 0.060948218 | 2.656275275 |
| ARL1 | 0.061056367 | 1.105155307 |
| PEX11A | 0.06109245 | 1.1940261 |
| SCARNA15 | 0.136298416 | 1.18188971 |
| RP13-104F24.3 | 0.106476293 | 1.181770098 |
| CD274 | 0.061171553 | 0.575203689 |
| WIZ | 0.061277446 | 0.903231212 |
| UBOX5-AS1 | 0.136354567 | 1.181556274 |
| NPTXR | 0.061322605 | 1.628322217 |
| CLEC4C | 0.061400565 | 0.608035818 |
| GIP | 0.061528085 | 5.97846544 |
| CHRNA7 | 0.06154103 | 1.626078718 |
| MYCBP2-AS1 | 0.128924654 | 1.180942523 |
| LRRC8A | 0.061628977 | 0.802858071 |
| MIER3 | 0.061630597 | 1.130938804 |
| ZBTB47 | 0.061649123 | 1.16167441 |
| C21orf59 | 0.061785691 | 1.141756301 |
| PARD6G | 0.061793766 | 0.772941199 |
| PVRL1 | 0.061798759 | 0.77204677 |
| EEF2KMT | 0.061844465 | 0.897021402 |
| PPP1R32 | 0.061876278 | 1.314443626 |
| OR10H1 | 0.06190411 | 2.348799312 |
| CTA-276F8.1 | 0.086797846 | 1.180392097 |
| GRAMD4 | 0.061920253 | 0.850572307 |
| BAIAP2L1 | 0.061922169 | 0.882810745 |
| RAB11FIP4 | 0.061939058 | 1.238074916 |
| ICAM1 | 0.062026215 | 1.332579099 |
| RP11-410L14.2 | 0.093796703 | 1.180325426 |
| SPTBN2 | 0.062053974 | 0.78247593 |
| C12orf56 | 0.062054429 | 0.655122641 |
| AGXT | 0.06217593 | 0.248187718 |
| ECT2L | 0.062233476 | 1.635634447 |
| ALKBH6 | 0.062237322 | 0.84595574 |
| DNAJC12 | 0.062296802 | 2.213116759 |
| SMARCC1 | 0.062300251 | 1.161850732 |
| PQLC2L | 0.062313935 | 2.493246805 |
| SPATA21 | 0.062344202 | 9.647604144 |
| TAMM41 | 0.062421472 | 1.127078139 |
| MANBAL | 0.062427471 | 0.875956593 |
| ATP13A2 | 0.062492154 | 0.858255126 |
| RARA-AS1 | 0.048711603 | 1.179074915 |
| PLGRKT | 0.062517723 | 0.553239561 |
| PRR16 | 0.062652513 | 1.519926002 |
| ZNF514 | 0.062704194 | 1.155176109 |
| HRASLS2 | 0.06270663 | 1.710919652 |
| EVA1C | 0.062804155 | 1.282935213 |
| AP4B1 | 0.062835663 | 1.169755937 |
| E2F2 | 0.063061655 | 0.80938636 |
| KRT76 | 0.063140123 | 0.172647789 |
| RHBDF2 | 0.063213002 | 0.849397912 |
| JRKL | 0.063215701 | 1.249988118 |
| RAI14 | 0.063250289 | 1.255521665 |
| GDF2 | 0.063260806 | 0.209809417 |
| CWC27 | 0.0633146 | 1.093365451 |
| NXT2 | 0.06335298 | 1.171708024 |
| COL25A1 | 0.063411858 | 4.084641833 |
| TPPP3 | 0.063508707 | 1.597437424 |
| RP11-96O20.4 | 0.063530297 | 0.687548138 |
| CYP26B1 | 0.063576157 | 0.676647518 |
| ZNF512 | 0.063720603 | 1.146216429 |
| EPB41L4A-AS2 | 0.083981164 | 1.176992632 |
| SNAP47 | 0.063721628 | 0.910836134 |
| LRGUK | 0.063743205 | 1.461475895 |
| GPNMB | 0.063758172 | 0.641294873 |
| TRAPPC11 | 0.063792263 | 1.107180294 |
| CARD18 | 0.063844818 | 0.341861731 |
| SUPT7L | 0.063857735 | 1.087825615 |
| LAMB1 | 0.064027539 | 1.328892864 |
| PRDX1 | 0.064088609 | 0.887400519 |
| LRP8 | 0.064165788 | 0.792034283 |
| CYCS | 0.064176386 | 0.854885937 |
| MATN1-AS1 | 0.146377725 | 1.17584242 |
| RP11-632K20.7 | 0.122488449 | 1.17583121 |
| RBMS3 | 0.064190512 | 1.354774515 |
| ASAH2B | 0.064194116 | 1.139587742 |
| KCTD3 | 0.064230227 | 0.812238864 |
| PON2 | 0.064236734 | 1.225844273 |
| ARL10 | 0.064291372 | 0.810156488 |
| MYBL1 | 0.064354937 | 1.26090219 |
| DNAJB2 | 0.064365637 | 0.849304599 |
| FAM87B | 0.133131075 | 1.174976667 |
| RP11-147L13.11 | 0.016894798 | 1.174701711 |
| HDAC5 | 0.064396488 | 0.853403661 |
| MFAP2 | 0.064423144 | 1.328916154 |
| FBL | 0.064430968 | 0.800033533 |
| TMEM59 | 0.064486372 | 1.10237866 |
| HEXIM2 | 0.064543927 | 1.150998219 |
| EIF3FP3 | 0.13768227 | 1.174266458 |
| HLA-F-AS1 | 0.143373457 | 1.174216619 |
| ZMYM2 | 0.064622875 | 1.146503914 |
| RUNDC1 | 0.064657384 | 1.393404109 |
| RP11-705C15.2 | 0.062617965 | 1.173990582 |
| MITF | 0.064658193 | 1.326647146 |
| RP11-288E14.2 | 0.148195443 | 1.173483426 |
| RP11-257O5.2 | 0.026743078 | 1.173476337 |
| ABHD16A | 0.064695168 | 1.146112812 |
| ULK1 | 0.064718829 | 0.872753333 |
| SLC35C1 | 0.064769396 | 1.227263825 |
| ANKRD35 | 0.064863363 | 0.451179878 |
| SEC16A | 0.064867856 | 1.115797408 |
| AC026271.5 | 0.042528321 | 1.173018028 |
| KIAA2026 | 0.065039738 | 0.631313965 |
| CDK14 | 0.06510995 | 1.327306185 |
| APOOL | 0.065114581 | 1.16202873 |
| HMGN2P4 | 0.114248558 | 1.172750524 |
| DNAAF1 | 0.065183282 | 1.793621271 |
| ACSS3 | 0.065201453 | 3.521765427 |
| ABCA11P | 0.106235391 | 1.172432901 |
| CCDC71L | 0.065201567 | 1.206982243 |
| CHAMP1 | 0.065290025 | 1.15456139 |
| SAV1 | 0.065302999 | 1.113178183 |
| LRRC34 | 0.065312644 | 1.478923053 |
| SNCAIP | 0.06532535 | 1.546864347 |
| RP11-259K5.2 | 0.092788929 | 1.171727911 |
| PANX3 | 0.065326535 | 2.813953651 |
| PRSS56 | 0.065331112 | 11.2584893 |
| DNAJC5 | 0.065416724 | 0.875707338 |
| BAG2 | 0.065519936 | 1.317853031 |
| VEGFA | 0.065533856 | 1.243262831 |
| SHMT1 | 0.065589611 | 1.181510155 |
| MECOM | 0.065609942 | 1.2782848 |
| C1orf86 | 0.065701306 | 0.780363165 |
| CYP2S1 | 0.065709794 | 0.595341913 |
| UTP14C | 0.065729378 | 1.164118016 |
| EIF4EBP2 | 0.065875711 | 1.098918242 |
| FN3K | 0.065925863 | 1.297215011 |
| RP11-756P10.6 | 0.137949929 | 1.170637964 |
| UHRF2 | 0.065977371 | 0.641795919 |
| FSTL1 | 0.065986381 | 1.402159352 |
| ADAMTS5 | 0.065996294 | 1.885736955 |
| EFNA4 | 0.06600298 | 0.860327057 |
| RP11-390P24.1 | 0.06775121 | 1.170112136 |
| PPID | 0.06601403 | 1.127601253 |
| RP3-323P24.3 | 0.150950352 | 1.169754381 |
| CHST7 | 0.066024516 | 0.767318386 |
| ZNF518A | 0.066111528 | 1.223919532 |
| RP11-1379J22.5 | 0.101934279 | 1.169362431 |
| CEP44 | 0.066118821 | 1.142706452 |
| PLA2G2C | 0.066136418 | 2.164642893 |
| BPIFB1 | 0.066166021 | 3.852891802 |
| RBM20 | 0.066167174 | 1.79111916 |
| SPATA18 | 0.066183336 | 1.266196689 |
| RBBP8NL | 0.066190628 | 0.773316163 |
| TLX3 | 0.06621572 | 0.674344796 |
| LRFN1 | 0.066225472 | 0.690978906 |
| EVX2 | 0.066282833 | 0.609088531 |
| RP11-88E10.5 | 0.144768306 | 1.168310487 |
| MAGED2 | 0.066300829 | 1.178463419 |
| RASSF3 | 0.066338058 | 1.141394408 |
| MKNK1 | 0.06643938 | 0.896841311 |
| NDRG4 | 0.066451991 | 0.52557802 |
| TMEM185B | 0.066516376 | 0.877827313 |
| SPATA9 | 0.06654714 | 1.259033131 |
| ZNF275 | 0.066580116 | 1.239466965 |
| C6orf1 | 0.066595974 | 0.850157679 |
| RP11-1072C15.4 | 0.145063589 | 1.167241232 |
| XPC | 0.066612623 | 1.138964804 |
| NCAM2 | 0.066628876 | 0.27152329 |
| EDDM3B | 0.066642687 | 5.931098336 |
| GSTK1 | 0.066678199 | 1.156030106 |
| THAP9-AS1 | 0.018169614 | 1.166309939 |
| BCAR3 | 0.066700592 | 1.289749941 |
| CALHM1 | 0.066707174 | 2.177391667 |
| CCR4 | 0.066874252 | 0.611714429 |
| FCHO2 | 0.066951756 | 1.143108933 |
| PDZD8 | 0.066964512 | 1.239216185 |
| FLT3 | 0.06706863 | 0.662309469 |
| GOLGA8H | 0.067079526 | 1.262100862 |
| KB-1208A12.3 | 0.137855403 | 1.165782672 |
| SCMH1 | 0.067080549 | 1.14910181 |
| SNX8 | 0.067091033 | 0.880357762 |
| CTA-941F9.10 | 0.121434684 | 1.165557439 |
| UMPS | 0.067101553 | 0.887810535 |
| FUT11 | 0.06723046 | 1.115421805 |
| ARL3 | 0.067306767 | 1.200077205 |
| KRTAP5-6 | 0.067307348 | 0.536916795 |
| HNRNPA1P4 | 0.149604582 | 1.165202353 |
| SPDL1 | 0.067326879 | 1.126038417 |
| PRDM7 | 0.067330594 | 0.565737483 |
| RCN2 | 0.067334397 | 1.152847222 |
| RUNX2 | 0.067353351 | 1.258989917 |
| HAL | 0.06736701 | 1.615648708 |
| RP11-342K2.1 | 0.106772529 | 1.164686246 |
| N4BP2L2 | 0.067393155 | 1.11651342 |
| NMT1 | 0.067436932 | 0.926999366 |
| EAF2 | 0.067471183 | 0.839181973 |
| KPRP | 0.067491807 | 0.301551845 |
| CECR5 | 0.067510083 | 0.890338905 |
| TIMM10B | 0.067554804 | 1.104546596 |
| RP13-104F24.2 | 0.123500124 | 1.164050709 |
| HRG | 0.067588358 | 0.123283152 |
| SUCLA2 | 0.06760691 | 1.123761977 |
| TMEM151A | 0.067700046 | 0.324073595 |
| ZNF618 | 0.067755337 | 1.262201173 |
| SAYSD1 | 0.067757341 | 1.115125552 |
| DPF3 | 0.067778673 | 1.284660041 |
| PTCRA | 0.067886192 | 0.478864285 |
| KCNJ14 | 0.067886301 | 0.78445404 |
| RP11-10C24.2 | 0.106296972 | 1.162936045 |
| MST1R | 0.067895307 | 1.309082351 |
| KLRC1 | 0.067959728 | 1.457413497 |
| PLD4 | 0.067990105 | 0.708653681 |
| CAT | 0.068075055 | 1.17126653 |
| FCF1P2 | 0.033111366 | 1.162409986 |
| QRFP | 0.06811952 | 0.710597177 |
| KRTAP9-9 | 0.068157936 | 6.146634671 |
| UTP3 | 0.068251717 | 1.093192782 |
| ANKRD66 | 0.06830063 | 7.017514722 |
| TMEM106A | 0.068382425 | 1.193158658 |
| FAM9C | 0.068399924 | 9.196961741 |
| SPINK13 | 0.068426212 | 11.50797193 |
| POU3F4 | 0.068429891 | 0.257933405 |
| TRAF3IP2-AS1 | 0.071773501 | 1.161922035 |
| EFEMP1 | 0.068451046 | 1.919022544 |
| FRYL | 0.068481733 | 1.120150017 |
| PABPC4L | 0.068524245 | 1.528892174 |
| CCNA1 | 0.068542651 | 4.659575304 |
| DST | 0.068566307 | 1.471000311 |
| RP11-950C14.10 | 0.147215338 | 1.161696818 |
| MBD1 | 0.068588363 | 0.902549733 |
| OPRL1 | 0.068597491 | 0.671275148 |
| RP13-516M14.1 | 0.119156607 | 1.16161384 |
| CARKD | 0.068644089 | 1.107101653 |
| PRR3 | 0.068667861 | 1.147359934 |
| SMARCA2 | 0.068703562 | 0.723630449 |
| DMRT2 | 0.068737612 | 0.551826771 |
| SNED1 | 0.068756572 | 1.367856855 |
| VWA8 | 0.068850412 | 1.146831549 |
| ANXA10 | 0.068897775 | 4.638571119 |
| SEPHS1 | 0.068916967 | 1.115754848 |
| SLC18B1 | 0.068997047 | 1.211372328 |
| TNFRSF10B | 0.069096163 | 1.166293674 |
| ARHGAP32 | 0.069146387 | 0.796496252 |
| CATSPER2P1 | 0.051163006 | 1.1606184 |
| ZNF133 | 0.069155682 | 0.868391714 |
| C14orf80 | 0.069196901 | 0.828832079 |
| HGD | 0.06921349 | 2.135393796 |
| PINK1-AS | 0.014594267 | 1.160487141 |
| CDKN2A | 0.069216044 | 0.822006494 |
| SELO | 0.069225807 | 0.896474534 |
| KCNE5 | 0.069253577 | 2.242732075 |
| LY6D | 0.069269061 | 0.656825107 |
| CTD-2008E3.1 | 0.147663781 | 1.160389077 |
| USO1 | 0.069293738 | 1.116821333 |
| RP11-278C7.1 | 0.044771994 | 1.160051134 |
| NPIPA3 | 0.069330592 | 1.381559274 |
| CDH8 | 0.069334235 | 3.165403845 |
| WDR89 | 0.069337146 | 1.094041548 |
| SLC5A8 | 0.069411355 | 3.472219844 |
| CD177 | 0.069449806 | 2.595507312 |
| ZCCHC12 | 0.069544526 | 2.065516117 |
| SC5D | 0.069577153 | 0.800735762 |
| GABRA5 | 0.069693115 | 0.126820078 |
| ASB17 | 0.069715034 | 8.908727244 |
| ZNF487 | 0.069756961 | 1.234139423 |
| CHRM4 | 0.069805888 | 1.485622103 |
| PPIAL4C | 0.069836292 | 1.50331393 |
| INHBA | 0.069875273 | 1.588242567 |
| SLC20A2 | 0.0699388 | 0.790651063 |
| KRT4 | 0.06999092 | 0.34813798 |
| MSLN | 0.070087097 | 1.639336034 |
| CEP76 | 0.070152727 | 1.121804655 |
| SLFN12 | 0.070160855 | 1.253229083 |
| LHX6 | 0.070192627 | 0.764851262 |
| S1PR3 | 0.070208146 | 1.397707752 |
| ATP6AP2 | 0.070290354 | 1.107433347 |
| AC010976.2 | 0.150626809 | 1.157636894 |
| MUC5AC | 0.070396205 | 3.509699847 |
| ATP8A2 | 0.070439086 | 1.81863652 |
| MAPKAPK5 | 0.070460521 | 1.079315222 |
| PYY | 0.070513944 | 2.795561443 |
| AC005229.1 | 0.079324378 | 1.157472873 |
| ZIM3 | 0.070519525 | 2.441076923 |
| BFAR | 0.070551832 | 1.116185189 |
| ECHDC3 | 0.070639635 | 0.762158841 |
| AADAC | 0.070741011 | 0.432160929 |
| CD97 | 0.070756708 | 1.266432963 |
| TNFRSF13B | 0.07088278 | 0.605075297 |
| TEAD2 | 0.070894121 | 1.240563575 |
| SERPINA4 | 0.070923112 | 3.450164888 |
| ARMCX2 | 0.070936703 | 1.462309407 |
| PARD3 | 0.070942663 | 0.855932628 |
| SMARCAD1 | 0.070943382 | 1.126306123 |
| ARL13A | 0.070993345 | 1.268334175 |
| FSHR | 0.071029667 | 3.511063646 |
| SORBS1 | 0.071048555 | 1.402023222 |
| GREB1 | 0.071126191 | 1.764744353 |
| HOXA9 | 0.071194013 | 0.71967293 |
| HABP4 | 0.071235279 | 1.280434859 |
| KIF24 | 0.071239217 | 1.16212496 |
| TCP10 | 0.071254655 | 3.302441276 |
| NOBOX | 0.071255136 | 15.13036072 |
| FAM216B | 0.071301125 | 9.847020157 |
| ZNF782 | 0.071317096 | 1.144167081 |
| ZNF575 | 0.071484688 | 0.848268343 |
| ACAD9 | 0.071516759 | 0.898424674 |
| AGPAT1 | 0.071525551 | 1.123915179 |
| FTSJ3 | 0.071577863 | 0.922632373 |
| FLRT3 | 0.071623379 | 1.452420208 |
| RP11-77K12.7 | 0.071646491 | 3.032828346 |
| HCG18 | 0.024543763 | 1.155330153 |
| PRKD2 | 0.07165481 | 0.913612406 |
| CALU | 0.071698936 | 1.156322282 |
| RP11-186N15.3 | 0.13546865 | 1.155264399 |
| CABLES1 | 0.071744451 | 1.348003916 |
| SSTR4 | 0.071776006 | 0.372838817 |
| FLT1 | 0.071867303 | 1.286950148 |
| ARNT2 | 0.071963059 | 1.773553082 |
| ADORA2A | 0.071991585 | 0.718087654 |
| AAED1 | 0.071993145 | 1.151218789 |
| SERPINB7 | 0.07200009 | 0.565300959 |
| ARL4A | 0.07201147 | 1.205107061 |
| PPT1 | 0.072115838 | 0.889864612 |
| CAPN7 | 0.072116128 | 1.145792909 |
| HNRNPAB | 0.072164619 | 1.095550994 |
| RP11-506M13.3 | 0.14737125 | 1.154331798 |
| MYCL | 0.072215573 | 0.687491684 |
| RP11-541N10.3 | 0.149838444 | 1.154186438 |
| AP000442.1 | 0.057718813 | 1.153984296 |
| FTX | 0.1315906 | 1.153950618 |
| GRK4 | 0.072228423 | 1.149720501 |
| LIPC | 0.072290663 | 3.017839005 |
| C17orf51 | 0.072334602 | 1.280929719 |
| HNF4G | 0.072376299 | 1.649857975 |
| RP11-421L21.3 | 0.135639052 | 1.152786139 |
| TMEM52B | 0.072409981 | 1.85081865 |
| FAM26D | 0.072603005 | 3.513787759 |
| LY96 | 0.072646107 | 0.757311432 |
| RASSF5 | 0.072665986 | 0.801764379 |
| AK7 | 0.072685117 | 1.54709535 |
| RYR1 | 0.072699131 | 0.769739766 |
| DDX31 | 0.072728863 | 0.9125254 |
| C16orf96 | 0.072879671 | 0.647697326 |
| AC019206.1 | 0.072971356 | 1.315887825 |
| CCDC74B | 0.07297374 | 1.618645668 |
| PDE4A | 0.072993941 | 1.237364076 |
| SHCBP1L | 0.073102726 | 1.563018674 |
| PCDH9 | 0.073212871 | 3.156036013 |
| ADAD2 | 0.073259136 | 0.667763246 |
| DLEU1 | 0.073269979 | 0.851226825 |
| DLG4 | 0.0732928 | 1.33608539 |
| RBMX | 0.073342984 | 1.071229653 |
| RP11-488L18.10 | 0.113345481 | 1.151744089 |
| ZNF333 | 0.073371106 | 1.12888177 |
| PSMD2 | 0.073372557 | 0.886793118 |
| PUS10 | 0.073472751 | 0.805572416 |
| EHBP1L1 | 0.073488025 | 0.877984693 |
| TXN | 0.073534219 | 0.772719552 |
| NODAL | 0.073611566 | 1.97169879 |
| CXCL1 | 0.073689229 | 1.840508422 |
| GFOD1 | 0.073762147 | 0.819214876 |
| NBR2 | 0.109867632 | 1.150949443 |
| PDGFRL | 0.07376342 | 1.362714948 |
| MFSD8 | 0.073787307 | 1.11222513 |
| ENOSF1 | 0.073832796 | 1.136782435 |
| PIGL | 0.073865629 | 1.139326504 |
| GLIPR1L1 | 0.073913247 | 1.237803316 |
| FBXO30 | 0.073971937 | 1.136926835 |
| GRAMD1C | 0.074082602 | 1.23132706 |
| TUBA8 | 0.074120246 | 1.291085156 |
| FOXC1 | 0.074153944 | 1.30544607 |
| TMEM14B | 0.074156852 | 1.140441774 |
| CEP78 | 0.074306047 | 1.112324667 |
| PGC | 0.07436538 | 3.943307362 |
| ASPG | 0.074386489 | 0.571587288 |
| SLC14A2 | 0.074489301 | 2.706181036 |
| PRR15L | 0.074493082 | 1.5013888 |
| EIF2AK4 | 0.07455166 | 1.1316404 |
| SMIM21 | 0.074573765 | 3.118377085 |
| ACOT9 | 0.074598259 | 1.16733536 |
| MORF4L1 | 0.074606779 | 1.086211821 |
| TGOLN2 | 0.074651436 | 1.140292427 |
| RP11-977G19.11 | 0.100129352 | 1.148501714 |
| FGF18 | 0.074688138 | 2.609096492 |
| TMEM261 | 0.074688454 | 0.604680541 |
| TCEAL3 | 0.074700438 | 1.221754815 |
| C9orf47 | 0.074711087 | 1.586565942 |
| BAMBI | 0.074721617 | 2.466712702 |
| ACKR2 | 0.074736796 | 1.729144896 |
| FAM204A | 0.074772019 | 1.100914231 |
| GNPTG | 0.074772745 | 1.100223394 |
| ZNF658 | 0.074777246 | 0.823937818 |
| RARRES1 | 0.074799229 | 2.349297801 |
| SNRPGP2 | 0.070245895 | 1.147459593 |
| PRMT8 | 0.074801337 | 1.81111662 |
| HS2ST1 | 0.07494545 | 1.104348145 |
| KCTD21 | 0.074981253 | 0.856082176 |
| ATG4B | 0.075010796 | 0.877208337 |
| KLF10 | 0.075012302 | 1.152476546 |
| MPV17L | 0.075013675 | 1.899808696 |
| ATG3 | 0.075033505 | 0.902749645 |
| RUNDC3A | 0.075049898 | 1.846535585 |
| UBE4B | 0.07506433 | 0.856609881 |
| SPOCD1 | 0.075073288 | 1.436300797 |
| ABHD14B | 0.075171795 | 1.191845628 |
| P2RX4 | 0.075305682 | 1.175466352 |
| TPM2 | 0.075309979 | 1.279765612 |
| EBLN2 | 0.075313223 | 1.273093229 |
| ASCC2 | 0.075391993 | 0.915191718 |
| ZNF396 | 0.075419722 | 1.214275902 |
| PHF7 | 0.075435199 | 1.178837136 |
| METTL20 | 0.075450023 | 1.147758327 |
| NRD1 | 0.075455717 | 0.904880865 |
| OCIAD1 | 0.07548368 | 1.093093941 |
| CACUL1 | 0.075498095 | 1.098526284 |
| RETNLB | 0.075535071 | 0.362837318 |
| MME | 0.075566024 | 2.092666316 |
| ZMYND12 | 0.075569413 | 1.401174554 |
| GNAI1 | 0.075584338 | 0.813739247 |
| SOCS3 | 0.075623451 | 1.281095728 |
| MSC | 0.075629528 | 1.42079627 |
| ZWINT | 0.075647009 | 1.137735499 |
| PFKFB2 | 0.075652124 | 1.442857209 |
| PSEN2 | 0.075685345 | 1.197778015 |
| TIMM50 | 0.075697694 | 0.843239755 |
| MTRF1 | 0.075716176 | 1.148359607 |
| CTNND1 | 0.075768622 | 0.863651516 |
| SETD5 | 0.075769126 | 1.131512773 |
| KCNN2 | 0.075841303 | 1.535657929 |
| PRAMEF18 | 0.075853644 | 5.831760803 |
| MRPS5 | 0.075907776 | 0.901111338 |
| LCAT | 0.075942939 | 1.278769802 |
| RBM26-AS1 | 0.146758232 | 1.144187479 |
| LATS2 | 0.075982138 | 1.194491927 |
| YIPF1 | 0.075983452 | 1.09428773 |
| RAB5B | 0.075987259 | 1.085566911 |
| KLHL14 | 0.075991271 | 2.132222331 |
| CTA-253N17.1 | 0.128836828 | 1.143882269 |
| SLC22A11 | 0.076010825 | 1.669033167 |
| F3 | 0.076019388 | 1.559677737 |
| TCEB3B | 0.076024821 | 0.635606225 |
| DYRK1A | 0.076036093 | 1.147653847 |
| TMEM253 | 0.07616584 | 0.651690357 |
| FBLN1 | 0.076184797 | 1.4031121 |
| HSD17B14 | 0.076204059 | 0.638626587 |
| ZMAT3 | 0.076263066 | 0.850514012 |
| FAM21FP | 0.136208507 | 1.142906102 |
| MCM3 | 0.076299984 | 1.114399344 |
| KLRG2 | 0.076304862 | 0.649991591 |
| OR5B21 | 0.07634778 | 3.158297395 |
| KIAA1211 | 0.07637957 | 1.589210241 |
| FIGLA | 0.076390713 | 2.393390863 |
| PTGS2 | 0.076437343 | 2.122513544 |
| CCDC82 | 0.076442073 | 1.223951217 |
| PTPRS | 0.076522052 | 0.832676561 |
| GUCY2F | 0.07661876 | 2.069127515 |
| TESK2 | 0.076754803 | 0.821515768 |
| GTF2H2C | 0.07682146 | 1.174061802 |
| RP11-33B1.1 | 0.071039209 | 1.141506458 |
| ARL6IP1 | 0.076886073 | 1.120728179 |
| SERPINB9 | 0.076917736 | 1.274524326 |
| TTC34 | 0.076947293 | 1.403852766 |
| NUP107 | 0.07696789 | 1.131750847 |
| TNS1 | 0.076982111 | 1.256070429 |
| GNB1L | 0.076992737 | 0.839257128 |
| FTL | 0.077038273 | 0.805513182 |
| ZNF85 | 0.077044728 | 1.470408852 |
| KRT6B | 0.077097734 | 0.523344025 |
| NKAIN1 | 0.077177049 | 0.590628595 |
| KIAA0101 | 0.077184489 | 1.133555036 |
| MMP23B | 0.077202192 | 0.664651724 |
| PYROXD1 | 0.077391082 | 1.125877002 |
| CATSPER4 | 0.077394512 | 1.405845661 |
| GPR37L1 | 0.077483461 | 1.65082063 |
| PARP8 | 0.077524643 | 1.198218021 |
| BCAS1 | 0.077564097 | 1.640652346 |
| ZNF792 | 0.077590941 | 1.216065286 |
| LZIC | 0.077634558 | 0.892699619 |
| LIX1 | 0.077644798 | 11.89708451 |
| PGBD3 | 0.077681542 | 1.327682507 |
| C8orf74 | 0.077700138 | 1.625792637 |
| CFAP44 | 0.077709966 | 1.251832459 |
| DMRTC2 | 0.07778464 | 5.174269348 |
| EGR1 | 0.077797002 | 1.346253876 |
| RBSN | 0.077848129 | 1.111739529 |
| SERTAD3 | 0.077901955 | 1.143119096 |
| FLOT2 | 0.077931764 | 1.111808225 |
| LIM2 | 0.077991796 | 0.245375736 |
| AP1S3 | 0.078006748 | 1.234927465 |
| DFNA5 | 0.078007343 | 1.419280763 |
| FGFR3 | 0.078050785 | 0.614429958 |
| SCAMP1-AS1 | 0.089042956 | 1.138085781 |
| IQCF2 | 0.078208117 | 7.439054953 |
| MAD2L2 | 0.078218689 | 1.154876103 |
| RNLS | 0.078283534 | 1.157586315 |
| STAB2 | 0.078286716 | 1.31773509 |
| KDSR | 0.078323704 | 0.88347879 |
| ATAD1 | 0.078341725 | 1.101249989 |
| COL6A5 | 0.078351411 | 1.809237127 |
| CALB2 | 0.07835378 | 0.381840355 |
| CAPS2 | 0.078386052 | 1.237540412 |
| EIF4A3 | 0.078408595 | 1.091027752 |
| ATP6V1A | 0.078643472 | 0.888257234 |
| TNFSF15 | 0.078649244 | 1.336371374 |
| PLIN5 | 0.078664773 | 1.692606945 |
| SLC7A5 | 0.078679165 | 1.715276281 |
| GSTM1 | 0.078749152 | 0.439523532 |
| HAMP | 0.078801344 | 0.705472392 |
| IL19 | 0.078810369 | 4.210100025 |
| EIF3J | 0.078831899 | 0.898202535 |
| FAM208A | 0.078848187 | 1.117600458 |
| LINC00997 | 0.151157188 | 1.136182532 |
| KIF15 | 0.078863362 | 1.175057786 |
| PPP1R11 | 0.078961355 | 1.101157478 |
| SMAD9 | 0.078970267 | 1.915606621 |
| RP11-120D5.1 | 0.144503147 | 1.13581571 |
| GEM | 0.079012226 | 1.62447848 |
| MED17 | 0.079195584 | 1.111447805 |
| ECSIT | 0.079214932 | 0.837487387 |
| BCKDHA | 0.079244026 | 0.865242849 |
| MED14OS | 0.092578753 | 1.135384718 |
| BCAS3 | 0.079258349 | 0.853795923 |
| TMEM102 | 0.079432855 | 0.894177196 |
| APOE | 0.079479029 | 0.684482457 |
| AURKC | 0.07948728 | 0.814735605 |
| SEC63P1 | 0.116415444 | 1.134995045 |
| RASGRF1 | 0.079570826 | 1.746634336 |
| TMEM88B | 0.07960946 | 0.528889506 |
| TSPAN13 | 0.079637782 | 1.252924358 |
| NCF2 | 0.079650068 | 0.803695826 |
| RP11-423P10.2 | 0.117517347 | 1.13426449 |
| NR1D2 | 0.079704614 | 1.172174769 |
| FEM1A | 0.07971033 | 0.85419061 |
| C14orf159 | 0.079796778 | 1.164332643 |
| C20orf85 | 0.079805091 | 11.45811143 |
| UNC5CL | 0.079826379 | 1.428290817 |
| PRMT6 | 0.079855302 | 1.150150382 |
| C6orf118 | 0.079880385 | 3.161976808 |
| DUSP1 | 0.079928596 | 1.339564263 |
| SYTL4 | 0.08002822 | 1.215341938 |
| HMGN1P37 | 0.122194699 | 1.133545041 |
| SREBF2 | 0.080146364 | 0.88410527 |
| RIN2 | 0.080192327 | 0.865896347 |
| STK39 | 0.080215148 | 1.185807552 |
| GTF2A1L | 0.08027785 | 1.927674027 |
| PCDHGA3 | 0.080311564 | 1.425301476 |
| ZNF674-AS1 | 0.124188497 | 1.132850628 |
| OGFRL1 | 0.080330551 | 0.821784331 |
| CKMT2-AS1 | 0.068026275 | 1.132788309 |
| GIPC1 | 0.080386236 | 0.886830625 |
| SETDB2 | 0.080484577 | 1.141269509 |
| ZFAND6 | 0.080495971 | 1.085236345 |
| CDK20 | 0.080509959 | 1.214481142 |
| C1R | 0.08054306 | 1.264431253 |
| C5orf22 | 0.080544468 | 1.143176216 |
| PSMA7 | 0.080562931 | 0.875535894 |
| MATN2 | 0.080637297 | 1.249441369 |
| WDR62 | 0.080728538 | 0.829725265 |
| TRIM26 | 0.080734009 | 1.106764975 |
| C2CD3 | 0.080876224 | 1.120785318 |
| ALPL | 0.080895429 | 2.1886733 |
| TNFRSF25 | 0.080922057 | 0.818208355 |
| TOMM40L | 0.080939568 | 0.877242945 |
| MORN2 | 0.080947161 | 1.1992577 |
| MAN2A1 | 0.081007491 | 1.146442663 |
| PRDX4 | 0.081013797 | 1.123227506 |
| PAK4 | 0.0810787 | 0.830305488 |
| DLX3 | 0.081081905 | 1.535781049 |
| MYEOV | 0.081114686 | 0.564273022 |
| C3orf20 | 0.081272678 | 1.348842979 |
| RP11-87H9.2 | 0.11748317 | 1.131041712 |
| SLC2A13 | 0.081284642 | 1.294275742 |
| POLR3D | 0.081296484 | 0.896531682 |
| SH3GLB2 | 0.081327106 | 1.124594119 |
| FLRT2 | 0.081406262 | 0.601521883 |
| KAT2B | 0.081433207 | 1.180654836 |
| FAM83G | 0.081438066 | 0.765974593 |
| CDH13 | 0.081518255 | 1.556517604 |
| UBE2C | 0.081528995 | 0.869667314 |
| FKSG48 | 0.081561003 | 0.611400897 |
| SWAP70 | 0.08158119 | 1.131547862 |
| DCXR | 0.08165674 | 0.824287612 |
| ZNF704 | 0.081679394 | 1.393240427 |
| C9orf57 | 0.081694973 | 0.366729579 |
| SLC9B2 | 0.081729636 | 0.746542973 |
| ZNF197 | 0.081742401 | 1.122133674 |
| FAM86EP | 0.128784676 | 1.129621235 |
| C6orf163 | 0.081756905 | 1.2474448 |
| E2F5 | 0.081759841 | 1.257920686 |
| ACAN | 0.08183396 | 1.555633931 |
| CXCL16 | 0.081842453 | 1.162527018 |
| HGFAC | 0.081846409 | 1.51065235 |
| IMPAD1 | 0.081870641 | 1.110186668 |
| POLRMT | 0.081927914 | 0.880599014 |
| OTOP2 | 0.081948375 | 0.396584909 |
| GABBR2 | 0.081951006 | 4.874112918 |
| CDC42BPG | 0.081959928 | 0.877431517 |
| FOS | 0.081967473 | 1.384864426 |
| GLT6D1 | 0.082009563 | 0.291353367 |
| COPB1 | 0.08201826 | 1.099951799 |
| ZNF582 | 0.08208018 | 1.359048216 |
| TMA7 | 0.082088274 | 1.147432298 |
| FUCA2 | 0.082120309 | 1.143780085 |
| RP11-1148L6.9 | 0.124902248 | 1.128720035 |
| ABHD6 | 0.082211405 | 1.211797457 |
| VWA5B2 | 0.082231765 | 2.38072582 |
| PLEKHH3 | 0.082239484 | 0.877214444 |
| HZGJ | 0.082336837 | 0.692560653 |
| C6orf57 | 0.08234077 | 1.139888185 |
| METTL6 | 0.082361708 | 1.097693363 |
| TRAPPC3L | 0.082389009 | 1.500907466 |
| NNMT | 0.082394145 | 1.503264613 |
| SMIM18 | 0.082406206 | 1.346242637 |
| SPTBN5 | 0.082416151 | 0.700105791 |
| DCTD | 0.082445286 | 1.094634143 |
| CLPB | 0.082456138 | 0.868604083 |
| LIMD2 | 0.082491211 | 0.820211592 |
| NUAK1 | 0.082504836 | 1.354999822 |
| RP11-196G11.5 | 0.140980205 | 1.126543899 |
| JAK2 | 0.082552722 | 0.550279419 |
| SEC31B | 0.082557047 | 1.259751811 |
| CIDECP | 0.057960005 | 1.126337023 |
| LTK | 0.082687504 | 1.70658362 |
| DCDC2 | 0.082780322 | 2.093466048 |
| SHH | 0.082907287 | 6.045531417 |
| ECD | 0.082911629 | 1.086467888 |
| MYOM2 | 0.082956204 | 0.639036436 |
| SEMA4B | 0.082962174 | 0.795049118 |
| SPO11 | 0.082964234 | 0.58866993 |
| ZNF735 | 0.082984446 | 3.84086066 |
| PSMG2 | 0.083018376 | 1.089023234 |
| FLOT1 | 0.083146503 | 1.138602224 |
| LARP1B | 0.08316258 | 1.144525608 |
| CCDC137 | 0.083291872 | 0.900926327 |
| TIMM21 | 0.083343219 | 0.907597999 |
| DHRS4 | 0.083352703 | 0.87750257 |
| SPINK5 | 0.083434252 | 0.516095129 |
| EMC3 | 0.083451727 | 1.100192691 |
| SS18L2 | 0.083580765 | 1.124727745 |
| OPTC | 0.083652534 | 5.558270804 |
| LSS | 0.083724671 | 0.868635856 |
| LAMTOR5-AS1 | 0.115256279 | 1.124866561 |
| DDX39B | 0.08372521 | 1.162662016 |
| CCDC177 | 0.083735664 | 0.66072156 |
| ATF6B | 0.083747179 | 1.120666829 |
| ZNF354A | 0.083767697 | 1.114631637 |
| FARSB | 0.083784915 | 0.86433526 |
| IGFBP5 | 0.083797876 | 1.756197755 |
| SFXN2 | 0.083813249 | 1.197323635 |
| TMEM191B | 0.083822295 | 0.630332124 |
| KBTBD11 | 0.08383432 | 1.576784284 |
| CCDC148 | 0.0838812 | 1.349747775 |
| TLCD1 | 0.083899534 | 0.804580222 |
| ADAMTS15 | 0.083943204 | 1.530603438 |
| ELOVL7 | 0.083947793 | 1.186549868 |
| CKM | 0.083962787 | 4.303906279 |
| F12 | 0.084000557 | 0.786948263 |
| BLM | 0.084087104 | 1.12426202 |
| CA5A | 0.084139081 | 1.392726495 |
| MED19 | 0.084143968 | 0.899609368 |
| ZNF654 | 0.084149726 | 1.118576454 |
| HPCAL1 | 0.084198329 | 1.130687295 |
| TMEM237 | 0.084207601 | 1.148971392 |
| RASGRP2 | 0.084225797 | 0.772001129 |
| ODAM | 0.084247757 | 7.074113193 |
| IKZF4 | 0.084273864 | 1.145808968 |
| INHBB | 0.084289803 | 1.947772061 |
| TTLL2 | 0.084329268 | 4.885337667 |
| CHCHD10 | 0.084346687 | 0.779449372 |
| PI3 | 0.084452556 | 0.476558199 |
| B4GALNT1 | 0.084467289 | 0.596629625 |
| RABL2B | 0.084475946 | 1.142730104 |
| DSP | 0.084487222 | 0.790793438 |
| UBA6-AS1 | 0.021391735 | 1.122139092 |
| PAN2 | 0.084510744 | 1.125554135 |
| PTK2 | 0.084564295 | 1.120151723 |
| MST1 | 0.08457924 | 1.235606985 |
| LINC01420 | 0.141837978 | 1.122018696 |
| SGCE | 0.084581634 | 1.287914542 |
| EPS15L1 | 0.08467462 | 0.911827481 |
| KIF3A | 0.084694268 | 1.147638328 |
| PTK7 | 0.084712916 | 1.169622265 |
| TTC28 | 0.084716587 | 1.203215507 |
| PIR | 0.084749786 | 0.762779992 |
| GATA2 | 0.084751037 | 1.580410323 |
| PODXL2 | 0.084755245 | 0.830480351 |
| TBC1D29 | 0.084800527 | 1.428082212 |
| TRABD | 0.084830263 | 0.888678243 |
| UMAD1 | 0.022505575 | 1.121133073 |
| PDZD2 | 0.084854443 | 1.363280126 |
| TOMM22 | 0.084905054 | 0.90545715 |
| MAGED4B | 0.084945205 | 1.903971246 |
| CES1 | 0.084954359 | 0.084129883 |
| DSTN | 0.085014611 | 0.880450427 |
| pk | 0.085046779 | 1.165553057 |
| TOP1MT | 0.085113166 | 0.816055652 |
| TBCC | 0.085119254 | 1.121946932 |
| IBA57 | 0.085125972 | 0.788847233 |
| ZXDB | 0.085157873 | 1.136838955 |
| RRP7A | 0.085288459 | 0.797863321 |
| HMGB1P5 | 0.149927473 | 1.120430617 |
| ECH1 | 0.08529455 | 0.841645845 |
| PEAK1 | 0.0853714 | 1.165482495 |
| DYNLL1-AS1 | 0.131406995 | 1.120099742 |
| SLC39A2 | 0.085385085 | 0.660991559 |
| CS | 0.085448218 | 0.918151777 |
| MTUS2 | 0.085476021 | 2.631995057 |
| MICU3 | 0.085490421 | 2.091393159 |
| CCDC127 | 0.085616074 | 1.166841908 |
| SMARCD2 | 0.085616415 | 0.899908304 |
| PDE6C | 0.085652946 | 1.25675973 |
| MCHR2 | 0.085652961 | 0.271889018 |
| COL8A2 | 0.085750503 | 0.772219601 |
| TFPI2 | 0.085767819 | 2.289962967 |
| CIRH1A | 0.085777406 | 0.893218704 |
| GP6 | 0.085789051 | 3.7091142 |
| RBM42 | 0.085871825 | 0.857103679 |
| GATA3 | 0.085924262 | 1.427761393 |
| CASZ1 | 0.085971343 | 0.800631598 |
| CNTFR | 0.085973717 | 0.529729444 |
| OTUD6B-AS1 | 0.082153613 | 1.118318872 |
| MICA | 0.08604427 | 1.156579628 |
| SCUBE2 | 0.086058381 | 1.906944066 |
| NAT6 | 0.086064163 | 1.111630267 |
| HS3ST2 | 0.086105993 | 0.558534353 |
| TMEM170A | 0.086113664 | 1.124088423 |
| BTBD18 | 0.086115676 | 0.771993597 |
| PPP3CB-AS1 | 0.139558693 | 1.117696406 |
| PAIP2 | 0.086139927 | 1.087135878 |
| MLLT1 | 0.08617404 | 0.901667827 |
| GRAMD1A | 0.086231229 | 1.192098632 |
| BRCA2 | 0.086285426 | 1.158648761 |
| RBM22 | 0.086308675 | 1.074644911 |
| RBM27 | 0.086334264 | 1.072989596 |
| DDN | 0.086369323 | 0.485623329 |
| ANO8 | 0.086403503 | 1.211622228 |
| VSIG10 | 0.086408989 | 1.167748935 |
| SLC5A10 | 0.086410687 | 0.675154967 |
| SLC1A1 | 0.086410749 | 1.357145216 |
| ASB11 | 0.086414206 | 1.866180213 |
| LYZL6 | 0.086475839 | 2.821544138 |
| ZNRD1 | 0.08648965 | 1.13942869 |
| UBE2Q2 | 0.086506434 | 1.111433786 |
| IL6ST | 0.086507192 | 1.138607364 |
| CD9 | 0.086507356 | 0.792152532 |
| DPY30 | 0.086527723 | 1.103625239 |
| FAM109A | 0.08660273 | 1.119979467 |
| LOXL4 | 0.08661582 | 1.558320474 |
| THPO | 0.086653264 | 2.580585049 |
| FBF1 | 0.086664693 | 0.820334186 |
| MPG | 0.086694752 | 1.115864681 |
| KIF6 | 0.086722659 | 1.92475672 |
| TMEM233 | 0.086775539 | 4.263765919 |
| ANKRD2 | 0.086812937 | 1.672213779 |
| FKSG52 | 0.086894982 | 0.674877181 |
| ZNF628 | 0.086943168 | 0.896293455 |
| ZNF84 | 0.086964782 | 1.133299489 |
| RP11-196G11.6 | 0.132057983 | 1.114545901 |
| TNFRSF12A | 0.087000805 | 1.254539842 |
| GOT2 | 0.087008042 | 0.902773993 |
| MSANTD2 | 0.087014867 | 1.169534114 |
| RSPH14 | 0.087021143 | 1.495166175 |
| LYPD4 | 0.087032917 | 1.579182661 |
| YEATS2 | 0.087314366 | 0.807403888 |
| MICALL1 | 0.087380319 | 0.849202752 |
| MOCS2 | 0.087402363 | 1.120977294 |
| UBE2V1 | 0.087416262 | 0.892333173 |
| PSMD11 | 0.08745829 | 0.889335791 |
| C16orf93 | 0.087481863 | 1.25665687 |
| PROB1 | 0.087541281 | 1.200100367 |
| PNMAL2 | 0.08763793 | 0.676463559 |
| PLEKHA6 | 0.08770274 | 1.364048315 |
| WRNIP1 | 0.087703552 | 1.099256706 |
| USHBP1 | 0.087706911 | 1.363255964 |
| ZCWPW2 | 0.087731691 | 1.177754799 |
| GRP | 0.087744256 | 0.629037866 |
| PCDH12 | 0.087786292 | 1.271342307 |
| LNP1 | 0.087871675 | 0.822919055 |
| DDX47 | 0.087900968 | 1.220270317 |
| ITM2B | 0.087912063 | 1.13807127 |
| TNFRSF17 | 0.087938104 | 0.601119057 |
| LRRTM4 | 0.087975474 | 0.219483096 |
| C5AR2 | 0.088057125 | 1.504998993 |
| IRGC | 0.08806501 | 1.59614681 |
| MINA | 0.088131866 | 0.880924772 |
| FAM3C | 0.088151503 | 1.176228669 |
| CDCA4 | 0.088184949 | 0.88292351 |
| LINC01184 | 0.127085019 | 1.111890664 |
| PRDM5 | 0.088205549 | 1.326141992 |
| PPHLN1 | 0.088230425 | 1.072010194 |
| ABCB6 | 0.088291199 | 0.847484035 |
| MEAF6 | 0.088306865 | 1.094687676 |
| DUSP28 | 0.088401922 | 0.863630356 |
| CSPG5 | 0.088423255 | 1.497086591 |
| DAZAP1 | 0.088452908 | 0.932325032 |
| CCDC51 | 0.088491204 | 1.122392005 |
| PRR15 | 0.088494577 | 1.610910655 |
| TMEM139 | 0.088503709 | 1.593943591 |
| TPBG | 0.088558939 | 1.165199474 |
| TMEM99 | 0.088570508 | 0.84108797 |
| RP11-342K6.1 | 0.044845083 | 1.110920441 |
| NUP35 | 0.088612052 | 0.892704358 |
| RGS7 | 0.088657026 | 3.106743316 |
| LDHAL6A | 0.088665489 | 2.83080614 |
| WDR25 | 0.088792406 | 1.121947314 |
| KCNH5 | 0.088892011 | 9.616332344 |
| CAMK2N1 | 0.089003675 | 1.420829425 |
| GLT1D1 | 0.089004583 | 1.830147381 |
| TINF2 | 0.08900705 | 0.90440957 |
| MAPK15 | 0.089074183 | 1.694449524 |
| EXOC6 | 0.089109105 | 1.160006957 |
| COLCA2 | 0.089134781 | 1.40446691 |
| FAM102B | 0.089141412 | 1.212914774 |
| BEND3 | 0.08917732 | 1.170773932 |
| FANCF | 0.089317576 | 1.176210754 |
| VPS16 | 0.089337068 | 0.886571727 |
| NR0B2 | 0.089356452 | 3.138130719 |
| NXPE2 | 0.08940003 | 1.7449962 |
| NCMAP | 0.089400304 | 1.818653656 |
| ZC3H15 | 0.089416991 | 0.878982256 |
| MYCN | 0.089485491 | 2.577134333 |
| CDC20B | 0.089491444 | 4.960160082 |
| EPHB1 | 0.089589165 | 0.13232838 |
| IFT22 | 0.089608364 | 1.180212523 |
| CLCN1 | 0.089620789 | 0.551870911 |
| PDGFA | 0.089624175 | 1.248541249 |
| NAA25 | 0.089637537 | 0.906277912 |
| HBE1 | 0.089646294 | 4.72226551 |
| LEMD1 | 0.089745008 | 1.440211865 |
| TCEAL1 | 0.089750198 | 1.154719812 |
| ZNF354B | 0.089800544 | 1.129885459 |
| NEUROD6 | 0.089938894 | 0.199049249 |
| SLAIN1 | 0.089941737 | 1.5294484 |
| ZNF746 | 0.089982071 | 1.110565085 |
| AIM1 | 0.090121628 | 0.816402178 |
| CDH20 | 0.090215583 | 0.543328111 |
| FRS2 | 0.090266939 | 1.129619554 |
| AQP12B | 0.090312669 | 6.808149021 |
| TICRR | 0.09033147 | 0.772295079 |
| SRSF1 | 0.090465825 | 1.067319261 |
| PPP2R5A | 0.090469885 | 0.894641162 |
| MYD88 | 0.090535196 | 1.130433423 |
| RP11-571M6.15 | 0.090552606 | 1.238267079 |
| CYP2F1 | 0.090556128 | 0.280268864 |
| HTRA1 | 0.090561287 | 1.301524753 |
| MMAA | 0.090562562 | 1.13788935 |
| LEPROT | 0.090687346 | 1.103947219 |
| CBLB | 0.090731135 | 1.230046067 |
| PHYH | 0.090786605 | 2.346410638 |
| CENPBD1P1 | 0.106527789 | 1.105723758 |
| ANKUB1 | 0.090791071 | 2.096570961 |
| MOGS | 0.090809421 | 1.082109142 |
| LPL | 0.090817824 | 0.404592398 |
| SNCG | 0.090901222 | 1.786914028 |
| SUZ12P1 | 0.141566512 | 1.105429732 |
| CNP | 0.090998985 | 0.896834846 |
| PLEKHG2 | 0.091113532 | 1.25179598 |
| TRAM1L1 | 0.091126763 | 1.701667665 |
| ECI2 | 0.091200217 | 1.315579292 |
| ZNF608 | 0.091217743 | 1.754583424 |
| EPM2A | 0.091238393 | 1.140466176 |
| CERS5 | 0.091278146 | 1.093181087 |
| AHDC1 | 0.091322824 | 0.830264813 |
| ACSF2 | 0.091370561 | 1.317687578 |
| IGFL1 | 0.09137209 | 2.377637304 |
| RWDD4P2 | 0.143741926 | 1.104203702 |
| LY86 | 0.091392153 | 0.771225614 |
| C18orf21 | 0.091392866 | 0.908455902 |
| EFCAB6 | 0.091411305 | 1.345082646 |
| TRIP6 | 0.091441112 | 0.871811959 |
| ACVR2B | 0.091442172 | 1.185183166 |
| HEATR1 | 0.091472719 | 0.860619149 |
| PAX6 | 0.091498308 | 1.274472572 |
| MZT2A | 0.091527492 | 0.785578696 |
| PPP1R3G | 0.091662387 | 0.658867987 |
| DTWD1 | 0.09166585 | 1.122811652 |
| C1QTNF9 | 0.091732177 | 1.508721526 |
| YES1 | 0.091814529 | 1.12943319 |
| TPRG1 | 0.091941066 | 0.644010873 |
| GLI2 | 0.09196218 | 0.59847335 |
| CAPNS2 | 0.091981618 | 0.679391822 |
| CA5BP1 | 0.140589323 | 1.101866072 |
| RPP25 | 0.09201849 | 0.854061021 |
| CLEC4F | 0.092035321 | 1.546847412 |
| SOCS5 | 0.092091152 | 1.107379742 |
| TMEM25 | 0.092110791 | 1.151551661 |
| ATP7B | 0.092131043 | 1.813705691 |
| ZNF468 | 0.092225227 | 1.210470201 |
| GALE | 0.092267762 | 1.156598146 |
| PLCD4 | 0.092291025 | 1.21680247 |
| WNK1 | 0.09229402 | 0.791420282 |
| SULT1A2 | 0.092307345 | 1.558087643 |
| KLHL34 | 0.09237276 | 3.227247312 |
| CAPN5 | 0.092391695 | 1.565354564 |
| TMEM144 | 0.092414757 | 1.190235286 |
| HES3 | 0.092424608 | 0.403687698 |
| OR6B3 | 0.092446311 | 25.08265338 |
| OBSL1 | 0.092478172 | 1.410771939 |
| SCG2 | 0.092501277 | 2.084785612 |
| RYR3 | 0.092522599 | 0.230981082 |
| HCN2 | 0.092560561 | 0.549058951 |
| NLRP13 | 0.092666907 | 2.330540417 |
| TBXA2R | 0.09270016 | 1.322812596 |
| NUTM2B-AS1 | 0.079993534 | 1.099785896 |
| PES1 | 0.092717292 | 0.877157608 |
| RP11-65B7.2 | 0.092918221 | 1.32385576 |
| NXPE3 | 0.092958976 | 0.793253474 |
| FUT6 | 0.092961489 | 1.520428078 |
| RNF168 | 0.093024502 | 0.798962758 |
| TSGA10IP | 0.093056597 | 2.229586725 |
| DNAJC14 | 0.09320828 | 1.119804314 |
| TRDMT1 | 0.093261213 | 1.206135292 |
| PP2D1 | 0.093263815 | 1.179359134 |
| RHCE | 0.09330525 | 0.763614012 |
| USP19 | 0.09330743 | 1.107066009 |
| NGLY1 | 0.093307483 | 1.115103791 |
| PPL | 0.093375714 | 0.788822789 |
| NME2 | 0.093409078 | 0.760738908 |
| IDUA | 0.093421594 | 1.196272911 |
| CSMD1 | 0.09347599 | 2.771976202 |
| PTPRJ | 0.093564489 | 1.248500855 |
| NPC1 | 0.09358566 | 0.878548408 |
| TMED6 | 0.093609929 | 1.553142944 |
| NOP56 | 0.093669871 | 0.862514575 |
| TMEM252 | 0.093688814 | 12.46889377 |
| SLC12A4 | 0.093707404 | 1.157639492 |
| ZBTB18 | 0.093797271 | 1.186475736 |
| ATP11C | 0.09401837 | 1.192976916 |
| FETUB | 0.094029483 | 0.143202622 |
| C2orf54 | 0.09409968 | 0.685801383 |
| NBPF1 | 0.09410687 | 1.183757401 |
| HOXA7 | 0.094191782 | 0.736444213 |
| MRPL1 | 0.094230193 | 1.120859591 |
| CTC-454I21.3 | 0.094293741 | 1.369327392 |
| AXIN2 | 0.094329941 | 3.468971899 |
| FAM174A | 0.094405705 | 1.132697975 |
| FBXL17 | 0.094466123 | 1.121366426 |
| DGAT2 | 0.094475234 | 0.721977425 |
| NR0B1 | 0.094486733 | 12.88791518 |
| AK8 | 0.09462951 | 1.373697044 |
| C1orf186 | 0.094636416 | 1.631761069 |
| PDS5B | 0.094655278 | 1.106271502 |
| NDUFB5 | 0.094694458 | 0.86606425 |
| WNT11 | 0.094707748 | 0.324641064 |
| AMDHD2 | 0.094710438 | 0.86859818 |
| GTF3C1 | 0.094720101 | 0.898423973 |
| COPG1 | 0.094721506 | 1.089595636 |
| MFAP3L | 0.094772238 | 1.519392318 |
| CHN1 | 0.094795852 | 1.297866028 |
| DFNB31 | 0.09485407 | 1.20974518 |
| BMP2 | 0.094904776 | 1.372152642 |
| POLR3G | 0.094917172 | 0.74096908 |
| AC008810.1 | 0.094945056 | 0.649528457 |
| DEFB4B | 0.095027283 | 0.174523011 |
| STX11 | 0.095060388 | 0.748116147 |
| VAPA | 0.095114543 | 1.082703024 |
| AC092159.1 | 0.095155087 | 2.578263092 |
| COMMD3 | 0.0951647 | 1.102617844 |
| ZCCHC13 | 0.095170078 | 3.710418097 |
| TANC2 | 0.095185291 | 1.150566576 |
| SEC14L1 | 0.095236804 | 1.181664497 |
| TLE2 | 0.095273451 | 1.315115816 |
| IQGAP2 | 0.095383421 | 1.39892394 |
| CCDC125 | 0.095416316 | 1.140350527 |
| APC2 | 0.095448806 | 0.67724308 |
| USH1G | 0.095472951 | 0.665470208 |
| H3F3A | 0.095534271 | 1.102679015 |
| LRRC61 | 0.095549003 | 1.174429933 |
| PLA2G4A | 0.09569092 | 1.545692948 |
| PABPC3 | 0.095730953 | 0.804405871 |
| CD1E | 0.095758704 | 0.660643808 |
| CDCP1 | 0.095821318 | 1.180817321 |
| HLCS | 0.095822785 | 1.15629488 |
| BAI1 | 0.09587228 | 0.374376119 |
| GNA15 | 0.095894975 | 0.81760186 |
| TTPA | 0.095929001 | 2.325688119 |
| PDGFC | 0.095932111 | 1.351272842 |
| LYG2 | 0.095944442 | 1.962547494 |
| AC009022.1 | 0.095969736 | 0.491077097 |
| AKAP5 | 0.096048818 | 1.288153593 |
| CLDN11 | 0.096073634 | 4.436763796 |
| AKR1B15 | 0.096077076 | 0.659044218 |
| AP000721.4 | 0.096125746 | 0.576400803 |
| CD59 | 0.096189434 | 1.174989372 |
| OR52B2 | 0.096200517 | 2.917392641 |
| CHSY1 | 0.096211129 | 1.148560967 |
| SLC39A10 | 0.096217653 | 1.214827319 |
| CDH18 | 0.096260067 | 8.335782934 |
| NT5DC3 | 0.096318397 | 1.255213586 |
| CDHR3 | 0.096363902 | 2.153528408 |
| RUSC1 | 0.096451128 | 0.90879122 |
| PDE4DIP | 0.096512896 | 1.168247634 |
| DNAJA1 | 0.096518694 | 1.126600477 |
| IQCF6 | 0.096625527 | 2.488882689 |
| COQ10B | 0.09663642 | 0.90658213 |
| NAPG | 0.096693474 | 1.096666389 |
| BARX2 | 0.096706331 | 0.639467726 |
| PLOD3 | 0.096733937 | 1.203347944 |
| ABCG5 | 0.09673722 | 1.314135896 |
| C1QTNF1 | 0.096761933 | 1.981917865 |
| BCAS4 | 0.096798385 | 0.74059024 |
| MRPL22 | 0.096833215 | 1.103965818 |
| HDAC11 | 0.096833438 | 1.151446395 |
| DNAJC18 | 0.096834807 | 1.15737949 |
| DCTPP1 | 0.096906085 | 0.82187586 |
| BET1 | 0.096928976 | 1.082098315 |
| TSPAN7 | 0.096929549 | 0.56518773 |
| POLR2E | 0.097010212 | 0.894935283 |
| USP35 | 0.097021633 | 0.733903567 |
| STK11IP | 0.0971081 | 0.88372974 |
| ANKRD34B | 0.097125026 | 1.576535467 |
| PRDX3 | 0.097277321 | 1.135996986 |
| NR2F2 | 0.097362022 | 1.288708415 |
| LRFN3 | 0.097394208 | 0.838670823 |
| CDHR4 | 0.097413364 | 2.816434385 |
| MSH5 | 0.0974155 | 1.236773515 |
| OR4A47 | 0.097444149 | 4.053764343 |
| KANK2 | 0.097446474 | 1.189463594 |
| MIXL1 | 0.097478421 | 0.56117362 |
| MCCC1 | 0.09748872 | 0.857410724 |
| SGSM3 | 0.097522891 | 1.195305539 |
| TOR1A | 0.097549187 | 1.079354748 |
| TRNP1 | 0.097558644 | 1.536225258 |
| ARIH2OS | 0.097595334 | 1.161492537 |
| MMP28 | 0.097611054 | 0.660223073 |
| CCT3 | 0.097730687 | 0.902250498 |
| SLC51B | 0.097753441 | 4.104735783 |
| MCOLN2 | 0.097788366 | 0.740512099 |
| FGF6 | 0.09779742 | 2.876002355 |
| GATA4 | 0.097928106 | 2.248997549 |
| ABHD17C | 0.09798252 | 1.162379537 |
| SGMS2 | 0.09801397 | 1.245331932 |
| PFKL | 0.098048527 | 0.895794111 |
| UQCR11 | 0.098084274 | 0.605884473 |
| EIF2B5 | 0.098170588 | 0.871301541 |
| NLRP7 | 0.098197259 | 2.736706692 |
| TNNT3 | 0.098304905 | 3.907553042 |
| STK36 | 0.098319036 | 1.237881221 |
| TRIM40 | 0.098331148 | 1.985988512 |
| BNIPL | 0.098336358 | 0.705662957 |
| TCF7 | 0.098338272 | 1.272401707 |
| LAMC1 | 0.098362407 | 1.199419563 |
| AUH | 0.098422584 | 1.118969553 |
| CIITA | 0.098463992 | 1.337401716 |
| CYP51A1 | 0.098522278 | 0.797337925 |
| GSTA3 | 0.098595557 | 3.831328288 |
| PHACTR4 | 0.098596229 | 1.098749984 |
| ODF2 | 0.098613784 | 1.094440993 |
| VSNL1 | 0.098618269 | 0.701971908 |
| S100G | 0.098706885 | 9.556845595 |
| KIAA0825 | 0.098822303 | 1.17145431 |
| ARHGEF28 | 0.098919905 | 1.179010018 |
| HOXB8 | 0.099041642 | 1.499747818 |
| DNAH10 | 0.09917438 | 3.214847703 |
| PCLO | 0.099174996 | 0.790246786 |
| ZBTB49 | 0.099231983 | 1.117808105 |
| SNTG2 | 0.099370534 | 1.975503241 |
| CETN3 | 0.099374083 | 1.11439088 |
| CASP9 | 0.099404561 | 1.118359588 |
| ALOX12 | 0.099438372 | 0.606478392 |
| SNX2 | 0.09946964 | 1.093369287 |
| AK5 | 0.099549742 | 1.912307524 |
| KRT20 | 0.09955333 | 8.361950277 |
| DNAH3 | 0.099575963 | 1.600004594 |
| MYO5C | 0.099588526 | 1.217602487 |
| UGT1A6 | 0.099602059 | 0.460911603 |
| C19orf71 | 0.099624045 | 1.210144613 |
| ENTPD7 | 0.099636053 | 1.236402867 |
| ZNF286A | 0.099653933 | 1.63396814 |
| FAM161A | 0.099788261 | 1.180007706 |
| RBKS | 0.099808279 | 1.147140446 |
| SLC46A2 | 0.099829548 | 3.136393385 |
| RFC1 | 0.099866471 | 1.105368619 |
| FRAT1 | 0.09995411 | 1.165916567 |
| CNGA4 | 0.099971707 | 2.498819152 |
| FAM72A | 0.100137654 | 0.868969992 |
| SLC9C2 | 0.100164625 | 2.450547268 |
| TMTC4 | 0.10019859 | 1.154553061 |
| ENTPD4 | 0.100226785 | 1.11675041 |
| CLCNKA | 0.100241888 | 1.76709719 |
| SLC6A4 | 0.10027359 | 0.443827759 |
| PNPLA6 | 0.100331742 | 0.910385932 |
| KIN | 0.100333168 | 1.082136772 |
| RFC3 | 0.100336492 | 1.161481594 |
| CCT6B | 0.100353954 | 1.188262635 |
| COMTD1 | 0.100379621 | 0.800864175 |
| JAKMIP2 | 0.100382578 | 0.625276335 |
| HK1 | 0.100427079 | 0.885245006 |
| OCRL | 0.10043776 | 1.136368756 |
| GRIPAP1 | 0.100517508 | 1.118266336 |
| SLC15A2 | 0.100766602 | 1.46025445 |
| PDCD2L | 0.100824449 | 0.809035026 |
| KLHL25 | 0.100857012 | 1.128827688 |
| CCDC17 | 0.100861134 | 1.639472062 |
| STARD3NL | 0.100892048 | 1.151566013 |
| RAB27A | 0.10091272 | 1.135046001 |
| TMEM225 | 0.100994122 | 2.887927887 |
| EYS | 0.101010466 | 1.192882485 |
| SLC15A4 | 0.101017494 | 1.080222437 |
| PRIM2 | 0.101050114 | 1.100715241 |
| PPAN | 0.101071829 | 0.768567835 |
| SPAG6 | 0.101108909 | 2.327305064 |
| ZNF692 | 0.101139204 | 0.870117163 |
| ADORA2B | 0.10121172 | 0.851543983 |
| RERGL | 0.101212802 | 2.812801289 |
| GPRC5B | 0.101297323 | 1.498477511 |
| ZNF286A | 0.101307673 | 1.142491049 |
| SLC25A31 | 0.101320887 | 1.579371719 |
| VAMP7 | 0.101382571 | 1.13929262 |
| ALPK1 | 0.101427856 | 1.157306284 |
| CHRNB4 | 0.101450833 | 0.687134923 |
| GPR183 | 0.101456516 | 0.771534441 |
| PHF1 | 0.101491359 | 1.111431968 |
| USP12 | 0.101567169 | 1.108265961 |
| FUOM | 0.101580024 | 0.737249826 |
| DRAM2 | 0.101596786 | 1.080487256 |
| SDCCAG3 | 0.101655564 | 0.90750929 |
| KRT5 | 0.101668965 | 0.781287146 |
| IP6K3 | 0.101796949 | 2.073176862 |
| NALCN | 0.101802122 | 2.456593938 |
| NAA50 | 0.101873982 | 0.891161396 |
| ETNPPL | 0.10196305 | 3.176240973 |
| OSBPL9 | 0.102098425 | 1.098166643 |
| SERPING1 | 0.102168386 | 1.275982371 |
| GHR | 0.102205619 | 0.562022355 |
| GPATCH4 | 0.102243574 | 0.894470396 |
| THOC1 | 0.102248099 | 1.090196342 |
| OR52I2 | 0.102299203 | 4.383027807 |
| SGCA | 0.102356594 | 0.659037121 |
| SLC27A6 | 0.102449449 | 2.532563393 |
| NBPF9 | 0.102466473 | 1.195432584 |
| GPR15 | 0.102481537 | 0.475428074 |
| HID1 | 0.10251338 | 1.466280775 |
| CHD4 | 0.102535934 | 1.090576648 |
| KRTAP10-7 | 0.102536949 | 7.182457346 |
| POM121C | 0.102560031 | 0.910029683 |
| PSMC1 | 0.102575113 | 0.883851906 |
| CCDC67 | 0.102671305 | 2.029991871 |
| FAM45A | 0.102683491 | 1.096056413 |
| PRRX1 | 0.102808541 | 1.463493275 |
| PGM5 | 0.102840241 | 1.833402737 |
| CD28 | 0.102861773 | 0.693667513 |
| HMX3 | 0.102862133 | 10.22462641 |
| SLC38A10 | 0.102981301 | 1.105402377 |
| TMEM161A | 0.102999161 | 0.880778974 |
| EVA1B | 0.10300261 | 0.789361466 |
| ADRA1B | 0.103004774 | 1.95640508 |
| PDGFB | 0.103009274 | 1.300831002 |
| PTCHD4 | 0.103015769 | 2.017974069 |
| AARD | 0.103040737 | 2.388974934 |
| FKSG61 | 0.103078934 | 0.73361972 |
| RBM17 | 0.103151787 | 1.083343286 |
| ARMC5 | 0.103200302 | 0.818049741 |
| CREB3 | 0.103225885 | 1.100867074 |
| CDC42EP1 | 0.103320272 | 0.841226512 |
| PIBF1 | 0.103325474 | 1.122152271 |
| ZSWIM4 | 0.103373959 | 1.160887765 |
| LPCAT2 | 0.103387285 | 1.196040388 |
| UCP2 | 0.103425908 | 1.212140639 |
| AL136419.6 | 0.126238134 | 0.89940732 |
| CHRNA6 | 0.103431993 | 0.546687905 |
| RGS9BP | 0.103435701 | 0.385486907 |
| BTC | 0.103437212 | 1.266524336 |
| SLC35C2 | 0.103503898 | 0.908196384 |
| CYP2C18 | 0.103590456 | 0.607479955 |
| MT3 | 0.103632383 | 2.280212998 |
| TBC1D26 | 0.103712188 | 3.335642289 |
| PLA2G4C | 0.103734438 | 0.63447259 |
| TYR | 0.103736474 | 3.227522619 |
| CENPC | 0.10375288 | 1.118767042 |
| RXRG | 0.10391684 | 8.451966873 |
| PLD5 | 0.103925792 | 0.12000142 |
| KIAA0391 | 0.104033723 | 1.088140192 |
| SH3RF2 | 0.104052404 | 0.79590813 |
| MUC3A | 0.104064372 | 6.765968053 |
| DNAH1 | 0.104083065 | 1.21669784 |
| ADAMTSL2 | 0.104186542 | 1.809844686 |
| GET4 | 0.104231288 | 0.898753977 |
| RPGRIP1 | 0.104364266 | 0.840749989 |
| GMDS | 0.1045273 | 1.360370198 |
| TBC1D5 | 0.104559622 | 1.132203738 |
| LINC00657 | 0.088096027 | 0.894670846 |
| ATP2A3 | 0.104718918 | 1.679449407 |
| ABCA13 | 0.104732304 | 0.654541329 |
| MBNL3 | 0.104770225 | 1.175984804 |
| ERP44 | 0.104796338 | 1.075394026 |
| MUC21 | 0.104796887 | 1.824760876 |
| ZNF284 | 0.104886711 | 1.201830368 |
| PTMA | 0.104977794 | 1.101263389 |
| PAGE1 | 0.104984652 | 33.90356175 |
| RNF123 | 0.105082804 | 1.134765266 |
| ITPR1 | 0.105116447 | 0.765949331 |
| M1AP | 0.105169509 | 1.281223089 |
| MFN1 | 0.105178231 | 0.843358329 |
| ST5 | 0.105230981 | 1.162712784 |
| NT5E | 0.1052472 | 1.461821899 |
| RANBP6 | 0.105270709 | 0.612407025 |
| SLC7A4 | 0.105288341 | 1.499351415 |
| CKMT2 | 0.105317622 | 1.971065808 |
| DCTN3 | 0.105329095 | 0.903815881 |
| MAPK11 | 0.105409876 | 0.827643167 |
| RHOF | 0.105509542 | 1.430955139 |
| MYO6 | 0.105532882 | 1.206776315 |
| IFNE | 0.105561306 | 1.407496365 |
| VGLL2 | 0.105585657 | 2.992776533 |
| RPL22 | 0.105605331 | 0.902304399 |
| KIF26B | 0.105671361 | 1.332162494 |
| ZBTB26 | 0.105674612 | 1.121973827 |
| MSR1 | 0.105697194 | 0.729312202 |
| PORCN | 0.105697578 | 1.215004646 |
| MRAS | 0.105906143 | 1.280067567 |
| HOXB3 | 0.106012204 | 1.298679405 |
| SARM1 | 0.10605732 | 1.242410129 |
| RFX3 | 0.106079795 | 1.295735093 |
| PHACTR1 | 0.106087232 | 1.390578125 |
| HOXC13 | 0.106148707 | 1.466751681 |
| C10orf82 | 0.106286952 | 5.866886815 |
| OR5P2 | 0.106356614 | 2.609275561 |
| DHX15 | 0.106398843 | 1.096983109 |
| C3 | 0.106550357 | 1.695516073 |
| NFYA | 0.106566337 | 1.11259211 |
| NDUFS7 | 0.106606906 | 0.866398434 |
| HOXB5 | 0.106702934 | 1.432243686 |
| CENPB | 0.106710801 | 0.872425187 |
| ATXN2 | 0.106800309 | 1.087086144 |
| PIGM | 0.106816252 | 1.129901788 |
| DOT1L | 0.10682797 | 0.872608328 |
| FAM60A | 0.106862121 | 1.161137387 |
| TMEM249 | 0.106891909 | 1.297520602 |
| SCLT1 | 0.106899898 | 1.108988981 |
| CARD9 | 0.106914414 | 0.75468785 |
| LRRFIP2 | 0.106921749 | 1.112041796 |
| DDO | 0.10692873 | 1.323925489 |
| PLXNA2 | 0.106952174 | 0.79173251 |
| ZIK1 | 0.106957519 | 1.419619675 |
| ATG12 | 0.106969735 | 1.074841238 |
| CLIC2 | 0.106995663 | 0.707943243 |
| TUBB8 | 0.106997217 | 0.656501582 |
| OAZ3 | 0.107054493 | 1.197866451 |
| LAMTOR5 | 0.107075849 | 1.090334298 |
| ZNF385A | 0.107093728 | 0.845001372 |
| C9orf172 | 0.107142095 | 0.717064684 |
| C16orf70 | 0.107179984 | 0.894272744 |
| NACC2 | 0.107213786 | 1.142098558 |
| BRPF3 | 0.107251825 | 1.137321632 |
| LRP2 | 0.107280192 | 3.729445484 |
| SUSD1 | 0.107297687 | 1.17021534 |
| TRAM1 | 0.107313872 | 1.129120476 |
| BSPH1 | 0.107416907 | 0.162895745 |
| SMOX | 0.107417922 | 1.343137125 |
| EPAS1 | 0.107436665 | 1.23368333 |
| NUDT1 | 0.107444864 | 0.805202499 |
| TICAM1 | 0.107527469 | 0.854630094 |
| RPL23AP82 | 0.086316391 | 0.88383382 |
| WFS1 | 0.107534356 | 1.180255756 |
| RBM4B | 0.107564679 | 1.090144403 |
| C19orf40 | 0.107705121 | 0.875705176 |
| CCER1 | 0.107727428 | 0.296425504 |
| PIAS4 | 0.107757052 | 0.919157302 |
| HTN3 | 0.107798788 | 5.02504507 |
| NCKAP5L | 0.107812179 | 1.187425081 |
| CDKL2 | 0.107823118 | 1.805339013 |
| ISY1-RAB43 | 0.107828057 | 1.285029898 |
| ACTL9 | 0.107850108 | 2.24446348 |
| PCBP4 | 0.107938504 | 1.152039588 |
| ELMO3 | 0.107968627 | 0.848048563 |
| ABCA12 | 0.10808816 | 0.683389885 |
| KBTBD12 | 0.108316758 | 1.414885396 |
| INTS8 | 0.108337558 | 1.095686213 |
| LGALS8 | 0.108378109 | 1.100354333 |
| UBD | 0.108523698 | 1.488753517 |
| SAMD4A | 0.108559125 | 1.268510502 |
| ABCA3 | 0.108595064 | 0.671761416 |
| ADAT2 | 0.108614097 | 1.129576908 |
| RUFY2 | 0.108617986 | 1.0997282 |
| FBXO27 | 0.108640241 | 0.784796445 |
| CUEDC2 | 0.108646151 | 1.115207852 |
| CDCA5 | 0.108717425 | 0.883661715 |
| RFX8 | 0.108743433 | 1.291445166 |
| RHBDL2 | 0.108761592 | 0.819716792 |
| MS4A18 | 0.108830289 | 0.276338211 |
| LAMTOR3 | 0.108831047 | 1.107424879 |
| TRAF5 | 0.108879544 | 1.197148251 |
| LNX1 | 0.108892923 | 1.207678129 |
| PSAT1 | 0.108958286 | 0.807194823 |
| PTER | 0.109145677 | 1.152004828 |
| VLDLR | 0.109156759 | 0.781825099 |
| NPAS3 | 0.109256198 | 3.933812583 |
| PDLIM5 | 0.109261387 | 1.125089423 |
| ITGB3BP | 0.109299791 | 1.109552436 |
| SOGA1 | 0.1093502 | 0.849289334 |
| HDAC10 | 0.109357044 | 0.861773813 |
| SCAMP3 | 0.109385583 | 0.916365536 |
| MROH1 | 0.109416498 | 1.091050818 |
| SPINK6 | 0.109450428 | 4.104155423 |
| KANK4 | 0.109471846 | 2.792313479 |
| PTPRU | 0.109474702 | 1.24143883 |
| OSER1 | 0.109482138 | 0.91048207 |
| RP11-395L14.11 | 0.047126944 | 0.876124023 |
| SLCO2B1 | 0.109538442 | 0.698127205 |
| GATSL3 | 0.109550196 | 0.784354427 |
| CDC37L1 | 0.109665143 | 0.73163095 |
| IL20RB | 0.109665455 | 0.754397759 |
| BTBD16 | 0.10968385 | 5.059146811 |
| CYB561D2 | 0.109740783 | 1.178909809 |
| FAM127A | 0.109806817 | 1.12170376 |
| AFMID | 0.109871185 | 1.128422247 |
| MTNR1B | 0.109908789 | 3.604255973 |
| TJP3 | 0.109940298 | 1.347533639 |
| NPM3 | 0.10995132 | 0.81155955 |
| PLCL2 | 0.109978801 | 1.375315919 |
| MAP3K6 | 0.109981244 | 0.858824244 |
| RASEF | 0.109982431 | 1.205802112 |
| RIOK2 | 0.11000969 | 1.083028047 |
| FSIP2 | 0.110104716 | 1.660087299 |
| MED16 | 0.110105804 | 0.870846321 |
| PROX1 | 0.110125298 | 3.540743855 |
| PPARGC1B | 0.110138955 | 0.794481105 |
| PTCD2 | 0.110143915 | 1.09148712 |
| WDR55 | 0.110184001 | 1.081686124 |
| PDE7B | 0.110197501 | 1.571666621 |
| MMP15 | 0.110256462 | 1.258944912 |
| GOLT1B | 0.110275761 | 1.080600982 |
| KCNK13 | 0.110278525 | 0.366293435 |
| KIAA2012 | 0.110281877 | 2.931769635 |
| SPC25 | 0.110332389 | 0.897799692 |
| AC007292.3 | 0.084827332 | 0.87022023 |
| NIPAL3 | 0.110499933 | 0.902619107 |
| RXFP1 | 0.110513242 | 3.111561052 |
| STX2 | 0.110562678 | 1.168691056 |
| ART4 | 0.11061394 | 4.468356758 |
| KCTD4 | 0.110626932 | 1.498047651 |
| KRTAP9-4 | 0.110663615 | 6.093770966 |
| OR7A17 | 0.110740229 | 3.253499285 |
| IZUMO2 | 0.110776156 | 7.685782765 |
| CHP2 | 0.11079852 | 0.477719459 |
| MYOT | 0.110874318 | 1.350747032 |
| MCU | 0.110907427 | 1.202159801 |
| TNFRSF8 | 0.110912569 | 1.291591069 |
| CLEC16A | 0.110923077 | 1.123340809 |
| RP11-428J1.5 | 0.150404346 | 0.867786589 |
| PPAPDC2 | 0.11097571 | 0.601299051 |
| GBP3 | 0.111028488 | 1.181448845 |
| CRNKL1 | 0.11111062 | 0.904733074 |
| BRPF1 | 0.111155515 | 1.110301199 |
| MOSPD3 | 0.111229079 | 0.906012871 |
| LRRC53 | 0.111306264 | 18.37456112 |
| TIMELESS | 0.111335601 | 1.110031809 |
| DDX42 | 0.111376833 | 1.069268271 |
| GPRIN1 | 0.111386284 | 0.834427898 |
| RALGDS | 0.111398609 | 0.886477803 |
| TMEM97 | 0.111433386 | 1.195165678 |
| TMEM45A | 0.111495814 | 0.563346983 |
| FGF9 | 0.111519276 | 4.049013474 |
| NLRP9 | 0.111540352 | 1.460973598 |
| FBXL14 | 0.111645531 | 1.178198568 |
| C16orf86 | 0.111706844 | 0.789258863 |
| ROS1 | 0.111719339 | 2.232423929 |
| DSEL | 0.111724503 | 1.567425231 |
| CTSD | 0.111760864 | 0.876254523 |
| ZNF442 | 0.11177063 | 1.278153881 |
| AGGF1 | 0.111771346 | 1.080782916 |
| CELF6 | 0.111778254 | 1.447051877 |
| CLDN24 | 0.111796913 | 0.478941982 |
| SMIM20 | 0.111806936 | 1.13114249 |
| GNA14 | 0.111842911 | 1.532692922 |
| CD8B | 0.111977843 | 1.790133643 |
| SLC44A4 | 0.112002152 | 1.698872811 |
| NCBP2-AS2 | 0.096638161 | 0.862425462 |
| RAD18 | 0.112010198 | 1.123351574 |
| FAM71A | 0.112073668 | 1.687077862 |
| APBB3 | 0.112130413 | 1.144852087 |
| S100A7L2 | 0.112175217 | 3.635926936 |
| TRAK2 | 0.112234975 | 1.18055684 |
| TBC1D9 | 0.112267048 | 1.144539789 |
| SLC48A1 | 0.112504899 | 0.891890721 |
| RSPH10B2 | 0.11252368 | 1.621219743 |
| CYP2B6 | 0.112527113 | 2.763716107 |
| TRIM52 | 0.112545044 | 1.155465962 |
| LMOD3 | 0.112566736 | 1.487284202 |
| RNF5 | 0.112592788 | 1.097380047 |
| SPP1 | 0.112630476 | 0.666414236 |
| SERBP1 | 0.112684829 | 0.914571086 |
| PATZ1 | 0.112723559 | 1.169301165 |
| TMEM190 | 0.112768291 | 3.30886427 |
| SAPCD1 | 0.112780448 | 1.227289915 |
| FNDC4 | 0.112833476 | 1.541651125 |
| PITPNM1 | 0.112881017 | 1.133951772 |
| CTNNA2 | 0.112924611 | 12.128293 |
| PRMT5-AS1 | 0.128244264 | 0.857427794 |
| WFDC13 | 0.112947088 | 1.659281746 |
| FAM132A | 0.112953265 | 0.440165615 |
| CTD-2233K9.1 | 0.086888381 | 0.85699957 |
| ZNF836 | 0.112978647 | 1.119505778 |
| NCLN | 0.112983989 | 0.894490673 |
| C2orf72 | 0.113023137 | 1.819838768 |
| RBFA | 0.113041151 | 0.890133416 |
| SKP1 | 0.113042354 | 1.080400638 |
| IL24 | 0.113046627 | 1.535994969 |
| CYLD | 0.113055555 | 0.878770886 |
| RAB38 | 0.11305761 | 0.774745077 |
| GBA2 | 0.113129977 | 1.108469553 |
| RASA4CP | 0.072291729 | 0.85483488 |
| REXO1 | 0.113134765 | 0.89935947 |
| C17orf100 | 0.108122929 | 0.854533748 |
| CHIA | 0.113167077 | 12.83038513 |
| CCDC121 | 0.113201807 | 1.172983348 |
| NR2C2 | 0.113247679 | 1.142819793 |
| SERINC3 | 0.1132647 | 0.917310799 |
| AC007292.6 | 0.139791462 | 0.8541346 |
| PHACTR2 | 0.113272456 | 1.176237889 |
| LEP | 0.113427933 | 2.287431138 |
| ZNF430 | 0.113448759 | 0.843695105 |
| MT1G | 0.113474078 | 1.581850738 |
| AC087651.1 | 0.113479227 | 0.238785724 |
| GEMIN8P4 | 0.139286465 | 0.853031255 |
| RP11-45M22.4 | 0.11353128 | 1.275543251 |
| PRPS1L1 | 0.113532286 | 2.120322349 |
| RP11-676J12.9 | 0.103049325 | 0.85271067 |
| TMEM50B | 0.113546431 | 1.116396225 |
| NRL | 0.11360245 | 0.893137286 |
| MRO | 0.113684699 | 0.740156389 |
| OSR1 | 0.113703607 | 1.335839961 |
| ERLIN1 | 0.113725494 | 1.112198724 |
| C1GALT1 | 0.113778506 | 1.16248045 |
| GM2A | 0.113830505 | 0.835597183 |
| QRFPR | 0.113894832 | 2.255589738 |
| DCAF17 | 0.114013654 | 0.898816317 |
| TRIM16L | 0.114026055 | 0.63741794 |
| PLK4 | 0.114130312 | 1.121779605 |
| RAB24 | 0.114146589 | 0.859544681 |
| ZNF729 | 0.114182078 | 2.688694177 |
| NME7 | 0.114197887 | 1.129622609 |
| PILRB | 0.114205315 | 1.272515684 |
| PMEL | 0.114242576 | 1.23876737 |
| TMPRSS11A | 0.114246204 | 0.610992467 |
| BAIAP3 | 0.114323022 | 1.510430063 |
| CNTNAP3 | 0.114344986 | 0.769532507 |
| WNK2 | 0.114347341 | 0.665328483 |
| PEF1 | 0.114349095 | 0.91780676 |
| RP11-390P2.4 | 0.13048312 | 0.84883794 |
| ATL1 | 0.114352597 | 1.2319845 |
| FAM150A | 0.114409119 | 2.419904614 |
| CTNNBIP1 | 0.114422912 | 0.841853638 |
| FAM92B | 0.114547391 | 2.078839865 |
| C1orf106 | 0.114617815 | 0.836681554 |
| SCGB2A1 | 0.114623861 | 3.370874772 |
| AC068580.6 | 0.145613719 | 0.8473413 |
| RP11-545E17.3 | 0.12621864 | 0.847289548 |
| DPP10 | 0.114674012 | 10.67878085 |
| ZC3H12A | 0.114769715 | 1.244204879 |
| MICU2 | 0.114898266 | 1.106587501 |
| GRIK1 | 0.114936212 | 1.671994478 |
| METTL21A | 0.114953353 | 1.150898127 |
| CAPN1 | 0.114965432 | 0.902207394 |
| AC087762.1 | 0.115034734 | 1.703683532 |
| LBX1 | 0.115067058 | 0.376157272 |
| BOP1 | 0.11511755 | 0.82824656 |
| N4BP2 | 0.115155313 | 1.161034836 |
| BIRC5 | 0.115255349 | 0.859958776 |
| VMP1 | 0.115305012 | 1.131126409 |
| LHX5 | 0.11532333 | 1.633669565 |
| GTF3C3 | 0.115407508 | 0.913536679 |
| GPR160 | 0.11542171 | 1.411279748 |
| SEMA3C | 0.115450171 | 1.278152083 |
| RP4-706A16.3 | 0.052536516 | 0.843124436 |
| MUL1 | 0.115554964 | 0.925482512 |
| ZCCHC4 | 0.115558342 | 1.092639493 |
| HES6 | 0.115563578 | 1.740293797 |
| UGT8 | 0.115617978 | 1.359117322 |
| PCDHGB4 | 0.115662879 | 1.749440909 |
| SPIN3 | 0.11570803 | 1.161918144 |
| TOX4 | 0.115731159 | 0.912205254 |
| MYCBP2 | 0.115737869 | 0.850064627 |
| LARP6 | 0.115742719 | 1.250812004 |
| UBTFL1 | 0.115762332 | 0.169280573 |
| MYH14 | 0.115772628 | 0.816191706 |
| ARHGAP22 | 0.115774845 | 1.257410326 |
| GSN-AS1 | 0.10606084 | 0.840061606 |
| RP11-178C3.2 | 0.130867068 | 0.839985313 |
| ZBTB11-AS1 | 0.056087806 | 0.839842486 |
| ZNF697 | 0.115825303 | 1.207071953 |
| ANXA2 | 0.115830068 | 0.88960901 |
| BCL6 | 0.115850837 | 0.838097302 |
| KLHL3 | 0.115859946 | 1.278595819 |
| AC004967.7 | 0.11255076 | 0.8391789 |
| PKDCC | 0.115866071 | 1.900525802 |
| ATP6V1G3 | 0.11587597 | 2.978666581 |
| MCCC2 | 0.115877615 | 1.117539838 |
| RPL5P23 | 0.101475363 | 0.838162875 |
| HSPA1L | 0.115962215 | 1.133658828 |
| GABRQ | 0.115988909 | 0.707722609 |
| APLN | 0.116024337 | 1.714781633 |
| GPT2 | 0.116057023 | 0.807283254 |
| EML4 | 0.116065212 | 1.126230707 |
| RTN4 | 0.116068381 | 0.902576617 |
| SAA2 | 0.116083496 | 1.749328037 |
| RP3-523K23.2 | 0.134969039 | 0.835308511 |
| ANKRD27 | 0.116128458 | 0.851501116 |
| YEATS2-AS1 | 0.146651977 | 0.834974459 |
| WDR31 | 0.116134379 | 1.188604129 |
| RP11-342M1.3 | 0.090603018 | 0.834654208 |
| C11orf74 | 0.116152481 | 1.150580856 |
| HNRNPA3P12 | 0.084969248 | 0.834224861 |
| DAP3P2 | 0.133786275 | 0.834028059 |
| FAM154B | 0.116262994 | 1.742747242 |
| LMX1B | 0.11629426 | 0.512429396 |
| EIF3D | 0.116334225 | 0.894081037 |
| PTPRZ1 | 0.116401005 | 0.685952156 |
| HNRNPM | 0.116464243 | 0.937179465 |
| SLITRK6 | 0.116481779 | 1.715565009 |
| RIMBP3 | 0.116519982 | 0.675829266 |
| AMFR | 0.11656017 | 0.915161736 |
| STIM1 | 0.116622491 | 0.893150656 |
| CLEC4D | 0.116637713 | 1.647336876 |
| GPR21 | 0.116661737 | 0.531235854 |
| FRMD5 | 0.116692605 | 1.49935741 |
| ERP29P1 | 0.146517779 | 0.831254427 |
| FTLP2 | 0.127984592 | 0.831085295 |
| PDK4 | 0.116782667 | 2.52078132 |
| CTB-25B13.5 | 0.122123429 | 0.830828015 |
| AC139100.4 | 0.143794332 | 0.830809061 |
| LASP1 | 0.116857801 | 1.102635294 |
| FZR1 | 0.116859205 | 0.90214941 |
| PRDM4 | 0.116879576 | 1.08564336 |
| IGSF9 | 0.116887931 | 0.802513458 |
| PSCA | 0.116979017 | 1.669117916 |
| RP11-121M22.1 | 0.134022365 | 0.829545233 |
| RP11-395A13.2 | 0.077723451 | 0.829318115 |
| HMGB2 | 0.117001575 | 1.147350511 |
| FOXE1 | 0.117009412 | 0.645350675 |
| OSMR | 0.117011234 | 1.214474175 |
| FSD2 | 0.117032852 | 1.254666883 |
| RP11-439E19.9 | 0.12483968 | 0.828483415 |
| XIAP | 0.117037944 | 1.122517325 |
| ATP5D | 0.117148185 | 0.698877322 |
| LPCAT1 | 0.117190037 | 1.750420359 |
| FTCD | 0.117223684 | 2.059738412 |
| LRP12 | 0.117255764 | 1.235071717 |
| GOLGA1 | 0.117300082 | 0.911612262 |
| DYNC1H1 | 0.117380898 | 0.916224039 |
| RP11-424M24.3 | 0.14345216 | 0.827201103 |
| TRDN | 0.117442107 | 2.870197775 |
| MESP1 | 0.117471353 | 1.616373824 |
| LFNG | 0.117483213 | 1.410278205 |
| ANKRD36B | 0.117506847 | 1.181961711 |
| RP11-46A10.5 | 0.076828458 | 0.825959719 |
| MOAP1 | 0.117534822 | 1.173340398 |
| RRP15 | 0.117557861 | 0.911535887 |
| TCAM1P | 0.117670129 | 0.825343026 |
| SEBOX | 0.117589281 | 2.239805312 |
| NELFB | 0.117647537 | 0.932916429 |
| BID | 0.117748723 | 0.881035276 |
| CDS2 | 0.117760476 | 0.894491662 |
| AKAP11 | 0.117851781 | 1.126429235 |
| SCAMP1 | 0.117858967 | 1.095125076 |
| IGSF22 | 0.117862564 | 1.299218775 |
| DDX19B | 0.117882403 | 1.105450466 |
| IFI16 | 0.11789859 | 0.839297658 |
| ATAT1 | 0.117936006 | 1.148341805 |
| KLF17 | 0.117957194 | 2.375595559 |
| VPS41 | 0.117965929 | 0.92006932 |
| FOXN3-AS1 | 0.094542352 | 0.822963415 |
| OR2B11 | 0.117968001 | 0.541591996 |
| CCL20 | 0.118032158 | 1.757238853 |
| VPS37D | 0.118127165 | 1.423863435 |
| EMD | 0.118216074 | 1.107887791 |
| CD164 | 0.118263668 | 1.116869677 |
| SIPA1L3 | 0.118280562 | 0.866050743 |
| UGT2B15 | 0.118312456 | 2.205995209 |
| TMC4 | 0.118459191 | 1.195512441 |
| DAP3 | 0.118501652 | 0.92135569 |
| PPP1R3D | 0.118558846 | 0.746215413 |
| KLHL42 | 0.118564433 | 1.172381446 |
| C1orf189 | 0.118591943 | 6.268431773 |
| CLDN2 | 0.118661456 | 13.65870224 |
| UBE2V1P2 | 0.134361512 | 0.819823311 |
| PXN-AS1 | 0.148129211 | 0.819806241 |
| BRSK1 | 0.118704733 | 1.410520778 |
| GFRA4 | 0.118737853 | 16.5031962 |
| UIMC1 | 0.11874963 | 1.086405789 |
| COL4A5 | 0.118833425 | 1.312383922 |
| TXNL1 | 0.118856353 | 0.929116514 |
| OR11A1 | 0.118867124 | 0.400590035 |
| LMO4 | 0.118917419 | 1.179649631 |
| CFP | 0.118982134 | 0.754604781 |
| CERS6-AS1 | 0.108498774 | 0.818279263 |
| ASNS | 0.118995672 | 0.808973646 |
| GAS2L2 | 0.119088898 | 1.790994249 |
| RGS5 | 0.119156079 | 1.527507927 |
| SIRPB1 | 0.119412907 | 0.548579841 |
| ZMYM6NB | 0.119439629 | 1.134997547 |
| BPIFA3 | 0.119470562 | 0.349644172 |
| CARD10 | 0.119536862 | 1.264918166 |
| DYNLL1P1 | 0.072547078 | 0.816289778 |
| ZAR1 | 0.119547209 | 0.541613767 |
| FDPS | 0.119574654 | 0.911111036 |
| AC144530.1 | 0.031245015 | 0.816120828 |
| KLHL10 | 0.119610291 | 1.25142784 |
| AC113189.5 | 0.034142038 | 0.815811179 |
| AC005795.1 | 0.121606155 | 0.815772058 |
| SNORA60 | 0.107023883 | 0.815418097 |
| DSG2-AS1 | 0.104381545 | 0.815215953 |
| EIF1P3 | 0.12665272 | 0.81489994 |
| HSH2D | 0.119627861 | 1.276820721 |
| RBM7 | 0.119668377 | 1.100623426 |
| RP11-111A21.1 | 0.148347754 | 0.814611587 |
| KIAA1045 | 0.119690979 | 0.443502897 |
| KCNJ1 | 0.119700625 | 2.382429898 |
| TFF2 | 0.119735431 | 5.824543019 |
| ITFG1-AS1 | 0.078597188 | 0.814110746 |
| SERTM1 | 0.119817465 | 2.409346435 |
| POC5 | 0.119819018 | 1.095015699 |
| RP5-1085F17.3 | 0.058661879 | 0.813606947 |
| NTN1 | 0.119821883 | 1.338033235 |
| GLRA1 | 0.119858246 | 1.784273853 |
| RP4-620E11.8 | 0.094374934 | 0.812742783 |
| RP11-727F15.9 | 0.100233269 | 0.812522898 |
| IL1R1 | 0.119878759 | 1.209796269 |
| FASN | 0.119944718 | 0.824847843 |
| KCNA3 | 0.1199919 | 0.529681802 |
| PLCH1 | 0.120004463 | 1.709731541 |
| PABPC1P1 | 0.129208993 | 0.811152837 |
| MAGEB1 | 0.120018791 | 13.80730648 |
| DOCK5 | 0.120085471 | 1.196823703 |
| MTERF2 | 0.120195258 | 1.13962219 |
| TRMT10C | 0.120270044 | 0.906350416 |
| PRRT2 | 0.120270285 | 0.717705886 |
| RP1-197B17.4 | 0.071782258 | 0.809807019 |
| DISP2 | 0.120271337 | 0.592527237 |
| AP3B1 | 0.120312756 | 1.079304505 |
| SP2-AS1 | 0.053343445 | 0.808816108 |
| DGCR14 | 0.120367536 | 0.936311563 |
| RP11-196G18.22 | 0.10766302 | 0.808157233 |
| SCARNA24 | 0.128952818 | 0.807793608 |
| ELAC1 | 0.120454859 | 0.900692454 |
| MAPRE3 | 0.120468877 | 0.826575202 |
| RABEPK | 0.120531213 | 0.902130015 |
| ANKRD29 | 0.120579578 | 0.696599175 |
| CTC-510F12.4 | 0.124939428 | 0.806885841 |
| DAW1 | 0.120647377 | 2.225609261 |
| AC068580.5 | 0.10501224 | 0.806226772 |
| RP11-6N17.4 | 0.049118198 | 0.806051415 |
| RPL5P22 | 0.135612717 | 0.805963742 |
| PRKCD | 0.120648126 | 1.129379959 |
| PPP2R1B | 0.120769961 | 1.12419655 |
| USE1 | 0.12080616 | 0.864242617 |
| CCL24 | 0.120835639 | 1.403791345 |
| UBQLN4 | 0.120920342 | 0.900668055 |
| RP11-82H13.2 | 0.140870747 | 0.805145893 |
| CLIC1P1 | 0.124612492 | 0.805092692 |
| PEX11G | 0.120926948 | 0.774220644 |
| AF131215.9 | 0.141545872 | 0.804920563 |
| ALDH16A1 | 0.120949599 | 0.906911932 |
| CYP7B1 | 0.120965523 | 0.765529728 |
| RP11-196G11.3 | 0.074293013 | 0.804525194 |
| HSPA1B | 0.121050226 | 1.317777522 |
| MZB1 | 0.121088093 | 0.595798514 |
| LGMNP1 | 0.105236916 | 0.803889907 |
| PLEKHF1 | 0.121194839 | 1.294324248 |
| AC007387.2 | 0.097787694 | 0.803478955 |
| SEZ6L | 0.121265541 | 15.33776323 |
| RP1-101A2.1 | 0.106557806 | 0.803237443 |
| SLC33A1 | 0.121275633 | 1.103764911 |
| MAN1C1 | 0.121278168 | 1.315727209 |
| ALG1L15P | 0.14618273 | 0.802741016 |
| AC093668.2 | 0.136169997 | 0.802644947 |
| RB1 | 0.121333009 | 1.127108501 |
| RP11-393I2.4 | 0.103850693 | 0.802236824 |
| AC091132.1 | 0.144285314 | 0.802235819 |
| MDK | 0.121341187 | 1.225944987 |
| RP11-1029M24.1 | 0.052383081 | 0.802023183 |
| TIE1 | 0.121363984 | 1.215558479 |
| SAT2 | 0.121374656 | 1.14248038 |
| ST3GAL4 | 0.12139524 | 1.305272624 |
| SLC38A8 | 0.12141605 | 7.673835453 |
| SERPINA3 | 0.12146976 | 1.872346963 |
| CHML | 0.121512291 | 1.177656382 |
| EXOC1 | 0.121514465 | 1.088689167 |
| YY2 | 0.121514797 | 1.192512761 |
| MIR1249 | 0.149297813 | 0.800249885 |
| AK3 | 0.121608841 | 0.752566921 |
| EIF4HP1 | 0.120476418 | 0.799982069 |
| FKBP6 | 0.121718172 | 0.627928844 |
| FOXO1 | 0.121732408 | 1.151770692 |
| YWHAZP2 | 0.085577975 | 0.799379116 |
| RP11-972P1.11 | 0.09165852 | 0.799231941 |
| PHYHIP | 0.121747395 | 1.416433424 |
| MAPKBP1 | 0.121793735 | 0.834956687 |
| KDM3B | 0.121833127 | 1.097881464 |
| RP3-439F8.1 | 0.092359527 | 0.798537912 |
| RP11-770J1.5 | 0.137659252 | 0.798367933 |
| GPR97 | 0.121846509 | 2.597999161 |
| POLR2B | 0.121918321 | 1.103098788 |
| HIVEP2 | 0.121948232 | 1.128250093 |
| AP000275.65 | 0.121984205 | 1.27378592 |
| RP11-84C10.1 | 0.07149726 | 0.797667356 |
| CDH5 | 0.121996598 | 1.370007086 |
| CDK15 | 0.122156245 | 0.775766667 |
| WDR7 | 0.122170716 | 0.91054453 |
| RPS2P5 | 0.039553735 | 0.796508346 |
| TIRAP | 0.122182144 | 1.152325878 |
| CEP250 | 0.122245016 | 0.854400922 |
| HAND1 | 0.122248467 | 53.29600356 |
| CTB-46B19.2 | 0.134858577 | 0.795166175 |
| CUBN | 0.122263887 | 1.28852642 |
| TRPV3 | 0.122280196 | 0.651020836 |
| TMEM50A | 0.122299972 | 1.065680015 |
| BTF3L4P1 | 0.101548746 | 0.794496765 |
| RNF20 | 0.122328623 | 0.923449603 |
| ZNF160 | 0.122347029 | 1.193069762 |
| MAPK4 | 0.122385019 | 6.768018682 |
| ZSWIM2 | 0.122422946 | 2.944464346 |
| BHLHE22 | 0.122431309 | 0.727487712 |
| RASGEF1C | 0.122503585 | 1.955892109 |
| PRPF19 | 0.122594054 | 0.931795073 |
| SNORD12B | 0.08610218 | 0.793274263 |
| MRPL47 | 0.122635138 | 0.869033857 |
| ITPRIPL1 | 0.122642687 | 0.774395659 |
| EIF2S2P4 | 0.113141378 | 0.792886567 |
| TRIM65 | 0.122660406 | 0.830828165 |
| CWH43 | 0.122799565 | 0.367766275 |
| RAD21-AS1 | 0.049751998 | 0.792183283 |
| DOHH | 0.122904704 | 0.863660568 |
| CIRBP-AS1 | 0.044170731 | 0.792128742 |
| ETS2 | 0.122915163 | 1.216476178 |
| EIF4G2 | 0.123094483 | 1.090484411 |
| MAP2K5 | 0.12320138 | 0.917239472 |
| C10orf71 | 0.1232169 | 12.965615 |
| BTBD11 | 0.123359012 | 0.78542934 |
| VCPIP1 | 0.123364055 | 1.084463945 |
| DHX32 | 0.123434206 | 1.108997844 |
| NCKIPSD | 0.123459822 | 1.116832483 |
| STK4-AS1 | 0.055801228 | 0.791166709 |
| CHAC2 | 0.123467197 | 0.848332535 |
| SLC20A1 | 0.123536377 | 1.134136246 |
| NHLH2 | 0.123598698 | 0.644131919 |
| RP11-442N24__B.1 | 0.056511166 | 0.790515906 |
| LMAN1 | 0.12372734 | 1.105495175 |
| SLC9B1 | 0.123791911 | 0.792954756 |
| ADRB3 | 0.123856802 | 3.202972943 |
| CPSF6 | 0.123858942 | 1.066946046 |
| SNORA5A | 0.130951941 | 0.789515533 |
| SUV39H2 | 0.123906477 | 1.100361294 |
| RP11-535C21.3 | 0.136627267 | 0.789271149 |
| FAM25A | 0.123995745 | 0.425744474 |
| AP000304.12 | 0.124013503 | 0.586861135 |
| NFXL1 | 0.124015091 | 1.125694199 |
| RP11-615I2.6 | 0.121116983 | 0.788735376 |
| XRCC3 | 0.124031975 | 0.894287807 |
| MTND5P28 | 0.1118993 | 0.78766512 |
| HIST2H2BC | 0.060824529 | 0.787566712 |
| HAUS4 | 0.124048375 | 0.883838813 |
| PPIC | 0.124161732 | 1.144321292 |
| MRPL4 | 0.124277368 | 0.866597977 |
| RP11-108K3.1 | 0.148027671 | 0.78675805 |
| BNIP3P11 | 0.117671998 | 0.786713871 |
| RP11-66B24.4 | 0.130430373 | 0.786616114 |
| ANKRD10 | 0.124315287 | 1.202219926 |
| HARS2 | 0.12433697 | 1.082161542 |
| WASH7P | 0.058137818 | 0.785912553 |
| ANK3 | 0.124369298 | 0.861384951 |
| BET1L | 0.124423675 | 1.071555235 |
| RPS10L | 0.059227933 | 0.785282287 |
| CTD-2292P10.4 | 0.081544411 | 0.785068387 |
| JAGN1 | 0.124436873 | 1.105246953 |
| SERBP1P1 | 0.100987166 | 0.78465677 |
| ADM | 0.124528805 | 1.356502204 |
| SPRY2 | 0.124568718 | 1.332627744 |
| RP11-522I20.3 | 0.014005722 | 0.783557016 |
| ANO7 | 0.124599707 | 0.639640251 |
| RP11-458F8.3 | 0.122105148 | 0.783266457 |
| DHFR | 0.124611407 | 1.138550612 |
| ZNF682 | 0.124634423 | 1.413845039 |
| EMC9 | 0.124644038 | 0.851968259 |
| RPL21P134 | 0.104657467 | 0.782582607 |
| CD33 | 0.124654265 | 0.740477165 |
| PFDN4 | 0.12471063 | 0.869449651 |
| RP5-882O7.4 | 0.116048168 | 0.781858257 |
| KIF2C | 0.12472104 | 0.906669002 |
| VAPB | 0.124741018 | 0.916226708 |
| SETP3 | 0.137929254 | 0.781639 |
| ELP6 | 0.124808026 | 1.10723871 |
| LRRN1 | 0.124850227 | 1.828337804 |
| WDTC1 | 0.124963255 | 1.108206919 |
| RP11-15N24.4 | 0.091005928 | 0.780926457 |
| AC079951.1 | 0.142275217 | 0.780497109 |
| MSLNL | 0.125008356 | 4.062768664 |
| RP11-177C12.1 | 0.12998876 | 0.780213809 |
| CHRM2 | 0.125015085 | 3.753120282 |
| DOLPP1 | 0.125026959 | 0.908953949 |
| MRPS31 | 0.125053021 | 1.094863407 |
| ACCS | 0.125076958 | 1.183314179 |
| AC068137.13 | 0.149095192 | 0.779499806 |
| C1orf101 | 0.125084547 | 1.173801145 |
| CCDC58P3 | 0.143665073 | 0.779124293 |
| SNHG11 | 0.042319662 | 0.778962451 |
| CLK1 | 0.125086749 | 1.112104315 |
| AP001469.9 | 0.018339147 | 0.77885922 |
| TMEM158 | 0.125168934 | 1.456978934 |
| KHDRBS1 | 0.125212486 | 1.051237741 |
| TSSK1A | 0.119596411 | 0.777984748 |
| RP11-131L12.3 | 0.138998737 | 0.777830398 |
| OSTN | 0.125219878 | 2.257745808 |
| CTD-3214H19.6 | 0.073352866 | 0.777476885 |
| AC016907.3 | 0.107083946 | 0.777458347 |
| ROR1 | 0.125232142 | 0.708309598 |
| RP11-733D4.2 | 0.144629034 | 0.776833993 |
| RP11-803P9.1 | 0.064731003 | 0.776816425 |
| RP11-44F14.7 | 0.103124429 | 0.776129582 |
| TPI1P2 | 0.049266371 | 0.775868502 |
| FBXO8 | 0.125261731 | 1.094224768 |
| REP15 | 0.125288174 | 1.211695528 |
| ZNF35 | 0.125366329 | 1.118090193 |
| GALNT16 | 0.125378275 | 1.595534137 |
| YBX2 | 0.12541893 | 1.271760851 |
| TIMM23B | 0.125438318 | 1.105027797 |
| AL672183.2 | 0.084562666 | 0.774552331 |
| NEO1 | 0.12551458 | 1.168939519 |
| SDC3 | 0.125531176 | 1.219104777 |
| NEU4 | 0.125549701 | 2.208580919 |
| SUSD5 | 0.125608167 | 1.63413775 |
| C1orf226 | 0.125730774 | 1.222445666 |
| chr22-38_28785274-29006793.1 | 0.119144029 | 0.774089627 |
| RP5-894A10.6 | 0.092379756 | 0.774046947 |
| MMP26 | 0.125770237 | 4.384144263 |
| RGS10 | 0.125779947 | 1.170377225 |
| CEACAM1 | 0.125893005 | 1.328292076 |
| RP1-149A16.16 | 0.016660734 | 0.773117306 |
| NDUFAF3 | 0.125897292 | 1.130075424 |
| BDNF | 0.125897988 | 1.891472598 |
| AKAP2 | 0.125904931 | 1.489322548 |
| BCKDHB | 0.125907822 | 1.146605193 |
| RP11-513I15.6 | 0.027593626 | 0.772343862 |
| GXYLT1 | 0.125936989 | 1.107095938 |
| KRT85 | 0.125944166 | 2.02867197 |
| CCDC36 | 0.126053963 | 0.713903015 |
| STX7 | 0.126127453 | 1.098012 |
| C20orf24 | 0.126134091 | 0.891260157 |
| ADAMDEC1 | 0.126144274 | 0.550440302 |
| CTC-378H22.1 | 0.150260034 | 0.771856498 |
| ADCY7 | 0.126162157 | 0.800707656 |
| BMP4 | 0.126166268 | 2.610765557 |
| TMX2 | 0.126217176 | 0.923745573 |
| RP11-61K9.2 | 0.141484663 | 0.771202793 |
| GTF2IRD1P1 | 0.107589132 | 0.770155539 |
| EPRS | 0.126335435 | 0.902620304 |
| PPP2R2C | 0.12653581 | 0.670034705 |
| LGI3 | 0.126541975 | 0.424123365 |
| SLC1A3 | 0.126578741 | 1.377076556 |
| TTC33 | 0.126618679 | 1.136012958 |
| RNU6V | 0.132786683 | 0.769211862 |
| AC098820.4 | 0.138663013 | 0.769192138 |
| PLXNC1 | 0.126636083 | 0.774667977 |
| ATP5G1P4 | 0.114332484 | 0.768892904 |
| ZNF563 | 0.12664267 | 1.217637044 |
| P2RY1 | 0.126648186 | 0.736956647 |
| CTB-32O4.2 | 0.072613314 | 0.768002187 |
| RP11-862L9.2 | 0.10750584 | 0.767900405 |
| PTPRVP | 0.105082667 | 0.767455642 |
| RASL11B | 0.126702556 | 2.085547487 |
| MYLK4 | 0.126769798 | 1.280176224 |
| LYRM7 | 0.126891177 | 0.900623046 |
| RP11-36C20.1 | 0.084248764 | 0.767099221 |
| ZNF232 | 0.126965603 | 1.155888635 |
| RP11-368J21.3 | 0.147808252 | 0.766795264 |
| RP11-434B12.1 | 0.074836219 | 0.766705402 |
| DTX2P1-UPK3BP1-PMS2P11 | 0.02128122 | 0.766669678 |
| RP11-111F5.5 | 0.13422871 | 0.766647786 |
| GS1-114I9.3 | 0.127115129 | 0.773396121 |
| CTD-3010D24.3 | 0.115825007 | 0.766195748 |
| UCK2 | 0.127126725 | 0.897011048 |
| CCDC132 | 0.127140978 | 0.913587948 |
| CGREF1 | 0.1271707 | 0.64626175 |
| ABHD17AP3 | 0.077023106 | 0.765926044 |
| RP1-146I3.1 | 0.121329754 | 0.765692113 |
| RAB31 | 0.127195884 | 1.208499739 |
| POLK | 0.127314672 | 1.10544689 |
| RP11-120K18.2 | 0.142620516 | 0.765277947 |
| VSTM2B | 0.127418003 | 0.228580249 |
| RP11-247L20.3 | 0.114482886 | 0.765016928 |
| RP11-133K1.9 | 0.122001999 | 0.764994129 |
| JMJD7 | 0.127464246 | 0.752710977 |
| ZPLD1 | 0.127519315 | 1.661101829 |
| RP3-337O18.9 | 0.143385413 | 0.763991688 |
| TIMP1 | 0.127536245 | 1.357681979 |
| RP4-669P10.20 | 0.049014811 | 0.763791768 |
| MAP6D1 | 0.127577262 | 0.831901007 |
| HKDC1 | 0.127725427 | 1.66590679 |
| RP11-658F2.3 | 0.142954972 | 0.763443128 |
| SFN | 0.127734564 | 0.820613134 |
| RP1-68D18.4 | 0.051540053 | 0.762758938 |
| AMY1B | 0.127800539 | 8.076108442 |
| CLDN10 | 0.127847353 | 1.697862933 |
| VEZF1 | 0.127885383 | 1.126556521 |
| PIK3R6 | 0.127926191 | 0.792803308 |
| RP11-715J22.1 | 0.144172734 | 0.761990803 |
| CH17-302M23.1 | 0.096108288 | 0.761920774 |
| KRT10 | 0.127930386 | 0.465112338 |
| BRI3P1 | 0.109438302 | 0.761413847 |
| CTD-2636A23.2 | 0.090909916 | 0.76139002 |
| LINC01588 | 0.025734963 | 0.761278039 |
| RN7SL138P | 0.06646014 | 0.761198099 |
| PJA2 | 0.128075282 | 1.117702239 |
| RP11-864I4.3 | 0.1396903 | 0.761042199 |
| RP11-895M11.2 | 0.125285739 | 0.760873556 |
| MFF | 0.128096481 | 1.097503145 |
| RP5-1165K10.2 | 0.093145635 | 0.760668824 |
| AC010733.5 | 0.122985771 | 0.760290467 |
| FDPSP7 | 0.144342728 | 0.759975821 |
| CERS2 | 0.128185688 | 1.108017348 |
| AL356585.3 | 0.060142205 | 0.75933134 |
| LYRM5 | 0.128205072 | 1.156643413 |
| AC021188.4 | 0.143651571 | 0.758619913 |
| PLBD1-AS1 | 0.063674211 | 0.758601704 |
| NAPSB | 0.079564209 | 0.758088401 |
| C18orf42 | 0.128305781 | 2.608690701 |
| MAD2L1 | 0.128317844 | 1.120799161 |
| SNHG17 | 0.018361084 | 0.757847296 |
| SYNPO | 0.12834359 | 1.184932032 |
| TRIOBP | 0.128377004 | 0.891322306 |
| RP11-1149M10.2 | 0.147084477 | 0.757472478 |
| RNF157 | 0.128395567 | 1.397552642 |
| HIST2H2BD | 0.078728096 | 0.756907596 |
| SPATA16 | 0.128406395 | 3.2892135 |
| RP11-350G24.2 | 0.13851371 | 0.756172709 |
| ARL4C | 0.128421845 | 0.809934764 |
| RP1-253P7.4 | 0.110120505 | 0.755517223 |
| ZIC5 | 0.128494135 | 0.790683191 |
| RP4-655J12.4 | 0.071631339 | 0.755504414 |
| MAGEA9B | 0.128540834 | 11.83512518 |
| SIAH2 | 0.128542236 | 1.19300164 |
| SLC35A3 | 0.128554417 | 1.159541432 |
| RP11-573D15.2 | 0.128582435 | 0.75447091 |
| EDRF1 | 0.12856596 | 1.101827594 |
| PRR34 | 0.128583216 | 0.867328654 |
| RPL35P2 | 0.113518394 | 0.754042127 |
| PLEKHM1 | 0.128595065 | 0.873138646 |
| C9orf85 | 0.128597765 | 0.906049577 |
| CTD-2523D13.2 | 0.064176756 | 0.752999462 |
| CTAGE4 | 0.128627946 | 0.724043192 |
| HTRA4 | 0.128726226 | 0.510809547 |
| RP11-61L23.2 | 0.049737888 | 0.752602117 |
| NAT9 | 0.128736985 | 0.901606736 |
| RP11-396C23.2 | 0.047744958 | 0.752564143 |
| WASH7P | 0.041116437 | 0.752347685 |
| PRKAG3 | 0.128753948 | 2.117386302 |
| IPO13 | 0.128791575 | 0.920454927 |
| RAC1P2 | 0.014649528 | 0.752065044 |
| AL022345.7 | 0.102207552 | 0.751916544 |
| CARF | 0.128818312 | 1.160413338 |
| AC007283.4 | 0.151195417 | 0.751409312 |
| NYAP2 | 0.128831297 | 1.838711314 |
| MRGPRX4 | 0.128861025 | 5.800223991 |
| SEC22A | 0.128878978 | 0.910577146 |
| CTC-281F24.1 | 0.130962071 | 0.75066802 |
| FAM135A | 0.128894611 | 0.827110935 |
| PATE4 | 0.128938204 | 1.507680657 |
| RP11-23P13.4 | 0.124880309 | 0.749867149 |
| C2 | 0.129078299 | 1.373649872 |
| MTMR2 | 0.129299791 | 1.205324727 |
| DNAI2 | 0.12930875 | 3.517064248 |
| MGEA5 | 0.129411009 | 1.08384012 |
| SNORD11 | 0.136078792 | 0.749042353 |
| GAS2L1 | 0.129413229 | 0.885932203 |
| RP11-568N6.1 | 0.055785045 | 0.748300536 |
| RP13-467H17.1 | 0.128688293 | 0.748273832 |
| IGF1R | 0.129472005 | 1.189120633 |
| CTD-2017C7.1 | 0.069117401 | 0.748104844 |
| RP11-383J24.2 | 0.143202864 | 0.747798806 |
| RP11-895M11.3 | 0.091513257 | 0.747678589 |
| RP11-413M3.4 | 0.050504247 | 0.747504657 |
| RP11-70P17.1 | 0.032726606 | 0.747310694 |
| C20orf141 | 0.129488393 | 2.350834932 |
| SERBP1P6 | 0.07522927 | 0.747191674 |
| RP11-440G5.2 | 0.077348415 | 0.747033154 |
| AC004156.3 | 0.10089405 | 0.746847332 |
| RP4-597N16.4 | 0.137415754 | 0.746804164 |
| UPK1B | 0.129501699 | 0.334864454 |
| DCAF10 | 0.129519274 | 1.074169177 |
| S100P | 0.129529957 | 1.470711531 |
| HSPE1P6 | 0.117325492 | 0.746229822 |
| TAF4B | 0.129581172 | 0.844159522 |
| RP11-745A24.1 | 0.08979898 | 0.745925292 |
| RP11-66B24.9 | 0.113933844 | 0.745771177 |
| DERL2 | 0.129601183 | 1.079313975 |
| RP11-262H14.5 | 0.113618929 | 0.745252391 |
| AL353662.3 | 0.10814367 | 0.744828237 |
| OR51J1 | 0.129632392 | 5.275592215 |
| AC106900.6 | 0.130253533 | 0.74469205 |
| C1orf147 | 0.081186938 | 0.744677482 |
| RP11-717A5.2 | 0.03756637 | 0.744389003 |
| RP4-621N11.2 | 0.074072214 | 0.743936028 |
| AP000320.6 | 0.081468409 | 0.743892774 |
| MGC45922 | 0.070222928 | 0.743747717 |
| RP11-91J19.2 | 0.11148253 | 0.743495741 |
| Y_RNA | 0.073136816 | 0.7428599 |
| RP11-378A13.2 | 0.14268707 | 0.742819803 |
| UBAP1L | 0.12968515 | 1.188589238 |
| SCARNA12 | 0.018034541 | 0.741606555 |
| STK40 | 0.129705351 | 0.899419107 |
| RP11-723O4.2 | 0.034292649 | 0.741362878 |
| RP11-467L13.7 | 0.092021174 | 0.741011474 |
| SYT12 | 0.129871714 | 1.726682731 |
| CNGB3 | 0.129912586 | 0.682039294 |
| LRRC9 | 0.129917964 | 2.7298489 |
| CRYGC | 0.129945012 | 3.358008642 |
| CHMP4B | 0.130000303 | 0.898529989 |
| C11orf88 | 0.130049604 | 3.447675413 |
| INSL4 | 0.1301062 | 11.05359325 |
| RP11-521I2.3 | 0.04580553 | 0.740088566 |
| RP11-203J24.8 | 0.127323727 | 0.739940327 |
| PRR14L | 0.130143986 | 0.904319367 |
| IRS1 | 0.130144393 | 1.228997843 |
| RPL21P8 | 0.11643721 | 0.73961875 |
| TMEM191C | 0.123988715 | 0.739577051 |
| PTPRM | 0.130150239 | 1.39683176 |
| RP11-452I5.2 | 0.066184077 | 0.739443405 |
| FRRS1 | 0.130198504 | 0.774357809 |
| CTD-3148I10.15 | 0.121719522 | 0.738973006 |
| RP11-383G10.3 | 0.118559809 | 0.738408808 |
| PMS2P10 | 0.061692287 | 0.738365964 |
| RP11-46I8.3 | 0.114578548 | 0.737818509 |
| C1S | 0.13040715 | 1.254927611 |
| KIAA1279 | 0.130434814 | 1.085978784 |
| HMGN2 | 0.130582119 | 1.102477184 |
| HPN-AS1 | 0.138996884 | 0.736942778 |
| KCNJ18 | 0.130591624 | 2.578976837 |
| RP11-467J12.4 | 0.032005319 | 0.736184114 |
| MCTS2P | 0.074150141 | 0.735958894 |
| KRCC1 | 0.130611194 | 1.129057506 |
| BEX4 | 0.130637224 | 1.351046143 |
| ANKRD13B | 0.130655568 | 0.789773068 |
| CHD9 | 0.130667744 | 0.888249672 |
| RPL15 | 0.130718596 | 1.107118071 |
| RP11-3L10.3 | 0.094608937 | 0.734251152 |
| AC005264.2 | 0.043418249 | 0.734161994 |
| RPS6KA4 | 0.130727341 | 0.890208708 |
| RP11-573D15.1 | 0.093641671 | 0.733861586 |
| APOBEC4 | 0.130736527 | 6.198032253 |
| AC005625.1 | 0.11672151 | 0.73313804 |
| HSPE1P8 | 0.130917262 | 0.732937431 |
| ZNF474 | 0.130861958 | 2.029898124 |
| LINC00957 | 0.001927601 | 0.732625117 |
| AC019048.1 | 0.106138337 | 0.732327441 |
| RP11-467H10.2 | 0.046783825 | 0.732286732 |
| EZR-AS1 | 0.139640571 | 0.732269742 |
| ZNF444P1 | 0.13648807 | 0.731784245 |
| HPS4 | 0.130893922 | 0.893140581 |
| LGMN | 0.130940117 | 0.866612105 |
| NXPH4 | 0.130945058 | 0.775639542 |
| MAGED1 | 0.130999782 | 1.188220933 |
| C19orf67 | 0.1310744 | 1.706377922 |
| RP4-545L17.11 | 0.141196102 | 0.730191924 |
| AC016747.3 | 0.056491995 | 0.730135096 |
| Z83844.1 | 0.023069193 | 0.730069744 |
| TMEM160 | 0.131104038 | 0.725911694 |
| CHD8 | 0.13113138 | 0.909056811 |
| WBP2NL | 0.13122614 | 0.818875284 |
| PIM1 | 0.131228805 | 0.837692928 |
| WEE1 | 0.131235656 | 1.135630343 |
| NPTX1 | 0.131257473 | 8.517943183 |
| LA16c-360A4.1 | 0.125906581 | 0.727349053 |
| DPYD-AS1 | 0.13324699 | 0.726927709 |
| Metazoa_SRP | 0.109879173 | 0.726840314 |
| XXbac-B444P24.10 | 0.072800657 | 0.726624538 |
| RP5-963E22.4 | 0.133820524 | 0.726273693 |
| AC074286.1 | 0.125885603 | 0.72625254 |
| AC091177.1 | 0.123651147 | 0.726134449 |
| PWP2 | 0.13133403 | 0.673953805 |
| SRPX | 0.131377288 | 1.483685107 |
| ZNF519 | 0.131391999 | 1.160610628 |
| F2RL2 | 0.131398736 | 0.554326699 |
| AC022431.2 | 0.131412997 | 1.417888493 |
| NLK | 0.131485679 | 1.096741785 |
| RP11-354M1.2 | 0.082541614 | 0.725182489 |
| RP11-540O11.1 | 0.036932251 | 0.725181725 |
| LINC00885 | 0.110669774 | 0.725119362 |
| PARD3-AS1 | 0.090995816 | 0.724937216 |
| RP1-78B3.1 | 0.020918723 | 0.724867074 |
| RP11-593F23.1 | 0.113380159 | 0.724855437 |
| RP11-20I23.10 | 0.074771992 | 0.724438752 |
| RP11-198M15.1 | 0.03971661 | 0.724421867 |
| ADH6 | 0.131505398 | 1.420672987 |
| AC090587.4 | 0.036560767 | 0.723790147 |
| HLA-DRB1 | 0.131518557 | 1.285700785 |
| GLIS3 | 0.131525913 | 1.45967734 |
| TRUB2 | 0.131550053 | 0.925996927 |
| CMYA5 | 0.13158594 | 1.443279034 |
| AC027612.1 | 0.105575392 | 0.723074694 |
| RP11-783K16.5 | 0.037374159 | 0.722297678 |
| RP11-136H19.1 | 0.120896023 | 0.722111924 |
| NDUFA5P11 | 0.066007665 | 0.722001228 |
| TFAM | 0.131615156 | 1.105159949 |
| SNRNP48 | 0.131632152 | 1.1097848 |
| RP4-778K6.1 | 0.061347505 | 0.721191613 |
| RP11-64C12.3 | 0.127099371 | 0.721069075 |
| MIR6859-3 | 0.123202128 | 0.720711788 |
| KAAG1 | 0.131656499 | 1.919452126 |
| RNF146 | 0.131777849 | 1.092323907 |
| CTD-3064M3.4 | 0.092793756 | 0.719535684 |
| RP11-135J2.3 | 0.074703246 | 0.719497232 |
| C11orf49 | 0.131788328 | 1.161554624 |
| KCNAB2 | 0.131790106 | 0.824014096 |
| RP11-160H22.5 | 0.029308067 | 0.718708911 |
| RP11-8L8.2 | 0.06372365 | 0.718416725 |
| RP11-395L14.17 | 0.075176271 | 0.718402595 |
| RP11-423F24.3 | 0.076996916 | 0.718318051 |
| LYPD6 | 0.131895637 | 1.216112388 |
| CTB-32O4.3 | 0.127037841 | 0.71816794 |
| NKAP | 0.131938933 | 1.095876295 |
| VSX1 | 0.131989451 | 0.52683595 |
| RP11-426K3.1 | 0.10051072 | 0.717951056 |
| RAB35 | 0.131999946 | 0.929050108 |
| CST1 | 0.132003909 | 18.87870025 |
| MAFIP | 0.133765219 | 0.717813728 |
| GNAT1 | 0.132014975 | 0.661665154 |
| C5orf46 | 0.132049835 | 4.388316119 |
| YBX1P4 | 0.084682206 | 0.716897357 |
| KIAA1586 | 0.132091369 | 1.112942826 |
| RP11-337N6.2 | 0.101292851 | 0.716845346 |
| RPL13P12 | 0.071006412 | 0.716588312 |
| TP63 | 0.132104836 | 0.792651838 |
| RALY-AS1 | 0.080974125 | 0.715692067 |
| CTD-2024I7.1 | 0.112069446 | 0.715626775 |
| APOC1P1 | 0.108729238 | 0.715587101 |
| MLYCD | 0.132234961 | 1.104908108 |
| AC002401.1 | 0.134908457 | 0.714940777 |
| PABPC1P10 | 0.135818584 | 0.714268674 |
| C17orf89 | 0.132255821 | 0.814797642 |
| JRK | 0.132277039 | 1.15367193 |
| WLS | 0.132301813 | 1.193515865 |
| AL365502.1 | 0.093133488 | 0.713576153 |
| RP11-10N16.3 | 0.075734634 | 0.713319466 |
| RP11-185E8.2 | 0.081265036 | 0.713264936 |
| FAAH | 0.132348174 | 1.193704158 |
| CTD-2527I21.5 | 0.146230594 | 0.712207426 |
| PSPC1 | 0.132385938 | 1.08529527 |
| RP11-131H24.4 | 0.097299366 | 0.711510136 |
| ERBB4 | 0.132422433 | 1.81466232 |
| RP11-314N13.9 | 0.078561165 | 0.711374475 |
| TTC3 | 0.132423039 | 1.13045759 |
| RASSF2 | 0.132524892 | 0.765098809 |
| NONOP2 | 0.063615265 | 0.710655575 |
| ETNK1 | 0.132528219 | 1.231020178 |
| TNK2-AS1 | 0.017844069 | 0.710462977 |
| RP11-486L19.2 | 0.148140068 | 0.710459641 |
| WI2-89031B12.1 | 0.131227868 | 0.709009041 |
| RBBP4P4 | 0.123690987 | 0.708725487 |
| MUC22 | 0.132578157 | 2.159186285 |
| RP11-390P2.2 | 0.080233374 | 0.708359923 |
| NCOA7 | 0.132629404 | 1.253122498 |
| METTL7B | 0.132647719 | 1.655929194 |
| CLIC4 | 0.132684731 | 1.126300184 |
| TMEM242 | 0.132696538 | 1.09625174 |
| INGX | 0.109466272 | 0.70759244 |
| RILPL2 | 0.132718074 | 1.136600043 |
| AC004985.12 | 0.04339895 | 0.707401633 |
| RP11-1018N14.5 | 0.122468514 | 0.707320049 |
| PA2G4P1 | 0.090928008 | 0.707087142 |
| CCDC97 | 0.132722649 | 0.915466553 |
| UPK3BP1 | 0.07720859 | 0.70672668 |
| RP5-894A10.2 | 0.088082917 | 0.706506373 |
| ERI3-IT1 | 0.067914678 | 0.706477172 |
| RP3-395M20.3 | 0.144978839 | 0.706101842 |
| SLC7A5P1 | 0.103273095 | 0.706017727 |
| RP11-58E21.3 | 0.120790748 | 0.705882833 |
| TNFSF13 | 0.13275515 | 1.182281891 |
| ACTG1 | 0.132828361 | 1.075887233 |
| MT1XP1 | 0.034636605 | 0.705032499 |
| RP11-798G7.5 | 0.114608175 | 0.704557237 |
| AC090587.5 | 0.130145503 | 0.704415955 |
| PI4KB | 0.132833297 | 0.925862503 |
| SAP18 | 0.132889965 | 1.094937682 |
| RP5-823G15.5 | 0.078510855 | 0.703985937 |
| PIK3CD-AS2 | 0.118257335 | 0.703932354 |
| HOXD-AS2 | 0.029078343 | 0.703403976 |
| CBFB | 0.132998903 | 1.081082304 |
| AC104699.1 | 0.113008095 | 0.702729862 |
| HSPA9P1 | 0.016570735 | 0.702398146 |
| RP11-624L4.1 | 0.08086591 | 0.702237328 |
| NEK11 | 0.13300605 | 1.219656285 |
| RP11-92K15.3 | 0.081299229 | 0.701858439 |
| CTB-174O21.2 | 0.080775773 | 0.701168245 |
| RP11-301G21.1 | 0.112308514 | 0.701110891 |
| MIR221 | 0.141319288 | 0.700772664 |
| RP11-480C16.1 | 0.134856579 | 0.700331834 |
| MRPL45 | 0.133072488 | 0.908953127 |
| TRPV1 | 0.133075051 | 1.278269967 |
| SEC11C | 0.133079134 | 0.888984506 |
| AC069257.8 | 0.08088338 | 0.699454926 |
| SHISA2 | 0.133102806 | 1.604519172 |
| RP11-8H2.1 | 0.13769285 | 0.698882976 |
| LRRC46 | 0.133184722 | 1.505692943 |
| RP11-58K22.5 | 0.061318325 | 0.698645526 |
| B3GNT8 | 0.13324269 | 0.763970636 |
| CALCRL | 0.133293512 | 1.257301636 |
| RP11-159F24.5 | 0.113576716 | 0.697472577 |
| LRRC38 | 0.13333544 | 2.193089567 |
| snoU13 | 0.036965645 | 0.696763706 |
| RP11-409K20.7 | 0.051574075 | 0.696698993 |
| FAM24A | 0.133358005 | 0.369321268 |
| CYP27A1 | 0.133403498 | 0.65627353 |
| PITPNC1 | 0.133440917 | 1.186704153 |
| MSL3P1 | 0.020668048 | 0.696302721 |
| RP11-700P18.1 | 0.047520222 | 0.695975448 |
| LINC00304 | 0.108803719 | 0.695803489 |
| MAMDC2 | 0.133467118 | 2.791792475 |
| NOP56P3 | 0.07234035 | 0.693962772 |
| RP5-1142J19.2 | 0.140998452 | 0.69379133 |
| CCNJ | 0.133494856 | 1.158016901 |
| RPL22L1 | 0.133516961 | 1.30504926 |
| RP11-713C19.2 | 0.108812378 | 0.693363798 |
| RP11-417L19.6 | 0.070335522 | 0.693130999 |
| RP4-651E10.4 | 0.147863026 | 0.693071585 |
| CNOT7P1 | 0.086743162 | 0.692832727 |
| PPP1R3A | 0.133597982 | 3.546854569 |
| FTLP5 | 0.095706715 | 0.692270066 |
| MDH2 | 0.133634913 | 0.902550009 |
| HM13-IT1 | 0.09138292 | 0.69096942 |
| CYCSP38 | 0.139741962 | 0.69052765 |
| OVOL1-AS1 | 0.079023632 | 0.690366865 |
| RDH5 | 0.133665083 | 1.275907138 |
| AL627171.2 | 0.107142825 | 0.689685515 |
| PMS2L2 | 0.115939419 | 0.689575651 |
| RP1-21O18.3 | 0.047879368 | 0.689546923 |
| Y_RNA | 0.062852865 | 0.689113929 |
| PSMD8P1 | 0.033968386 | 0.688493591 |
| RP11-419M24.4 | 0.135430509 | 0.688423051 |
| RP4-550H1.5 | 0.135644745 | 0.688088112 |
| RP4-647C14.2 | 0.138586262 | 0.688082455 |
| RP4-616B8.5 | 0.005630402 | 0.687909813 |
| MIR5010 | 0.0757084 | 0.687854996 |
| ERICH6 | 0.13369092 | 1.211834763 |
| RP11-635N19.3 | 0.144664849 | 0.687590066 |
| SNRNP70 | 0.133703548 | 0.908923734 |
| RP11-157D23.2 | 0.136116514 | 0.687504141 |
| LRRC3C | 0.133823307 | 2.58538006 |
| PEPD | 0.133853952 | 0.894177404 |
| Y_RNA | 0.112716248 | 0.687315836 |
| NUF2 | 0.133921754 | 0.893949644 |
| SLAMF9 | 0.134061602 | 2.056898029 |
| RP11-449H3.2 | 0.103934687 | 0.686928302 |
| RP11-323C15.2 | 0.07674751 | 0.686695948 |
| CERS3-AS1 | 0.057841927 | 0.686547076 |
| KLHL12 | 0.134066001 | 0.909941524 |
| TPK1 | 0.134117683 | 1.180940874 |
| AC124789.1 | 0.032480497 | 0.686088895 |
| ALS2CL | 0.134166941 | 1.191839978 |
| OR10V1 | 0.134171233 | 4.606127454 |
| CXorf38 | 0.134202688 | 1.111952305 |
| CSNK2A1 | 0.134222753 | 0.916014912 |
| NOP56P1 | 0.073780367 | 0.685445101 |
| SMURF2P1 | 0.091766202 | 0.685223408 |
| MYO3B | 0.134257588 | 1.568401231 |
| RP11-867G23.1 | 0.08803218 | 0.685075669 |
| RP11-494O16.4 | 0.074492534 | 0.684903081 |
| FAIM | 0.134336004 | 1.110384329 |
| PGDP1 | 0.108638297 | 0.684528441 |
| MOCS3 | 0.134406356 | 0.911692536 |
| RP11-94D20.1 | 0.073501029 | 0.684391767 |
| TBC1D27 | 0.129537844 | 0.684341619 |
| LYPLA1P3 | 0.025198827 | 0.684294882 |
| AL020997.1 | 0.123861548 | 0.68377822 |
| AC097711.1 | 0.123424305 | 0.683628018 |
| OR1Q1 | 0.13444884 | 1.590641624 |
| AC010980.2 | 0.052208418 | 0.682674971 |
| RNPC3 | 0.13447428 | 1.161962164 |
| AC006547.14 | 0.134505334 | 1.417416249 |
| EGFR-AS1 | 0.124160332 | 0.681897583 |
| LHFPL3 | 0.134561082 | 2.43368594 |
| PTH2R | 0.134565889 | 0.406077825 |
| EIF5 | 0.134573511 | 0.91264693 |
| RP11-51F16.1 | 0.001508661 | 0.680265178 |
| TMEM145 | 0.134655159 | 0.627788413 |
| FABP5P2 | 0.101183866 | 0.680032314 |
| AC004386.4 | 0.13772883 | 0.680030769 |
| XXbac-BPG13B8.10 | 0.105005454 | 0.679847882 |
| GALNT15 | 0.134655413 | 1.465405958 |
| HS6ST1P1 | 0.059589246 | 0.679618928 |
| TIMM23 | 0.134655764 | 1.069872675 |
| ADORA2A-AS1 | 0.150511189 | 0.67900454 |
| FAM83H-AS1 | 0.0017706 | 0.678708866 |
| ATP6V0CP1 | 0.0430187 | 0.678691619 |
| RP11-44F14.8 | 0.049230299 | 0.67843259 |
| RP11-114B7.6 | 0.072464207 | 0.678127052 |
| HNF1A | 0.134674104 | 2.037610709 |
| RN7SL118P | 0.13804747 | 0.677914829 |
| SLC25A5P8 | 0.111698594 | 0.677783547 |
| HSD17B13 | 0.134678488 | 7.30080177 |
| SLC5A3 | 0.134682569 | 1.144056398 |
| TMEM239 | 0.134730848 | 1.770013535 |
| RORA | 0.134734236 | 0.793948656 |
| RP11-310P5.1 | 0.043581228 | 0.677000974 |
| RP11-394O2.3 | 0.040641517 | 0.676834047 |
| GGN | 0.134754542 | 0.75312417 |
| GABRB1 | 0.134755938 | 1.975175748 |
| RP11-104H15.10 | 0.07635695 | 0.676578033 |
| PHGR1 | 0.134757819 | 0.057196887 |
| AC005624.2 | 0.022657986 | 0.676413655 |
| CLEC7A | 0.13476459 | 0.500247811 |
| CTD-2286N8.1 | 0.142901337 | 0.675668325 |
| CTD-2184D3.3 | 0.119722931 | 0.675623053 |
| AC009501.4 | 0.068717406 | 0.675178611 |
| PQLC1 | 0.134784424 | 0.91651396 |
| SNRPEP6 | 0.020486703 | 0.675072304 |
| HCFC1-AS1 | 0.099546668 | 0.675004437 |
| APRT | 0.134800716 | 0.88776611 |
| PLEKHG4 | 0.134821905 | 0.879079164 |
| LINC00582 | 0.142371447 | 0.674803332 |
| RP11-447H19.4 | 0.087825173 | 0.674705966 |
| LINC01415 | 0.110964302 | 0.674514283 |
| RNY3P8 | 0.132660765 | 0.674488368 |
| TPTE2P2 | 0.102185275 | 0.674263521 |
| PRDM16 | 0.135016742 | 1.59439727 |
| RP11-544M22.8 | 0.030935815 | 0.673569375 |
| RP11-553L6.2 | 0.094709365 | 0.673407618 |
| MIR4793 | 0.103067238 | 0.673130869 |
| LLNLR-304A6.1 | 0.103072652 | 0.672773888 |
| RP11-501J20.5 | 0.113240303 | 0.672768879 |
| RP3-514P16.1 | 0.044055478 | 0.672711184 |
| RP11-74M13.5 | 0.142918105 | 0.67251704 |
| C1QL2 | 0.135024284 | 0.233177513 |
| SPCS3 | 0.135083838 | 1.082632782 |
| FZD8 | 0.135093768 | 1.451982499 |
| AC007040.6 | 0.112181319 | 0.67110791 |
| AC016745.3 | 0.105778243 | 0.67109763 |
| PRLR | 0.13512057 | 1.440655426 |
| AC145343.1 | 0.017594273 | 0.670861924 |
| IL3 | 0.135159144 | 2.368008276 |
| RAPGEF4-AS1 | 0.077786601 | 0.670329137 |
| PSMA1 | 0.135285509 | 0.662558233 |
| ADCK3 | 0.135300897 | 0.833672981 |
| DLX2 | 0.135350885 | 4.350424441 |
| SRP72P2 | 0.022159836 | 0.669553454 |
| RP11-44N11.3 | 0.034286529 | 0.669497565 |
| TRAV12-2 | 0.12656871 | 0.669230973 |
| AC093388.3 | 0.113265017 | 0.669195342 |
| HMGN2P32 | 0.025798092 | 0.669075988 |
| RP11-573D15.8 | 0.07716066 | 0.668759661 |
| SFXN3 | 0.135390002 | 1.125781811 |
| RP1-102K2.9 | 0.04087042 | 0.668620281 |
| ANGPTL2 | 0.135596607 | 1.222304712 |
| AC009505.2 | 0.137579741 | 0.668310331 |
| ANKRD55 | 0.135622729 | 0.723581109 |
| PPM1N | 0.135672317 | 0.711398232 |
| SUCLA2P1 | 0.092885021 | 0.667128837 |
| CTC-251D13.1 | 0.040981132 | 0.666745953 |
| APOD | 0.135708847 | 2.02011568 |
| AC092377.1 | 0.135443305 | 0.666521323 |
| COX7A1 | 0.135711377 | 0.71238238 |
| GALNTL6 | 0.135749005 | 2.391151281 |
| LINC01539 | 0.128599234 | 0.666155206 |
| MIR4653 | 0.039180064 | 0.666026351 |
| COA7 | 0.135801192 | 0.895600694 |
| DRD1 | 0.135805552 | 2.800104505 |
| BDP1 | 0.135828711 | 1.123471878 |
| AC007016.3 | 0.085023591 | 0.665440085 |
| SERF1B | 0.135898123 | 1.176766845 |
| CTD-2215E18.2 | 0.118925406 | 0.664958174 |
| FRMD8P1 | 0.138600894 | 0.66467216 |
| C7orf25 | 0.135906695 | 1.25188527 |
| SMURF1 | 0.135928283 | 0.888646224 |
| TMEM63B | 0.136053686 | 1.113417248 |
| RP11-797J4.1 | 0.081913071 | 0.663966897 |
| RP11-613M5.1 | 0.123771215 | 0.663464316 |
| CTB-186G2.1 | 0.099939764 | 0.662681408 |
| SLC15A1 | 0.136215514 | 1.418730088 |
| RP11-15F12.6 | 0.053864255 | 0.662466456 |
| FKBP1A | 0.136355275 | 0.887459983 |
| THEG | 0.136358308 | 1.850902264 |
| CYCSP52 | 0.125909008 | 0.661983538 |
| RP11-731J8.1 | 0.106677732 | 0.661874998 |
| TAAR1 | 0.136372951 | 50.31478361 |
| RP11-624D20.1 | 0.132477332 | 0.661587852 |
| GHDC | 0.136440002 | 1.128513372 |
| ZFYVE9P2 | 0.140458442 | 0.661212402 |
| E2F8 | 0.136499061 | 1.163552113 |
| RP11-132A1.4 | 0.05457849 | 0.661107573 |
| ORAI2 | 0.13652946 | 1.226163257 |
| ZNF320 | 0.13653312 | 1.273220415 |
| RP11-498C9.12 | 0.11903321 | 0.66088496 |
| ANKRD52 | 0.136556548 | 1.117439382 |
| HDAC8 | 0.13658214 | 1.088048585 |
| CTA-941F9.9 | 0.044738861 | 0.660562281 |
| RPL21P67 | 0.119156826 | 0.660416247 |
| RP4-612C19.1 | 0.092801183 | 0.660295807 |
| TRIM36 | 0.13658638 | 1.392603358 |
| RP11-649A18.3 | 0.018617067 | 0.65972932 |
| POLD2 | 0.136636996 | 0.876734134 |
| FABP5 | 0.136708577 | 0.717843604 |
| LRCH1 | 0.136709572 | 1.11251988 |
| VSTM4 | 0.136765464 | 0.591418483 |
| PTRHD1 | 0.136776048 | 0.886545125 |
| TLR6 | 0.13680493 | 0.800450863 |
| METTL8P1 | 0.134699543 | 0.658090845 |
| RP11-182J1.1 | 0.059905482 | 0.658085234 |
| GPR128 | 0.13685348 | 6.95711798 |
| RN7SL684P | 0.023243288 | 0.657485378 |
| AP001962.3 | 0.108014894 | 0.657479098 |
| RP1-90L6.3 | 0.14389742 | 0.657417535 |
| ZFP62 | 0.136863293 | 1.114742231 |
| C6orf58 | 0.136865825 | 1.390141387 |
| CTD-2528A14.5 | 0.041613515 | 0.656198228 |
| ASB15 | 0.137081627 | 2.012079337 |
| DACH1 | 0.137095868 | 1.5284775 |
| EIF4G1 | 0.137202939 | 0.908012675 |
| AC105399.2 | 0.148512419 | 0.654728323 |
| MBIP | 0.1373026 | 1.152712501 |
| RP11-672A2.6 | 0.080205959 | 0.654487615 |
| CTB-58E17.9 | 0.085295532 | 0.65400876 |
| RP11-191L9.4 | 0.107745023 | 0.653872701 |
| BAI3 | 0.13738366 | 1.786409309 |
| RP11-1006G14.2 | 0.0683983 | 0.653584603 |
| CH17-360D5.3 | 0.049407148 | 0.653421521 |
| RNY3P16 | 0.104097715 | 0.65281374 |
| RNU6-554P | 0.032516289 | 0.652406825 |
| RP11-234K24.3 | 0.098003894 | 0.652120187 |
| ERICH2 | 0.137393691 | 1.462151252 |
| RP11-327F22.4 | 0.125976486 | 0.651858476 |
| GTF2F2 | 0.137398486 | 1.090518143 |
| ACOX3 | 0.137416132 | 0.860984296 |
| AC013404.1 | 0.091758236 | 0.65044923 |
| CTD-2547E10.3 | 0.017762335 | 0.650085536 |
| ANGPTL5 | 0.137489758 | 0.416647099 |
| RP11-386I14.2 | 0.05811173 | 0.649814104 |
| RP11-327F22.6 | 0.032492945 | 0.649774885 |
| VNN3 | 0.137559412 | 1.705987101 |
| CTD-2619J13.13 | 0.022098623 | 0.649354327 |
| RP11-798K3.3 | 0.135684663 | 0.649086109 |
| C5orf66-AS1 | 0.095184681 | 0.648545279 |
| AC012370.3 | 0.128068262 | 0.648453676 |
| RP3-347M6.2 | 0.109388765 | 0.648405376 |
| PCCB | 0.137559575 | 0.89356246 |
| RP11-467K18.2 | 0.075796888 | 0.647562536 |
| RP11-44F14.10 | 0.040094211 | 0.647269663 |
| RP4-777L9.2 | 0.026150887 | 0.646832708 |
| SIGLEC22P | 0.119509842 | 0.646469949 |
| RP4-781K5.6 | 0.137802855 | 0.646289487 |
| ST8SIA3 | 0.137630199 | 0.048097308 |
| AC005578.3 | 0.01984196 | 0.645870523 |
| PRSS8 | 0.137729104 | 1.213443246 |
| DGKD | 0.1377628 | 1.184177658 |
| RN7SL329P | 0.138656418 | 0.645037893 |
| CCDC112 | 0.137790239 | 1.163213258 |
| FAM83F | 0.137801551 | 0.825434287 |
| GBP7 | 0.137931432 | 1.721671468 |
| COL4A3BP | 0.138001937 | 1.118902757 |
| AVPR2 | 0.13800944 | 0.249842968 |
| NDNF | 0.138040708 | 1.998493911 |
| RP5-1056H1.2 | 0.032522074 | 0.643807985 |
| ABCA17P | 0.011301444 | 0.643638959 |
| RP11-927P21.4 | 0.026316699 | 0.642823896 |
| AC019109.1 | 0.146223041 | 0.642687524 |
| LCE3D | 0.138075754 | 0.276145571 |
| RPL12P41 | 0.092752369 | 0.641851836 |
| ID4 | 0.138097704 | 1.356467068 |
| NPW | 0.138103279 | 0.507398676 |
| RP11-431N15.2 | 0.144375281 | 0.64128253 |
| RP5-1068H6.6 | 0.070841554 | 0.641235435 |
| RP1-202O8.2 | 0.09254766 | 0.640953099 |
| TDRD5 | 0.13818087 | 0.668627166 |
| TXNP1 | 0.087505149 | 0.640652399 |
| RP4-550H1.4 | 0.136730136 | 0.640464647 |
| IL20RB-AS1 | 0.053961782 | 0.639991197 |
| OTOS | 0.138188024 | 44.36482269 |
| C9orf116 | 0.138192759 | 1.366547536 |
| KLHL1 | 0.138193918 | 2.560216736 |
| CTD-2194D22.3 | 0.10898476 | 0.639235288 |
| KRTAP4-7 | 0.138299585 | 16.71980635 |
| RP11-35G22.1 | 0.076283146 | 0.638935734 |
| C11orf73 | 0.138350764 | 0.889544362 |
| NRN1L | 0.13844487 | 1.40154698 |
| RP11-496H15.2 | 0.143806026 | 0.638487189 |
| SUMO2P8 | 0.132688572 | 0.638374428 |
| RP11-339A7.1 | 0.087958078 | 0.638125553 |
| IRF1 | 0.138448567 | 1.181608958 |
| ENPP7P12 | 0.095025794 | 0.636797412 |
| RP11-757F18.3 | 0.023271114 | 0.636766698 |
| RASA4DP | 0.020338028 | 0.636722449 |
| LINC01389 | 0.044708439 | 0.636417745 |
| LOXL2 | 0.138536696 | 1.439631054 |
| ACOX2 | 0.13865526 | 1.478435413 |
| RP11-470P21.2 | 0.085316703 | 0.636068819 |
| RP11-546O6.4 | 0.108732821 | 0.635766994 |
| ASCL5 | 0.138738273 | 2.551795895 |
| CTD-2308L22.1 | 0.083148064 | 0.635310503 |
| RP1-111C20.3 | 0.13621633 | 0.634989357 |
| Z69720.3 | 0.082270046 | 0.63463351 |
| RP11-203I2.1 | 0.049230447 | 0.634550787 |
| RPL35P6 | 0.128216863 | 0.634477746 |
| SEC24B | 0.138758441 | 1.095906044 |
| PROX2 | 0.138774629 | 1.249039996 |
| LGR6 | 0.138846488 | 1.562907049 |
| KB-1184D12.1 | 0.124608728 | 0.633765854 |
| SYT10 | 0.138890246 | 3.523387586 |
| FKSG66 | 0.138895369 | 1.55666625 |
| RP11-313A24.1 | 0.031485716 | 0.633159352 |
| Y_RNA | 0.033238951 | 0.632584026 |
| FLJ33360 | 0.101585044 | 0.632253242 |
| IYD | 0.138926291 | 2.125424917 |
| RP11-27M24.2 | 0.121808053 | 0.632135437 |
| AC098828.2 | 0.070566392 | 0.632114902 |
| TRBV30 | 0.150992043 | 0.632053104 |
| RP11-3B7.1 | 0.019537869 | 0.631783979 |
| RP11-307I14.2 | 0.067705619 | 0.631650237 |
| AC007563.1 | 0.082894328 | 0.631447945 |
| SNORD62A | 0.059968901 | 0.631154416 |
| TBCEL | 0.138966469 | 1.124339616 |
| PCAT19 | 0.118344043 | 0.630843525 |
| RP11-408A13.3 | 0.126158746 | 0.630618938 |
| GNLY | 0.138979109 | 1.414615007 |
| TSACC | 0.138986328 | 0.846311685 |
| RP4-607I7.1 | 0.016657009 | 0.630264168 |
| AC156455.1 | 0.088961625 | 0.629837852 |
| AC097495.2 | 0.099343534 | 0.629836526 |
| CKS1BP6 | 0.056967107 | 0.629660129 |
| LINC01451 | 0.051370159 | 0.629605157 |
| RNU6-304P | 0.114327626 | 0.629557608 |
| PHLPP2 | 0.13904744 | 1.171491081 |
| GALNT13 | 0.139079961 | 0.537807188 |
| RP3-324O17.8 | 0.069549769 | 0.628867715 |
| CTD-2129N1.1 | 0.023687097 | 0.628680868 |
| AGER | 0.139102124 | 1.154587168 |
| RP11-91I20.2 | 0.087751896 | 0.628252501 |
| RP11-411K7.1 | 0.039782602 | 0.628190437 |
| CTD-2659N19.4 | 0.148740995 | 0.628060934 |
| OTUD3 | 0.139117398 | 1.149320106 |
| RN7SL395P | 0.070415967 | 0.627849906 |
| SPOP | 0.139182035 | 1.075937331 |
| LINC00238 | 0.078828358 | 0.627025956 |
| RP11-44F14.2 | 0.018071786 | 0.626899644 |
| AP001055.6 | 0.021384316 | 0.626882388 |
| RP11-426L16.9 | 0.146400146 | 0.6265431 |
| AC137695.1 | 0.06360833 | 0.626526216 |
| RP11-402G3.4 | 0.129407551 | 0.626337914 |
| RBFOX1 | 0.139201439 | 4.882918978 |
| RP11-830F9.5 | 0.146867231 | 0.625973024 |
| FLJ42393 | 0.124649755 | 0.625565222 |
| RNH1 | 0.139236974 | 0.918955926 |
| RP11-69M1.4 | 0.136485356 | 0.625094257 |
| DYNLL1P7 | 0.077286138 | 0.624393739 |
| GAS6-AS1 | 0.144763365 | 0.624328425 |
| RP11-768B22.2 | 0.132219097 | 0.624060396 |
| RNU6-892P | 0.101809535 | 0.623731881 |
| LINC01559 | 0.112300756 | 0.623633234 |
| TMEM191A | 0.004580517 | 0.621766832 |
| STK33 | 0.139307594 | 1.649831416 |
| AF127577.13 | 0.138478843 | 0.620912327 |
| TRIM45 | 0.139391247 | 1.149034999 |
| RP11-416N2.4 | 0.059332113 | 0.620288474 |
| CTB-187L3.1 | 0.083019944 | 0.61998383 |
| RP11-386I14.3 | 0.069513523 | 0.619961062 |
| AGBL5-AS1 | 0.065130069 | 0.619788359 |
| RP11-18H21.1 | 0.030639642 | 0.619737618 |
| CENPBD1 | 0.139434911 | 0.847435767 |
| CTD-2017C7.3 | 0.125990179 | 0.618626984 |
| CTA-398F10.2 | 0.111515162 | 0.618584219 |
| RP11-616M22.5 | 0.113540865 | 0.618199854 |
| TINAGL1 | 0.139443576 | 1.225507222 |
| MRPS17P1 | 0.091442298 | 0.617904385 |
| CTC-265F19.1 | 0.128170752 | 0.617363149 |
| RP11-547M24.1 | 0.073273549 | 0.617262801 |
| RP11-255G12.2 | 0.11086305 | 0.616487144 |
| CTB-49A3.2 | 0.087561794 | 0.61638978 |
| IGKV3OR2-268 | 0.124960634 | 0.616311587 |
| HMGB3P10 | 0.068286619 | 0.615535863 |
| RP11-159F24.6 | 0.051735342 | 0.615119492 |
| YAP1P1 | 0.085951024 | 0.614575492 |
| AC107218.3 | 0.147443059 | 0.614551692 |
| SAPCD2P3 | 0.04973239 | 0.61445879 |
| TRIAP1 | 0.139475541 | 0.935371846 |
| MYC | 0.139492617 | 0.701057847 |
| RP11-21C4.1 | 0.076839676 | 0.614218742 |
| RP11-417L19.2 | 0.142411759 | 0.614128221 |
| RP11-5A11.1 | 0.101304702 | 0.613032343 |
| RP11-5P18.10 | 0.041387784 | 0.613028649 |
| SSSCA1 | 0.139549079 | 0.849953866 |
| CES5A | 0.139571134 | 0.157954742 |
| WNT5A-AS1 | 0.083833532 | 0.612231154 |
| AP001058.3 | 0.114948751 | 0.612102506 |
| KDELR1 | 0.139631728 | 1.098711378 |
| LIN37 | 0.13964723 | 0.894657976 |
| ADRA2B | 0.139744377 | 0.633882867 |
| Y_RNA | 0.098998887 | 0.611110641 |
| AC004813.1 | 0.13987252 | 0.243251377 |
| AC027319.1 | 0.05185366 | 0.610890446 |
| HMGB1P39 | 0.038828538 | 0.610699813 |
| RP11-455F5.3 | 0.10697556 | 0.610672041 |
| RNA5SP288 | 0.108177266 | 0.610103078 |
| DSTNP4 | 0.124823166 | 0.609956678 |
| U51244.2 | 0.076763322 | 0.60992503 |
| S100PBP | 0.139931659 | 1.082031292 |
| MAP9 | 0.139981507 | 1.493293951 |
| RP11-259O2.1 | 0.129033279 | 0.608302454 |
| RPS21 | 0.140000201 | 0.734951479 |
| HSPD1P3 | 0.132974284 | 0.607648088 |
| CTSL | 0.140097488 | 1.231738688 |
| MRPL37 | 0.140261403 | 0.907083008 |
| MIR6747 | 0.127983295 | 0.607304066 |
| USP47 | 0.140395025 | 1.076069578 |
| SDCCAG3P1 | 0.046379021 | 0.606573728 |
| SOD2 | 0.14039529 | 1.206513507 |
| RP11-408I18.9 | 0.111699322 | 0.606372845 |
| CDC37L1-AS1 | 0.116645364 | 0.606185722 |
| AC006262.5 | 0.116982205 | 0.605901121 |
| TRAPPC2B | 0.140403413 | 0.826349898 |
| MYRF | 0.140493978 | 1.482035363 |
| RP11-443C10.1 | 0.056055456 | 0.605790342 |
| ATP5F1P6 | 0.130192525 | 0.605678311 |
| SYNDIG1 | 0.140518427 | 1.679920316 |
| PDLIM2 | 0.140535255 | 0.838603597 |
| Y_RNA | 0.139753096 | 0.60495852 |
| RP11-319E16.1 | 0.088586161 | 0.604911765 |
| IGKV3-15 | 0.096333022 | 0.604763045 |
| RIOK3 | 0.140677623 | 0.899783968 |
| RP11-589N15.2 | 0.027886725 | 0.604639749 |
| TCEB2P2 | 0.146809999 | 0.604572991 |
| Y_RNA | 0.10973367 | 0.604425518 |
| MNX1-AS1 | 0.040955293 | 0.604287606 |
| RP1-278O22.1 | 0.028438528 | 0.603819558 |
| RP11-451F14.1 | 0.12336172 | 0.603675073 |
| OSTN-AS1 | 0.133067817 | 0.603620563 |
| KLF16 | 0.140687623 | 0.884703457 |
| CTB-52I2.4 | 0.002979316 | 0.603240278 |
| PLCH2 | 0.140764063 | 0.804593429 |
| LA16c-60H5.7 | 0.129932829 | 0.602687671 |
| CTD-2215E18.3 | 0.104038709 | 0.602285349 |
| HSPA8P1 | 0.006889094 | 0.602048366 |
| LLNLR-284B4.2 | 0.114344857 | 0.601710525 |
| RSF1 | 0.140796787 | 1.087335257 |
| RP11-1228E12.2 | 0.148136563 | 0.601490273 |
| GJB1 | 0.140904554 | 1.843435255 |
| LRRC8E | 0.141032827 | 0.759478027 |
| RP11-332O19.2 | 0.060863709 | 0.600875537 |
| PRKY | 0.14590611 | 0.600673052 |
| GOLGA8N | 0.141072773 | 1.233727527 |
| RP11-569G9.7 | 0.039980053 | 0.599550072 |
| TPH1 | 0.141128752 | 5.276051976 |
| AC007193.6 | 0.143382854 | 0.599425884 |
| NALCN-AS1 | 0.085032898 | 0.599299912 |
| RP11-244K5.6 | 0.118899889 | 0.599288612 |
| AL118506.2 | 0.049425182 | 0.599052633 |
| RNU6-1161P | 0.009910766 | 0.598808312 |
| GSDMB | 0.141132418 | 1.22959864 |
| ATP6V1E1P1 | 0.051610368 | 0.598611389 |
| PGAP1 | 0.141157252 | 0.833905907 |
| KLC1 | 0.141178822 | 0.919614577 |
| RP4-655L22.4 | 0.072769629 | 0.598212079 |
| RP11-1129I3.1 | 0.084333403 | 0.598074689 |
| PSMD7P1 | 0.087855514 | 0.597621697 |
| TM4SF19-AS1 | 0.018243162 | 0.597383917 |
| CACFD1 | 0.141218326 | 1.171890856 |
| RP11-573D15.3 | 0.103465691 | 0.596560771 |
| LINC01269 | 0.064516529 | 0.596115655 |
| AC105760.3 | 0.126064626 | 0.595952853 |
| RN7SL48P | 0.131933634 | 0.595901589 |
| NPFFR2 | 0.141287783 | 6.100477698 |
| C17orf99 | 0.141292465 | 0.464860378 |
| SLC39A3 | 0.141326159 | 0.882872126 |
| AL133168.3 | 0.077376654 | 0.594011697 |
| RP3-395M20.2 | 0.031159137 | 0.59365145 |
| RAC1P4 | 0.00903084 | 0.593589037 |
| PDK1 | 0.141328849 | 0.876299456 |
| RP11-216L13.21 | 0.01156239 | 0.593371936 |
| AC010492.5 | 0.110007378 | 0.593121738 |
| RP11-44F14.9 | 0.016652165 | 0.593095188 |
| CDIPT-AS1 | 0.077743037 | 0.593039793 |
| PAPL | 0.141365327 | 1.549985904 |
| SNRPBP1 | 0.117996397 | 0.592431663 |
| GPR50 | 0.141414942 | 5.2088675 |
| LECT1 | 0.141434423 | 5.124083445 |
| RP11-44N11.2 | 0.110251314 | 0.592249774 |
| RP11-116D17.5 | 0.146306791 | 0.591972031 |
| RP11-387A1.6 | 0.046310862 | 0.59188891 |
| MYOG | 0.141459 | 0.211591241 |
| RP11-571F15.2 | 0.041750728 | 0.591352319 |
| RP5-940F7.2 | 0.113660925 | 0.590808336 |
| MTND6P21 | 0.114629576 | 0.590774013 |
| SIGLEC5 | 0.141467502 | 0.791769998 |
| RP11-626H12.2 | 0.102530695 | 0.590546242 |
| MAGEA5 | 0.141370093 | 0.590452465 |
| RP11-15E18.5 | 0.06796293 | 0.590059242 |
| AC003006.1 | 0.031511219 | 0.589562956 |
| IGHV3-22 | 0.144267459 | 0.589530383 |
| RHO | 0.141532492 | 1.392504983 |
| TBC1D8B | 0.141536497 | 1.163386498 |
| TOM1 | 0.141696007 | 0.915609856 |
| RP11-211C9.1 | 0.142292415 | 0.588124082 |
| AC019129.2 | 0.054829454 | 0.587793241 |
| EIF5P1 | 0.037486332 | 0.587751244 |
| RP11-252K23.1 | 0.044448778 | 0.587311385 |
| RP11-403P17.5 | 0.141793763 | 1.697906134 |
| RP11-524L6.2 | 0.126345325 | 0.587115865 |
| MTIF3 | 0.141804846 | 1.100369746 |
| CHKB-AS1 | 0.113568502 | 0.58592382 |
| AC022098.1 | 0.048473722 | 0.585293858 |
| ADIPOR1P2 | 0.14962827 | 0.584866093 |
| CTB-127C13.1 | 0.147255997 | 0.584796236 |
| CTD-2523D13.1 | 0.083845309 | 0.584697311 |
| TRAV6 | 0.075871289 | 0.584069351 |
| RP4-701O16.5 | 0.015279288 | 0.584037524 |
| AC000110.1 | 0.139368286 | 0.583415784 |
| RP11-67L3.2 | 0.063334343 | 0.583384027 |
| snoU13 | 0.089283874 | 0.583088125 |
| RP11-113D6.10 | 0.130512548 | 0.582337804 |
| PIH1D3 | 0.141847954 | 2.726459036 |
| AC016716.1 | 0.029473307 | 0.581449195 |
| AC004691.1 | 0.082667915 | 0.580998679 |
| MLIP-IT1 | 0.016175527 | 0.580968897 |
| AP001065.7 | 0.118932697 | 0.580412449 |
| LINC00384 | 0.110963381 | 0.580018509 |
| RP11-348J12.5 | 0.095909835 | 0.579522718 |
| RP11-81H14.2 | 0.143410444 | 0.579397905 |
| TMEM261P1 | 0.10392433 | 0.57866264 |
| RP11-310E22.4 | 0.147391433 | 0.578081733 |
| CXCL10 | 0.141856784 | 1.520657855 |
| Y_RNA | 0.097323566 | 0.577608219 |
| RP11-60E8.4 | 0.143079474 | 0.577540705 |
| TINCR | 0.013290537 | 0.577257033 |
| MIRLET7F1 | 0.082136425 | 0.57699748 |
| DSG1-AS1 | 0.084028377 | 0.57688548 |
| FAM83C-AS1 | 0.0002835 | 0.576717359 |
| RP11-24M17.6 | 0.076793564 | 0.576531565 |
| TMCC3 | 0.141955159 | 0.823884708 |
| PRKAA1 | 0.142052391 | 1.12425416 |
| UFC1 | 0.142069939 | 1.086736318 |
| KRT8P34 | 0.117996198 | 0.576050755 |
| RNA5SP425 | 0.068863054 | 0.576007078 |
| RP11-546M21.6 | 0.044539094 | 0.576004305 |
| RP11-33I11.3 | 0.087862611 | 0.575587641 |
| OCA2 | 0.142070385 | 0.487416517 |
| VN1R80P | 0.017488441 | 0.574643642 |
| RP11-153F1.1 | 0.126909 | 0.574462175 |
| HIST2H3DP1 | 0.140538468 | 0.574073874 |
| MT-TV | 0.137592572 | 0.573811756 |
| RP11-10J18.3 | 0.034287148 | 0.57375011 |
| Metazoa_SRP | 0.132697454 | 0.573597648 |
| HSPA8P8 | 0.055249544 | 0.573488121 |
| RP11-254F7.1 | 0.136607748 | 0.573199785 |
| AC073869.1 | 0.095148292 | 0.573161095 |
| GMNC | 0.142087617 | 6.809540655 |
| PRRX2-AS1 | 0.026312096 | 0.572484864 |
| CTD-2384B9.1 | 0.126343094 | 0.572323665 |
| RP11-761N21.1 | 0.107295319 | 0.571946175 |
| GALNT14 | 0.142104755 | 0.782118023 |
| HIST2H3C | 0.142171106 | 0.557670193 |
| AADACL2 | 0.142184165 | 0.115908924 |
| NOL4 | 0.142250611 | 2.211123101 |
| CTC-458A3.1 | 0.02751672 | 0.57065757 |
| RPLP2P1 | 0.042464344 | 0.570479085 |
| C5orf56 | 0.14228752 | 1.187497015 |
| BANF1P2 | 0.104736263 | 0.570132541 |
| LINC00652 | 0.039789553 | 0.569493768 |
| IGKC | 0.081477592 | 0.569249323 |
| CTD-2501E16.2 | 0.0091227 | 0.569170364 |
| CTB-33O18.2 | 0.069380566 | 0.566852281 |
| SLC16A8 | 0.1423572 | 0.798302867 |
| RP11-326L2.1 | 0.035659672 | 0.566555937 |
| AC007278.2 | 0.129395382 | 0.566002508 |
| SUCNR1 | 0.142366849 | 4.348946616 |
| RP11-220I1.2 | 0.009972786 | 0.565697794 |
| FAM154A | 0.142519771 | 1.279128799 |
| FLG2 | 0.142572928 | 0.497206055 |
| TM6SF1 | 0.142576439 | 0.778060831 |
| AC104024.1 | 0.046036281 | 0.564094196 |
| RP11-667K14.8 | 0.043611008 | 0.563759803 |
| SND1-IT1 | 0.026171401 | 0.563753109 |
| B4GALT4-AS1 | 0.017130217 | 0.563726212 |
| RP4-686C3.7 | 0.12295403 | 0.563665252 |
| CTC-457L16.1 | 0.127126667 | 0.563355935 |
| SDCBP | 0.142591345 | 1.118785564 |
| IGLC3 | 0.093333115 | 0.563316455 |
| KNOP1P4 | 0.120002201 | 0.563206554 |
| RP3-477O4.5 | 0.083196687 | 0.562995167 |
| SF3A2 | 0.142603521 | 0.90821637 |
| RP11-414H23.2 | 0.146539764 | 0.562775236 |
| RP11-481J2.3 | 0.112676887 | 0.562456827 |
| KCTD9P1 | 0.144281582 | 0.562343021 |
| EIF4A2P1 | 0.060270356 | 0.562124767 |
| RPL7P14 | 0.058982017 | 0.562102127 |
| ARTN | 0.142635244 | 0.687353256 |
| MYO5BP2 | 0.067057528 | 0.561929959 |
| RP11-297B17.3 | 0.111178558 | 0.561628421 |
| ASIP | 0.142654984 | 1.786876462 |
| ATP5BP1 | 0.072728016 | 0.560959118 |
| AC096579.13 | 0.035671813 | 0.560312043 |
| MYEF2 | 0.142713678 | 1.639246337 |
| RNF128 | 0.14275357 | 1.530422846 |
| RP11-126L15.4 | 0.046202392 | 0.559537685 |
| RSPH10B | 0.142800754 | 1.311720228 |
| RP11-245G13.2 | 0.04620628 | 0.559198203 |
| RN7SKP98 | 0.115380096 | 0.559017245 |
| RP11-3K16.1 | 0.033499192 | 0.558879478 |
| TMEM19 | 0.142902699 | 0.835218464 |
| IFNG-AS1 | 0.12313831 | 0.558307743 |
| RP11-211G23.2 | 0.060519829 | 0.558200704 |
| RPLP0P7 | 0.13611188 | 0.558081059 |
| PRPF18 | 0.143021281 | 1.078560921 |
| Metazoa_SRP | 0.128155794 | 0.557585599 |
| SNORA25 | 0.145949868 | 0.557327286 |
| RP11-265D19.6 | 0.07694701 | 0.556995379 |
| LINC00844 | 0.135938686 | 0.556779483 |
| LINC00029 | 0.150024039 | 0.556471969 |
| EPHA3 | 0.143038909 | 0.344880197 |
| PSAT1P1 | 0.147673745 | 0.555971964 |
| DGKH | 0.143111179 | 1.144390496 |
| RP6-99M1.1 | 0.149537204 | 0.555681881 |
| AC005086.3 | 0.068573838 | 0.555561129 |
| UQCRFS1P1 | 0.118203122 | 0.555275614 |
| DNAJC3 | 0.143133204 | 1.113490814 |
| AC131097.4 | 0.063163229 | 0.554487257 |
| EPS8L3 | 0.143181894 | 1.919012568 |
| SIGLEC20P | 0.121085816 | 0.554253445 |
| RNU6-167P | 0.080302388 | 0.553964037 |
| RNU4-42P | 0.077462248 | 0.553825662 |
| CTB-113P19.4 | 0.125050752 | 0.553813858 |
| DPPA2P3 | 0.095317509 | 0.553398019 |
| MRPS7 | 0.143252493 | 0.850960263 |
| TRAV26-2 | 0.138109093 | 0.552944973 |
| RP11-706C16.8 | 0.031853996 | 0.552745803 |
| RP11-334E6.3 | 0.121269197 | 0.552743697 |
| RNF10 | 0.143267946 | 1.058422155 |
| CALHM3 | 0.143345439 | 2.361839079 |
| IGHM | 0.123141027 | 0.551628015 |
| RP11-246E12.2 | 0.012833866 | 0.551498529 |
| RNU6-1077P | 0.124208277 | 0.550531788 |
| MKRN1 | 0.14349816 | 1.076946601 |
| HSPA5 | 0.143548919 | 1.089363788 |
| CTD-2104P17.1 | 0.116819522 | 0.54951839 |
| CTD-2315E11.1 | 0.124317468 | 0.54911809 |
| FANCB | 0.143599751 | 1.11630683 |
| RP11-493E3.3 | 0.113136621 | 0.548688955 |
| ABCG1 | 0.143639253 | 1.199629802 |
| AC007036.4 | 0.048984227 | 0.548376376 |
| FTH1P25 | 0.120654945 | 0.548278072 |
| AC079150.2 | 0.113653021 | 0.548096616 |
| TXNDC11 | 0.143691542 | 1.16047163 |
| ZNF736 | 0.143722631 | 1.320201837 |
| SAFB | 0.143744946 | 0.950094159 |
| AC008984.6 | 0.100767243 | 0.547462789 |
| RP11-626A5.2 | 0.130386643 | 0.547369138 |
| WNT9A | 0.143764473 | 0.824150743 |
| RP11-523O18.1 | 0.119180343 | 0.546523031 |
| PPP1R11P1 | 0.117098947 | 0.546373183 |
| IGLV3-10 | 0.145380201 | 0.54624697 |
| CTB-79E8.2 | 0.098530401 | 0.546227101 |
| AC074366.3 | 0.077064595 | 0.545530479 |
| HES4 | 0.143800735 | 0.82591086 |
| RP11-557C18.4 | 0.091298409 | 0.544797079 |
| RP11-46I1.1 | 0.117885463 | 0.544609425 |
| CTC-265F19.3 | 0.123359124 | 0.543748404 |
| IGHV3-74 | 0.139533054 | 0.543530451 |
| C4orf33 | 0.143858891 | 1.131804664 |
| Y_RNA | 0.13094077 | 0.543353216 |
| C20orf96 | 0.143908028 | 1.188186322 |
| RP11-603B24.1 | 0.122278464 | 0.542818934 |
| RP11-159J3.1 | 0.025682302 | 0.542558163 |
| RABGAP1L-IT1 | 0.042586428 | 0.542548713 |
| RP4-631H13.4 | 0.146010251 | 0.542453446 |
| ADAM21P1 | 0.057096976 | 0.541952934 |
| IGLV3-31 | 0.14242399 | 0.54162229 |
| MYF5 | 0.143914413 | 6.438517682 |
| IRS2 | 0.14398224 | 1.279560326 |
| AC018755.16 | 0.02458599 | 0.541115054 |
| LARS2-AS1 | 0.063236959 | 0.540999614 |
| RP11-108P20.2 | 0.042318704 | 0.540806815 |
| RP11-328J14.2 | 0.121691946 | 0.540490622 |
| KCNS3 | 0.144108303 | 0.766100562 |
| IGHG1 | 0.092335358 | 0.539911743 |
| RP11-342M21.2 | 0.087853488 | 0.539668535 |
| RP11-545A16.3 | 0.095290873 | 0.539368969 |
| RP11-486B10.4 | 0.012341497 | 0.539339645 |
| YBX1P8 | 0.101353691 | 0.538638933 |
| DKFZp434J0226 | 0.054134279 | 0.538634368 |
| SMAD5-AS1 | 0.036805165 | 0.538191523 |
| VN2R19P | 0.095024513 | 0.538186679 |
| TVP23CP1 | 0.049926413 | 0.537917844 |
| ADAM15 | 0.144174784 | 0.888006485 |
| RP11-436I9.3 | 0.091912527 | 0.537473103 |
| ARHGEF10L | 0.144216775 | 0.861696696 |
| CHD1 | 0.144253006 | 1.091954137 |
| DYNLRB1 | 0.144264134 | 0.88022296 |
| RP3-333B15.5 | 0.081862424 | 0.535794506 |
| FAM129C | 0.144297445 | 0.729308014 |
| Y_RNA | 0.101156695 | 0.535099142 |
| RP11-630H24.4 | 0.080776851 | 0.534819849 |
| CTD-2008L17.1 | 0.138805073 | 0.534759953 |
| SLC25A5P5 | 0.060825481 | 0.533077344 |
| RN7SKP116 | 0.008256616 | 0.532935911 |
| SMARCE1P6 | 0.026989096 | 0.532218484 |
| GUCA1C | 0.144326795 | 4.974474274 |
| SALRNA3 | 0.107796282 | 0.531081422 |
| GNRHR2P1 | 0.036524574 | 0.530734891 |
| RP11-274H24.1 | 0.051728661 | 0.530589111 |
| RP11-363E6.3 | 0.013442646 | 0.529827437 |
| C15orf48 | 0.144349664 | 1.309823475 |
| CACNA1D | 0.144356896 | 1.401907757 |
| SYP-AS1 | 0.032495437 | 0.529613045 |
| BOLA3P4 | 0.14205783 | 0.529108454 |
| COX6B1P1 | 0.057691987 | 0.528959829 |
| RP11-342M1.6 | 0.067369421 | 0.528911949 |
| ZNF683 | 0.144368939 | 1.424697146 |
| HAR1B | 0.138644002 | 0.528628744 |
| PIN1 | 0.144384447 | 0.896140034 |
| SNRPGP18 | 0.125494878 | 0.527684788 |
| ZNF566 | 0.144418753 | 0.840110743 |
| RNF175 | 0.144438392 | 1.252046716 |
| MIR5187 | 0.078939482 | 0.526444162 |
| RP11-314A15.2 | 0.091001198 | 0.526378691 |
| GGCX | 0.144480869 | 0.917422451 |
| C6orf10 | 0.144549189 | 2.605932563 |
| IGLVIVOR22-1 | 0.112967238 | 0.525629871 |
| PPY | 0.144553201 | 0.621315981 |
| CACNA1G | 0.144584532 | 0.717896629 |
| RP11-66N5.2 | 0.063951498 | 0.52473971 |
| DDX3P3 | 0.112747912 | 0.524095627 |
| LINC01378 | 0.146616032 | 0.524037938 |
| RP11-535A19.1 | 0.116117741 | 0.523915157 |
| SNORA36B | 0.038100396 | 0.523729626 |
| MIR4506 | 0.092226195 | 0.523727423 |
| RP11-73G16.2 | 0.091302096 | 0.523653582 |
| RP11-334E6.2 | 0.134168074 | 0.523625769 |
| RP11-554D14.2 | 0.14163461 | 0.523607246 |
| LINC00605 | 0.11428298 | 0.523584457 |
| AL360091.1 | 0.048359291 | 0.521912627 |
| CTD-2066L21.3 | 0.12291341 | 0.519652481 |
| DTYMK | 0.144621812 | 0.828679681 |
| SPRED1 | 0.144649764 | 1.124437014 |
| RP13-452N2.1 | 0.113998147 | 0.518955608 |
| KCNQ5-IT1 | 0.121279515 | 0.518597818 |
| AC018641.7 | 0.066313531 | 0.518068596 |
| RP11-12A2.1 | 0.037342153 | 0.517268386 |
| RP5-1037N22.2 | 0.075476445 | 0.516991036 |
| PES1P2 | 0.078329222 | 0.516538929 |
| AC004951.5 | 0.02076487 | 0.516355796 |
| RP11-321A17.4 | 0.064163761 | 0.516115327 |
| PAQR6 | 0.144754619 | 1.196352388 |
| AC084854.1 | 0.110268038 | 0.515952697 |
| SPTSSA | 0.144773443 | 1.113355617 |
| RP4-806M20.4 | 0.085206857 | 0.515375656 |
| RP1-144C9.2 | 0.102140402 | 0.515296768 |
| GXYLT1P6 | 0.083869446 | 0.515278461 |
| IL1RN | 0.144807226 | 0.732871959 |
| KIAA0125 | 0.14148552 | 0.514522524 |
| SPATA13-AS1 | 0.122611493 | 0.514026626 |
| LINC01154 | 0.041098876 | 0.514003559 |
| RP11-445P17.3 | 0.127654616 | 0.513909387 |
| RP11-400N13.3 | 0.097696009 | 0.513872816 |
| RFPL4AP6 | 0.031023086 | 0.513094975 |
| CECR9 | 0.140612422 | 0.512949814 |
| EPHB2 | 0.14485544 | 1.22803135 |
| RP5-1039K5.18 | 0.087134186 | 0.512652492 |
| RPL7P2 | 0.134537297 | 0.51253684 |
| LINC00176 | 0.035258504 | 0.512515526 |
| TAZ | 0.144869739 | 1.108434205 |
| RP11-185E8.1 | 0.063808758 | 0.512285403 |
| CCND2P1 | 0.148767881 | 0.512251947 |
| RCC2P7 | 0.028389273 | 0.512017807 |
| MED21 | 0.144890141 | 1.090674053 |
| NDUFA5 | 0.144908498 | 1.100814754 |
| AC004257.1 | 0.053854405 | 0.510471428 |
| AC114765.2 | 0.059710809 | 0.509835349 |
| RP4-576H24.2 | 0.127254993 | 0.509810865 |
| PDXP | 0.144966965 | 0.813568107 |
| RP11-629B11.4 | 0.120361372 | 0.508179516 |
| RP11-404F10.6 | 0.073277567 | 0.507421313 |
| CRIPAK | 0.144980862 | 1.15492299 |
| RP4-570O12.2 | 0.127193096 | 0.506711454 |
| RP4-587D13.1 | 0.074383893 | 0.506181138 |
| RP4-806M20.3 | 0.136377011 | 0.506136251 |
| LGALS7B | 0.145200985 | 0.483453078 |
| RN7SL234P | 0.104930279 | 0.505047345 |
| MIR7976 | 0.090403325 | 0.504565808 |
| TMED9 | 0.145203292 | 1.077208119 |
| CTA-280A3.2 | 0.025461094 | 0.504172652 |
| CTC-526N19.1 | 0.000583479 | 0.504170615 |
| ATP6V1B1-AS1 | 0.012330819 | 0.503320372 |
| AC007386.2 | 0.03190888 | 0.502844739 |
| IGLV8OR8-1 | 0.142132838 | 0.502283107 |
| OR5P3 | 0.145245479 | 2.527694892 |
| LINC00964 | 0.060369749 | 0.500969536 |
| RP11-325O24.2 | 0.05711931 | 0.500709451 |
| RP11-264M12.4 | 0.136306492 | 0.500630323 |
| FNBP1L | 0.14534591 | 1.156564097 |
| RPL5P27 | 0.063257976 | 0.49949491 |
| RP11-498C9.17 | 0.068015568 | 0.499306903 |
| HDAC6 | 0.145365633 | 1.106280228 |
| RP11-501J20.3 | 0.116883146 | 0.49874108 |
| HAS3 | 0.145389527 | 0.6701436 |
| RP11-1102P22.2 | 0.045135521 | 0.498127555 |
| IGKV1-27 | 0.130180902 | 0.497927555 |
| RPL23AP4 | 0.115144087 | 0.497218916 |
| RPE65 | 0.14541329 | 0.118466213 |
| RP11-599B13.9 | 0.136975331 | 0.496776062 |
| PTCHD3P3 | 0.072611715 | 0.496162047 |
| AKIRIN1P2 | 0.033867581 | 0.496040431 |
| AC005237.4 | 0.025092597 | 0.495206594 |
| PCDHA8 | 0.1454164 | 2.093195553 |
| RNU6-16P | 0.065668285 | 0.494306054 |
| UBE2FP2 | 0.089077164 | 0.494282093 |
| LINC01586 | 0.025724255 | 0.494003507 |
| RP1-122P22.4 | 0.13860685 | 0.493948182 |
| RP11-439A17.10 | 0.068662014 | 0.493890068 |
| FMO9P | 0.052534788 | 0.493805996 |
| RP11-403A3.1 | 0.132101763 | 0.49342492 |
| RP11-1055B8.10 | 0.067433768 | 0.493235923 |
| RNU6-968P | 0.131867441 | 0.492970414 |
| TMBIM1 | 0.145453685 | 0.891120233 |
| AC103563.5 | 0.121075882 | 0.491708784 |
| CSE1L-AS1 | 0.059283877 | 0.491649822 |
| ZSWIM7 | 0.145483933 | 1.108557632 |
| Metazoa_SRP | 0.096086892 | 0.491332325 |
| IGHV4-39 | 0.129945618 | 0.491211338 |
| ITIH6 | 0.145714315 | 0.630527291 |
| RUVBL1-AS1 | 0.001350656 | 0.491069815 |
| AC013476.1 | 0.124006842 | 0.491047418 |
| ATP5HP3 | 0.090487403 | 0.490833761 |
| MILR1 | 0.145769145 | 1.223958616 |
| MIR4514 | 0.132333197 | 0.490603577 |
| SMPD4P1 | 0.106060672 | 0.489956723 |
| RP5-907D15.3 | 0.092187598 | 0.489740297 |
| IGHV3-65 | 0.067402196 | 0.489544826 |
| AP000525.10 | 0.129151845 | 0.489432352 |
| IGHV1-3 | 0.113117097 | 0.489377744 |
| RP11-370I10.4 | 0.018482681 | 0.48936064 |
| GULOP | 0.130514436 | 0.489174176 |
| CTB-189B5.3 | 0.003247835 | 0.489072559 |
| RNU6-230P | 0.070486238 | 0.488164281 |
| RP11-476B13.2 | 0.096088124 | 0.487790509 |
| HSPE1P13 | 0.10394859 | 0.487550275 |
| CLDN14 | 0.145791927 | 1.528214302 |
| RP11-475J5.10 | 0.080122258 | 0.487391962 |
| SIGLEC18P | 0.035889803 | 0.486970828 |
| LINC01010 | 0.056354102 | 0.486552494 |
| ABHD17AP6 | 0.09247067 | 0.486515616 |
| AC114812.10 | 0.138800819 | 0.48637297 |
| RNU4-40P | 0.032455879 | 0.485848978 |
| HOXB13 | 0.145792156 | 2.286507439 |
| HNRNPCL4 | 0.145840838 | 3.372098346 |
| CHCHD2P4 | 0.06604645 | 0.485000544 |
| SNRPCP1 | 0.100271997 | 0.484730063 |
| RP11-470L19.2 | 0.072131545 | 0.484249266 |
| RP11-560I19.1 | 0.100657516 | 0.483907817 |
| TMEM120B | 0.145860681 | 1.129912161 |
| DCT | 0.14588296 | 4.7325806 |
| C14orf2 | 0.145883751 | 0.881096248 |
| RN7SL127P | 0.119519237 | 0.482464333 |
| CSNK1G2P1 | 0.141289173 | 0.481181299 |
| JADE3 | 0.145969233 | 1.119711687 |
| AC007326.10 | 0.059254638 | 0.479527721 |
| LINC00824 | 0.123226342 | 0.479389503 |
| RNU1-22P | 0.08130809 | 0.479356324 |
| LGALS3BP | 0.145980504 | 1.138516332 |
| SPOCK1 | 0.146090478 | 1.412955003 |
| COPS7B | 0.146132465 | 1.090913803 |
| RP11-456O19.5 | 0.107912895 | 0.478612348 |
| TMEM173 | 0.146134897 | 1.152313446 |
| GLRA4 | 0.146175797 | 5.169395902 |
| RP11-740P5.3 | 0.100830906 | 0.477261847 |
| PGR | 0.146182752 | 1.775853781 |
| CPNE3 | 0.14628319 | 0.884316055 |
| SPATC1L | 0.146315539 | 0.811209118 |
| PLXNA3 | 0.146331413 | 1.16419268 |
| TRBV10-1 | 0.043428669 | 0.475081983 |
| PRKRIRP2 | 0.136697645 | 0.473856669 |
| RP1-140J1.1 | 0.039742794 | 0.473726986 |
| IGHV3-49 | 0.056293889 | 0.473511691 |
| IFNL4 | 0.042331636 | 0.473270766 |
| RN7SL187P | 0.13145408 | 0.472795666 |
| Metazoa_SRP | 0.103297435 | 0.472472356 |
| MIR365A | 0.065360314 | 0.472374577 |
| RP13-455A7.1 | 0.028690502 | 0.472286956 |
| MIR2355 | 0.092215001 | 0.471249868 |
| RP11-54I5.1 | 0.022125152 | 0.470311903 |
| RAPGEF3 | 0.146339209 | 1.256765161 |
| RP11-85O21.4 | 0.047642973 | 0.469952751 |
| FLYWCH1 | 0.146378274 | 0.877931126 |
| MIR7848 | 0.118247731 | 0.46970311 |
| IGKV1D-17 | 0.10538749 | 0.468324174 |
| IGLV2-8 | 0.113519318 | 0.467748779 |
| RN7SL353P | 0.07471725 | 0.467248407 |
| RN7SKP90 | 0.066926762 | 0.46700072 |
| snoU109 | 0.093697206 | 0.466675901 |
| RP11-344N17.8 | 0.076446123 | 0.466610583 |
| CNTFR-AS1 | 0.059769163 | 0.466443937 |
| CTC-463N11.1 | 0.062276778 | 0.466185076 |
| CTB-3M24.2 | 0.117561727 | 0.466117884 |
| AC107977.1 | 0.07033016 | 0.46598243 |
| TAS2R31 | 0.146535845 | 1.346218574 |
| IMPG1 | 0.146610294 | 1.324483502 |
| AHCYP4 | 0.077101925 | 0.464096528 |
| RP11-74K11.2 | 0.145335978 | 0.463668431 |
| TECRP2 | 0.079912269 | 0.463388683 |
| DDX18P6 | 0.126421091 | 0.462828018 |
| COX6B1P4 | 0.085310992 | 0.462752351 |
| ADCK2 | 0.146649409 | 0.900850272 |
| MIR6776 | 0.020929167 | 0.462564035 |
| KIAA1024L | 0.146709486 | 3.781036965 |
| ABHD17AP4 | 0.089104355 | 0.461889534 |
| RP4-813D12.3 | 0.044002786 | 0.461620062 |
| AC017028.7 | 0.105782375 | 0.461490647 |
| CTC-329D1.3 | 0.104175225 | 0.461337322 |
| PINLYP | 0.146764766 | 1.300625237 |
| AL606500.1 | 0.127835341 | 0.460205443 |
| RP11-50B3.1 | 0.119959718 | 0.459884121 |
| NETO2 | 0.146779555 | 0.869781311 |
| LINC00265-2P | 0.134766365 | 0.458391663 |
| DEFB136 | 0.146876868 | 5.271492343 |
| NIFKP8 | 0.060525774 | 0.458318839 |
| NUDCD1 | 0.146877881 | 1.106128032 |
| BEND3P2 | 0.126099625 | 0.458142326 |
| AC003688.1 | 0.02978394 | 0.458128694 |
| AL928768.3 | 0.077389304 | 0.457453705 |
| RP11-384C21.9 | 0.099136227 | 0.457053018 |
| RP11-77K12.4 | 0.144211319 | 0.455575396 |
| IGHV2-26 | 0.150075022 | 0.454883593 |
| RP3-340B19.5 | 0.132616147 | 0.45457506 |
| AC128709.3 | 0.096749103 | 0.454501895 |
| RP11-120C12.3 | 0.059760645 | 0.454093988 |
| RP1-90G24.10 | 0.124900087 | 0.454026435 |
| RP11-555J4.3 | 0.10234495 | 0.453989905 |
| CTC-559E9.2 | 0.121856947 | 0.45390734 |
| AF131217.1 | 0.059267976 | 0.453747414 |
| RP11-509A17.3 | 0.150871098 | 0.4533402 |
| RN7SKP273 | 0.076489297 | 0.453304203 |
| Y_RNA | 0.035782396 | 0.453171766 |
| NKIRAS1 | 0.146878237 | 1.106762175 |
| RNU6-346P | 0.098471938 | 0.452640543 |
| AC013251.1 | 0.118386507 | 0.452410479 |
| AC007050.18 | 0.098088841 | 0.451847086 |
| TRAJ8 | 0.134577868 | 0.45127444 |
| MOGAT1 | 0.146894598 | 1.712792054 |
| IGHV3-41 | 0.116365333 | 0.450958723 |
| CTD-2165H16.3 | 0.008936276 | 0.450739917 |
| RPS3AP9 | 0.116603076 | 0.450448215 |
| INSL6 | 0.146938503 | 5.569672038 |
| RP11-795H16.3 | 0.042951658 | 0.448755462 |
| RP11-136B18.2 | 0.145984192 | 0.448594045 |
| RP11-525E9.1 | 0.0516458 | 0.448173392 |
| CTD-2066L21.1 | 0.099317255 | 0.448103146 |
| FAM177A1P1 | 0.110820113 | 0.447588791 |
| RP11-74M11.2 | 0.107567094 | 0.447558196 |
| C1orf185 | 0.146964189 | 1.69497019 |
| GAPDHP1 | 0.064566143 | 0.447138186 |
| RP13-820C6.4 | 0.069388402 | 0.447045433 |
| SOX2-OT | 0.072762083 | 0.445538552 |
| RP4-604K5.2 | 0.054276217 | 0.444142042 |
| CGRRF1 | 0.146966918 | 1.110764028 |
| RP11-164N3.2 | 0.061439637 | 0.443678648 |
| RBBP4 | 0.147020691 | 1.087935588 |
| CH507-216K13.1 | 0.111438197 | 0.443395723 |
| MIR6510 | 0.020661484 | 0.443209913 |
| RP4-715N11.2 | 0.053429676 | 0.443162487 |
| RP11-276H19.4 | 0.01108038 | 0.442642804 |
| Y_RNA | 0.093352886 | 0.441159002 |
| RP11-384P7.7 | 0.09723025 | 0.44070629 |
| LINC01067 | 0.015503735 | 0.440609593 |
| HSPB11 | 0.147028028 | 1.103564104 |
| OPLAH | 0.147035044 | 0.835846962 |
| LINC01247 | 0.115852541 | 0.439885523 |
| NT5DC4 | 0.147258279 | 0.741517529 |
| MIR548AC | 0.019029575 | 0.439519599 |
| MAS1LP1 | 0.114061661 | 0.438790247 |
| RP11-296E23.2 | 0.124363307 | 0.437997684 |
| CTD-2006K23.1 | 0.147795509 | 0.43781554 |
| KRT127P | 0.082105601 | 0.437810848 |
| RP11-507F16.1 | 0.120465858 | 0.437762284 |
| Metazoa_SRP | 0.086968214 | 0.437203797 |
| IGLL5 | 0.147285432 | 0.630848947 |
| RP11-497E19.1 | 0.0841454 | 0.433840129 |
| RP11-268I9.1 | 0.014411576 | 0.433796645 |
| RP11-1137G4.3 | 0.047610234 | 0.433587642 |
| RNU6-770P | 0.055365601 | 0.433455775 |
| AC104076.2 | 0.081449325 | 0.433160897 |
| AF212831.2 | 0.089191017 | 0.432491233 |
| SPATA19 | 0.147304291 | 0.543365202 |
| TRAJ35 | 0.110971221 | 0.432077039 |
| YIPF2 | 0.147323848 | 1.188953144 |
| ME2P1 | 0.033718394 | 0.429574383 |
| MIR203A | 0.029168922 | 0.429530995 |
| SLC9A9-AS1 | 0.080009025 | 0.426993639 |
| RP11-39K24.12 | 0.136340834 | 0.426136105 |
| CYCSP2 | 0.081444889 | 0.425791649 |
| SHC4 | 0.147354691 | 1.406083164 |
| TUBB4BP7 | 0.09195283 | 0.425685008 |
| FAM76B | 0.147376568 | 1.152714105 |
| RP11-959F10.5 | 0.089516684 | 0.423721023 |
| RP11-96H17.3 | 0.118180255 | 0.423438087 |
| LINC01164 | 0.112681618 | 0.423230529 |
| GAPDHP24 | 0.041570004 | 0.422186508 |
| LINC01524 | 0.013786038 | 0.421631245 |
| SLC9A3P2 | 0.086989962 | 0.420749103 |
| RP11-125M16.1 | 0.140034719 | 0.420470097 |
| RN7SL116P | 0.062923553 | 0.420437601 |
| AC009276.4 | 0.079182056 | 0.420271629 |
| PLA2G2D | 0.14738092 | 0.293646081 |
| CTC-246B18.8 | 0.006756348 | 0.418695109 |
| MIEF1 | 0.147414631 | 0.918704799 |
| KCNAB3 | 0.14744392 | 1.22922255 |
| RNA5SP305 | 0.09139457 | 0.418546547 |
| SCAND3P1 | 0.055345995 | 0.418330121 |
| AC010423.1 | 0.039668269 | 0.417888446 |
| HMGN1P16 | 0.138155386 | 0.417657797 |
| GS1-72M22.1 | 0.087679378 | 0.41671297 |
| MAP3K10 | 0.147538064 | 0.827479778 |
| CTD-2587M23.1 | 0.145645491 | 0.416553369 |
| OR5BS1P | 0.14803696 | 0.416035485 |
| AK4P6 | 0.10074194 | 0.41550234 |
| ST13P12 | 0.052931472 | 0.41374318 |
| RP11-364C11.3 | 0.118943214 | 0.413698152 |
| AL136231.1 | 0.020503577 | 0.413403845 |
| RP11-370I10.2 | 0.04258894 | 0.413345463 |
| FAM187B | 0.147587448 | 0.670960163 |
| SNORA76A | 0.065435132 | 0.41219609 |
| RP11-513D4.1 | 0.032777366 | 0.412058487 |
| IGLV3-19 | 0.097942516 | 0.411737673 |
| LINC01018 | 0.031563357 | 0.41067186 |
| RP11-533E19.2 | 0.114629969 | 0.410480134 |
| RP11-556I14.2 | 0.036720679 | 0.410427592 |
| MRPS25 | 0.147756294 | 1.13380483 |
| RP11-561O23.9 | 0.055264012 | 0.410063609 |
| MIR125B2 | 0.074167922 | 0.409965642 |
| RXFP2 | 0.147777093 | 2.134315166 |
| BRK1 | 0.147788694 | 1.08455858 |
| ST13P20 | 0.016419029 | 0.408457498 |
| RP11-297M9.1 | 0.114234846 | 0.407519627 |
| RP11-438B23.2 | 0.042930296 | 0.407457202 |
| TMEM108-AS1 | 0.07869168 | 0.407418273 |
| NAA40 | 0.147800139 | 1.102603655 |
| RP11-475I24.9 | 0.064788752 | 0.406917462 |
| RP11-484N16.1 | 0.061199555 | 0.406207176 |
| PAGE2 | 0.147827913 | 24.10484486 |
| ACSM5P1 | 0.147378378 | 0.40597062 |
| RHBG | 0.147867756 | 0.704249233 |
| LINC00557 | 0.036345209 | 0.404393953 |
| HBP1 | 0.147870629 | 0.894142008 |
| RP11-663N22.1 | 0.150613704 | 0.403315584 |
| CUL9 | 0.147908854 | 0.873901217 |
| RP3-462D8.2 | 0.119863924 | 0.399798585 |
| AC007679.4 | 0.066186073 | 0.399658317 |
| RN7SL688P | 0.029217461 | 0.399526653 |
| MIR101-2 | 0.07549705 | 0.399198074 |
| AC009305.1 | 0.084616605 | 0.397012911 |
| RPL5P20 | 0.12384076 | 0.396961333 |
| GRHL1 | 0.147939347 | 0.794304265 |
| TMEM256P2 | 0.044364885 | 0.39598951 |
| RP11-275H4.1 | 0.144318728 | 0.395894439 |
| FTLP8 | 0.01221477 | 0.393702714 |
| RP4-777L9.3 | 0.007658356 | 0.391753855 |
| CASC19 | 0.125438304 | 0.390251055 |
| ACOT6 | 0.14797728 | 1.3849749 |
| RP11-574F11.3 | 0.041305507 | 0.389429352 |
| KIAA0930 | 0.148114489 | 0.888732943 |
| RP11-146E13.5 | 0.135479258 | 0.388685071 |
| RP11-624L4.2 | 0.064611893 | 0.386497824 |
| AP000473.6 | 0.074972584 | 0.386479954 |
| AC091729.7 | 0.089844412 | 0.38616987 |
| RP11-404P21.6 | 0.147694624 | 0.385669404 |
| OR6J1 | 0.148135853 | 2.761642938 |
| RNU6-742P | 0.09230374 | 0.385170771 |
| TRBV26OR9-2 | 0.043207579 | 0.384556489 |
| GDF5 | 0.148142601 | 1.719971792 |
| TPRG1-AS2 | 0.025856141 | 0.383691423 |
| ATP13A5-AS1 | 0.120125688 | 0.382704312 |
| RNA5SP320 | 0.15048697 | 0.382680436 |
| RNU6-103P | 0.039382148 | 0.382617612 |
| RP11-235P11.1 | 0.078336127 | 0.382562435 |
| CTB-138E5.1 | 0.100293257 | 0.381968228 |
| CITED4 | 0.148142861 | 1.594758234 |
| Y_RNA | 0.085008093 | 0.381742209 |
| PSPC1P1 | 0.043688478 | 0.379682545 |
| AL122127.25 | 0.103763043 | 0.379386305 |
| Metazoa_SRP | 0.121972043 | 0.377690537 |
| RP11-153F5.3 | 0.118973142 | 0.376232008 |
| SNORD75 | 0.07516388 | 0.376165859 |
| SLC26A4 | 0.148200755 | 2.211442268 |
| GPR141 | 0.148203627 | 1.494397355 |
| RNFT1P2 | 0.039019234 | 0.375676862 |
| RP4-669B10.3 | 0.122403927 | 0.375654674 |
| RNU6-291P | 0.077996707 | 0.374724129 |
| CD79B | 0.148278372 | 0.714980632 |
| ABCC13 | 0.130779543 | 0.374188667 |
| AC087499.7 | 0.09816049 | 0.373180279 |
| NPRL2 | 0.148292875 | 1.100184676 |
| AC073850.6 | 0.114958121 | 0.37275876 |
| CAPNS1P1 | 0.016253029 | 0.372249089 |
| SNORA9 | 0.057056006 | 0.371663273 |
| MIR4674 | 0.132908994 | 0.369964168 |
| RP11-973F15.2 | 0.150283936 | 0.369566973 |
| HP | 0.148395514 | 2.347463875 |
| Y_RNA | 0.082531324 | 0.369208735 |
| RP11-208N14.4 | 0.134632508 | 0.369161025 |
| MIR4446 | 0.125056392 | 0.369138265 |
| RP11-486B10.3 | 0.036481957 | 0.368909913 |
| LCE1E | 0.148559157 | 1.436271697 |
| RP11-182I10.2 | 0.056060906 | 0.366910869 |
| VTCN1 | 0.148590134 | 1.476904766 |
| RP11-346D14.1 | 0.062113148 | 0.36645089 |
| OR2A25 | 0.148650319 | 0.477630122 |
| XXbac-BPG248L24.13 | 0.144593725 | 0.366239621 |
| RP11-553E24.1 | 0.020955931 | 0.365729036 |
| RNA5SP30 | 0.143217988 | 0.36525952 |
| RP11-338L18.1 | 0.119880063 | 0.364068133 |
| GNAS-AS1 | 0.080493803 | 0.363908112 |
| RP11-155N3.4 | 0.107371828 | 0.363526843 |
| RP11-177F11.1 | 0.117924549 | 0.362460053 |
| TSNAXIP1 | 0.148700177 | 1.420050991 |
| IGHV7-40 | 0.089293231 | 0.360951923 |
| IGKV3-20 | 0.074027293 | 0.360933326 |
| LINC00898 | 0.087371238 | 0.360735979 |
| RN7SL371P | 0.09799469 | 0.360522757 |
| CYP4F10P | 0.149251596 | 0.360475111 |
| RP11-568G11.5 | 0.097199386 | 0.358943912 |
| AL158069.1 | 0.040060618 | 0.357914576 |
| TDRD15 | 0.148760906 | 0.527920307 |
| RP11-25P11.2 | 0.117119706 | 0.357884094 |
| B3GALNT2 | 0.148802256 | 0.859257207 |
| RP11-642D21.2 | 0.092338554 | 0.357406848 |
| TRAPPC13P1 | 0.084532926 | 0.356568548 |
| RP11-92C4.4 | 0.087967712 | 0.35650624 |
| FAM183A | 0.14882759 | 1.361976344 |
| PRAMENP | 0.026622476 | 0.355031672 |
| MIR627 | 0.146878564 | 0.354633494 |
| CTD-2272G21.2 | 0.101986297 | 0.352752344 |
| FYTTD1P1 | 0.072207641 | 0.352639013 |
| RP11-497E19.2 | 0.046506527 | 0.352248108 |
| RP11-164N3.1 | 0.017075655 | 0.351546545 |
| PTENP1-AS | 0.043495352 | 0.351088997 |
| PAQR9 | 0.148854822 | 0.410340211 |
| RNU6-1306P | 0.098127684 | 0.349628258 |
| ETV6 | 0.148908755 | 1.129002659 |
| HMGB3P15 | 0.101504829 | 0.349236586 |
| ATP5A1P7 | 0.093867499 | 0.348919733 |
| SLC9A9-AS2 | 0.117193434 | 0.348070433 |
| RP6-159A1.3 | 0.085121973 | 0.34783596 |
| CTC-448F2.7 | 0.02218641 | 0.347547931 |
| RP11-547C5.1 | 0.05599153 | 0.347403311 |
| RP11-292B8.2 | 0.072179079 | 0.346285453 |
| RP11-46B11.2 | 0.094142783 | 0.345400982 |
| ARGLU1 | 0.148981606 | 1.115139232 |
| IGHV3-21 | 0.097481029 | 0.342581925 |
| OR2C3 | 0.148983894 | 1.794285503 |
| CCDC151 | 0.149001786 | 1.58375544 |
| RP13-492C18.1 | 0.134228841 | 0.341614744 |
| PRKD3 | 0.149053179 | 1.112648498 |
| U3 | 0.106512291 | 0.340078416 |
| AKAP8P1 | 0.013417133 | 0.338752694 |
| RP11-473E2.3 | 0.095765816 | 0.338706009 |
| RP11-76E17.1 | 0.043202739 | 0.33868491 |
| SNORA17 | 0.069463474 | 0.337715256 |
| PDZRN3-AS1 | 0.100896098 | 0.335565981 |
| MIR624 | 0.092259062 | 0.335553574 |
| CACNG5 | 0.149076035 | 4.022159624 |
| RP11-18H21.2 | 0.027434991 | 0.334417484 |
| AC004837.4 | 0.073086836 | 0.334417405 |
| XXbac-BPG34I8.3 | 0.103309445 | 0.333594435 |
| HRCT1 | 0.149084736 | 1.518779857 |
| RPL17P37 | 0.049198221 | 0.333102178 |
| RP11-222O23.1 | 0.054920609 | 0.332249784 |
| COL4A6 | 0.149093376 | 1.763287093 |
| MIR3940 | 0.018973294 | 0.328963798 |
| RNU7-143P | 0.033129173 | 0.325806283 |
| RP11-123O10.1 | 0.095344092 | 0.325704339 |
| MIR4314 | 0.149667977 | 0.325231565 |
| MAGEC3 | 0.149118044 | 2.939780983 |
| MAPRE1P3 | 0.038189454 | 0.325013516 |
| LRP1B | 0.149292106 | 1.833574376 |
| KIAA0020 | 0.149293984 | 0.769741057 |
| ZFP64P1 | 0.071671945 | 0.324019716 |
| RP11-39K24.14 | 0.094190623 | 0.321818992 |
| TRAJ48 | 0.073053093 | 0.320960402 |
| RNU6-313P | 0.097835306 | 0.320346951 |
| MIR937 | 0.118491049 | 0.319718439 |
| RP11-498M15.1 | 0.024559846 | 0.318959225 |
| AC000081.2 | 0.11208404 | 0.318654545 |
| SNORA26 | 0.016687268 | 0.318543731 |
| IGKV2-28 | 0.121679959 | 0.318356237 |
| LSG1 | 0.14933224 | 0.901287408 |
| AC078794.1 | 0.111277221 | 0.316363178 |
| UBE2J1 | 0.149337519 | 1.091214255 |
| LBX1-AS1 | 0.059170058 | 0.313013124 |
| LINC01038 | 0.142505313 | 0.312657599 |
| RP4-753M9.1 | 0.137731217 | 0.312629984 |
| RNU6-82P | 0.088079068 | 0.312490082 |
| AC002480.2 | 0.032184277 | 0.31205215 |
| RP11-510P12.1 | 0.081408859 | 0.311096584 |
| C1orf61 | 0.149365596 | 0.383778834 |
| RN7SL20P | 0.017475162 | 0.308742406 |
| RP11-433J8.1 | 0.123490417 | 0.307139422 |
| IGHV4-4 | 0.07643171 | 0.306703745 |
| RP11-114M1.1 | 0.101920644 | 0.306505021 |
| AC023271.2 | 0.041778299 | 0.305547867 |
| AC000032.2 | 0.132972673 | 0.305387388 |
| RP11-39K24.13 | 0.111822225 | 0.305374316 |
| RP11-61D1.2 | 0.131583568 | 0.304126415 |
| RNY3P12 | 0.090351276 | 0.302389035 |
| CYP4Z1 | 0.149376686 | 0.469751827 |
| MTRR | 0.149483091 | 1.116636312 |
| SLIT1-AS1 | 0.14505571 | 0.30051472 |
| KLF2P3 | 0.096052824 | 0.298011109 |
| RN7SL121P | 0.035244553 | 0.297800954 |
| RP11-112L18.1 | 0.134302575 | 0.296879081 |
| MED15P8 | 0.148494711 | 0.296473405 |
| GALNT2 | 0.149500012 | 1.283535612 |
| Y_RNA | 0.114162452 | 0.294819766 |
| TSPAN8 | 0.149511407 | 1.979806653 |
| RP11-180I4.1 | 0.136516179 | 0.293379744 |
| ZNF728 | 0.149537584 | 1.621875639 |
| AC067945.2 | 0.119954896 | 0.291223107 |
| AC127383.1 | 0.122765634 | 0.289441434 |
| VDAC1P10 | 0.086645084 | 0.28904036 |
| Y_RNA | 0.135970145 | 0.288927085 |
| RP11-443P15.2 | 0.135669636 | 0.288356212 |
| RP11-424M21.1 | 0.088583084 | 0.287325806 |
| RNA5SP125 | 0.147386554 | 0.286526485 |
| RNU6-35P | 0.039429611 | 0.286156638 |
| AJ239322.1 | 0.133315508 | 0.285206035 |
| CASC23 | 0.07830588 | 0.284559584 |
| AC005606.14 | 0.055814783 | 0.283790813 |
| MIR4436A | 0.104713898 | 0.283614438 |
| RP11-1136G4.1 | 0.036447795 | 0.283258178 |
| RP5-1195D24.1 | 0.134930037 | 0.282457138 |
| RP11-582E3.5 | 0.093149079 | 0.280852562 |
| FAM223A | 0.091911399 | 0.280618863 |
| RN7SL189P | 0.070622 | 0.280387723 |
| RP11-75A9.1 | 0.033546238 | 0.280361338 |
| IL1B | 0.149538108 | 1.62967296 |
| NAP1L4P2 | 0.146466435 | 0.280213006 |
| RNU6-897P | 0.046254912 | 0.27946669 |
| RN7SL252P | 0.099411092 | 0.279193199 |
| AC073464.7 | 0.096363947 | 0.279038842 |
| OR6D1P | 0.12833088 | 0.279017383 |
| RP11-626K17.3 | 0.098316222 | 0.278724863 |
| U6 | 0.125237567 | 0.278493594 |
| AC009960.4 | 0.133618157 | 0.27809588 |
| MIR4308 | 0.148763704 | 0.277636903 |
| RNU6-977P | 0.068631929 | 0.276840731 |
| RP11-384P7.5 | 0.113906273 | 0.276613094 |
| GLRA3 | 0.149559098 | 0.519494321 |
| HNRNPK | 0.149563013 | 1.047009547 |
| XX-DJ76P10__A.2 | 0.07042194 | 0.274393288 |
| ATP5G3 | 0.149565342 | 0.911853222 |
| CTB-33O18.1 | 0.044656844 | 0.271601019 |
| KIF2A | 0.149593928 | 1.112309966 |
| AP000457.2 | 0.120972143 | 0.271158945 |
| RNU7-30P | 0.124326807 | 0.270316269 |
| CTD-2050E21.1 | 0.005270702 | 0.269538069 |
| LA16c-60G3.8 | 0.060477096 | 0.268782747 |
| RP11-63N3.1 | 0.128279227 | 0.267927763 |
| CTD-2507G9.1 | 0.084651821 | 0.265854332 |
| AC012181.1 | 0.027334302 | 0.264935872 |
| AC110620.1 | 0.14573008 | 0.26490297 |
| AC105461.1 | 0.121699844 | 0.264157568 |
| RNU6-238P | 0.012930238 | 0.263907157 |
| NCKAP5 | 0.149645855 | 0.77473834 |
| DGAT2L7P | 0.143664186 | 0.258708244 |
| DXO | 0.149653494 | 1.106751556 |
| RNU7-80P | 0.07685626 | 0.257159975 |
| RNA5SP468 | 0.047153022 | 0.257129212 |
| TRAJ22 | 0.063756867 | 0.255997477 |
| RNU6-857P | 0.035732079 | 0.255835589 |
| LINC01338 | 0.02130763 | 0.254342549 |
| NEK4P1 | 0.141868891 | 0.254050022 |
| RP11-429B14.3 | 0.001732508 | 0.253340442 |
| RNU6-269P | 0.098155782 | 0.25302164 |
| RP1-228H13.2 | 0.002320123 | 0.252839216 |
| RP11-574M7.2 | 0.119920655 | 0.252721433 |
| CTA-299D3.8 | 0.130452787 | 0.252003327 |
| RP3-365I19.2 | 0.083112572 | 0.25196636 |
| LINC01280 | 0.128211704 | 0.250206376 |
| ID3 | 0.149854236 | 1.321755251 |
| RP4-738P15.1 | 0.11118976 | 0.249746413 |
| RP11-165E7.1 | 0.114483671 | 0.248362209 |
| CBWD1 | 0.14986371 | 0.803209089 |
| RNU1-83P | 0.115034573 | 0.247907573 |
| IGKV6D-41 | 0.069017833 | 0.247820834 |
| MKNK2P1 | 0.149249946 | 0.246015322 |
| NIFKP9 | 0.112808287 | 0.245483275 |
| AC034228.4 | 0.098731301 | 0.245426339 |
| REV3L | 0.149866731 | 1.155326639 |
| GIN1 | 0.149873455 | 1.075633566 |
| RNU6-1089P | 0.150602634 | 0.242995884 |
| AC006262.11 | 0.136985018 | 0.242856578 |
| RNA5SP341 | 0.10891702 | 0.24254774 |
| AC004878.8 | 0.105591812 | 0.2419956 |
| AL138963.1 | 0.024842547 | 0.241516611 |
| RNU7-50P | 0.068728377 | 0.240892599 |
| RP11-407A16.5 | 0.149767086 | 0.240487182 |
| RP1-172B20.6 | 0.046687079 | 0.239603901 |
| CYP1D1P | 0.063355976 | 0.239134765 |
| F8A2 | 0.149876595 | 2.508048934 |
| LINC00381 | 0.134087994 | 0.237685857 |
| RP11-9L18.3 | 0.070510102 | 0.237212074 |
| RP11-697H9.5 | 0.016405894 | 0.236312305 |
| SCARNA2 | 0.107012407 | 0.235537889 |
| MIR603 | 0.057356343 | 0.23547236 |
| RP11-522M21.2 | 0.03461956 | 0.234235664 |
| LINC01487 | 0.06788025 | 0.234114354 |
| RPSAP42 | 0.124662613 | 0.233689909 |
| DNA2 | 0.149899497 | 1.122021022 |
| RP11-567N4.3 | 0.110207186 | 0.232821751 |
| OR13K1P | 0.073934253 | 0.232607317 |
| TRAJ14 | 0.140200997 | 0.232586558 |
| AC103564.7 | 0.020604567 | 0.231937043 |
| RAP1GAP | 0.149904918 | 1.461891236 |
| MIR4652 | 0.0764562 | 0.229110545 |
| ELL | 0.149960952 | 0.936254843 |
| SNORA32 | 0.02682657 | 0.228482866 |
| RP11-723G8.2 | 0.113893213 | 0.22730932 |
| DHX35 | 0.149997877 | 0.899873438 |
| IGLVI-70 | 0.056575765 | 0.226392256 |
| AC061992.1 | 0.017156996 | 0.224966314 |
| RPL7AP56 | 0.141077594 | 0.2226409 |
| RP11-397O4.1 | 0.079944687 | 0.222255739 |
| AC006372.5 | 0.118033381 | 0.220454635 |
| RP11-215E13.1 | 0.099665295 | 0.220127302 |
| WNT3 | 0.150040589 | 0.203973963 |
| PCNXL4 | 0.150102837 | 1.103586607 |
| OR4F2P | 0.142388073 | 0.216918501 |
| RP11-415C15.1 | 0.123591814 | 0.214922615 |
| MIR149 | 0.054231728 | 0.214643481 |
| CTD-3022G6.1 | 0.143015965 | 0.214219749 |
| SNORA26 | 0.106960699 | 0.21402376 |
| PPIAP17 | 0.086363799 | 0.213202615 |
| SNORA27 | 0.014243616 | 0.213043737 |
| CTD-2522B17.4 | 0.066695992 | 0.211944814 |
| C11orf30 | 0.150174273 | 1.088828531 |
| DDX51 | 0.15017826 | 0.921683724 |
| RP11-345J4.1 | 0.132572555 | 0.209194289 |
| RP11-232D9.2 | 0.1274593 | 0.208003796 |
| AC005159.1 | 0.073781996 | 0.207564249 |
| RP11-789A21.1 | 0.132367392 | 0.207205887 |
| RNY3P7 | 0.036485847 | 0.206673103 |
| SNORD116-29 | 0.038282281 | 0.205906077 |
| IGHVII-30-1 | 0.064028011 | 0.20580074 |
| RP11-728G15.1 | 0.06605956 | 0.205695297 |
| RN7SL564P | 0.146481609 | 0.20559664 |
| MIR3158-1 | 0.105175827 | 0.205131524 |
| CICP24 | 0.042210407 | 0.204259595 |
| GPR89B | 0.150207579 | 1.086373316 |
| RPS3AP23 | 0.127248859 | 0.203835193 |
| RP11-580I1.2 | 0.055476871 | 0.2035664 |
| AC093668.1 | 0.053933572 | 0.202702811 |
| AP000439.1 | 0.131547008 | 0.199945355 |
| NUMB | 0.150211933 | 1.078072859 |
| TAL2 | 0.150222223 | 1.398467959 |
| FAM205CP | 0.126278331 | 0.198576179 |
| AL590708.1 | 0.069304762 | 0.197525845 |
| RP4-576H24.5 | 0.125348807 | 0.197455034 |
| SNORA40 | 0.084516332 | 0.195757358 |
| MIR1238 | 0.131219717 | 0.195527001 |
| RPL30P5 | 0.086642658 | 0.195061174 |
| MIR1273D | 0.114646803 | 0.191494597 |
| CTD-2503I6.1 | 0.013383359 | 0.191170028 |
| RP11-344B23.2 | 0.117770729 | 0.190590675 |
| CTD-2323K18.3 | 0.065296174 | 0.189926913 |
| OR6L1P | 0.139843289 | 0.187980786 |
| RP11-393K19.1 | 0.146908821 | 0.187874475 |
| RP11-275A14.1 | 0.059938795 | 0.187651093 |
| RN7SL804P | 0.085369397 | 0.18711868 |
| RP11-521H3.3 | 0.081303439 | 0.186925736 |
| RP4-753D10.3 | 0.008412876 | 0.185001322 |
| GUSBP10 | 0.14546181 | 0.184778101 |
| RN7SKP86 | 0.088347415 | 0.183571044 |
| IGHVII-31-1 | 0.129801542 | 0.183466605 |
| ST6GALNAC2P1 | 0.0892261 | 0.18333449 |
| ZFY-AS1 | 0.122905081 | 0.18333099 |
| SNORA40 | 0.129525157 | 0.182007276 |
| RP11-85O21.2 | 0.055893659 | 0.181528067 |
| RP11-321A17.5 | 0.137863557 | 0.180086908 |
| RN7SL272P | 0.052689036 | 0.179966107 |
| AC004911.2 | 0.091483235 | 0.179809777 |
| RBM17P1 | 0.0699369 | 0.178643733 |
| LINC00552 | 0.101723296 | 0.178540341 |
| NUTF2P8 | 0.111775768 | 0.177898546 |
| RN7SL443P | 0.079405906 | 0.176681898 |
| CYCSP6 | 0.018862805 | 0.175725242 |
| MIR29A | 0.141499307 | 0.175573366 |
| CTD-2015A6.1 | 0.123836342 | 0.175139482 |
| ZNF568 | 0.150263916 | 1.305994079 |
| AC009305.2 | 0.074176726 | 0.173129001 |
| SRRD | 0.15030876 | 0.90949913 |
| RP11-62F24.1 | 0.08285871 | 0.172633076 |
| PPP3R2 | 0.15035187 | 1.559152614 |
| RP4-754E20__A.5 | 0.033977601 | 0.168321836 |
| RN7SKP134 | 0.076340538 | 0.16599403 |
| RP11-285B24.1 | 0.127308773 | 0.165717529 |
| Y_RNA | 0.059513426 | 0.165092063 |
| RN7SL51P | 0.047764414 | 0.164839069 |
| RP11-20I6.1 | 0.113964103 | 0.164211425 |
| MIR5196 | 0.090861564 | 0.163151795 |
| RNF214 | 0.150382802 | 1.105647502 |
| FGF7P2 | 0.027446073 | 0.162248755 |
| FDPSP6 | 0.117157807 | 0.16215554 |
| RP11-59O6.3 | 0.125813437 | 0.16183675 |
| RP11-342C20.2 | 0.002345351 | 0.1617193 |
| COTL1P1 | 0.10258427 | 0.161561529 |
| MIR3162 | 0.130495508 | 0.15965758 |
[truncated: 31,955 more chars]
